# Methotrexate Is a JAK/STAT Pathway Inhibitor

**DOI:** 10.1371/journal.pone.0130078

**Published:** 2015-07-01

**Authors:** Sally Thomas, Katherine H. Fisher, John A. Snowden, Sarah J. Danson, Stephen Brown, Martin P. Zeidler

**Affiliations:** 1 The Bateson Centre, and The Department of Biomedical Science, The University of Sheffield, Sheffield, S10 2TN, United Kingdom; 2 Academic Unit of Clinical Oncology, The University of Sheffield, Sheffield, S10 2TN, United Kingdom; 3 Department of Haematology, Royal Hallamshire Hospital, Sheffield, S10 2JF, United Kingdom; 4 Sheffield Experimental Cancer Medicine Centre, Weston Park Hospital, Sheffield, S10 2SJ, United Kingdom; 5 The Sheffield RNAi Screening Facility, Department of Biomedical Science, The University of Sheffield, Firth Court, Sheffield, S10 2TN, United Kingdom; University of East London, UNITED KINGDOM

## Abstract

**Background:**

The JAK/STAT pathway transduces signals from multiple cytokines and controls haematopoiesis, immunity and inflammation. In addition, pathological activation is seen in multiple malignancies including the myeloproliferative neoplasms (MPNs). Given this, drug development efforts have targeted the pathway with JAK inhibitors such as ruxolitinib. Although effective, high costs and side effects have limited its adoption. Thus, a need for effective low cost treatments remains.

**Methods & Findings:**

We used the low-complexity *Drosophila melanogaster* pathway to screen for small molecules that modulate JAK/STAT signalling. This screen identified methotrexate and the closely related aminopterin as potent suppressors of STAT activation. We show that methotrexate suppresses human JAK/STAT signalling without affecting other phosphorylation-dependent pathways. Furthermore, methotrexate significantly reduces STAT5 phosphorylation in cells expressing JAK2 V617F, a mutation associated with most human MPNs. Methotrexate acts independently of dihydrofolate reductase (DHFR) and is comparable to the JAK1/2 inhibitor ruxolitinib. However, cells treated with methotrexate still retain their ability to respond to physiological levels of the ligand erythropoietin.

**Conclusions:**

Aminopterin and methotrexate represent the first chemotherapy agents developed and act as competitive inhibitors of DHFR. Methotrexate is also widely used at low doses to treat inflammatory and immune-mediated conditions including rheumatoid arthritis. In this low-dose regime, folate supplements are given to mitigate side effects by bypassing the biochemical requirement for DHFR. Although independent of DHFR, the mechanism-of-action underlying the low-dose effects of methotrexate is unknown. Given that multiple pro-inflammatory cytokines signal through the pathway, we suggest that suppression of the JAK/STAT pathway is likely to be the principal anti-inflammatory and immunosuppressive mechanism-of-action of low-dose methotrexate. In addition, we suggest that patients with JAK/STAT-associated haematological malignancies may benefit from low-dose methotrexate treatments. While the JAK1/2 inhibitor ruxolitinib is effective, a £43,200 annual cost precludes widespread adoption. With an annual methotrexate cost of around £32, our findings represent an important development with significant future potential.

## Introduction

JAK/STAT signalling is an evolutionarily conserved pathway that transduces signals from growth factors and cytokines and is required for both development and adult homeostasis. In the canonical JAK/STAT pathway, multiple ligands, including pro-inflammatory cytokines such as IL-2, IL-6 and IL-12, bind to transmembrane receptors. This association leads to the activation of associated Janus Kinases (JAKs), which tyrosine phosphorylate both themselves and intracellular residues of their receptors. This creates binding sites for Signal Transducers and Activators of Transcription (STATs) which are then themselves phosphorylated by JAKs converting them to an active form that translocates to the nucleus and activates transcription (reviewed in [1,2]).

In addition to essential developmental roles, the pathway is required for adult haematopoiesis, inflammation and immune responses. In addition, inappropriate activation of the pathway is associated with the pathogenesis of a multiple malignancies and inflammatory diseases [3–5]. Such pathological activation of JAK/STAT signalling is a particular feature of myeloproliferative neoplasms (MPNs). Evidence from multiple studies has shown that 95% of patients with polycythaemia vera (PV) and 40–60% of patients with essential thrombocytosis and primary myelofibrosis have a gain-of-function V617F mutation in JAK2 [6–9]. Furthermore, this mutation has also been shown to cause erythrocytosis in a mouse model which is rescued by deletion of STAT5a/b, suggesting that JAK2 mediated STAT5 activation plays a key role in these disorders [10].

Given its roles in human disease, the JAK/STAT signalling pathway represents an appealing drug target. Indeed, the discovery of JAK2 V617F mutations in 2005 has already led to the development of the JAK1/2 inhibitor ruxolitinib. Strikingly, this kinase inhibitor has been developed, trialled and approved and is already an established treatment for both primary and secondary myelofibrosis [11,12]. Effective in reducing spleen volume and able to substantially improve quality of life, ruxolitinib has recently been shown to prolong life [13] and also shows promise in PV clinical trials [14]. However, despite this evidence of clinical effectiveness, ruxolitinib use has not been approved by the UK National Institute for Health and Care Excellence (NICE) on the grounds of cost effectiveness, a decision that reflects its £43,200 per annum cost [15].

**Fig 1:**
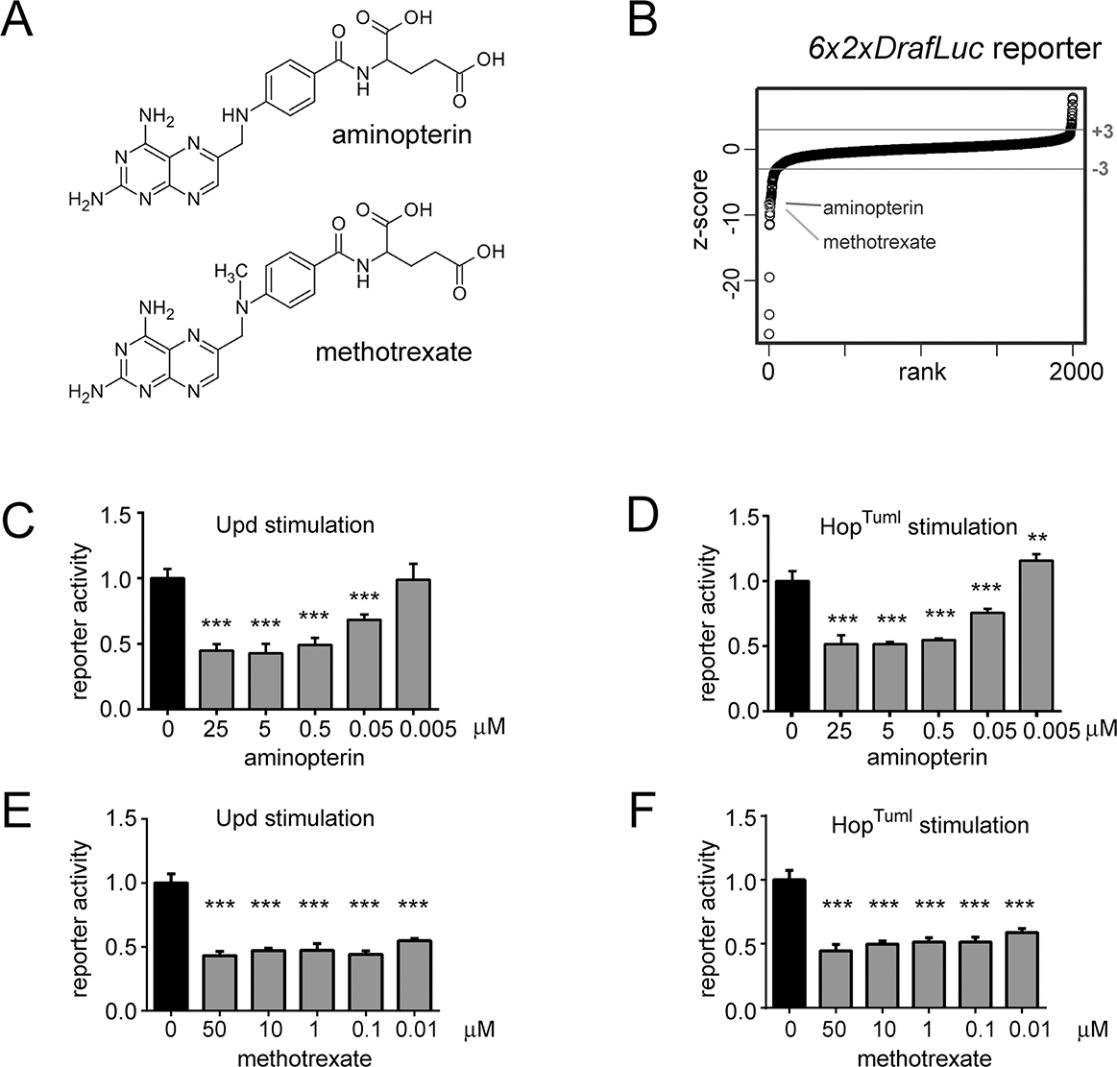
Identification of methotrexate and aminopterin as suppressors of the *Drosophila* JAK/STAT pathway. A) The chemical structures of methotrexate (top) and aminopterin (bottom). B) A rank plot of the compounds screened using the *6x2xDrafLuc* reporter system. Compounds are ranked according to the z-scores of their interactions with a score >3 indicating a significant increase in activity and an score <-3 indicating a significant suppression. The position of aminopterin and methotrexate are indicated. C-D) Normalised *6x2xDrafLuc* transcriptional reporter activity in *Drosophila* Kc_167_ cells treated with the indicated concentrations of aminopterin and stimulated with the pathway ligand Unpaired (Upd) (C) or by transfection with the gain-of-function JAK mutant Hop^Tuml^ (D). E-F) Normalised *6x2xDrafLuc* transcriptional reporter activity in *Drosophila* Kc_167_ cells treated with the indicated concentrations of methotrexate and stimulated with Upd or Hop^Tuml^. In graphs reporter activity has been normalised to carrier control (0 μM drug) and error bars show the standard deviation of four independent experiments. *** = p < 0.001, ** = p < 0.01, * = p < 0.05.

First developed as a folate analogue, aminopterin and the chemically very similar methotrexate (Fig 1A) are some of the first chemotherapy agents to have been used clinically [16]. Acting as competitive inhibitors of dihydrofolate reductase (DHFR), enzymatic inhibition reduces intracellular levels of downstream folate pathway intermediates required for nucleotide synthesis. This results in impaired DNA replication and repair so slowing cellular proliferation and ultimately leading to cell death [17]. Methotrexate is still used in a number of chemotherapy regimes to treat acute leukaemias and lymphomas. However, its most widespread use is as a first line treatment for a range of inflammatory diseases including rheumatoid arthritis, Crohn’s disease and psoriasis [18]. In these diseases, methotrexate is typically administered at a level only 1/100^th^ of that used for chemotherapy and efficacy is not thought to be mediated by the modulation of folate metabolism. Rather, any DHFR enzymatic block is routinely bypassed by the prescription of folate supplements, including folinic acid–a downstream metabolite that reduces adverse side effects but does not affect anti-inflammatory effectiveness [19]. However, although methotrexate has been approved for the treatment of inflammatory disease for over 35 years, the mechanism-of-action of low-dose methotrexate remains unclear. Links to cellular adenosine release, intercellular adhesion and T-cell apoptosis have all been suggested [20]. However, a mechanism directly linking the drug to inflammatory pathways remains elusive.

In this report we describe the identification of aminopterin and methotrexate as potent, specific and folate-independent suppressors of constitutive JAK/STAT activation. We find that cells treated with methotrexate retain the capacity to activate the pathway in response to stimulation with ligands. Furthermore, these effects occurred at drug concentrations already routinely prescribed for rheumatoid arthritis patients. We suggest that our results identify a novel mechanism of action for methotrexate that may explain its anti-inflammatory and immune suppressive activity. Furthermore, we suggest that further investigation and clinical trials of methotrexate as a therapeutic agent in MPNs and other haematological malignancies featuring ectopically activated JAK/STAT signalling may be justified.

## Methods

### Drug screening in *Drosophila* cells

*Drosophila* Kc_167_ cells [21] were cultured under standard growth conditions in Schneiders Insect Media (Gibco), supplemented with 10% FBS and 1% penicillin/streptomycin at 25°C. (Previously described in [22]).

The Spectrum Collection Library (Microsource Discovery Systems), provided at 2.5mM in dimethyl sulphoxide (DMSO), was aliquoted and formatted into 25x 96 deep well plates using a Hamilton Star Robot. Columns 1 and 12 were left empty for controls as shown in S1 Fig panel A Controls included DMSO-only negative controls and the human JAK2 inhibitor AG490 (Invitrogen; [23]) used as a positive control at a final concentration of 9 and 25μM (S1 Fig panel A and C).

On the day of screening, 1μl of each compound was diluted 1:250 in cell culture media. Controls were spiked into columns 1 and 12. These mother plates were stored at 4°C until needed. Once cells were prepared for screening, screening plates were made by aliquoting 10μl from each mother plate into 4x replicate daughter plates. Final compound concentrations were 1μM in 0.04% DMSO.

On the day prior to screening, 40 million *Drosophila* Kc_167_ cells were seeded into 15x T75 flasks and allowed to recover overnight. Cells were then transfected with plasmids required by the STAT92E-reporter assay as previously described [24,25]. Per flask: *6x2xDrafLuc* (4μg), *pAc-Renilla* (2μg), *pAc-UpdGFP* (4.8μg), *pAc* empty vector (5.2μg). Cells were then incubated for 6hrs before 50,000 cells per well were aliquoted in 90μl media across all screening plates containing compounds and controls, and left to incubate for 3d at 25°C.

Luciferase assays were adapted from those previously described [24,25]. In brief, media was removed from the plate by tipping, cells were lysed in 40μl per well lysis buffer for 10min, before adding 60μl buffer containing the substrate for firefly luciferase (D-luciferin; Apollo Scientific), and measuring activity immediately on a Varioskan Plate reader (Thermo Fisher). After all 25 plates were read for firefly luciferase, 60μl buffer containing substrate for *Renilla* luciferase (Coelenterazine; Apollo Scientific) was added and measured, utilising a 500nm long-pass filter. Each replicate set of plates was assayed in turn.

Raw luciferase units were analysed using the CellHTS2 R/Bioconductor package as previously described [24]. Robust z-scores were calculated using z = median/MAD, where MAD is the median absolute deviation. The median and MAD are calculated for a whole plate on a plate-by-plate basis. These robust z-scores for ratio (FL/RL) values were considered significant above 3 or below -3.

RNAi screens were performed and analysed as in [24].

### Human cell lines and cell culture

HDLM-2 cells and HEL cells were obtained from the DSMZ German Collection of Microorganisms and Cell Cultures (Leibniz Institute). The HDLM-2 line was derived from a pleural effusion in a 74 year old man with stage IV Nodular sclerosis classical Hodgkin lymphoma. The HEL cell line was originally derived from peripheral blood from a 30 year old man who had relapsed acute myeloid leukaemia (WHO AML classification acute erythroid leukaemia). The patient had previously been treated for Hodgkin Lymphoma. HDLM-2 cells were seeded at 1 x 10^6^ cells/ml in RPMI 1640 + GlutaMAX medium (Life Technologies) supplemented with 20% heat-inactivated Foetal Bovine Serum (FBS), 100 units/ml penicillin and 100μg/ml streptomycin. HEL cells were seeded at 0.5 x 10^6^ cells/ml in RPMI 1640 + GlutaMAX medium supplemented with 10% FBS, 100 units/ml penicillin and 100μg/ml streptomycin. All cells were grown in a 37°C humidified incubator with 5% CO_2_.

#### Drug and ligand treatments

Methotrexate and aminopterin (Sigma) and ruxolitinib (Cayman Chemical) were dissolved in 100% DMSO. All treated cells experienced a final concentration of DMSO 1:1000 volume:volume in addition to the dissolved drugs. 1:1000 DMSO containing no drugs was used as a control treatment. Leucovorin (calcium folinate, Refolinon) was obtained from Pharmacia as a 3mg/ml solution for injection and used at a final concentration of 0.3μg/ml. This concentration was chosen to correspond to peak serum folate concentrations measured after oral administration [26]. Human recombinant erythropoietin (EPO; Sigma) was used at a final concentration of 15ng/ml. This concentration was chosen to correspond to serum EPO levels measured in secondary erythrocytosis [27,28] since these are likely to represent the levels of EPO produced in response to physiological stimuli.

HDLM-2 and HEL cells were grown for 24 hours prior to the application of DMSO alone, methotrexate, aminopterin or Ruxolitinib, and continued to grow for a further 48 hours before protein extraction. Where cells were treated with calcium folinate this was added 24 hours after methotrexate. Where cells were stimulated with EPO this was added 20 minutes before protein extraction.

### Western blotting

Cell pellets were suspended in lysis buffer containing 50mM Tris-HCL pH 7.4, 250mM NaCl, 5mM EDTA, 0.3% Triton X 100 and protease inhibitor. Lysates were then boiled in Laemmli buffer consisting of 75mM Tris-HCL pH 6.8, 10% glycerol, 2% SDS, 0.0025% bromophenol blue and 2.5% β-Mercaptoethanol. Samples were loaded onto polyacrylamide gels, separated by electrophoresis and transferred to nitrocellulose membranes. Membranes were blocked in 5% dried skimmed milk in Tris buffered saline (pH 7.4) supplemented with 0.1% Tween 20 (TBST) for 30 minutes, then incubated in primary antibody in 5% Bovine Serum Albumen (BSA) in TBST overnight at 4°C. Membranes were incubated in horseradish peroxidase conjugated secondary antibodies (Dako) at a dilution of 1:10,000, and developed using ECL reagent (GE Healthcare). All western blots were performed at least three times using protein extract from independent repeats of the cell culture experiments. Image-J software was used to quantify band intensities on western blots.

### Antibodies

Anti β-Actin was obtained from Abcam and used at a dilution of 1:2000. Anti phospho-JAK1 (Tyr 1022/1023) was obtained from Millipore and used at a dilution of 1:1000. All other antibodies—JAK1, JAK2, STAT1, STAT3, STAT5, phospho-JAK2 (Tyr 1007/1008), phospho-STAT1 (Tyr 701), phospho-STAT3 (Tyr 705), phospho-STAT5 (Tyr 694), phospho-c-Jun (Ser 73), phospho-ERK1/2 (Erk1 Thr 202 and Tyr 204, Erk2 Thr 185 and Tyr 187) and phospho-Akt (Thr 308)—were obtained from Cell Signalling Technology and used at a dilution of 1:1000.

### Statistical analysis

To calculate Western blot band intensities, values were corrected for loading based on β-actin levels then normalised to the value of the DMSO-treated lanes. All blots were repeated on three independent samples. Statistical analysis was carried out using GraphPad Prism software. One-way ANOVA and Dunnett’s multiple comparison test were used to compare groups.

## Results

### Screening for inhibitors of JAK/STAT signalling

To identify regulators of JAK/STAT pathway signalling in the low complexity *Drosophila* system, we used the transcriptional reporter *6x2xDrafLuc* to screen a library of 2000 small molecules consisting of FDA-approved drugs, agrochemicals, cytotoxic chemicals and pure natural products [29]. This screen was undertaken in four independent experimental replicates using *Drosophila* Kc_167_ cells by the Sheffield RNAi Screening Facility using standard approaches previously developed by the Zeidler lab for RNAi screening (S1 Fig; [25]). The complete data set was analysed following best bioinformatic practice [24] and is presented in S1 Table. Considering z-scores of >3 or <-3 as significant, we independently identified aminopterin and the closely related methotrexate (Fig 1A) as strong inhibitors of the *Drosophila* JAK/STAT pathway (methotrexate z-score -8.72; aminopterin z-score -8.20; Fig 1B). We next verified the screen results with new samples of compounds generated independently of the original library and found that suppression of *6x2xDrafLuc* reporter activity by aminopterin is dose dependent and is observed following activation of the pathway both by over expression of the *Drosophila* JAK/STAT ligand Unpaired (Upd) (Fig 1C) and by expression of a constitutively active allele of the *Drosophila* JAK (termed *hop* ^*Tum-l*^) (Fig 1D; [30]). In addition, methotrexate is also a strong suppressor of reporter activity following pathway stimulation by both Upd and *hop* ^*Tum-l*^ (Fig 1E and 1F). As such, both methotrexate and aminopterin appear to be potent inhibitors of the *Drosophila* JAK/STAT pathway following stimulation both by natural ligands and gain-of-function JAK alleles analogous to human disease-associated JAK2 mutants.

### Methotrexate and aminopterin reduce JAK/STAT pathway activity in human cells

**Fig 2:**
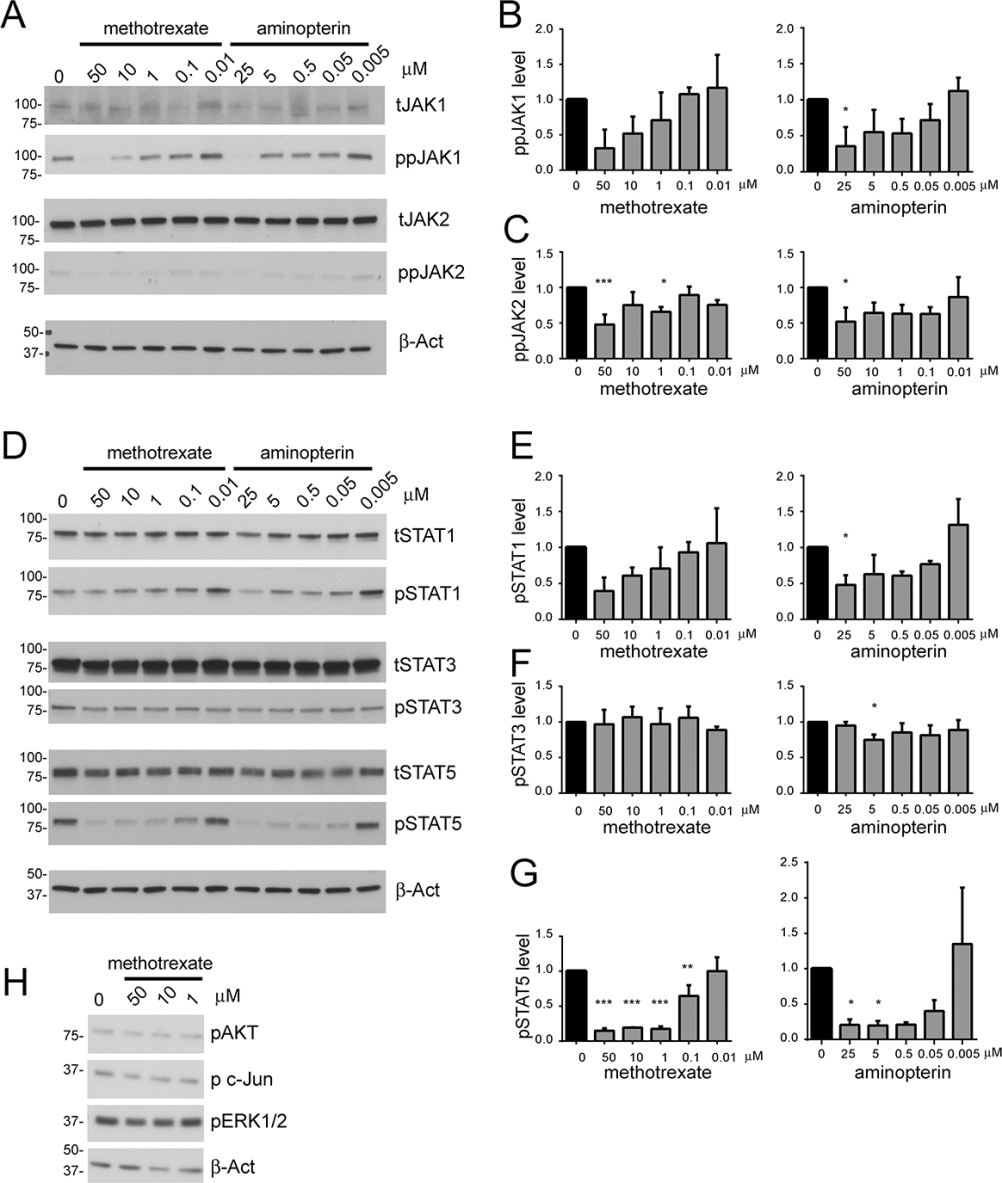
The effect of methotrexate on the constitutively active JAK/STAT pathway present in human HDLM-2 cells. A) HDLM-2 cells treated with the indicated concentrations of methotrexate and aminopterin and blotted to show levels of total JAK1 (tJAK1), dual phosphorylated JAK1 (ppJAK1), total JAK2 (tJAK2), dual phosphorylated JAK2 (ppJAK2) and ß-actin shown as a loading control. Levels of ppJAK1 and ppJAK2 are reduced at high drug concentrations. B-C) Quantification of ppJAK1 (B) and ppJAK2 (C) following treatment with methotrexate and aminopterin. D) HDLM-2 cells treated with the indicated concentrations of methotrexate and aminopterin and blotted to show levels of total STAT1 (tSTAT1), phosphorylated STAT1 (pSTAT1), total STAT3 (tSTAT3), phosphorylated STAT3 (pSTAT3), total STAT5 (tSTAT5), phosphorylated STAT5 (pSTAT5) and β-actin shown as a loading control. Levels of pSTAT1 and pSTAT5 are reduced at most drug concentrations while levels of pSTAT3 appear to be unaffected. E-G) Quantification of pSTAT1 (E), pSTAT3 (F) and pSTAT5 (G) following treatment with methotrexate and aminopterin. H) Human HDLM-2 cells treated with the indicated concentrations of methotrexate and blotted to show levels of phosphorylated AKT (pAKT), phosphorylated c-Jun (p c-Jun), phosphorylated ERK1/2 (pERK1/2) and β-actin shown as a loading control. The levels of each phospho-protein tested appears to be unaffected by methotrexate. In the representative western blots shown the position and apparent molecular weights of markers used are indicated in kDa. For graphs levels have been normalised to carrier control (0μM drug) and error bars show the standard deviation of three independent experiments. *** = p < 0.001, ** = p < 0.01, * = p < 0.05.

Given that methotrexate and aminopterin suppress STAT92E dependant transcription in *Drosophila* cells we investigated whether these drugs affect the conserved JAK/STAT pathway present in human cells. The Hodgkin lymphoma cell line HDLM-2 has been demonstrated to show constitutive phosphorylation of JAK1 and JAK2, and STAT1, STAT3, STAT5 and STAT6 [31]. It has also been previously used to examine the effects of small molecules upon JAK/STAT signalling [32]. In HDLM-2 cells constitutive phosphorylation of JAK1 was present in cells treated with drug vehicle alone but clearly reduced by both methotrexate and aminopterin in a dose-dependent manner (Fig 2A and 2B) while total JAK1 levels were not affected. While less striking, methotrexate also appeared to affect the lower levels of JAK2 phosphorylation that can just be detected in these cells (Fig 2A and 2C).

The principal physiological substrates of the JAK kinases are the STATs and all STATs contain an invariant C-terminal tyrosine residue phosphorylation of which is absolutely essential for activity. We therefore used phospho-specific antibodies able to specifically recognise C-terminal STAT tyrosine phosphorylation to report pathway activation (see Methods for details). As expected, both aminopterin and methotrexate produce a dose-responsive reduction of both STAT1 and STAT5 phosphorylation (Fig 2D, 2E and 2G) in HDLM-2 cells. By contrast levels of pSTAT3 are not substantially altered in these cells (Fig 2F). These effects are not due to a reduction in overall STAT levels or changes in cell number as illustrated by the ß-actin loading control.

Although consistent with *Drosophila* data, in order for the effect of methotrexate on human JAK/STAT signalling to be potentially clinically useful, suppression of STAT phosphorylation must occur at drug concentrations achievable in patients. When methotrexate is given intravenously in chemotherapy regimes, plasma concentrations peak at around 50μM [33]. Following oral administration of low- dose methotrexate for the treatment of rheumatoid arthritis the peak plasma concentration of methotrexate is around one hundred times lower at 0.4–0.8μM [34]. Although care must be taken when comparing *ex vivo* and *in vivo* levels, we observe strong suppression of STAT5 phosphorylation at 1μM drug concentrations, levels approximately equivalent to those seen in patients taking low-dose oral methotrexate.

In order to exclude the possibility that inhibition of STAT phosphorylation could be the result of a more general non-specific effect on intracellular protein phosphorylation we examined the effect of methotrexate on a number of additional phosphorylated proteins. Examination of Akt, cJun and ERK1/2, all of which are constitutively activated in HDLM-2 cells, showed that phosphorylation of these proteins was unaffected by methotrexate, even at the highest concentrations examined (Fig 2H). This suggests that the associated cellular signalling pathways are unlikely to be directly affected by methotrexate and supports the contention that the interaction of methotrexate with the JAK/STAT signalling pathway is likely to be specific and not a more general effect on protein phosphorylation or cellular homeostasis.

### Methotrexate reduces MPN-associated pathway signalling

Although HDLM-2 cells have been used in multiple studies because of their endogenous pathway activation, the basis of this activation is not clear and is unlikely to reflect physiological signalling. As an alternative, we also sought to examine whether methotrexate might suppress ectopic JAK/STAT signalling as found in MPNs. We therefore examined its effects on JAK/STAT pathway activation in HEL cells, which are homozygous for the JAK2 V617F gain-of-function mutation [9]. These cells show constitutive phosphorylation of STAT5, an activity that is dependent on JAK2 [35].

**Fig 3:**
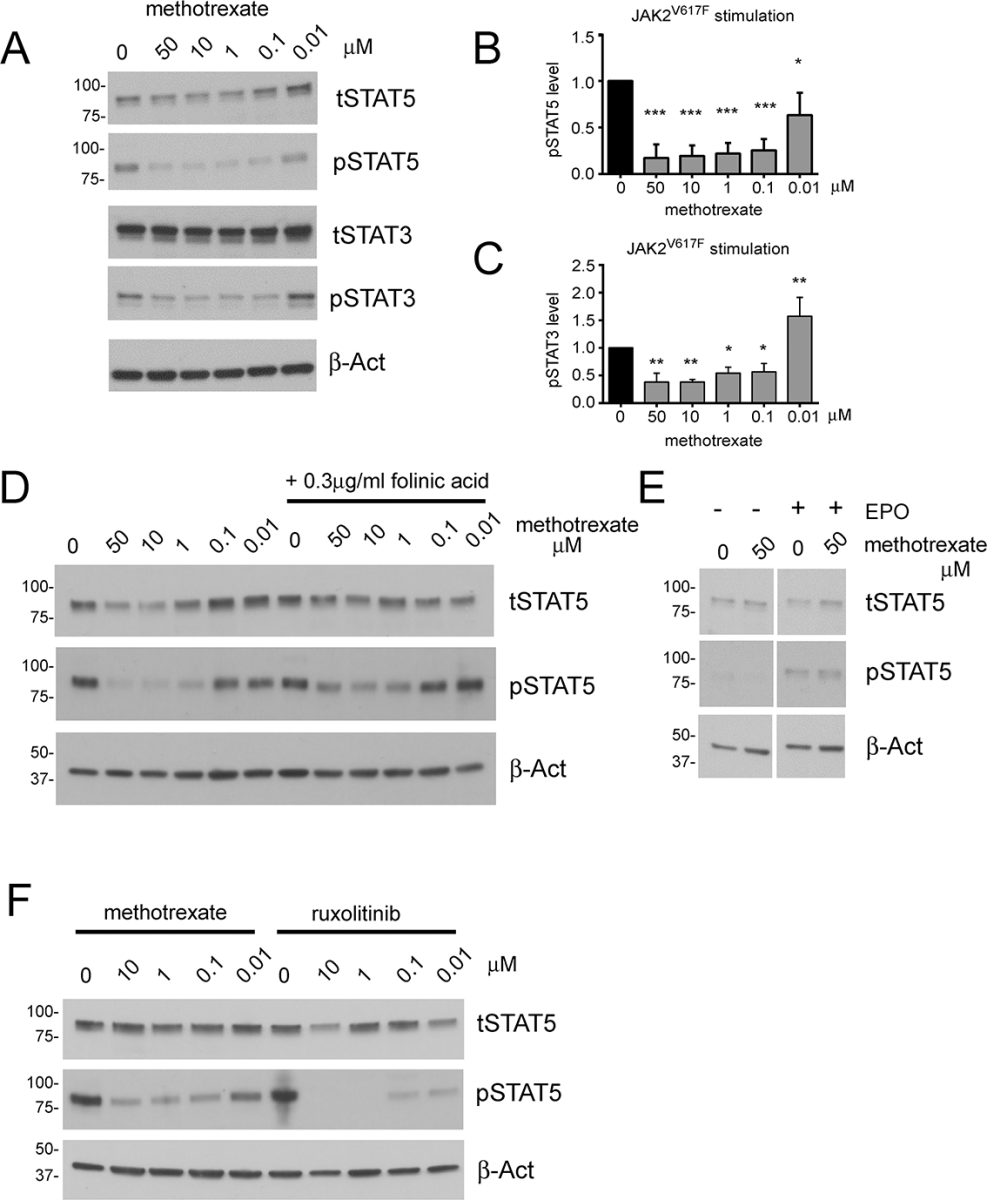
The effect of methotrexate on human HEL cells homozygous for the activating JAK2 V617F mutation. A) HEL cells treated with the indicated concentrations of methotrexate and blotted to show levels of total STAT5 (tSTAT5), phosphorylated STAT5 (pSTAT5), total STAT3 (tSTAT3), phosphorylated STAT3 (pSTAT3) and β-actin shown as a loading control. Levels of pSTAT5 and pSTAT3 are clearly reduced at most drug concentrations. B & C) Quantification of pSTAT5 (B) and pSTAT3 (C) levels present in HEL cells treated with the indicated concentrations of methotrexate. D) HEL cells treated with the indicated concentrations of methotrexate and blotted to show levels of total STAT5 (tSTAT5), phosphorylated STAT5 (pSTAT5) and β-actin shown as a loading control. Indicated lanes have been grown in the presence of 0.3μg/ml folinic acid. Suppression of pSTAT5 levels is seen at most drug concentrations. E) HEL cells treated with the indicated concentrations of methotrexate and blotted to show levels of total STAT5 (tSTAT5), phosphorylated STAT5 (pSTAT5) and β-actin shown as a loading control. Right hand lanes have also been treated with 15ng/ml recombinant human erythropoietin (EPO). Blots have been underexposed to show the difference in pSTAT5 levels and so are not comparable to other panels. F) HEL cells treated with the indicated concentrations of methotrexate or ruxolitinib and blotted to show levels of total STAT5 (tSTAT5), phosphorylated STAT5 (pSTAT5) and β-actin shown as a loading control. Levels of pSTAT5 are reduced at most drug concentrations. In the representative western blots shown the position and apparent molecular weights of markers used are indicated in kDa. For graphs levels have been normalised to carrier control (0μM drug) and error bars show the standard deviation of three independent experiments. *** = p < 0.001, ** = p < 0.01, * = p < 0.05.

We find that methotrexate is also able to reduce levels of STAT3 and STAT5 phosphorylation in HEL cells (Fig 3A)–an interaction that occurs over a concentration range comparable to previous results in HDLM-2 cells. This inhibition is statistically significant for both STAT5 and STAT3 following the quantification of multiple independent experiments (Fig 3B and 3C). In addition, although methotrexate appears to produce a mild reduction in total STAT3 and STAT5 levels, this effect is not statistically significant.

As already observed for constitutively active *Drosophila* JAK (Fig 1F), methotrexate is also able to suppress the constitutive pathway activation mediated by human JAK2 V617F. Furthermore, this effect is elicited by levels of methotrexate comparable to those found in the serum of rheumatoid arthritis patients.

### The effect of methotrexate on STAT phosphorylation is not reversed by folinic acid

Methotrexate exerts its effects as a chemotherapy agent via competitive inhibition of DHFR so leading to an impairment of folate metabolism [17]. To establish whether the effects of methotrexate on JAK/STAT signalling might be linked to DHFR inhibition we examined the effect of folinic acid on STAT phosphorylation in methotrexate treated cells. Folate supplements, including folinic acid are used to alleviate the side effects of low-dose methotrexate in rheumatoid arthritis patients and act by bypassing the enzymatic activity of DHFR [19]. We find that the ability of methotrexate to suppress STAT5 phosphorylation persisted in the presence of 0.3μg/ml folinic acid (Fig 3D)—a high concentration of folinic acid representative of that measured in patient plasma following methotrexate overdose treatment [36]. The ability of methotrexate to inhibit JAK/STAT pathway activation even in the presence of folinic acid is consistent with *Drosophila* RNAi results. In these experiments, the *6x2xDrafLuc* reporter is not affected by RNAi-mediated knockdown of multiple biosynthetic enzymes within the folate pathway, a result which suggests that reduced folate pathway activity is not sufficient to inhibit the *Drosophila* JAK/STAT pathway (S2 Table).

However, while methotrexate still suppresses pSTAT5 activation in the presence of folinic acid, the magnitude of the suppression is reduced. While 50μM methotrexate produces an approximately five-fold reduction in STAT5 phosphorylation, the same concentration only results in a two-fold pSTAT5 reduction in the presence of folinic acid (Fig 3D). Although yet to be definitively proven, the attenuation observed may be the result of competition for the cellular folate transporters responsible for the uptake of these closely related molecules [37].

### Methotrexate-treated cells can respond to EPO

The JAK/STAT pathway is essential for multiple developmental and physiological processes including haematopoiesis and immunity. As a result, thrombocytopaenia, anaemia and susceptibility to infection have been significant side effects of JAK inhibitors used in clinical practice [11,38]. If methotrexate were to be used clinically to treat patients with pathway-associated diseases such as MPNs it would be desirable for the suppression of JAK/STAT signalling to occur in such a way that inhibition can be overcome by physiological stimuli. To test whether this was the case for methotrexate-induced pathway suppression we stimulated methotrexate-treated HEL cells with recombinant erythropoietin (EPO). To recapitulate EPO levels produced by the physiological stimulus of hypoxia we used an EPO concentration calculated to correspond to that measured in the serum of individuals with secondary erythrocytosis [28]. As previously shown, STAT5 phosphorylation is strongly reduced by 50μM of methotrexate, however this effect was reversed following EPO stimulation such that pSTAT5 levels were rescued to levels comparable to that observed in control cells even in the presence of methotrexate (Fig 3E).

### Methotrexate is comparable to ruxolitinib

Ruxolitinib has been recently developed as a specific JAK1/2 inhibitor and has been extensively trialled for the treatment of JAK2 V617F-associated MPNs [39]. We therefore wished to determine whether the effects of methotrexate on STAT phosphorylation in HEL cells were comparable to the effects of ruxolitinib. As expected, both ruxolitinib and methotrexate treatment resulted in a clear suppression of STAT5 phosphorylation (Fig 3F). Comparing peak serum concentrations measured in patients taking the drugs orally [0.4–0.8μM for methotrexate and 1μM for ruxolitinib [34,40]], ruxolitinib produced a more profound suppression of STAT5 phosphorylation than methotrexate (Fig 3F). However the effects of both drugs on STAT5 phosphorylation were comparable at physiologically realistic concentrations.

## Discussion

In this study we show that methotrexate suppresses activation of the JAK/STAT signalling pathway, including JAK/STAT activation caused by JAK2 V617F. We suggest that our results have implications for understanding the basic biology of methotrexate and propose that low-dose methotrexate treatments may provide a low cost and effective treatment option for a number of novel patient groups.

### Methotrexate in inflammatory disease

In addition to its continuing use as a chemotherapy drug, low-dose methotrexate has also been used for many years to treat a range of inflammatory disorders including rheumatoid arthritis, Crohn’s disease and psoriasis [19,41]. However, its mechanism of action in these conditions is not fully understood with links to cellular adenosine release, intercellular adhesion and T-cell apoptosis having all been suggested [20]. Our data shows that methotrexate is also able to suppress JAK/STAT pathway signalling and STAT phosphorylation at concentrations equivalent to those measured in the plasma of patients. Indeed, clear pathway suppression is observed at concentrations analogous to both chemotherapy doses and those taking methotrexate at the much lower levels prescribed for rheumatoid arthritis [33,34]. Therefore, although care must be taken when comparing experiments in cell culture to drug concentrations in patients, our results suggest that methotrexate is likely to suppress JAK/STAT activation *in vivo*.

Recently, it has been shown that the JAK/STAT signalling pathway plays an important role in the development and resolution of inflammation [3,4]. Indeed, the JAK/STAT pathway is responsible for the transduction of multiple pro-inflammatory cytokines and has been shown to contribute to disease pathogenesis in rheumatoid arthritis [42]. Given this role, considerable drug development efforts have focused on targeting the JAK/STAT pathway. This includes the development of tocilizumab, an antibody based inhibitor of the receptor bound by the pro-inflammatory IL-6 [43], and tofacitinib, a specific inhibitor of JAK3 which has recently shown efficacy in clinical trials [38].

Given the role played by the JAK/STAT pathway in inflammatory processes, considered together with the efficacy of methotrexate in treating rheumatoid arthritis-associated inflammation, our data suggests that suppression of JAK/STAT activation may represent the mechanism of action by which low-dose methotrexate moderates inflammatory conditions.

### Potential for methotrexate in JAK/STAT-associated disease

The suppression of constitutive STAT phosphorylation by methotrexate suggests that methotrexate may benefit patient groups for whom JAK/STAT activation plays a role in pathogenesis. These potentially include patients with fusions of JAK2 with PCM1, ETV6 and BCR [44], T-cell large granular lymphocytic leukaemia [45], chronic lympho-proliferative disorders of natural killer cells [46], Waldenstrom’s Macroglobulinaemia [47], chronic myeloid leukaemia [48] and chronic lymphocytic leukaemia [49]. Indeed, low-dose methotrexate is already used for the treatment of large granular lymphocytic leukaemia, which is associated with activating mutations in STAT3, where its effectiveness may result at least partly from its capacity to suppress JAK/STAT pathway activation [50]. However, the largest group of diseases in which the ectopic activation of the JAK/STAT pathway has been identified are the JAK2 V617F positive MPNs. Identified in around 95% of patients with polycythaemia vera and 40–60% of patients with essential thrombocytosis and primary myelofibrosis [6–9], the identification of this gain-of-function mutation has revolutionised MPN diagnostics and has led directly to the development of multiple JAK kinase inhibitors [39]. Currently the best developed of these is the JAK1/2 inhibitor ruxolitinib. Ruxolitinib has recently shown to reduce symptoms and improve survival in myelofibrosis patients [11,13], a striking contrast to other treatments for myelofibrosis that may be no better than placebo [51]. However, despite evidence of clinical effectiveness, ruxolitinib use has not been approved by the UK agency NICE on the grounds of cost effectiveness [15]. Given that the £43,200 per annum cost of ruxolitinib [15] compares to an annual drug cost for low-dose methotrexate of around £32, we suggest that methotrexate may represent an alternative treatment option for this disease by delivering many of the clinical benefits of JAK/STAT inhibition at a substantially reduced cost.

### Mechanism of action of methotrexate

Although clearly effective as a JAK/STAT inhibitor *in vitro*, and effective as an anti-inflammatory and immunosuppressant *in vivo*, the molecular mechanism via which methotrexate inhibits JAK/STAT pathway signalling remains unclear. Our data suggests that the effect of methotrexate on JAK/STAT signalling is at least partially independent of its effects on folate metabolism, as suppression of STAT phosphorylation persists in the presence of folinic acid. Furthermore, this independence is supported by results from RNAi screens where there is no interaction between multiple enzymes in the folate biosynthetic pathway and STAT transcriptional activity (S2 Table). Rather, we suggest that the attenuation of methotrexate effectiveness by folinic acid may be a consequence of reduced intracellular concentrations of drug since both methotrexate and folinic acid enter cells via the same transporter [41].

An additional factor of relevance to the action of methotrexate is the ability of drug-treated cells to activate their JAK/STAT pathway signalling in response to physiological levels of ligand stimulation (Fig 3E). Consistent with this, we have also found that a short incubation with methotrexate does not reduce ligand stimulated STAT phosphorylation in CD4+ cells, B cells and monocytes obtained from peripheral blood (Dr. Pia Isomäki and Prof Olli Silvennoinen, personal communication). Given our results, and the ability of rheumatoid arthritis patients to tolerate low-dose methotrexate over many years, we suggest that methotrexate may ‘dampen’ the pathological over-activation of the JAK/STAT pathway sufficiently to control disease without preventing physiological activation when needed for haematopoiesis or infection response. Furthermore, given that the levels of STAT5 phosphorylation in CD34+ cells from patients with MPNs are only about 1.5 fold greater than in healthy individuals [35], it is possible that a relatively mild long-term suppression of pathway activation may be sufficient to control the disease. This is also important in the context of the effects of ruxolitinib, which produces a more profound inhibition of STAT phosphorylation (Fig 3F), but for which thrombocytopaenia, and to a lesser extent anaemia and susceptibility to infection, are significant side effects [11,38].

In conclusion, our results indicate that methotrexate suppresses JAK/STAT signalling and suggest that this suppression may explain the effectiveness of low-dose methotrexate treatments currently used as a first line treatment for inflammatory diseases such as rheumatoid arthritis. In addition, we propose that low dose methotrexate may represent a promising treatment for patients with MPNs and other haematological malignancies associated with inappropriate pathway activation. In this context, we feel that the established safety, dosing regimes and cost-effectiveness of methotrexate make it a particularly attractive candidate worthy of further investigation. Undertaking clinical trials for the efficacy of methotrexate in haematological malignancies associated with activated JAK/STAT mutations has the potential to revolutionise the treatment of this large class of chronic disease and may ultimately represent a new, financially attractive treatment option.

## Supporting Information

S1 Fig Screening for regulators of *Drosophila* JAK/STAT signalling. A) 96-well plate layout used for screening included 80 samples (yellow) as well as negative controls (blues) and positive controls (greens) containing the JAK inhibitor AG490. Final concentrations of DMSO are indicated. B) Flow chart illustrating the plasmids and screening protocol used. See Methods for details. C) The z-scores of DMSO negative controls and AG490 positive controls from the screened plates indicate that the *Drosophila* JAK/STAT pathway reporter is sensitive to suppression of JAK kinase activity. **** = p<0.0001, NS = not significant
(PDF)

S1 Table The effect of the small molecule Spectrum Library on *6x2xDrafLuc* JAK/STAT pathway reporter activity. Table showing the details of the plates screened. Columns show: compound ID, Molecular name, plate number well position, content, CAS number, formula, molecular weight, reported bioactivity, source, approval status, references, and the calculated z-scores for the three replicates of the screen (Rep1, Rep2, Rep3, Rep4). The position of empty wells, wells treated with the positive control AG490 and wells containing the negative control DMSO are indicated for each plate.
(PDF)

S2 Table Knockdown of folate pathway enzymes do not affect *Drosophila* JAK/STAT pathway activity. Mean *6x2xDrafLuc* JAK/STAT pathway reporter activity (expressed as z-scores) following knockdown of the indicated *Drosophila* genes and stimulation with the three ligands Unpaired, Unpaired2 and Unpaired3. Knockdown of enzymes central to the folate, purine and thymidine synthesis pathways do not interact significantly in any assay while knockdown of *hopscotch*, the *Drosophila* JAK homologue, produces a strong and consistent reduction in activity. Each screen was carried out in triplicate and scores shown are the mean of three replicates. Scores above +3 or below -3 are considered significant. Human homologues and their enzymatic activities are shown.
(PDF)
