# Supplementary material for: Methotrexate Is a JAK/STAT Pathway Inhibitor
Source: PLoS One. 2015 Jul 1;10(7):e0130078. doi: 10.1371/journal.pone.0130078 (PMC4489434; doi:10.1371/journal.pone.0130078)
Supplement: S1 Fig — A) 96-well plate layout used for screening included 80 samples (yellow) as well as negative controls (blues) and positive controls (greens) containing the JAK inhibitor AG490. Final concentrations of DMSO are indicated. B) Flow chart illustrating the plasmids and screening protocol used. See Methods for details. C) The z-scores of DMSO negative controls and AG490 positive controls from the screened plates indicate that the Drosophila JAK/STAT pathway reporter is sensitive to suppression of JAK kinase activity. **** = p<0.0001, NS = not significant (PDF) [file pone.0130078.s001.pdf]

S1 Figure

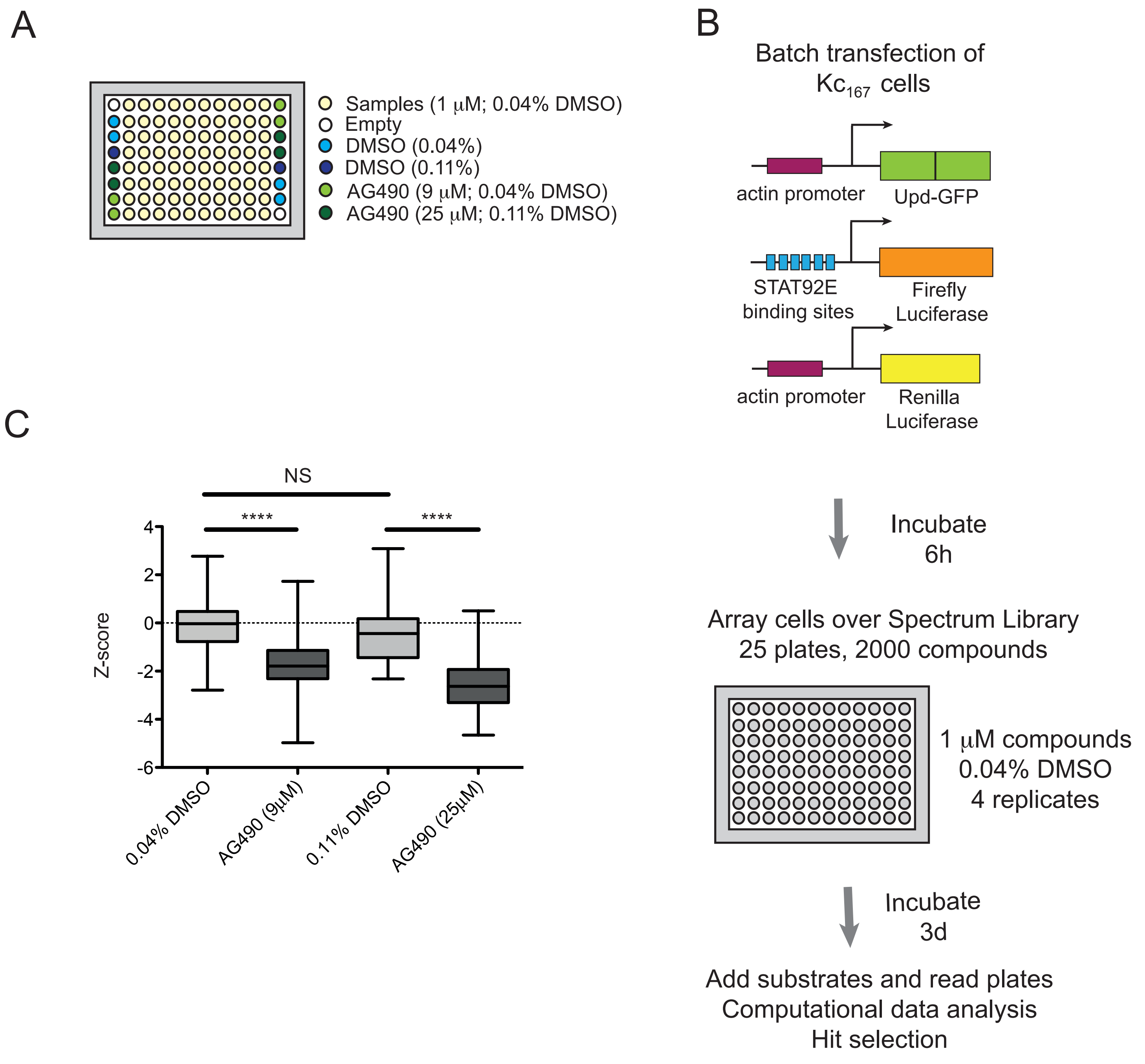

Screening for regulators of *Drosophila* JAK/STAT signalling
A) 96-well plate layout used for screening included 80 samples (yellow) as well as negative controls (blues) and positive controls (greens) containing the AG490 JAK inhibitor. Final concentrations of DMSO are indicated.
B) Flow chart illustrating the plasmids and screening protocol used. See Methods for details.
C) The z-scores of DMSO negative controls and AG490 positive controls from the screened plates indicate that the system is sensitive to suppression of JAK kinase activity. ****=p<0.0001, NS = not significant
