# Supplementary material for: Methotrexate Is a JAK/STAT Pathway Inhibitor
Source: PLoS One. 2015 Jul 1;10(7):e0130078. doi: 10.1371/journal.pone.0130078 (PMC4489434; doi:10.1371/journal.pone.0130078)
Supplement: S1 Table — Table showing the details of the plates screened. Columns show: compound ID, Molecular name, plate number well position, content, CAS number, formula, molecular weight, reported bioactivity, source, approval status, references, and the calculated z-scores for the three replicates of the screen (Rep1, Rep2, Rep3, Rep4). The position of empty wells, wells treated with the positive control AG490 and wells containing the negative control DMSO are indicated for each plate. (PDF) [file pone.0130078.s002.pdf]

S1 Table

												Screen Score (z-score)				
Compound ID	MolNAME	plate	position	Content	cas#	Formula	MolWt	Bioactivity	Source	Status	Reference	rep1	rep2	rep3	rep4	Average
	empty	100122-01	A01	sample								NA	NA	NA	NA	NA
01500372	MAFENIDE HYDROCHLORIDE	100122-01	A02	sample	138-39-6	C7H11ClN2O2S	222.69492	antibacterial	synthetic	USAN, INN, BAN		0.28	-1.37	-0.82	-2.36	-1.07
01500373	MAPROTILINE HYDROCHLORIDE	100122-01	A03	sample	10347-81-6, 10262-69-8	C20H24ClN	313.87398	antidepressant	synthetic	USAN, INN, BAN		1.47	-1.74	-0.91	-0.61	-0.45
01500374	MECAMYLAMINE HYDROCHLORIDE	100122-01	A04	sample	826-39-1, 60-40-2	C11H22ClN	203.75769	antihypertensive	synthetic	USP, INN, BAN		0.88	4.29	-0.06	-0.38	1.18
01500375	MECHLORETHAMINE	100122-01	A05	sample	55-86-7, 51-75-2	C5H11Cl2N	156.05612	antineoplastic, alkylating agent	synthetic	USP, INN, BAN, JAN		-0.95	-1.74	-2.12	-1.98	-1.70
01500376	MECLIZINE HYDROCHLORIDE	100122-01	A06	sample	31884-77-2, 1104-22-9	C25H29Cl3N2	463.88228	antiemetic	synthetic	USP, INN, BAN, JAN		-0.32	-2.98	1.68	-1.87	-0.87
01500377	MECLOFENAMATE SODIUM	100122-01	A07	sample	6385-02-0	C14H10Cl2NNaO2	318.1371	antiinflammatory, antipyretic	synthetic	USP		0.38	-1.38	-1.05	-0.96	-0.75
01500380	MEDRYSONE	100122-01	A08	sample	2668-66-8	C22H32O3	344.49854	glucocorticoid	semisynthetic	USAN, INN		0.78	-1.19	-1.10	-0.47	-0.50
01500381	MEGESTROL ACETATE	100122-01	A09	sample	595-33-5, 3562-63-8	C24H32O4	384.52024	progestogen, antineoplastic	semisynthetic	USP, INN, BAN		-1.75	-2.51	-1.01	0.85	-1.11
01500382	MELPHALAN	100122-01	A10	sample	148-82-3	C13H18Cl2N2O2	305.20661	antineoplastic, alkylating agent	synthetic	USP, INN, BAN, JAN		-0.09	-0.74	0.03	-1.24	-0.51
01500388	MESTRANOL	100122-01	A11	sample	72-33-3	C21H26O2	310.44017	estrogen, with progesterone as oral contraceptive	semisynthetic	USP, INN, BAN, JAN		1.56	-2.14	-1.84	-1.27	-0.93
	0.04% JAK2inhib	100122-01	A12	0.04% jak2inhib								0.98	-3.38	1.79	-1.91	-0.63
	0.04% DMSO	100122-01	B01	0.04% dmso								-0.65	-1.33	-1.07	-1.61	-1.17
01500390	METAPROTERENOL	100122-01	B02	sample	586-06-1, 5874-97-5	C11H17NO3	211.26304	bronchodilator	synthetic	USP, JAN		-0.60	-0.91	0.30	2.05	0.21
01500391	METHACHOLINE CHLORIDE	100122-01	B03	sample	62-51-1, 55-92-5	C8H18ClNO2	195.69116	cholinergic, diagnostic aid	synthetic	USP, INN, BAN		0.43	0.36	1.17	-0.60	0.34
01500396	METHIMAZOLE	100122-01	B04	sample	60-56-0	C4H6N2S	114.16982	antihyperthyroid	synthetic	USP, INN, BAN, JAN		0.48	1.30	0.84	6.65	2.32
01500397	METHOCARBAMOL	100122-01	B05	sample	532-03-6	C11H15NO5	241.2459	muscle relaxant (skeletal)	synthetic	USP, INN, BAN, JAN		0.12	2.37	-0.22	-0.95	0.33
01500398	METHOTREXATE(+/-)	100122-01	B06	sample	60388-53-6	C20H22N8O5	454.44894	antineoplastic, antirheumatic, folic acid antagonist	synthetic; AMETHOPTERIN	USP, INN, BAN, JAN		-8.26	-9.36	-8.49	-8.77	-8.72
01500399	METHOXAMINE HYDROCHLORIDE	100122-01	B07	sample	61-16-5, 390-28-3	C11H18ClNO3	247.72401	alpha1 adrenoreceptor agonist, vasoconstrictor	synthetic	USP-XXII, INN, BAN, JAN		-1.43	-1.83	-0.45	-0.97	-1.17
01500403	METHYLDOPA	100122-01	B08	sample	41372-08-1, 555-30-6	C10H13NO4	211.21941	antihypertensive	synthetic	USP, INN, BAN, JAN		-0.35	0.13	-0.26	2.17	0.42
01500406	METHYLPREDNISOLONE	100122-01	B09	sample	83-43-2	C22H30O5	374.4814	glucocorticoid	semisynthetic	USP, INN, BAN, JAN		0.73	-0.59	-0.02	2.31	0.61
01500410	METOCLOPRAMIDE HYDROCHLORIDE	100122-01	B10	sample	54143-57-6, 7232-21-5	C14H23Cl2N3O2	336.26431	antiemetic	synthetic	USP, INN, BAN, JAN		0.04	-0.36	-0.30	0.01	-0.15
01500411	METOPROLOL TARTRATE	100122-01	B11	sample	56392-17-7, 37350-58-6	C19H31NO9	417.46022	antihypertensive, antianginal	synthetic	USP, JAN		-0.51	0.90	2.77	-0.01	0.79
	0.04% JAK2inhib	100122-01	B12	0.04% jak2inhib								-1.80	-2.00	-1.44	-1.48	-1.68
	0.04% DMSO	100122-01	C01	0.04% dmso								-1.93	-1.31	-2.32	4.04	-0.38
01500412	METRONIDAZOLE	100122-01	C02	sample	443-48-1, 69198-10-3	C6H9N3O3	171.15693	antiprotozoal	synthetic	USP, INN, BAN, JAN		-0.67	-0.09	-0.70	-1.46	-0.73
01500414	MINOCYCLINE HYDROCHLORIDE	100122-01	C03	sample	13614-98-7, 10118-90-8	C23H28ClN3O7	493.94851	antibacterial	semisynthetic	USAN, INN, BAN		-0.49	0.27	-0.30	-0.49	-0.25
01500415	MINOXIDIL	100122-01	C04	sample	38304-91-5	C9H15N5O	209.2528	antihypertensive, antialopecia agent	synthetic	USP, INN, BAN		0.35	0.54	-0.80	0.91	0.25
01500418	MOXALACTAM DISODIUM	100122-01	C05	sample	8031-09-2	C20H18N6Na2O9S	564.44486	antibacterial	semisynthetic	USAN, INN, BAN		-0.50	-0.01	-1.55	0.43	-0.41
01500419	NADIDE	100122-01	C06	sample	53-84-9	C21H27N7O14P2	663.43544	alcohol and narcotic antagonist	synthetic	USAN, INN, BAN, JAN		-0.15	3.17	-0.25	-0.45	0.58
01500420	NAFCILLIN SODIUM	100122-01	C07	sample	7177-50-6, 985-16-0	C21H21N2NaO5S	436.46572	antibacterial	semisynthetic	USP, INN, BAN		-0.26	4.97	-0.17	0.02	1.14
01500422	NALOXONE HYDROCHLORIDE	100122-01	C08	sample	357-08-4, 51481-60-8	C19H22ClNO4	363.84449	narcotic antagonist	synthetic	USP, INN, BAN, JAN	Brain Res 839:209 (1999); Brit J Pharmacol 127:605 (1999)	-0.06	0.01	0.33	0.37	0.16
01500424	NAPHAZOLINE HYDROCHLORIDE	100122-01	C09	sample	550-99-2, 835-31-4	C14H15ClN2	246.74205	adrenergic agonist, nasal decongestant	synthetic	USP, INN, BAN, JAN		-0.54	-0.16	2.17	0.11	0.40
01500425	NAPROXEN(+)	100122-01	C10	sample	22204-53-1	C14H14O3	230.26588	antiinflammatory, analgesic, antipyretic	synthetic	USP, INN, BAN, JAN		-0.72	0.44	-0.01	-0.06	-0.09
01500428	NEOSTIGMINE BROMIDE	100122-01	C11	sample	114-80-7, 59-99-4	C12H19BrN2O2	303.20143	cholinergic	synthetic	USP, INN, BAN, JAN		-0.29	-0.17	1.55	0.16	0.31
	0.11% JAK2inhib	100122-01	C12	0.11% jak2inhib								-4.94	-4.14	-3.27	-2.11	-3.61
	0.11% DMSO	100122-01	D01	0.11% dmso								-0.50	5.57	1.57	5.72	3.09
01500430	NIACIN	100122-01	D02	sample	59-67-6	C6H5NO2	123.11225	antihyperlipidemic, vitamin (enzyme cofactor)	widespread in the plant and fungal kingdom	USP, INN, JAN		1.29	0.18	1.83	3.66	1.74
01500431	NIFEDIPINE	100122-01	D03	sample	21829-25-4	C17H18N2O6	346.34281	antianginal, antihypertensive	synthetic	USP, INN, BAN, JAN		0.24	-0.08	-0.94	-0.99	-0.44
01500433	NITROFURANTOIN	100122-01	D04	sample	67-20-9, 54-87-5 [nitrofurantoin	C8H6N4O5	238.16082	antibacterial	synthetic	USP, INN, BAN, JAN		1.59	-1.95	-1.18	-1.67	-0.80
01505399	OXYBUTYNIN CHLORIDE	100122-01	D05	sample	1508-65-2	C22H32ClNO3	393.95824	anticholinergic	synthetic; MJ-4309-1, 5058	USP, JAN		1.65	-0.89	-2.25	-0.40	-0.47
01500436	NOREPINEPHRINE	100122-01	D06	sample	69815-49-2, 51-40-1	C8H11NO3	169.18177	adrenergic agonist, antihypotensive	synthetic	USP, INN, BAN		-0.31	0.94	0.26	5.84	1.68
01500437	NORETHINDRONE	100122-01	D07	sample	68-22-4	C20H26O2	298.42902	progestogen	synthetic	USP, INN, BAN, JAN		-0.41	-0.81	2.93	-0.06	0.42
01500439	NORETHYNODREL	100122-01	D08	sample	68-23-5	C20H26O2	298.42902	progestogen, in combination with estrogen as oral contraceptive	synthetic	USP		0.63	0.34	0.77	1.24	0.75
01500440	NORFLOXACIN	100122-01	D09	sample	70458-96-7	C16H18FN3O3	319.33856	antibacterial	synthetic	USP, INN, BAN, JAN		2.72	0.42	-0.26	-0.31	0.64
01500441	NORGESTREL	100122-01	D10	sample	6533-00-2	C21H28O2	312.45611	progestogen	synthetic	USP, INN, BAN, JAN		0.23	-0.77	0.64	2.91	0.75
01500442	NORTRIPTYLINE	100122-01	D11	sample	894-71-3, 72-69-5	C19H21N	263.38592	antidepressant	synthetic	USP, INN, BAN, JAN		-0.80	-1.22	0.17	-0.07	-0.48
	0.11% JAK2inhib	100122-01	D12	0.11% jak2inhib								-1.68	-4.14	-3.04	-2.86	-2.93
	0.11% JAK2inhib	100122-01	E01	0.11% jak2inhib								-3.42	-3.75	-3.75	-2.32	-3.31
01500443	NOSCAPINE HYDROCHLORIDE	100122-01	E02	sample	912-60-7, 128-62-1 [noscapine]	C22H24ClNO7	449.89208	antitussive	Corydalis cava and Papaver spp	USP, INN, BAN, JAN	Tetrahedron Letters 1965: 669	-0.20	1.23	-0.93	-0.10	0.00
01500444	NOVOBIOCIN SODIUM	100122-01	E03	sample	1476-53-5, 303-81-1	C31H35N2NaO11	634.6212	antibacterial	Streptomyces niveus and S griseus	USP		-0.59	-1.17	-0.93	-1.48	-1.04
01500445	NYLIDRIN HYDROCHLORIDE	100122-01	E04	sample	849-55-8	C19H26ClNO2	335.87757	vasodilator (peripheral)	synthetic	USP-XII, INN, BAN		1.32	1.43	-0.22	0.42	0.74
01500446	NYSTATIN	100122-01	E05	sample	114-90-9	C47H75NO17	926.1183	antifungal, binds to membrane sterols	Streptomyces noursei	USP, INN, BAN, JAN		-4.46	0.15	0.36	0.68	-0.82
01500447	ORPHENADRINE CITRATE	100122-01	E06	sample	4682-36-4, 83-98-7	C24H31NO8	461.51657	muscle relaxant (skeletal), antihistaminic	synthetic	USP, INN, BAN		1.74	0.44	1.18	-0.05	0.83
01500448	OXACILLIN SODIUM	100122-01	E07	sample	7240-38-2, 1173-88-2	C19H18N3NaO5S	423.42621	antibacterial	semisynthetic	USP, INN, BAN, JAN		-0.52	2.03	0.45	5.71	1.92
01500451	OXYBENZONE	100122-01	E08	sample	131-57-7	C14H12O3	228.24994	ultraviolet screen	synthetic	USP, INN		0.58	0.40	0.17	-0.34	0.20
01500453	OXYMETAZOLINE HYDROCHLORIDE	100122-01	E09	sample	2315-02-8, 1491-59-4	C16H25ClN2O	296.84345	adrenergic agonist, nasal decongestant	synthetic	USP, INN, BAN, JAN		0.83	-0.38	0.01	-0.29	0.04
01500455	OXYPHENBUTAZONE	100122-01	E10	sample	7081-38-1, 129-20-4	C19H20N2O3	324.38285	antiinflammatory	synthetic	USP-XXIII, INN, BAN		0.54	0.96	1.13	0.57	0.80
01500457	OXYTETRACYCLINE	100122-01	E11	sample	6153-64-6, 79-57-2	C22H25ClN2O9	496.90555	antibacterial	Streptomyces rimosus	USP, INN, BAN, JAN		3.08	-1.03	0.44	-0.33	0.54
	0.11% DMSO	100122-01	E12	0.11% dmso								-1.77	-1.56	-0.73	6.46	0.60
	0.11% JAK2inhib	100122-01	F01	0.11% jak2inhib								-2.79	-2.92	-2.46	-3.42	-2.90
01500459	PAPAVERINE HYDROCHLORIDE	100122-01	F02	sample	61-25-6, 58-74-2 [papaverine]	C20H22ClNO4	375.85564	muscle relaxant (smooth), cerebral vasodilator	Papaver somniferum, Rauwolfia serpentina	USP, BAN, JAN		-0.98	0.04	-1.75	-0.77	-0.87
01500460	PARACHLOROPHENOL	100122-01	F03	sample	106-48-9	C6H5ClO	128.55915	topical antibacterial (topical)	synthetic	USP		0.46	1.68	4.32	0.73	1.80
01500462	PARGYLINE HYDROCHLORIDE	100122-01	F04	sample	306-07-0, 555-57-7 [pargyline]	C11H14ClN	195.69393	antihypertensive	synthetic	USAN, INN, BAN		2.02	1.44	0.76	4.50	2.18
01500464	PENICILLAMINE	100122-01	F05	sample	52-67-5	C5H11NO2S	149.21292	chelating agent (Cu), antirheumatic	semisynthetic	USP, INN, BAN, JAN		0.77	0.18	-0.14	0.39	0.30
01500472	PHENACEMIDE	100122-01	F06	sample	63-98-9	C9H10N2O2	178.19225	anticonvulsant	synthetic	USP-XIII, INN, BAN		0.19	-0.27	0.02	0.27	0.05
01500473	PHENAZOPYRIDINE HYDROCHLORIDE	100122-01	F07	sample	136-40-3, 94-78-0	C11H12ClN5	249.70479	analgesic	synthetic	USP, INN, BAN		2.51	1.28	0.87	0.96	1.41
01500476	PHENELZINE SULFATE	100122-01	F08	sample	156-51-4, 51-71-8 [phenelzine]	C8H14N2O4S	234.27578	antidepressant	synthetic	USP, INN, BAN		0.36	0.94	1.00	1.11	0.85
01500477	PHENINDIONE	100122-01	F09	sample	83-12-5	C15H10O2	222.24575	anticoagulant	synthetic	USP-XXII, INN, BAN		-0.37	-1.00	0.24	0.29	-0.21
01500478	PHENIRAMINE MALEATE	100122-01	F10	sample	132-20-7, 86-21-5	C20H24N2O4	356.42528	antihistaminic	synthetic	USP, INN, BAN		0.01	0.20	0.68	0.17	0.27
01500482	PHENYLBUTAZONE	100122-01	F11	sample	50-33-9	C19H20N2O2	308.38345	antiinflammatory	synthetic	USP, INN, BAN, JAN		-0.27	-0.28	0.75	-0.54	-0.09
	0.04% DMSO	100122-01	F12	0.04% dmso								-1.10	-0.61	-0.05	1.20	-0.14
	0.04% JAK2inhib	100122-01	G01	0.04% jak2inhib								-0.88	-2.77	0.20	-2.83	-1.57
01500485	PHENYTOIN SODIUM	100122-01	G02	sample	630-93-3, 57-41-0 [phenytoin]	C15H11N2NaO2	274.25692	anticonvulsant, antieleptic	synthetic	USP, JAN		-1.30	-0.75	-0.39	-1.57	-1.00
01501010	FENOFIBRATE	100122-01	G03	sample	49562-28-9	C20H21ClO4	360.84097	antihyperlipidemic	synthetic	USAN, INN, BAN		0.32	-1.29	-0.86	-0.29	-0.53
01501011	FENOPROFEN	100122-01	G04	sample	31879-05-7	C15H14O3	242.27703	antiinflammatory	synthetic	USAN, INN, BAN		0.47	0.06	0.28	-0.24	0.14
01501015	FLUFENAMIC ACID	100122-01	G05	sample	530-78-9	C14H10F3NO2	281.2365	antiinflammatory, analgesic	synthetic	USAN, INN, BAN, JAN		-0.01	-0.23	0.13	5.94	1.46
01501016	FENBENDAZOLE	100122-01	G06	sample	43210-67-9	C15H13N3O2S	299.35376	anthelmintic	synthetic	USAN, INN, BAN		1.28	0.51	0.67	0.32	0.70
01501021	FENSPIRIDE HYDROCHLORIDE	100122-01	G07	sample	5053-08-7, 5053-06-5	C15H21ClN2O2	296.79982	antiinflammatory, bronchodilator	synthetic	USAN, INN		-0.96	0.08	-0.31	0.21	-0.25
01501103	MEFENAMIC ACID	100122-01	G08	sample	61-68-7	C15H15NO2	241.2923	antiinflammatory, analgesic	synthetic	USP, INN, BAN, JAN		-0.10	-0.77	-0.54	0.41	-0.25
01501104	METHACYCLINE HYDROCHLORIDE	100122-01	G09	sample	3963-95-9, 914-00-1	C22H23ClN2O8	478.89021	antibacterial	semisynthetic	USAN, INN, BAN		0.13	4.44	0.32	0.32	1.30
01501108	MEFEXAMIDE	100122-01	G10	sample	1227-61-8	C15H25ClN2O3	316.8311	stimulant (central)	synthetic	USAN, INN		-0.80	0.29	-0.30	0.62	-0.05
01501109	PROBUCOL	100122-01	G11	sample	23288-49-5	C31H48O2S2	516.85501	antihyperlipidemic	synthetic	USP, INN, BAN, JAN		-0.40	2.65	-0.43	-0.23	0.40
	0.04% DMSO	100122-01	G12	0.04% dmso								-0.23	-0.27	0.15	1.07	0.18
	0.04% JAK2inhib	100122-01	H01	0.04% jak2inhib								0.18	-2.88	-1.54	-3.26	-1.88
01501105	PUROMYCIN HYDROCHLORIDE	100122-01	H02	sample	58-58-2, 53-79-2 [puromycin]	C22H31Cl2N7O5	544.44227	antineoplastic, antiprotozoal	Streptomyces alboniger	USAN, INN, BAN		-7.85	-4.85	-7.96	-8.97	-7.41
01501110	MEBENDAZOLE	100122-01	H03	sample	31431-39-7	C16H13N3O3	295.30031	anthelmintic	synthetic	USP, INN, BAN, JAN		2.54	4.10	3.04	-0.74	2.24
01501115	NALBUPHINE HYDROCHLORIDE	100122-01	H04	sample	23277-43-2, 20594-83-6	C21H28ClNO4	393.91461	analgesic, narcotic antagonist	synthetic	USAN, INN, BAN		0.39	0.71	-0.68	-0.21	0.06
01501119	PROGLUMIDE	100122-01	H05	sample	6620-60-6	C18H26N2O4	334.41892	anticholinergic	synthetic	USAN, INN, BAN, JAN		-0.06	-0.16	0.23	-0.11	-0.02
01501120	MINAPRINE HYDROCHLORIDE	100122-01	H06	sample	25953-17-7, 25905-77-5	C17H24Cl2N4O	371.31303	psychotropic	synthetic	USAN		0.35	-0.75	-0.55	2.57	0.41
01501121	MEMANTINE HYDROCHLORIDE	100122-01	H07	sample	19982-08-2	C12H22ClN	215.76884	muscle relaxant (skeletal)	synthetic	USAN		-4.80	-0.16	0.10	0.05	-1.20
01501127	ATENOLOL	100122-01	H08	sample	29122-68-7	C14H22N2O3	266.34304	beta adrenergic blocker	synthetic	USP, INN, BAN, JAN		-0.18	0.27	0.33	0.56	0.25
01501129	CARBETAPENTANE CITRATE	100122-01	H09	sample	23142-01-0, 77-23-6	C26H39NO10	525.60143	antitussive	synthetic	NF-XIII, INN, BAN		-0.79	1.82	-0.07	0.44	0.35
01501134	PIMOZIDE	100122-01	H10	sample	2062-78-4	C28H29F2N3O	461.55963	antipsychotic	synthetic	USP, INN, BAN, JAN		0.62	-0.16	0.07	0.87	0.35
01501135	NICARDIPINE HYDROCHLORIDE	100122-01	H11	sample	54527-84-3, 55985-32-5	C26H30ClN3O6	515.9985	vasodilator	synthetic	USAN, INN, BAN, JAN		-0.39	-0.48	0.15	0.12	-0.15
	empty	100122-01	H12	sample								NA	NA	NA	NA	NA
	empty	100122-02	A01	sample								NA	NA	NA	NA	NA
01501137	NEFOPAM	100122-02	A02	sample	23327-57-3, 13669-70-0	C17H19NO	253.34708	analgesic	synthetic	USAN, INN, BAN		-0.28	-0.02	3.33	-0.48	0.64
01501138	PIRENZEPINE HYDROCHLORIDE	100122-02	A03	sample	29868-97-1, 28797-61-7	C19H23Cl2N5O2	424.33346	antiulcer	synthetic	USAN, INN, BAN, JAN		0.13	0.48	0.00	-2.43	-0.46
01501139	PRAMOXINE HYDROCHLORIDE	100122-02	A04	sample	637-58-1, 140-65-8	C17H28ClNO3	329.87061	anesthetic (topical)	synthetic	USAN, INN, BAN		-0.97	-0.74	-1.03	-1.58	-1.08
01501140	MEPHENESIN	100122-02	A05	sample	59-47-2	C10H14O3	182.22128	muscle relaxant (skeletal)	synthetic	NF-XII, INN, BAN		-1.17	-1.69	-0.17	-0.77	-0.95
01501142	SULFACHLORPYRIDAZINE	100122-02	A06	sample	80-32-0	C10H9ClN4O2S	284.72583	antibacterial	synthetic	USP, INN, BAN		0.77	-1.31	0.13	-0.86	-0.32
01501144	SULFADIMETHOXINE	100122-02	A07	sample	122-11-2	C12H14N4O4S	310.33378	antibacterial	synthetic	USP, INN, BAN, JAN		0.70	-0.56	0.00	-0.17	-0.01
01501146	SULFAGUANIDINE	100122-02	A08	sample	57-67-0	C7H10N4O2S	214.24735	antibacterial	synthetic	NF-XI, INN, BAN		-1.20	-0.59	-0.77	3.70	0.28
01501147	SULFAMONOMETHOXINE	100122-02	A09	sample	1220-83-3	C11H12N4O3S	280.30729	antibacterial	synthetic	USAN, INN, BAN, JAN		-1.54	-1.10	-0.79	-0.73	-1.04
01501148	SULCONAZOLE NITRATE	100122-02	A10	sample	61318-91-0, 61318-90-9	C18H16Cl3N3O3S	460.76952	antifungal	synthetic	USP, INN, BAN, JAN		-3.98	-2.48	-2.26	-1.35	-2.52
01501149	RITODRINE HYDROCHLORIDE	100122-02	A11	sample	23239-51-2, 26652-09-5	C17H22ClNO3	323.82279	muscle relaxant (smooth)	synthetic	USAN, INN, BAN		0.44	-0.49	0.03	0.15	0.03
	0.04% JAK2inhib	100122-02	A12	0.04% jak2inhib								-3.93	-3.30	-2.30	-1.59	-2.78
	0.04% DMSO	100122-02	B01	0.04% dmso								-0.38	-0.27	-1.05	-2.79	-1.12
01501150	SULPIRIDE	100122-02	B02	sample	15676-16-1	C15H23N3O4S	341.43226	dopamine receptor antagonist, antipsychotic	synthetic	USAN, INN, BAN, JAN		0.53	0.19	-0.07	-0.80	-0.04
01501151	RANITIDINE	100122-02	B03	sample	66357-35-5	C13H22N4O3S	314.40929	H2 antihistamine	synthetic	USAN, INN, BAN		-0.02	0.53	0.02	-1.78	-0.31
01501153	SULOCTIDIL	100122-02	B04	sample	54063-56-8	C20H35NOS	337.57205	peripheral vasodilator	synthetic	USAN, INN, BAN	hepatotoxic at clinical dose (600mg)	0.60	0.02	-0.03	-1.28	-0.17
01501154	RONIDAZOLE	100122-02	B05	sample	7681-76-7	C6H8N4O4	200.15506	antiprotozoal	synthetic	USAN, INN, BAN		-0.04	1.75	1.55	-0.76	0.63
01501155	SULFAMETER	100122-02	B06	sample	651-06-9	C11H12N4O3S	280.30729	antibacterial	synthetic	USAN, INN, BAN		-1.58	0.33	-0.31	0.05	-0.38
01501156	SULFAMETHOXYPYRIDAZINE	100122-02	B07	sample	80-35-3	C11H12N4O3S	280.30729	antibacterial	synthetic	USP-XVII, INN, BAN		-0.22	-0.89	-0.21	0.10	-0.31
01501161	SUPROFEN	100122-02	B08	sample	40828-46-4	C14H12O3S	260.31394	antiinflammatory	synthetic	USP, INN, BAN, JAN		-1.66	-0.61	-1.13	2.38	-0.26
01501171	SACCHARIN	100122-02	B09	sample	81-07-2	C7H5NO3S	183.1868	sweetener	synthetic	USAN, NF		-1.05	-0.44	-0.99	-0.94	-0.85
01501173	ACETANILIDE	100122-02	B10	sample	103-84-4	C8H9NO	135.16703	analgesic, antipyretic	synthetic	NF		-0.49	-0.45	1.61	2.03	0.67
01501175	FLURANDRENOLIDE	100122-02	B11	sample	1524-88-5	C24H33FO6	436.52541	antiinflammatory	semisynthetic	USP, INN, BAN, JAN		-1.51	-1.02	0.05	-1.00	-0.87
	0.04% JAK2inhib	100122-02	B12	0.04% jak2inhib								-4.04	-1.83	-1.12	-2.10	-2.27
	0.04% DMSO	100122-02	C01	0.04% dmso								-1.06	-1.31	-1.44	2.80	-0.25
01501184	ESTRADIOL ACETATE	100122-02	C02	sample	4245-41-4	C20H26O3	314.42842	estrogen	semisynthetic	USAN		-0.03	2.44	0.36	3.23	1.50
01501185	ECONAZOLE NITRATE	100122-02	C03	sample	68797-31-9, 27220-47-9	C18H16Cl3N3O4	444.70492	antifungal	SQ-13050; synthetic	USP, INN, BAN, JAN		-0.08	0.14	0.44	-2.00	-0.37
01501187	FLUNISOLIDE	100122-02	C04	sample	77326-96-6, 3385-03-3	C24H31FO6	434.50947	antiinflammatory	semisynthetic	USP, INN, BAN, JAN		-0.23	0.44	-0.02	-0.14	0.01
01501196	FLUMETHASONE	100122-02	C05	sample	2135-17-3	C22H28F2O5	410.46226	antiinflammatory	semisynthetic	USAN, INN, BAN		-0.83	-0.41	-0.74	-0.76	-0.68

												Screen Score (z-score)				
Compound ID	MolNAME	plate	position	Content	cas#	Formula	MolWt	Bioactivity	Source	Status	Reference	rep1	rep2	rep3	rep4	Average
01501200	XYLAZINE	100122-02	C06	sample	23076-35-9, 7361-61-7	C12H16N2S	220.33872	analgesic	synthetic	USP, INN, BAN		0.26	-0.35	-0.53	0.12	-0.12
01501201	TOLAZAMIDE	100122-02	C07	sample	1156-19-0	C14H21N3O3S	311.40577	antidiabetic	synthetic	USP, INN, BAN, JAN		0.35	0.48	-0.46	2.09	0.62
01501202	GALANTHAMINE HYDROBROMIDE	100122-02	C08	sample	357-70-0, 1953-04-4	C17H22BrNO3	368.27379	anticholinesterase, analgesic, antiAlzheimer	Galanthus, Narcissus and other Lillaceae	USAN		-3.43	-0.69	0.35	5.13	0.34
01505602	FENRETINIDE	100122-02	C09	sample	65646-68-6	C26H33NO2	391.55841	antineoplastic	synthetic; MCN-R-1967	USAN, INN		1.12	-0.26	-0.49	0.07	0.11
01501214	ENALAPRIL MALEATE	100122-02	C10	sample	76095-16-4, 75847-73-3	C24H32N2O9	492.53064	ACE inhibitor, antihypertensive	synthetic	USP, INN, BAN, JAN		-0.77	-0.52	0.58	-0.10	-0.20
01501215	KETOPROFEN	100122-02	C11	sample	22071-15-4	C16H14O3	254.28818	antiinflammatory	synthetic	USP, INN, BAN, JAN		0.64	-0.58	-0.33	0.40	0.03
	0.11% JAK2inhib	100122-02	C12	0.11% jak2inhib								-4.66	-5.87	-2.92	-4.83	-4.57
	0.11% DMSO	100122-02	D01	0.11% dmso								-1.34	-1.67	-1.85	-2.92	-1.95
01501217	LISINOPRIL	100122-02	D02	sample	83915-83-7, 76547-98-3	C21H31N3O5	405.49832	ACE inhibitor	synthetic	USP, INN, BAN, JAN		0.88	0.75	2.50	-1.77	0.59
01502004	BUMETANIDE	100122-02	D03	sample	28395-03-1	C17H20N2O5S	364.42335	diuretic	synthetic	USP, INN, BAN, JAN		-0.67	5.11	0.12	2.91	1.87
01502005	CARBENOXOLONE SODIUM	100122-02	D04	sample	7421-40-1, 5697-56-3	C34H48Na2O7	614.73706	antiinflammatory, antisecretory, antiulcer	semisynthetic	USAN, INN, BAN, JAN		-0.28	-0.40	-0.40	-1.71	-0.70
01502020	FOLIC ACID	100122-02	D05	sample	59-30-3	C19H19N7O6	441.40658	hematopoietic vitamin	liver, kidney, green plants and fungi	USP, INN, BAN, JAN		-0.12	1.61	0.17	-1.17	0.12
01502021	PHTHALYLSULFATHIAZOLE	100122-02	D06	sample	85-73-4	C17H13N3O5S2	403.43826	antibacterial	synthetic	USP-XX, INN, BAN		1.01	0.79	1.32	0.03	0.79
01502025	SUCCINYLSULFATHIAZOLE	100122-02	D07	sample	116-43-8	C13H13N3O5S2	355.39366	antibacterial	synthetic	USP-XVIII, INN, BAN		-0.08	0.20	0.33	3.25	0.93
01502026	TRANEXAMIC ACID	100122-02	D08	sample	1197-18-8	C8H15NO2	157.21425	hemostatic	synthetic	USAN, INN, BAN, JAN		0.77	2.48	0.29	0.02	0.89
01502028	CEPHALEXIN	100122-02	D09	sample	23325-78-2, 15686-71-2	C16H17N3O4S	347.39559	antibacterial	semisynthetic	USP, INN, BAN, JAN		-0.66	-0.40	-0.46	-0.14	-0.41
01502030	OXOLINIC ACID	100122-02	D10	sample	14698-29-4	C13H11NO5	261.23632	antibacterial	synthetic	USAN, INN, BAN		-0.07	-0.90	0.07	1.30	0.10
01502031	CEFOXITIN SODIUM	100122-02	D11	sample	66309-69-1, 61622-34-2	C16H16N3NaO7S2	449.43962	antibacterial	semisynthetic	USP, INN, BAN, JAN		-0.83	-0.72	-0.58	0.69	-0.36
	0.11% JAK2inhib	100122-02	D12	0.11% jak2inhib								-3.40	-3.91	-2.35	-2.73	-3.10
	0.11% JAK2inhib	100122-02	E01	0.11% jak2inhib								-1.72	-3.33	-0.12	-3.52	-2.17
01502032	SURAMIN	100122-02	E02	sample	129-46-4, 145-63-1 [suramin]	C51H34N6Na6O23S6	1429.18883	antiprotozoal, trypanocidal, antiviral	synthetic; Bayer-205, 309F	USP		0.01	0.67	0.81	-0.79	0.18
01502033	CEFUROXIME SODIUM	100122-02	E03	sample	56238-63-2	C16H15N4NaO8S	446.37375	antibacterial	semisynthetic	USAN, INN, BAN		-0.21	-0.11	1.33	-1.41	-0.10
01502036	VIGABATRIN	100122-02	E04	sample	60643-86-9	C6H11NO2	129.16007	anticonvulsant	synthetic; MDL-71754	USAN, INN, BAN		0.26	0.86	0.24	-0.02	0.33
01502037	LOMEFLOXACIN HYDROCHLORIDE	100122-02	E05	sample	98079-52-8, 98079-51-7	C17H20ClF2N3O3	387.81705	antibacterial	synthetic	USAN, JAN		-0.26	0.41	0.73	0.14	0.26
01502038	CEFAMANDOLE SODIUM	100122-02	E06	sample	34444-01-4	C18H17N6NaO5S2	484.49119	antibacterial	semisynthetic	USAN, INN, BAN		2.01	1.57	1.80	1.01	1.60
01502040	CEFMETAZOLE SODIUM	100122-02	E07	sample	56796-39-5	C15H16N7NaO5S3	493.52047	antibacterial	semisynthetic	USP, INN		0.09	-0.33	-0.53	-0.49	-0.32
01502042	CEFOPERAZONE SODIUM	100122-02	E08	sample	62893-20-3, 62893-19-0	C25H26N9NaO8S2	667.65927	antibacterial	semisynthetic	USP, INN, BAN, JAN		-0.19	0.30	-0.21	0.21	0.03
01502044	OFLOXACIN	100122-02	E09	sample	82419-36-1	C18H20FN3O4	361.3762	antibacterial	synthetic	USP, INN, BAN, JAN		1.28	-0.51	-0.53	0.61	0.21
01502046	BEZAFIBRATE	100122-02	E10	sample	41859-67-0	C19H20ClNO4	361.82855	antihyperlipidemic	synthetic	USAN, INN, BAN, JAN		0.15	-0.47	-0.58	4.41	0.88
01505371	CETIRIZINE HYDROCHLORIDE	100122-02	E11	sample	83881-52-1	C21H27Cl3N2O3	461.81994	H1 antihistamine	synthetic	USAN, INN, BAN		-3.93	-0.97	0.42	-0.03	-1.13
	0.11% DMSO	100122-02	E12	0.11% dmso								-1.12	-2.34	-1.30	-1.82	-1.64
	0.11% JAK2inhib	100122-02	F01	0.11% jak2inhib								-1.89	-3.92	-2.73	-4.64	-3.30
01505398	PHENYLETHYL ALCOHOL	100122-02	F02	sample	60-12-8	C8H10O	122.1683	antimicrobial	synthetic	USP, BAN		0.50	0.37	0.29	-0.96	0.05
01501118	MECLOCYCLINE SULFOSALICYLATE	100122-02	F03	sample	73816-42-9, 2013-58-3	C29H27ClN2O14S	695.06054	antibacterial	semisynthetic; GS-2989, NSC-78502	USAN, INN, BAN		2.23	0.03	-0.25	4.73	1.68
01505347	RIBOFLAVIN	100122-02	F04	sample	83-88-5	C17H20N4O6	376.37215	Vitamin B2; Vitamin cofactor; LD50(rat) 560 mg/kg ip	retina, whey and urine	USP, INN, BAN, JAN	Helv Chim Acta 35:457 (1952); Tet. Lett.,1969:3489; JACS	0.64	0.14	0.05	0.21	0.26
01500665	ACEBUTOLOL HYDROCHLORIDE	100122-02	F05	sample	34381-68-5, 37517-30-9	C18H29ClN2O4	372.89583	antihypertensive, antianginal, antiarrhythmic	synthetic	USAN, INN, BAN		-0.14	0.53	-0.15	3.30	0.88
01505306	ASPARTAME	100122-02	F06	sample	22839-47-0	C14H18N2O5	294.30996	sweetener	synthetic	USAN, NF, INN, BAN		0.81	-0.18	2.62	0.88	1.03
01505374	VARDENAFIL HYDROCHLORIDE	100122-02	F07	sample	224789-15-5	C23H33ClN6O4S	525.07426	erectile dysfunction, PD5 inhibitor	synthetic	USAN, INN, BAN		-0.56	0.85	0.55	1.26	0.53
01505396	FLUORESCEIN	100122-02	F08	sample	2321-07-5	C20H12O5	332.31564	corneal trama indicator	synthetic	USP, BAN, JAN		0.98	0.34	0.01	0.82	0.54
01505397	NIACINAMIDE	100122-02	F09	sample	98-92-0	C6H6N2O	122.12752	Vitamin B3; enzyme cofactor; anti-pellagra	widespread in plants, yeasts, fungi	USP, INN, JAN		0.20	-0.22	-0.72	0.51	-0.06
01505270	PROPRANOLOL HYDROCHLORIDE (+/-)	100122-02	F10	sample	318-98-9, 525-66-6	C16H22ClNO2	295.81224	antihypertensive, antianginal, antiarrhythmic	synthetic	USP, INN, BAN, JAN		-1.21	-1.94	-0.07	0.44	-0.69
01500401	METHSCOPOLAMINE BROMIDE	100122-02	F11	sample	155-41-9	C18H24BrNO4	398.30028	anticholinergic	semisynthetic	USP-XXII, BAN, JAN		-1.37	0.13	-0.02	0.35	-0.23
	0.04% DMSO	100122-02	F12	0.04% dmso								-1.24	-0.53	0.77	-0.63	-0.41
	0.04% JAK2inhib	100122-02	G01	0.04% jak2inhib								-1.56	-2.11	-2.34	-4.22	-2.56
02300219	EDROPHONIUM CHLORIDE	100122-02	G02	sample	116-38-1, 312-48-1	C10H16ClNO	201.69812	acetylcholinesterase inhibitor	synthetic	USP, INN, BAN, JAN		2.69	-0.04	0.39	-1.01	0.50
01900005	THIOPENTAL SODIUM	100122-02	G03	sample	71-73-8, 76-75-5 [thiopental]	C11H17N2NaO2S	264.32414	anesthetic	synthetic	USP, INN, BAN, JAN		1.12	0.56	0.95	-0.60	0.51
02300061	CLOMIPRAMINE HYDROCHLORIDE	100122-02	G04	sample	17321-77-6, 303-49-1	C19H24Cl2N2	351.32253	antidepressant	synthetic	USP, INN, BAN, JAN		0.68	-0.14	-0.12	-0.81	-0.10
01505983	PHENFORMIN HYDROCHLORIDE	100122-02	G05	sample	834-28-6	C10H16ClN5	241.72552	antidiabetic	synthetic	USP-XIX, INN, BAN	JACS 81:2220 (1959); Ann Pharmacother27:1183 (1993)	0.20	0.25	0.52	0.87	0.46
01505691	PENFLURIDOL	100122-02	G06	sample	26864-56-2	C28H27ClF5NO	523.97849	antipsychotic	synthetic; McN-JR-16341, R-16341	USAN, INN, BAN		0.67	4.88	0.43	0.04	1.51
02300009	TILORONE	100122-02	G07	sample	27591-69-1, 27591-97-5	C25H34N2O3	410.56133	antiviral	synthetic	USAN, INN		-0.92	0.36	2.22	0.11	0.44
01505672	VINCRISTINE SULFATE	100122-02	G08	sample	2068-78-2	C46H58N4O14S	923.05756	antineoplastic	Vinca rosea; 37231, NSC-67574	USP, INN, BAN, JAN	Int Pharmacodyn Ther 157:299 (1965)	0.37	0.82	-0.24	1.59	0.63
01505693	OMEPRAZOLE	100122-02	G09	sample	73590-58-6	C17H19N3O3S	345.42328	gastric acid depressant	synthetic; H-168/68	USP, INN, BAN, JAN		-0.01	0.40	0.41	1.50	0.58
01505281	ZOLMITRIPTAN	100122-02	G10	sample	139264-17-8	C16H21N3O2	287.36467	antimigraine, 5HT[1B/1D] agonist	synthetic	USAN, INN, BAN		1.57	-0.81	-0.41	-0.20	0.04
01506066	DEBRISOQUIN SULFATE	100122-02	G11	sample	581-88-4	C10H15N3O4S	273.31275	anti-hypertensive	synthetic; RO-5-3307/1	USAN, INN, BAN		0.32	0.08	-0.02	0.97	0.34
	0.04% DMSO	100122-02	G12	0.04% dmso								0.12	-0.42	0.69	0.34	0.18
	0.04% JAK2inhib	100122-02	H01	0.04% jak2inhib								-0.51	-1.94	-0.86	-3.65	-1.74
01506086	SULFADOXINE	100122-02	H02	sample	2447-57-6	C12H14N4O4S	310.33378	antibacterial	synthetic; RO-4-4393	USP, INN, BAN, JAN		0.51	0.28	-0.69	3.03	0.78
01506069	FINASTERIDE	100122-02	H03	sample	98319-26-7	C23H36N2O2	372.55557	anti-androgen, alpha-reductase inhibitor	synthetic; MK=909	USP, INN, BAN		0.83	1.64	0.08	-0.88	0.42
01506082	PENTETIC ACID	100122-02	H04	sample	67-43-6	C14H23N3O10	393.35351	chelating agent, diagnostic aid	synthetic; DTPA	USP, INN, BAN		0.52	-0.65	0.81	-0.70	-0.01
01506084	PROSCILLARIDIN	100122-02	H05	sample	466-06-8	C29H40O9	532.63675	cardiotonic	Scilla spp; A-32686, 2936	USAN, INN, BAN, JAN		0.89	1.08	-0.36	-0.26	0.34
01505438	HYDROCORTISONE VALERATE	100122-02	H06	sample	57524-89-7	C26H38O6	446.58916	antiinflammatory, glucocorticoid	semisynthetic	USP		0.17	0.03	2.97	-1.28	0.47
01506035	REPAGLINIDE	100122-02	H07	sample	135062-02-1	C27H36N2O4	452.59897	antidiabetic	synthetic; AG-EE-623-ZW	USP, INN, BAN		0.49	0.74	0.08	-1.33	0.00
01505271	CROTAMITON	100122-02	H08	sample	483-63-6	C13H17NO	203.28654	antipruritic, scabicide	synthetic	USP, INN, BAN, JAN		-0.57	-0.40	-0.18	-0.68	-0.46
01505364	CEFPROZIL	100122-02	H09	sample	121123-17-9	C18H19N3O5S	389.43323	antibacterial	semisynthetic; BMY-28100-03-800	USP, INN, BAN		0.31	-0.11	-1.23	0.88	-0.04
01505384	METHYLDOPATE HYDROCHLORIDE	100122-02	H10	sample	2508-79-4	C12H18ClNO4	275.73456	antihypertensive	synthetic	USP, BAN		-0.13	-0.26	-0.50	-0.71	-0.40
01501145	SULFAQUINOXALINE SODIUM	100122-02	H11	sample	59-40-5	C14H11N4NaO2S	322.32317	antibacterial	synthetic	USP, INN, BAN		-1.55	-1.34	-0.03	-0.70	-0.90
	empty	100122-02	H12	sample								NA	NA	NA	NA	NA
	empty	100122-03	A01	sample								NA	NA	NA	NA	NA
01500113	POTASSIUM p-AMINOBENZOATE	100122-03	A02	sample	150-13-0 (acid)	C7H6KNO2	175.23337	ultraviolet screen	synthetic	USP		-0.41	0.02	-0.89	-0.90	-0.55
01500145	BETAMETHASONE VALERATE	100122-03	A03	sample	2152-44-5	C27H37FO6	476.59074	glucocorticoid	semisynthetic	USP, INN, BAN, JAN		-0.56	0.35	-1.07	0.90	-0.09
01500280	ERYTHROMYCIN	100122-03	A04	sample	114-07-8	C37H67NO13	733.94544	antibacterial	Streptomyces erythreus	USP, INN, BAN, JAN		-2.50	-0.98	-1.17	-0.68	-1.33
01500510	PROMETHAZINE HYDROCHLORIDE	100122-03	A05	sample	58-33-3, 60-87-7	C17H21ClN2S	320.88732	antihistaminic	synthetic	USP, INN, BAN, JAN		0.84	0.55	2.20	5.08	2.17
01500534	SCOPOLAMINE HYDROBROMIDE	100122-03	A06	sample	6533-68-2, 114-49-8	C17H22BrNO4	384.27319	anticholinergic, treatment of motion sickness	Scopolia, Datura, Atropa spp	USP, BAN, JAN		-0.93	-1.28	0.10	0.01	-0.52
01500568	THEOPHYLLINE	100122-03	A07	sample	5967-84-0, 58-55-9	C7H8N4O2	180.16741	bronchodilator	Camelia, thea, Paullinia cupana	USP, BAN, JAN		0.29	0.57	-0.42	-1.18	-0.19
01500583	TOLNAFTATE	100122-03	A08	sample	2398-96-1	C19H17NOS	307.41744	antifungal	synthetic; SCH-10144	USP, INN, BAN, JAN		0.60	-0.48	0.57	-1.24	-0.14
01500594	TRIMETHOBENZAMIDE HYDROCHLORIDE	100122-03	A09	sample	554-92-7, 138-56-7	C21H29ClN2O5	424.92868	antiemetic	synthetic	USP, INN		-1.56	-1.18	0.52	-0.83	-0.77
01500611	VINBLASTINE SULFATE	100122-03	A10	sample	143-67-9, 865-21-4	C46H60N4O13S	909.0741	antineoplastic, spindle poison	Vinca rosea	USP, INN, BAN, JAN		-9.67	-8.05	-4.10	-8.26	-7.52
01504283	ARMODAFINIL	100122-03	A11	sample	112111-43-0	C15H15NO2S	273.3563	analeptic	synthetic; R-enatiomer of modafinil (01505361)	USAN		-6.12	-0.56	0.00	2.02	-1.16
	0.04% JAK2inhib	100122-03	A12	0.04% jak2inhib								-2.32	-3.68	-0.80	-4.07	-2.72
	0.04% DMSO	100122-03	B01	0.04% dmso								0.27	3.48	-1.05	-0.79	0.48
01502195	PIRACETAM	100122-03	B02	sample	7491-74-9	C6H10N2O2	142.1588	antinauseant	synthetic	USAN, INN, BAN		-0.46	0.07	-1.63	0.51	-0.37
01503092	GLUCONOLACTONE	100122-03	B03	sample	90-80-2	C6H10O6	178.143	chelating agent	synthetic	USP		-0.63	0.38	-0.31	-0.40	-0.24
01503101	AZLOCILLIN SODIUM	100122-03	B04	sample	37091-66-0	C20H22N5NaO6S	483.48204	antibacterial	semisynthetic	USAN, INN, BAN		-0.03	-0.10	-0.41	2.18	0.41
01503428	CHOLINE CHLORIDE	100122-03	B05	sample	67-48-1, 62-49-7 [choline]	C5H14ClNO	139.62643	choleretic, lipotropic, hepatoprotectant	widespread in the plant and animal kingdoms	USP, INN		0.88	-0.95	0.33	0.73	0.25
01503722	ATORVASTATIN CALCIUM	100122-03	B06	sample	134523-03-8, 134523-00-5	C33H33CaFNO5	582.71306	antihyperlipidemic, HMGCoA reductase inhibitor	synthetic	USAN, INN, BAN		3.11	0.24	-0.37	0.82	0.95
01503932	OXYPHENCYCLIMINE HYDROCHLORIDE	100122-03	B07	sample	125-52-0, 125-53-1	C20H29ClN2O3	380.91873	anticholinergic	synthetic	USP-XXII, INN, BAN, JAN		1.18	-0.29	0.64	0.43	0.49
01503935	PROPAFENONE HYDROCHLORIDE	100122-03	B08	sample	34183-22-7, 54063-53-5	C21H28ClNO3	377.91521	antiarrhythmic	synthetic	USP, INN, BAN, JAN		-0.55	-1.11	-0.11	3.12	0.34
01503975	FLUCONAZOLE	100122-03	B09	sample	86386-73-4	C13H12F2N6O	306.27699	antifungal	synthetic	USAN, INN, BAN, JAN		2.56	-0.12	-0.35	-0.19	0.48
01503977	LOVASTATIN	100122-03	B10	sample	75330-75-5	C24H36O5	404.55152	antihyperlipidemic, HMGCoA reductase inhibitor	Aspergillus spp; mevinolin	USP, INN, BAN	PNAS 77:3957 (1980); Int J Oncol 12:717 (1998)	0.83	-0.36	2.18	0.89	0.88
01505407	ATROPINE OXIDE	100122-03	B11	sample	4438-22-6	C17H25NO4	307.3931	anticholinergic	semisynthetic	USAN, INN		-0.36	-1.21	0.32	-0.67	-0.48
	0.04% JAK2inhib	100122-03	B12	0.04% jak2inhib								-1.10	-0.61	-0.97	-1.95	-1.16
	0.04% DMSO	100122-03	C01	0.04% dmso								0.11	0.23	-1.74	0.07	-0.33
01504078	SENNOSIDE A	100122-03	C02	sample	81-27-6	C42H38O20	862.75916	cathartic	Cassia & Rheum spp	USP, JAN	Helv Chim Acta 33: 313 (1950)	0.56	-0.25	-0.94	0.01	-0.16
01504094	TENIPOSIDE	100122-03	C03	sample	29767-20-2	C32H32O13S	656.66804	antineoplastic	semisynthetic	USAN, INN, BAN		-9.31	-11.19	-13.15	-12.42	-11.51
01504105	TANNIC ACID	100122-03	C04	sample	1401-55-4	C76H52O46	1701.23424	nonspecific enzyme/receptor blocker	principal constituent of tree galls, esp Quercus spp;	USP, JAN	mixed galloyl esters of penta-o-galloylglucose (m-digalloyl	2.78	4.32	2.71	3.41	3.30
01502006	CARPROFEN	100122-03	C05	sample	53716-49-7	C15H12ClNO2	273.72139	antiinflammatory, analgesic	synthetic	USAN, INN, BAN		0.28	0.24	0.07	0.42	0.25
01503978	HYDROXYCHLOROQUINE SULFATE	100122-03	C06	sample	747-36-4, 118-42-3	C18H28ClN3O5S	433.95796	antimalarial, lupus suppressant	synthetic	USP-XXII, INN		1.12	0.24	-0.45	-0.46	0.11
01504144	DIRITHROMYCIN	100122-03	C07	sample	62013-04-1	C42H78N2O14	835.09496	antibacterial	semisynthetic	USP, INN, BAN	Drugs 61: 443 (2001)	-0.29	-1.05	0.13	-0.82	-0.51
01504148	MEPIVACAINE HYDROCHLORIDE	100122-03	C08	sample	1722-62-9, 96-88-8	C15H23ClN2O	282.81636	anesthetic (local)	synthetic	USP, INN, BAN, JAN	Mol Pharmacol 54: 162 (1998)	-0.63	-1.05	0.23	-0.33	-0.44
01504152	NILUTAMIDE	100122-03	C09	sample	63612-50-0	C12H10F3N3O4	317.2264	antiandrogen	synthetic	USAN, INN, BAN	Pharmacotherapy 31: 65 (1997)	0.91	-0.75	-0.51	-1.54	-0.47
01504184	AMINOLEVULINIC ACID HYDROCHLORIDE	100122-03	C10	sample	5451-09-2	C5H10ClNO3	167.59335	antineoplastic	synthetic	USAN		0.68	-0.27	0.96	-0.04	0.33
01505456	PARAMETHADIONE	100122-03	C11	sample	115-67-3	C7H11NO3	157.17062	anticonvulsant	synthetic	USP-XXII, INN, BAN		0.93	-0.71	0.29	3.07	0.89
	0.11% JAK2inhib	100122-03	C12	0.11% jak2inhib								-4.30	-3.73	-2.42	-4.07	-3.63
	0.11% DMSO	100122-03	D01	0.11% dmso								1.03	1.26	-1.05	5.48	1.68
01504229	METAXALONE	100122-03	D02	sample	1665-48-1	C12H15NO3	221.25825	muscle relaxant (skeletal)	synthetic	USAN, INN, BAN		-0.34	0.23	-0.76	-0.59	-0.37
01504211	CHLOROGUANIDE HYDROCHLORIDE	100122-03	D03	sample	637-32-1	C11H17Cl2N5	290.19764	antimalarial	synthetic	USP-XIV, INN, BAN		1.16	0.63	3.00	1.23	1.50
01504231	CLARITHROMYCIN	100122-03	D04	sample	81103-11-9	C38H69NO13	747.97253	antibacterial	Streptomyces erythreus	USP, INN, BAN, JAN		1.32	0.38	-0.58	-0.31	0.20
01504237	HYDROQUINONE	100122-03	D05	sample	123-31-9	C6H6O2	110.11352	antioxidant	synthetic	USP		0.17	0.41	0.06	4.67	1.33
01504258	NATEGLINIDE	100122-03	D06	sample	105816-04-4	C19H27NO3	317.43194	antidiabetic	synthetic	USAN, INN, BAN		1.57	1.08	0.55	-0.53	0.67
01504261	CANDESARTAN CILEXTIL	100122-03	D07	sample	139481-59-7	C33H34N6O6	610.67553	angiotensin 1 receptor antagonist	synthetic	USAN, INN		-3.53	-1.16	-0.38	-0.88	-1.49
01504263	ROSIGLITAZONE	100122-03	D08	sample	122320-73-4	C18H19N3O3S	357.43443	antidiabetic	synthetic	USAN, INN, BAN		1.32	1.15	1.48	0.04	1.00
01504268	LOSARTAN	100122-03	D09	sample	124750-99-8, 114798-26-4	C22H23ClN6O	422.92121	antihypertensive, AT1 angiotensin II antagonist	synthetic	USAN, INN, BAN		0.14	0.61	1.59	0.77	0.78
01505020	HOMOSALATE	100122-03	D10	sample	118-56-9	C16H22O3	262.35194	UV screen, analgesic	synthetic	USP, INN		0.80	0.95	1.24	-0.01	0.75
00300618	SALICYLANILIDE	100122-03	D11	sample	87-17-2	C13H11NO2	213.23812	antipyretic, fungicide	synthetic	NF-XIII		-0.16	0.15	1.19	3.82	1.25
	0.11% JAK2inhib	100122-03	D12	0.11% jak2inhib								-4.00	-4.76	-2.01	-2.87	-3.41
	0.11% JAK2inhib	100122-03	E01	0.11% jak2inhib								-3.26	-2.86	0.84	-0.98	-1.57
01505022	PROPOFOL	100122-03	E02	sample	2078-54-8	C12H18O	178.27666	anesthetic	synthetic; ICI-35868	USAN, INN, BAN		0.04	2.17	-1.63	-0.11	0.12
00200046	GRISEOFULVIN	100122-03	E03	sample	126-07-8	C17H17ClO6	352.77444	antifungal, inhibits mitosis in metaphase	Penicillium griseofulvum	USP, INN, BAN, JAN	J Chem Soc 1952: 3949	-0.62	-0.94	-1.20	-0.49	-0.81
01505200	BENAZEPRIL HYDROCHLORIDE	100122-03	E04	sample	86541-74-4	C24H29ClN2O5	460.96213	ACE inhibitor, antihypertensive	synthetic	USAN, INN, BAN, JAN		-1.26	-0.12	-1.24	0.68	-0.48
01505209	VALSARTAN	100122-03	E05	sample	137862-53-4	C24H29N5O3	435.53043	Angiotensin II inhibitor, antihypertensive	synthetic; CGP-48933	USAN, INN, BAN		1.73	0.31	0.55	0.79	0.85
00200331	SALSALATE	100122-03	E06	sample	552-94-3	C14H10O5	258.2328	analgesic	synthetic	USP, INN, BAN		0.03	-1.25	0.24	-0.11	-0.27
00300024	HYDROCORTISONE	100122-03	E07	sample	50-23-7	C21H30O5	362.47025	glucocorticoid, antiinflammatory	adrenal glands	USP, INN, BAN, JAN		0.97	-0.30	0.09	-0.28	0.12
01505321	RIFAXIMIN	100122-03	E08	sample	80621-81-4	C43H51N3O11	785.89942	antibacterial, RNA synthesis inhibitor	semisynthetic	USAN, INN	Drugs 49:467 (1995)	0.41	0.06	0.00	-0.10	0.09
01505248	CANRENONE	100122-03	E09	sample	976-71-6	C22H28O3	340.46666	aldosterone antagonist; antifibrogenic	synthetic	USAN, INN	Eur J Clin Pharmacol 9: 253 (1976); Gastroenterology 124:	-0.72	-1.00	-0.14	-0.81	-0.67
01505361	MODAFINIL	100122-03	E10	sample	68693-11-8	C15H15NO2S	273.3563	analeptic	synthetic; CRL-40476, CEP-1538	USAN, INN, BAN		0.50	0.21	0.10	0.11	0.23
01505114	CLIOQUINOL	100122-03	E11	sample	130-26-7	C9H5ClINO	305.5037	antiseptic, antiamebic	synthetic	USP, INN, BAN		-0.33	0.45	0.04	-0.83	-0.17
	0.11% DMSO	100122-03	E12	0.11% dmso								-1.44	-2.17	-0.94	-2.04	-1.65
	0.11% JAK2inhib	100122-03	F01	0.11% jak2inhib								-1.97	-1.25	-2.49	-3.40	-2.28

														Screen Score (z-score)		
**Compound ID**	**MolNAME**	**plate**	**position**	**Content**	**cas#**	**Formula**	**MolWt**	**Bioactivity**	**Source**	**Status**	**Reference**	**rep1**	**rep2**	**rep3**	**rep4**	**Average**
01505366	RANOLAZINE	100122-03	F02	sample	95635-55-5	C24H33N3O4	427.54831	antianginal, antiischemic	synthetic; RS-43285-003; CVT-303	USAN, INN		0.48	2.49	2.73	0.54	1.56
00211468	DANTHRON	100122-03	F03	sample	117-10-2	C14H8O4	240.21746	cathartic	Rheum palmatum, Xyris semifuscata	USP-XXI, INN, BAN	JACS 53: 4112 (1931)	-0.23	-0.02	-0.94	0.91	-0.07
01505376	ACEDAPSONE	100122-03	F04	sample	77-46-3	C16H16N2O4S	332.38092	antimalarial, leprostatic	synthetic; CI-556, CN-1883, DADDS, PAM-MAR-1165	USAN, INN, BAN		-0.33	0.90	-0.29	-0.32	-0.01
01505385	ATOMOXETINE HYDROCHLORIDE	100122-03	F05	sample	82248-59-7	C16H20ClNO	277.7969	norepinephrine reuptake inhibitor	synthetic; LY-139603	USAN, INN, BAN		0.29	0.48	-0.09	3.67	1.09
00300029	DESOXYCORTICOSTERONE ACETATE	100122-03	F06	sample	56-47-3	C23H32O4	372.50909	mineralocorticoid	adrenocortex	USP, INN, BAN		0.96	-0.68	2.04	-0.13	0.55
01505389	TRAMADOL HYDROCHLORIDE	100122-03	F07	sample	36282-47-0	C16H26ClNO2	299.84412	analgesic	synthetic; U-26225A, CG-315E	USAN, INN, BAN, JAN		0.41	-0.32	0.46	0.30	0.21
01505392	TERBINAFINE HYDROCHLORIDE	100122-03	F08	sample	78628-80-5, 91161-71-6(base)	C21H26ClN	327.90107	antifungal	synthetic, SF-86-327	USAN, INN, BAN		0.88	-1.14	1.01	0.09	0.21
01505801	TOPIRAMATE	100122-03	F09	sample	97240-79-4	C12H21NO8S	339.36707	anticonvulsant, antimigraine, GABA-A agonist, AMP/kinate	synthetic; RWJ-17021	USAN, INN, BAN		-1.08	-0.21	1.16	0.86	0.18
01505802	GEMIFLOXACIN MESYLATE	100122-03	F10	sample	204519-65-3	C19H24FN5O7S	485.49483	antibacterial	synthetic	USAN		-6.80	0.30	1.20	0.09	-1.30
01505803	PRAVASTATIN SODIUM	100122-03	F11	sample	81131-70-6	C23H35NaO7	446.521	antihyperlipidemic, HMGCoA reductase inhibitor	CS-514; SQ-31000	USAN, INN, BAN, JAN		-1.60	-0.45	0.38	-1.35	-0.75
	0.04% DMSO	100122-03	F12	0.04% dmso								0.24	-0.77	2.53	0.20	0.55
	0.04% JAK2inhib	100122-03	G01	0.04% jak2inhib								-0.96	2.50	0.95	-1.25	0.31
01505811	LEVALBUTEROL HYDROCHLORIDE	100122-03	G02	sample	50293-90-8	C13H22ClNO3	275.77819	bronchodilator, tocolytic	synthetic	USAN		-0.32	0.29	-0.94	0.84	-0.03
01505814	METFORMIN HYDROCHLORIDE	100122-03	G03	sample	1115-70-4, 657-24-	C4H12ClN5	165.62674	antidiabetic	synthetic; LA-6023	USAN, JAN		1.03	1.32	-0.31	0.78	0.71
01505816	PREGABALIN	100122-03	G04	sample	148553-50-8	C8H17NO2	159.23019	anticonvulsant	synthetic; CI-1008	USAN, INN		0.12	0.64	-0.85	0.60	0.13
01505820	TOPOTECAN HYDROCHLORIDE	100122-03	G05	sample	119413-54-6	C23H24ClN3O5	457.91783	antineoplastic; topoisomerase I inhibitor	semisynthetic	USAN, INN, BAN		0.17	1.04	-0.39	0.15	0.24
02300176	PHENOXYBENZAMINE HYDROCHLORIDE	100122-03	G06	sample	63-92-3, 59-96-1	C18H23Cl2NO	340.29611	alpha adrenergic blocker	synthetic	USP		-0.41	0.55	-0.14	-0.13	-0.03
02300270	PINACIDIL	100122-03	G07	sample	85371-64-8, 60560-33-0	C13H19N5	245.32988	K channel agonist, antihypertensive	synthetic	USAN, INN		-0.31	-0.46	-0.18	-0.10	-0.26
02300307	VERAPAMIL HYDROCHLORIDE	100122-03	G08	sample	152-11-4, 52-53-9 [verapamil]	C27H39ClN2O4	491.07588	adrenegic blocker, Ca channel blocker, coronary vasodilator,	synthetic	USP, INN, BAN, JAN		0.12	-0.30	-0.14	-0.10	-0.10
01505818	PANTOPRAZOLE	100122-03	G09	sample	102625-70-7	C16H15F2N3O4S	383.37645	proton pump inhibitor, gastric acid release inhibitor, antiulcer	synthetic; SK&F-96022, BY-1023	USAN, INN, BAN		-0.07	-0.03	0.49	1.93	0.58
02300241	LOPERAMIDE HYDROCHLORIDE	100122-03	G10	sample	34552-83-5, 53179-11-6	C29H34Cl2N2O2	513.51253	Ca channel blocker	synthetic	USP, INN, BAN, JAN		-1.89	-0.56	-0.38	1.54	-0.32
02300332	PODOFILOX	100122-03	G11	sample	518-28-5	C22H22O8	414.41584	antineoplastic, inhibits microtubule assembly, and human DNA	Podophylum peltatum; podophylotoxin	USAN, BAN	JACS 73: 2909 (1951); 75: 1308 (1953); J Med Chem 32:	-10.56	-6.56	-5.80	-3.21	-6.53
	0.04% DMSO	100122-03	G12	0.04% dmso								-0.10	5.78	0.17	5.24	2.77
	0.04% JAK2inhib	100122-03	H01	0.04% jak2inhib								-1.89	-1.52	-3.45	-2.43	-2.32
02300205	LEVODOPA	100122-03	H02	sample	59-92-7	C9H11NO4	197.19232	antiparkinsonian	Vicia faba seedlings, Sarothamnus spp, & other palnts	USP, INN, BAN, JAN		-0.65	-0.17	-1.13	0.30	-0.41
00300607	RUTOSIDE (rutin)	100122-03	H03	sample	153-18-4	C27H30O16	610.53055	vascular protectant	Ruta graveolens, widespread in plants	NF-XI, INN, BAN, JAN		-0.43	0.43	0.56	-0.41	0.04
01500615	ZOMEPIRAC SODIUM	100122-03	H04	sample	64092-49-5, 64092-48-4	C15H14ClNO3	291.73673	analgesic, antiinflammatory	synthetic	USAN, INN, BAN		0.05	1.02	-0.36	0.56	0.32
00300548	SPARTEINE SULFATE	100122-03	H05	sample	6160-12-9, 299-39-8	C15H28N2O4S	332.46541	oxytocic	Lupinus spp and other Leguminosae	USAN, BAN, JAN	J Chem Soc 1949: 663	-0.16	0.02	-0.89	-1.08	-0.53
00300034	TESTOSTERONE PROPIONATE	100122-03	H06	sample	57-85-2	C22H32O3	344.49854	androgen, antineoplastic	semisynthetic	USP, JAN		0.11	0.76	-0.10	-0.76	0.00
01506009	METHIMAZOLE	100122-03	H07	sample	60-56-0	C4H6N2S	114.16982	thyroid inhibitor	synthetic	USP, INN, BAN, JAN		-0.22	1.72	-0.45	5.54	1.65
01506067	ENILCONAZOLE	100122-03	H08	sample	35554-44-0	C14H14Cl2N2O	297.18648	antifungal	synthetic; R-23979	USAN, INN, BAN		-0.78	-0.37	0.22	-0.11	-0.26
01504526	FIROCOXIB	100122-03	H09	sample	189954-96-9	C17H20O5S	336.40995	analgesic, antiinflammatory, antipyretic, COX-II inhibitor	synthetic; ML-1785713	USAN, INN		0.51	0.59	0.24	-0.72	0.15
00330071	LINDANE	100122-03	H10	sample	58-89-9	C6H6Cl6	290.83272	insecticide	synthetic; gamma-BHC	USP, INN, BAN	Use: insecticide	-0.38	0.74	3.01	0.78	1.04
01504218	ACRISORCIN	100122-03	H11	sample	7527-91-5	C25H28N2O2	388.51411	antifungal	synthetic	USP-XXII, INN		-0.05	-0.04	0.73	-0.59	0.01
	empty	100122-03	H12	sample								NA	NA	NA	NA	NA
	empty	100122-04	A01	sample								NA	NA	NA	NA	NA
00305025	PHENYL AMINOSALICYLATE	100122-04	A02	sample	133-11-9	C13H11NO3	229.23752	antibacterial (tuberculostatic)	synthetic; NSC-40144	USAN, INN, BAN		-1.33	-1.12	-1.72	-0.52	-1.17
00307023	TESTOSTERONE	100122-04	A03	sample	58-22-0	C19H28O2	288.43381	androgen, antineoplastic	mamallian male hormone	USP, INN, BAN		-1.29	-0.98	1.04	1.99	0.19
00310035	SANGUINARINE SULFATE	100122-04	A04	sample	5578-73-4	C20H15NO8S	429.40845	antineoplastic, antiplaque agent	Sanguinaria canadensis	USAN, INN	J Heterocyclic Chem 9: 1453 (1972)	-1.60	-1.52	-2.51	-1.83	-1.86
00310039	alpha-TOCHOPHEROL	100122-04	A05	sample	59-02-9	C29H50O2	430.72065	vitamin E	soya, wheat germ and other plant oils	USP		-0.21	-0.91	-0.81	-1.66	-0.90
00310040	alpha-TOCHOPHERYL ACETATE	100122-04	A06	sample	58-95-7	C31H52O3	472.75829	vitamin E	derivative	USP		-0.23	0.44	-0.68	-0.90	-0.34
00330001	DACTINOMYCIN	100122-04	A07	sample	50-76-0	C62H86N12O16	1255.44752	antineoplastic, intercalating agent	Actinomyces spp	USP, INN, BAN, JAN		-3.11	-0.51	-2.64	1.26	-1.25
00330002	MITOMYCIN C	100122-04	A08	sample	50-07-7	C15H18N4O5	334.33451	antineoplastic	Streptomyces verticillatus	USP, INN, BAN, JAN		-1.03	-1.98	-0.90	-2.85	-1.69
00330018	DICHLORVOS	100122-04	A09	sample	62-73-7	C4H7Cl2O4P	220.97779	insecticide, cholinesterase inhibitor	synthetic	USAN, INN, BAN		-0.77	-1.84	2.03	-0.48	-0.26
00330062	TEMEFOS	100122-04	A10	sample	3383-96-8	C16H20O6P2S3	466.4738	insecticide	synthetic	USAN, INN		-1.23	-1.77	0.59	-2.04	-1.11
00330082	MITOTANE	100122-04	A11	sample	53-19-0	C14H10Cl4	320.0478	insecticide, antineoplastic	synthetic	USP, BAN, JAN		-0.32	-2.23	0.04	-1.84	-1.09
	0.04% JAK2inhib	100122-04	A12	0.04% jak2inhib								-2.29	-2.04	-0.72	-2.99	-2.01
	0.04% DMSO	100122-04	B01	0.04% dmso								-0.98	-0.24	-0.88	4.77	0.67
01300027	IVERMECTIN	100122-04	B02	sample	70288-86-7	C48H74O14	875.11658	antiparasitic	semisynthetic	USP, INN, BAN	Science 221:823 (1983); J Vet Pharmacol Ther 7:1 (1984)	-9.09	-4.82	-3.73	-2.40	-5.01
01300037	SODIUM NITROPRUSSIDE	100122-04	B03	sample	13755-38-9	C5FeN6Na2O	261.92195	antihypertensive	synthetic	USP	J Clin Pharmacol 14:494 (1974)	-3.52	-3.51	-3.55	-2.58	-3.29
01300038	SODIUM OXYBATE	100122-04	B04	sample	502-85-2	C4H7NaO3	126.08839	anesthetic	synthetic; WY-3478, NSC-84223	USAN		0.16	2.78	-1.47	-1.12	0.09
01400151	ETHYL PARABEN	100122-04	B05	sample	120-47-8	C9H10O3	166.17825	antifungal	synthetic	USAN, NF, JAN		-1.02	-0.48	4.52	0.02	0.76
01400208	COUMARIN	100122-04	B06	sample	91-64-5	C9H6O2	146.14697	antineoplastic, antiinflammatory, antihyperglycaemic	Coumarouna odorata, tonka beans, lavender oil	NF-X, INN	Prog Chem Org Nat Prod 9: 225 (1952)	0.09	-0.41	-0.65	-0.93	-0.47
01500101	ACETAMINOPHEN	100122-04	B07	sample	103-90-2	C8H9NO2	151.16643	analgesic, antipyretic	synthetic	USP, INN, BAN		-0.13	-0.01	0.71	0.18	0.19
01500102	ACETAZOLAMIDE	100122-04	B08	sample	59-66-5	C4H6N4O3S2	222.24542	carbonic anhydrase inhibitor, diuretic, antiglaucoma	synthetic	USP, INN, BAN, JAN		-0.58	-0.51	0.59	-0.63	-0.28
01500103	ACETOHYDROXAMIC ACID	100122-04	B09	sample	546-88-3	C2H5NO2	75.06765	urease inhibitor, antiurolithic, antbacterial	synthetic	USP, INN		0.20	0.35	0.55	0.01	0.28
01500104	ACETYLCHOLINE	100122-04	B10	sample	60-31-1, 51-84-3	C7H16ClNO2	181.66407	antiarrhythmic, miotic, vasodilator (peripheral)	synthetic	USP		0.36	0.74	0.40	-0.07	0.36
01500105	ACETYLCYSTEINE	100122-04	B11	sample	616-91-1	C5H9NO3S	163.19638	mucolytic	synthetic	USP, INN, BAN, JAN		0.82	-1.12	1.52	-0.60	0.16
	0.04% JAK2inhib	100122-04	B12	0.04% jak2inhib								-1.44	-1.55	4.40	-0.08	0.33
	0.04% DMSO	100122-04	C01	0.04% dmso								-0.33	-0.29	-1.76	1.45	-0.23
01500107	ADENOSINE	100122-04	C02	sample	58-61-7	C10H13N5O4	267.24621	antiarrhythmic, cardiac depressant	widespread in living tissue	USP, BAN		-1.02	-0.44	-0.78	-1.00	-0.81
01500108	ALLOPURINOL	100122-04	C03	sample	315-30-0	C5H4N4O	136.11383	antihyperuricemia, antigout, antiurolithic	synthetic	USP, INN, BAN, JAN		-0.77	-0.37	-0.97	-0.20	-0.58
01500109	ALVERINE CITRATE	100122-04	C04	sample	5560-59-8, 150-59-4 [alverine]	C26H35NO7	473.57135	anticholinergic	synthetic	USAN, INN, BAN		-0.94	-0.02	-0.96	0.16	-0.44
01500110	AMANTADINE HYDROCHLORIDE	100122-04	C05	sample	665-66-7, 768-94-5	C10H18ClN	187.71466	antiviral, antiparkinsonian; treatment of drug-induced	synthetic	USP, INN, BAN		-0.18	-0.73	-0.97	1.81	-0.02
01500111	AMIKACIN SULFATE	100122-04	C06	sample	39831-55-5, 37517-28-5	C22H47N5O21S2	781.76879	antibacterial	semisynthetic	USP, JAN		0.59	-0.22	0.12	2.51	0.75
01500112	AMILORIDE HYDROCHLORIDE	100122-04	C07	sample	17440-83-4, 2016-88-8	C6H9Cl2N7O	266.09093	Na+ channel inhibitor, diuretic	synthetic	USP, INN, BAN	Biochim Biophys Acta 944: 383 (1988)	0.69	0.00	0.51	-0.36	0.21
01500114	AMINOCAPROIC ACID	100122-04	C08	sample	60-32-2	C6H13NO2	131.17601	hemostatic	synthetic	USP, INN, BAN		-0.54	-0.15	-0.04	-0.34	-0.27
01500115	AMINOGLUTETHIMIDE	100122-04	C09	sample	125-84-8	C13H16N2O2	232.28467	aromatase inhibitor, antineoplastic, testosterone suppressant	synthetic	USP, INN, BAN		1.09	-0.03	0.54	-0.51	0.27
01500116	AMINOSALICYLATE SODIUM	100122-04	C10	sample	6018-19-5	C7H6NNaO3	175.12057	antibacterial, tuberculostatic	synthetic	USP		0.00	-0.57	1.37	1.11	0.48
01500117	AMITRIPTYLINE HYDROCHLORIDE	100122-04	C11	sample	549-18-8	C20H24ClN	313.87398	antidepressant	synthetic	USP, INN, BAN, JAN		0.22	-0.76	0.42	-0.02	-0.04
	0.11% JAK2inhib	100122-04	C12	0.11% jak2inhib								-3.07	-4.27	-1.80	-1.92	-2.76
	0.11% DMSO	100122-04	D01	0.11% dmso								-1.87	-2.11	-3.27	-2.04	-2.32
01500119	AMODIAQUINE DIHYDROCHLORIDE	100122-04	D02	sample	6398-98-7, 69-44-3	C20H24Cl3N3O	428.79278	antimalarial	synthetic	USP, INN, BAN		-0.08	2.24	-1.33	-0.53	0.07
01500120	AMOXICILLIN	100122-04	D03	sample	61336-70-7, 26787-78-0	C16H19N3O5S	365.41093	antibacterial	semisynthetic	USP, INN, BAN, JAN		-0.23	0.15	-1.61	-0.50	-0.55
01500122	AMPHOTERICIN B	100122-04	D04	sample	1397-89-3	C47H73NO17	924.10236	antifungal	Streptomycetes nodosus	USP, INN, BAN, JAN	New Engl J Med 296: 784 (1977)	0.32	-0.26	0.80	-0.87	0.00
01500123	AMPICILLIN SODIUM	100122-04	D05	sample	69-52-3, 69-53-4 [ampicillin]	C16H18N3NaO4S	371.39336	antibacterial	semisynthetic	USP, INN, BAN, JAN		0.33	0.85	-0.32	0.75	0.40
01500124	AMPROLIUM	100122-04	D06	sample	121-25-5	C14H20Cl2N4	315.2483	coccidiostat	synthetic	USP, INN, BAN		0.39	0.87	0.00	0.66	0.48
01500126	ANTAZOLINE PHOSPHATE	100122-04	D07	sample	154-68-7, 91-75-8 [antazoline]	C17H22N3O4P	363.35639	antihistaminic	synthetic	USP		0.46	0.01	0.70	3.82	1.25
01500127	ANTHRALIN	100122-04	D08	sample	480-22-8, 1143-38-0	C14H10O3	226.234	antipsoriatic	synthetic	USP		0.29	0.00	0.55	0.71	0.39
01500128	ANTIPYRINE	100122-04	D09	sample	60-80-0	C11H12N2O	188.23109	analgesic	synthetic	USP, INN, BAN, JAN		0.39	0.01	0.86	-0.29	0.24
01500129	APOMORPHINE HYDROCHLORIDE	100122-04	D10	sample	41372-20-7, 314-19-2	C17H18ClNO2	303.79151	emetic	synthetic	USP, BAN		0.41	-0.52	1.48	-0.58	0.20
01500130	ASPIRIN	100122-04	D11	sample	50-78-2	C9H8O4	180.16171	analgesic, antipyretic, antiinflammatory	synthetic	USP, BAN, JAN		1.71	1.16	2.34	1.07	1.57
	0.11% JAK2inhib	100122-04	D12	0.11% jak2inhib								-3.39	-3.26	-1.91	-3.28	-2.96
	0.11% JAK2inhib	100122-04	E01	0.11% jak2inhib								-3.73	-2.64	-3.46	-2.44	-3.07
01500131	ATROPINE SULFATE	100122-04	E02	sample	51-55-8, 5908-99-6 [atropine	C17H25NO7S	387.4553	anticholinergic, mydriatic	Atropa and Datura spp	USP, BAN		-0.28	-0.34	-1.30	-0.45	-0.59
01500132	AUROTHIOGLUCOSE	100122-04	E03	sample	12192-57-3	C6H11AuO5S	392.18257	antirheumatic	synthetic	USP		-0.32	0.22	-0.26	0.28	-0.02
01500133	AZATHIOPRINE	100122-04	E04	sample	446-86-6	C9H7N7O2S	277.26584	immunosuppressant, antineoplastic, antirheumatic	synthetic	USP, INN, BAN, JAN		-1.16	0.47	-0.79	-0.58	-0.52
01500134	BACITRACIN	100122-04	E05	sample	1405-87-4	C66H103N17O16S	1422.72511	antibacterial	Bacillus licheniformis and B subtilis	USP, INN, BAN, JAN		1.02	0.50	-0.27	-0.31	0.23
01500135	BACLOFEN	100122-04	E06	sample	1134-47-0	C10H12ClNO2	213.66564	muscle relaxant (skeletal)	synthetic	USP, INN, BAN, JAN		0.28	-0.19	-0.75	0.15	-0.13
01500136	BECLOMETHASONE DIPROPIONATE	100122-04	E07	sample	5534-09-8, 4419-39-0	C28H37ClO7	521.05589	antiasthmatic, topical antiinflammatory	synthetic	USP, INN, BAN, JAN		0.03	1.23	0.46	0.63	0.59
01500137	BENSERAZIDE HYDROCHLORIDE	100122-04	E08	sample	322-35-0	C10H16ClN3O5	293.70912	decarboxylase inhibitor	component of Madopa (Hoffmann-LaRoche)	USAN, INN, BAN, JAN		0.15	0.08	0.25	5.38	1.47
01500138	BENZETHONIUM CHLORIDE	100122-04	E09	sample	121-54-0	C27H42ClNO2	448.09429	antiinfective (topical)	synthetic	USP, INN, BAN, JAN		1.31	-0.53	0.75	-0.01	0.38
01500139	BENZOCAINE	100122-04	E10	sample	94-09-7	C9H11NO2	165.19352	anesthetic (topical)	synthetic	USP, INN, BAN		1.66	0.23	0.74	0.54	0.79
01500141	BENZTHIAZIDE	100122-04	E11	sample	91-33-8	C15H14ClN3O4S3	431.94153	diuretic, antihypertensive	synthetic	USP-XIII, INN, BAN, JAN		0.32	-0.14	1.17	-0.50	0.21
	0.11% DMSO	100122-04	E12	0.11% dmso								-0.61	1.27	0.68	-1.86	-0.13
	0.11% JAK2inhib	100122-04	F01	0.11% jak2inhib								-3.66	-3.68	-3.39	-3.40	-3.53
01500143	beta-CAROTENE	100122-04	F02	sample	7235-40-7	C40H56	536.89232	provitamin A	provitamin A; widespread in plants and animals	USP, INN		-7.36	-1.96	-1.78	-0.40	-2.87
01500144	BETAMETHASONE	100122-04	F03	sample	378-44-9	C22H29FO5	392.47183	glucocorticoid, antiinflammatory	semisynthetic	USP, INN, BAN, JAN		-0.87	0.63	-0.88	0.44	-0.17
01500146	BETHANECHOL CHLORIDE	100122-04	F04	sample	590-63-6, 674-38-4	C7H17ClN2O2	196.67874	cholinergic	synthetic	USP, BAN, JAN		0.00	1.37	-0.84	0.69	0.31
01500147	BISACODYL	100122-04	F05	sample	603-50-9	C22H19NO4	361.40103	cathartic	synthetic	USP, INN, BAN, JAN		0.15	0.06	-0.87	0.18	-0.12
01500148	BITHIONATE SODIUM	100122-04	F06	sample	97-18-7	C12H4Cl4Na2O2S	400.02008	anthelmintic, antiseptic	synthetic	USAN, INN, BAN, JAN		0.68	0.40	-0.44	-0.92	-0.07
01500151	BROMOCRIPTINE MESYLATE	100122-04	F07	sample	22260-51-1, 25614-03-3	C33H44BrN5O8S	750.71533	prolactin inhibitor, antiparkinsonian	semisynthetic	USP, BAN, JAN		-0.20	-1.18	-1.62	6.53	0.88
01500152	BUSULFAN	100122-04	F08	sample	55-98-1	C6H14O6S2	246.30288	antineoplastic, alkylating agent	synthetic	USP, INN, BAN, JAN		1.32	-0.59	0.85	0.77	0.59
01500155	CAFFEINE	100122-04	F09	sample	58-08-2, 5743-12-4	C8H10N4O2	194.1945	CNS stimulant	Cofea, Theobroma, Camelia and Cola spp	USP, INN, BAN, JAN		2.14	0.17	0.07	0.65	0.75
01500156	CAMPHOR (1R)	100122-04	F10	sample	464-49-3; 76-22-2	C10H16O	152.23842	analgesic, antiinfective, antipruritic	Cinnamomum camphora	USP, JAN		1.12	0.63	-0.14	0.47	0.52
01500157	CAPREOMYCIN SULFATE	100122-04	F11	sample	1405-37-4, 11003-38-6	C25H46N14O12S	766.79597	antibacterial, tuberculostatic	Streptomyces capreolis	USP, INN, BAN, JAN		0.74	-0.81	0.58	-0.12	0.10
	0.04% DMSO	100122-04	F12	0.04% dmso								0.81	-0.62	0.63	-0.89	-0.02
	0.04% JAK2inhib	100122-04	G01	0.04% jak2inhib								-2.50	-0.57	-2.72	-0.70	-1.62
01500158	CARBACHOL	100122-04	G02	sample	51-83-2	C6H15ClN2O2	182.65165	cholinergic, miotic	synthetic	USP, INN, BAN, JAN		-0.10	0.68	-1.33	-0.77	-0.38
01500159	CARBAMAZEPINE	100122-04	G03	sample	298-46-4	C15H12N2O	236.27569	analgesic, anticonvulsant	synthetic	USP, INN, BAN, JAN		-1.04	0.58	-1.34	0.02	-0.45
01500160	CARBENICILLIN DISODIUM	100122-04	G04	sample	4800-94-6, 4697-36-3	C17H16N2Na2O6S	422.37047	antibacterial	semisynthetic	USP, INN, BAN, JAN		-0.54	-0.16	-0.32	0.02	-0.25
01500161	CARBINOXAMINE MALEATE	100122-04	G05	sample	3505-38-2, 486-16-8	C20H23ClN2O5	406.86971	antihistaminic	synthetic	USP, INN, BAN, JAN		-0.07	3.24	-0.38	-0.08	0.68
01500162	CARISOPRODOL	100122-04	G06	sample	78-44-4	C12H24N2O4	260.33608	muscle relaxant (skeletal)	synthetic	USP, INN, BAN		-0.17	0.34	0.00	0.36	0.13
01500163	CEFADROXIL	100122-04	G07	sample	66592-87-8, 50370-12-2	C16H17N3O5S	363.39499	antibacterial	semisynthetic	USP, INN, BAN, JAN		0.12	-0.68	0.36	1.09	0.22
01500165	CEFOTAXIME SODIUM	100122-04	G08	sample	64485-93-4, 63527-52-6	C16H16N5NaO7S2	477.45302	antibacterial	semisynthetic	USP, INN, BAN, JAN		-0.11	0.09	0.10	4.59	1.17
01500166	CEPHALOTHIN SODIUM	100122-04	G09	sample	58-71-9, 153-61-7 [cephalothin]	C16H15N2NaO6S2	418.42555	antibacterial	semisynthetic	USP, INN, BAN, JAN		1.09	0.14	0.13	-0.54	0.20
01500167	CEPHAPIRIN SODIUM	100122-04	G10	sample	24356-60-3, 21593-23-7	C17H16N3NaO6S2	445.45137	antibacterial	semisynthetic	USP, INN, BAN, JAN		0.91	0.34	0.79	0.87	0.73
01500168	CEPHRADINE	100122-04	G11	sample	38821-53-3 [anhydrous], 58456-	C16H19N3O4S	349.41153	antibacterial	semisynthetic	USP, INN, BAN, JAN		1.08	0.51	0.95	0.04	0.64
	0.04% DMSO	100122-04	G12	0.04% dmso								0.80	-1.06	0.73	-0.25	0.06
	0.04% JAK2inhib	100122-04	H01	0.04% jak2inhib								-2.28	-1.68	2.14	-2.64	-1.12
01500169	CETYLPYRIDINIUM CHLORIDE	100122-04	H02	sample	6004-24-6, 123-03-5	C21H38ClN	339.99671	antiinfective (topical)	synthetic	USP, INN, BAN, JAN		-0.40	2.54	0.36	1.92	1.10
01500171	CHLORAMBUCIL	100122-04	H03	sample	305-03-3	C14H19Cl2NO2	304.21903	antineoplastic, alkylating agent	synthetic	USP, INN, BAN		-0.48	0.12	-1.47	-0.36	-0.55
01500172	CHLORAMPHENICOL PALMITATE	100122-04	H04	sample	530-43-8	C27H42Cl2N2O6	561.55159	antibacterial, antirickettsial	semisynthetic	USP, BAN, JAN		-6.53	-0.54	-0.47	0.34	-1.80
01500173	CHLORAMPHENICOL HEMISUCCINATE	100122-04	H05	sample	982-57-0, 56-75-7	C15H16Cl2N2O8	423.20937	antibacterial, antirickettsial, inhibits protein synthesis	semisynthetic	USP, BAN, JAN		-0.24	0.17	-0.61	0.30	-0.10
01500174	CHLORAMPHENICOL	100122-04	H06	sample	56-75-7	C11H12Cl2N2O5	323.13469	antibacterial, antirickettsial, inhibits protein synthesis	Streptomyces venezuelae	USP, INN, BAN, JAN		0.93	0.60	-0.88	0.07	0.18
01500175	CHLORCYCLIZINE HYDROCHLORIDE	100122-04	H07	sample	1620-21-9, 82-93-9 [base]	C18H22Cl2N2	337.29544	H1-antihistamine	synthetic	USP XXI, INN, BAN		-0.49	0.30	-0.63	-1.20	-0.50
01500177	CHLORHEXIDINE	100122-04	H08	sample	3697-42-5, 55-56-1	C22H32Cl4N10	578.37934	antibacterial (topical), disinfectant	synthetic	USP, INN, BAN, JAN		0.92	0.17	0.53	0.28	0.48
01500178	CHLOROCRESOL	100122-04	H09	sample	59-50-7	C7H7ClO	142.58624	antiinfectant	synthetic	USAN, NF, INN		0.14	1.20	0.14	1.08	0.64

												Screen Score (z-score)				
**Compound ID**	**MolNAME**	**plate**	**position**	**Content**	**cas#**	**Formula**	**MolWt**	**Bioactivity**	**Source**	**Status**	**Reference**	**rep1**	**rep2**	**rep3**	**rep4**	**Average**
01500179	CHLOROQUINE DIPHOSPHATE	100122-04	H10	sample	54-05-7	C18H32ClN3O8P2	515.87164	antimalarial, antiamebic, antirheumatic, intercalating agent	synthetic	USP, BAN		0.80	-0.23	2.12	-0.10	0.65
01500180	CHLOROTHIAZIDE	100122-04	H11	sample	58-94-6	C7H6ClN3O4S2	295.72457	diuretic, antihypertensive	synthetic	USP, INN, BAN		2.17	0.30	0.53	0.29	0.82
	empty	100122-04	H12	sample								NA	NA	NA	NA	NA
	empty	100122-05	A01	sample								NA	NA	NA	NA	NA
01500181	CHLOROTRIANISENE	100122-05	A02	sample	569-57-3	C23H21ClO3	380.87502	estrogen	synthetic	USP-XIII, INN, BAN		-0.41	-0.50	-1.51	0.16	-0.56
01500182	CHLOROXYLENOL	100122-05	A03	sample	88-04-0	C8H9ClO	156.61333	antibacterial, topical and urinary antiseptic	synthetic	USP, INN, BAN		0.02	-0.53	-1.26	0.39	-0.35
01500183	CHLORPHENIRAMINE (S) MALEATE	100122-05	A04	sample	113-92-8, 132-22-9	C20H23ClN2O4	390.87031	antihistaminic	synthetic	USP, INN, BAN		-0.82	-0.44	0.35	-0.67	-0.39
01500184	CHLORPROMAZINE	100122-05	A05	sample	50-53-3	C17H19ClN2S	318.87138	antiemetic, antipsychotic	synthetic	USP, INN, BAN, JAN		-0.05	2.54	-0.76	-0.11	0.41
01500185	CHLORPROPAMIDE	100122-05	A06	sample	94-20-2	C10H13ClN2O3S	276.74371	antidiabetic	synthetic	USP, INN, BAN, JAN		0.05	-0.97	0.28	0.21	-0.11
01500186	CHLORTETRACYCLINE HYDROCHLORIDE	100122-05	A07	sample	64-72-2	C22H24Cl2N2O8	515.35118	antibacterial, antiamebic, Ca chelator, hepatotoxic; inhibits protein	Streptomyces aureofaciens	USP, BAN	Ann NY Acad Sci 51: 177 (1948); JACS 74: 4976 (1952);	0.05	-0.82	-0.43	-0.03	-0.31
01500187	CHLORTHALIDONE	100122-05	A08	sample	77-36-1	C14H11ClN2O4S	338.77177	diuretic, antihypertensive	synthetic	USP, INN, BAN, JAN		-0.49	-0.34	-0.52	-0.10	-0.36
01500188	CHLORZOXAZONE	100122-05	A09	sample	95-25-0	C7H4ClNO2	169.56843	muscle relaxant (skeletal)	synthetic	USP, INN, BAN, JAN		-0.40	-0.14	0.77	0.69	0.23
01500189	CICLOPIROX OLAMINE	100122-05	A10	sample	41621-49-2	C14H24N2O3	268.35898	antifungal	synthetic	USP, INN, BAN		-0.99	-1.94	-0.32	-0.57	-0.95
01500190	CINOXACIN	100122-05	A11	sample	28657-80-9	C12H10N2O5	262.2239	antibacterial	synthetic	USP, INN, BAN, JAN		0.00	-0.88	0.04	-0.50	-0.34
	0.04% JAK2inhib	100122-05	A12	0.04% jak2inhib								-1.16	-1.31	-0.18	-1.85	-1.12
	0.04% DMSO	100122-05	B01	0.04% dmso								0.35	0.24	-1.29	-0.20	-0.23
01500191	CLEMASTINE	100122-05	B02	sample	15686-51-8	C25H30ClNO5	459.97455	antihistaminic	synthetic	USAN, BAN		-0.44	-0.27	-0.67	-0.25	-0.41
01500192	CLIDINIUM BROMIDE	100122-05	B03	sample	3485-62-9	C22H26BrNO3	432.36142	anticholinergic	synthetic	USP, INN, BAN		0.29	0.14	-0.10	0.06	0.10
01500193	CLINDAMYCIN HYDROCHLORIDE	100122-05	B04	sample	21462-39-5, 58207-19-5	C18H34Cl2N2O5S	461.45208	antibacterial, inhibits protein synthesis	semisynthetic; U-21251	USAN, INN, BAN		-0.24	0.37	3.90	-0.38	0.91
01500196	CLOMIPHENE CITRATE	100122-05	B05	sample	50-41-9, 911-45-5 [clomiphene]	C32H36ClNO8	598.09862	gonad stimulating principle	synthetic	USP, INN, BAN		-0.70	-0.72	-0.01	0.50	-0.23
01500198	CLONIDINE HYDROCHLORIDE	100122-05	B06	sample	4205-91-8, 4205-90-7	C9H10Cl3N3	266.55915	antihypertensive	synthetic	USP, INN, BAN		0.37	-0.17	0.59	1.54	0.58
01500200	CLOTRIMAZOLE	100122-05	B07	sample	23593-75-1	C22H17ClN2	344.84719	antifungal	synthetic	USP, INN, BAN, JAN		-1.49	-1.42	-0.98	-2.76	-1.66
01500201	CLOXACILLIN SODIUM	100122-05	B08	sample	7081-44-9, 642-78-4	C19H17ClN3NaO5S	457.87124	antibacterial	semisynthetic	USP, INN, BAN, JAN		-0.58	0.55	0.32	1.29	0.40
01500202	CLOXYQUIN	100122-05	B09	sample	130-16-5	C9H6ClNO	179.60727	antibacterial, antifungal	synthetic	USAN, INN		0.69	0.54	0.39	0.26	0.47
01500205	COLCHICINE	100122-05	B10	sample	64-86-8	C22H25NO6	399.44765	antimitotic, antigout agent	Colchicum autumnale	USP, JAN	J Am Chem Soc 74: 487 (1952)	-8.48	-7.47	-7.62	-7.13	-7.67
01500206	COLISTIMETHATE SODIUM	100122-05	B11	sample	8068-28-8, 21362-08-3	C57H103N16Na5O28S	1735.81586	antibacterial	Bacillus colistinus	USP, INN, BAN, JAN	Brit.J.Pharmacol.Chemother. 23: 552 (1964)	-0.37	1.30	0.56	3.01	1.13
	0.04% JAK2inhib	100122-05	B12	0.04% jak2inhib								-0.82	-2.31	0.33	-1.12	-0.98
	0.04% DMSO	100122-05	C01	0.04% dmso								-0.90	-1.37	-2.33	-0.66	-1.31
01500207	CORTISONE ACETATE	100122-05	C02	sample	50-04-4, 53-06-5 [cortisone]	C23H30O6	402.49195	glucocorticoid	semisynthetic	USP, INN, BAN, JAN		0.50	0.46	-0.66	4.40	1.17
01500208	COTININE	100122-05	C03	sample	486-56-6, 5695-98-7[fumarate]	C10H12N2O	176.21994	antidepressant	Nicotiana tabacum	USAN, INN	JACS 79: 149 (1957)	0.66	0.53	-0.76	0.77	0.30
01500209	CRESOL	100122-05	C04	sample	1319-77-3	C7H8O	108.14121	antiinfectant	coal tar	USAN, NF, JAN		-0.12	0.51	-0.55	0.44	0.07
01500210	CROMOLYN SODIUM	100122-05	C05	sample	15826-37-6, 16110-51-3	C23H14Na2O11	512.34103	antiasthmatic, antiallergy	synthetic	USP, INN, BAN, JAN		0.17	0.75	0.01	0.49	0.36
01500211	CYCLIZINE	100122-05	C06	sample	82-92-8	C18H22N2	266.38944	H1 antihistamine	synthetic	USP, BAN		0.55	1.48	0.08	2.89	1.25
01500212	CYCLOPENTOLATE HYDROCHLORIDE	100122-05	C07	sample	5870-29-1, 512-15-2	C17H26ClNO3	327.85467	mydriatic	synthetic	USP, INN, BAN, JAN		0.06	-0.02	-0.02	0.82	0.21
01500213	CYCLOPHOSPHAMIDE HYDRATE	100122-05	C08	sample	6055-19-2, 50-18-0	C7H17Cl2N2O3P	279.10494	antineoplastic, alkylating agent	synthetic	USP, INN, BAN, JAN		0.86	-0.20	-0.32	0.18	0.13
01500215	CYCLOSERINE	100122-05	C09	sample	68-41-7	C3H6N2O2	102.09347	antibacterial (tuberculostatic)	Streptomyces spp	USP, INN, BAN, JAN		1.76	-0.42	0.07	1.40	0.70
01500216	CYPROTERONE ACETATE	100122-05	C10	sample	427-51-0	C24H29ClO4	416.94933	antiandrogen	synthetic	USP		0.16	0.60	0.79	-0.34	0.30
01500217	CYTARABINE	100122-05	C11	sample	147-94-4	C9H13N3O5	243.22106	antineoplastic, antiviral, antimetabolite	synthetic	USP, INN, BAN, JAN		-0.57	-1.88	-1.41	-1.86	-1.43
	0.11% JAK2inhib	100122-05	C12	0.11% jak2inhib								-1.78	-2.41	-1.01	-1.61	-1.70
	0.11% DMSO	100122-05	D01	0.11% dmso								-0.38	0.29	-0.78	-1.49	-0.59
01500218	DACARBAZINE	100122-05	D02	sample	4342-03-4	C6H10N6O	182.1862	antineoplastic	synthetic	USP, INN, BAN, JAN		0.13	-0.11	-0.39	0.43	0.01
01500220	DANAZOL	100122-05	D03	sample	17230-88-5	C22H27NO2	337.46599	anterior pituitary suppressant	synthetic	USP, INN, BAN, JAN		-0.71	0.71	1.61	-0.41	0.30
01500222	DAPSONE	100122-05	D04	sample	80-08-0	C12H12N2O2S	248.30564	antibacterial, leprostatic, dermatitis herpetiformis suppressant	synthetic	USP, INN, BAN		1.20	0.29	-0.08	0.23	0.41
01500223	DAUNORUBICIN	100122-05	D05	sample	20830-81-3	C27H29NO10	527.53288	antineoplastic	Streptomyces peucetius; FI-6339, NDC-0082-4155, RP-13057	USAN, INN, BAN, JAN		-8.14	-7.08	-5.60	-2.35	-5.79
01500225	SODIUM DEHYDROCHOLATE	100122-05	D06	sample	145-41-5	C24H33NaO5	424.51741	choleretic	semisynthetic	USP, INN, BAN, JAN		0.36	1.11	0.26	0.50	0.56
01500226	DEMECLOCYCLINE HYDROCHLORIDE	100122-05	D07	sample	127-33-3	C21H22Cl2N2O8	501.32409	antibacterial	Streptomyces aureofaiens	USP, BAN, JAN		2.37	0.49	0.75	0.65	1.07
01500227	DESIPRAMINE HYDROCHLORIDE	100122-05	D08	sample	58-28-6, 50-47-5 [desipramine]	C18H23ClN2	302.85041	antidepressant	synthetic	USP, INN, BAN, JAN		-0.47	0.02	-0.26	1.89	0.29
01500230	DEXAMETHASONE	100122-05	D09	sample	50-02-2	C22H29FO5	392.47183	glucocorticoid	semisynthetic	USP, INN, BAN, JAN		0.22	0.37	0.14	5.68	1.61
01500231	DEXAMETHASONE ACETATE	100122-05	D10	sample	55812-90-3, 1177-87-3	C24H31FO6	434.50947	glucocorticoid, antiinflammatory	semisynthetic	USP, INN, BAN, JAN		0.21	0.35	1.54	-0.60	0.37
01500224	DEFEROXAMINE MESYLATE	100122-05	D11	sample	138-14-7, 70-51-9	C26H52N6O11S	656.80194	chelating agent (Fe & Al)	Strptomyces pilosus	USAN, INN, BAN		1.23	0.75	0.33	-0.41	0.47
	0.11% JAK2inhib	100122-05	D12	0.11% jak2inhib								-3.78	-2.78	-3.01	1.96	-1.90
	0.11% JAK2inhib	100122-05	E01	0.11% jak2inhib								-2.05	-2.77	-4.04	-2.14	-2.75
01500232	DEXAMETHASONE SODIUM PHOSPHATE	100122-05	E02	sample	2392-39-4, 312-93-6	C22H28FNa2O8P	516.41546	glucocorticoid, antiinflammatory	semisynthetic	USP, BAN, JAN		-0.07	0.61	-0.06	-0.29	0.05
01500233	DEXTROMETHORPHAN HYDROBROMIDE	100122-05	E03	sample	6700-34-1, 125-69-9	C18H26BrNO	352.31802	antitussive	synthetic	USP, INN, BAN		-0.11	1.39	-0.79	1.32	0.45
01500235	DIBENZOTHIOPHENE	100122-05	E04	sample	132-65-0	C12H8S	184.26156	keratolytic	synthetic	USAN		-0.10	0.27	-0.46	-0.25	-0.14
01500236	DIBUCAINE HYDROCHLORIDE	100122-05	E05	sample	61-12-1, 85-79-0 [dibucaine]	C20H30ClN3O2	379.934	anesthetic (local)	synthetic	USP, INN, BAN		0.76	-0.12	-1.04	0.48	0.02
01500237	DICLOFENAC SODIUM	100122-05	E06	sample	15307-79-6	C14H10Cl2NNaO2	318.1371	antiinflammatory	synthetic	USP, JAN		0.41	-0.02	0.56	-0.11	0.21
01500238	DICLOXACILLIN SODIUM	100122-05	E07	sample	13412-64-1, 343-55-5	C19H16Cl2N3NaO5S	492.31627	antibacterial	semisynthetic	USAN, INN, BAN		0.17	0.16	-0.13	0.03	0.06
01500239	DICUMAROL	100122-05	E08	sample	66-76-2	C19H12O6	336.30389	anticoagulant	synthetic	USAN, INN	J Biol Chem 138:21, 513, 529 (1041); 142:941 (1942)	1.75	-0.29	2.15	2.08	1.42
01500240	DICYCLOMINE HYDROCHLORIDE	100122-05	E09	sample	67-92-5, 77-19-0 [dicyclomine]	C19H36ClNO2	345.95727	anticholinergic	synthetic	USP, INN, BAN, JAN		0.22	0.27	0.03	4.43	1.24
01500241	DIENESTROL	100122-05	E10	sample	84-17-3, 13029-44-2 [//E,E//]	C18H18O2	266.34296	estrogen	synthetic	USP, INN, BAN		0.83	0.17	0.54	-0.70	0.21
01500242	DIETHYLCARBAMAZINE CITRATE	100122-05	E11	sample	1642-54-2, 90-89-1	C16H29N3O8	391.42483	anthelmintic	synthetic	USP, INN, BAN, JAN		1.40	-0.08	1.15	4.60	1.77
	0.11% DMSO	100122-05	E12	0.11% dmso								-0.10	-1.03	0.69	-0.69	-0.28
	0.11% JAK2inhib	100122-05	F01	0.11% jak2inhib								-3.00	-2.94	-4.28	2.78	-1.86
01500244	DIETHYLSTILBESTROL	100122-05	F02	sample	56-53-1	C18H20O2	268.3589	estrogen	synthetic	USP, INN, BAN		-1.15	-1.36	-2.98	-0.48	-1.49
01500245	DIFLUNISAL	100122-05	F03	sample	22494-42-4	C13H8F2O3	250.20371	analgesic, antiinflammatory	synthetic	USP, INN, BAN, JAN		-0.67	0.31	-0.62	-0.07	-0.26
01500246	DIGITOXIN	100122-05	F04	sample	71-63-6	C41H64O13	764.95943	inotropic, cardiotonic	Digitalis spp	USP, INN, BAN, JAN	Chem Rev 17: 187 (1935); Anal Profiles Drug Subst 3:149	-5.20	-1.60	-3.04	-2.20	-3.01
01500247	DIGOXIN	100122-05	F05	sample	20830-75-5	C41H64O14	780.95883	cardiac stimulant	Digitalis lanata or D. orientalis Lam., Scrophulariaceae	USP, INN, BAN, JAN	J.Chem.Soc.1930: 508; 1954: 2012	-0.16	0.21	-0.51	3.36	0.73
01500248	DIHYDROERGOTAMINE MESYLATE	100122-05	F06	sample	6190-39-2	C34H41N5O8S	679.79857	vasoconstrictor, antimigraine	semisynthetic	USP, INN, BAN, JAN		0.12	0.35	0.05	-0.16	0.09
01500249	DIHYDROSTREPTOMYCIN SULFATE	100122-05	F07	sample	5490-27-7, 128-46-1	C21H43N7O16S	681.67816	antibacterial, tuberculostatic	Streptomyces humidus	USP, INN, BAN, JAN		0.24	1.01	0.37	2.42	1.01
01500251	DIMENHYDRINATE	100122-05	F08	sample	523-87-5	C24H28ClN5O3	469.97546	antiemetic	synthetic	USP, INN, BAN, JAN		0.00	0.14	-0.02	-0.49	-0.09
01500253	DIMETHADIONE	100122-05	F09	sample	695-53-4	C5H7NO3	129.11644	anticonvulsant	synthetic	USAN, INN		0.76	0.77	0.67	-0.64	0.39
01500255	DIOXYBENZONE	100122-05	F10	sample	131-53-3	C14H12O4	244.24934	ultraviolet screen	synthetic	USP, INN		0.78	-0.09	0.76	-1.33	0.03
01500256	DIPHENHYDRAMINE HYDROCHLORIDE	100122-05	F11	sample	147-24-0	C17H22ClNO	291.82399	antihistaminic	synthetic	USP, INN, BAN, JAN		0.77	0.15	0.31	-1.01	0.06
	0.04% DMSO	100122-05	F12	0.04% dmso								1.06	0.53	0.29	-1.38	0.12
	0.04% JAK2inhib	100122-05	G01	0.04% jak2inhib								-1.78	-1.48	-2.41	-1.37	-1.76
01500258	DIPHENYLPYRALINE HYDROCHLORIDE	100122-05	G02	sample	132-18-3 147-20-6	C19H24ClNO	317.86223	antihistaminic	synthetic	USP-XXI, INN, BAN, JAN		-0.48	-0.09	-0.70	-0.11	-0.35
01500259	DIPYRIDAMOLE	100122-05	G03	sample	58-32-2	C24H40N8O4	504.6376	coronary vasodilator	synthetic	USP, INN, BAN, JAN		0.10	-0.13	-1.83	-0.66	-0.63
01500260	PYRITHIONE ZINC	100122-05	G04	sample	13463-41-7	C10H10N2O2S2Zn	319.7014	antibacterial, antifungal, antiseborrheic	synthetic	USAN, INN, BAN		-2.57	-3.27	-4.17	-2.76	-3.19
01500261	DISOPYRAMIDE PHOSPHATE	100122-05	G05	sample	3737-09-5	C21H32N3O5P	437.48009	antiarrhythmic	synthetic	USP, INN, BAN, JAN		0.40	0.29	-0.61	0.79	0.22
01500262	DISULFIRAM	100122-05	G06	sample	97-77-8	C10H20N2S4	296.5403	alcohol antagonist	synthetic	USP, INN, BAN, JAN		-1.96	-3.75	-0.92	0.16	-1.62
01500263	DOPAMINE HYDROCHLORIDE	100122-05	G07	sample	62-31-7, 51-61-6 [dopamine]	C8H12ClNO2	189.64334	cardiotonic, antihypotensive	synthetic	USP, INN, BAN, JAN		-0.49	-0.07	0.71	-0.75	-0.15
01500264	DOXEPIN HYDROCHLORIDE	100122-05	G08	sample	1229-29-4, 1668-19-5	C19H22ClNO	315.84629	antidepressant	synthetic	USP, INN, BAN		-0.36	0.13	0.45	-1.13	-0.23
01500266	DOXYCYCLINE HYDROCHLORIDE	100122-05	G09	sample	17086-28-1, 564-25-0	C22H25ClN2O8	480.90615	antibacterial	semisynthetic; GS-3065	USP, INN, BAN		0.35	0.45	0.17	3.93	1.23
01500267	DOXYLAMINE SUCCINATE	100122-05	G10	sample	562-10-7, 469-21-6	C21H28N2O5	388.46771	antihistaminic, hypnotic	synthetic	USP, INN, BAN		0.49	-0.32	0.10	-0.59	-0.08
01500268	DYCLONINE HYDROCHLORIDE	100122-05	G11	sample	536-43-6, 586-60-7 [dyclonine]	C18H28ClNO2	325.88236	anesthetic (topical)	synthetic	USP, INN, BAN		-0.29	-0.19	0.50	0.04	0.01
	0.04% DMSO	100122-05	G12	0.04% dmso								0.71	-0.14	-0.16	0.05	0.12
	0.04% JAK2inhib	100122-05	H01	0.04% jak2inhib								-1.00	-2.40	-1.81	0.40	-1.20
01500269	DYPHYLLINE	100122-05	H02	sample	479-18-5	C10H14N4O4	254.24748	PDE inhibitor, bronchodilator, vasodilator	synthetic	USP, INN, BAN, JAN		-0.38	-0.52	-1.48	-0.62	-0.75
01500270	TRISODIUM ETHYLENEDIAMINE TETRACETATE	100122-05	H03	sample	150-38-9	C10H16N2Na3O8	361.21702	chelating agent, antioxidant	synthetic	USAN		-0.06	-0.03	-1.44	0.98	-0.14
01500272	EMETINE	100122-05	H04	sample	316-42-7, 483-18-1 [emetine]	C29H42Cl2N2O4	553.57509	inhibits RNA, DNA and protein synthesis	Uragoga ipecacuanha	USP, BAN	Nature 162: 524 (1948)	-4.57	-3.97	-5.54	-0.18	-3.57
01500274	ADRENALINE BITARTRATE	100122-05	H05	sample	51-42-3	C13H19NO9	333.29768	adrenergic agonist, bronchodilator, antiglaucoma agent	Portulaca grandiflora	USP, JAN		0.37	0.41	-0.17	0.05	0.17
01500275	EQUILIN	100122-05	H06	sample	474-86-2	C18H20O2	268.3589	estrogen	pregnant mare urine	USP		-0.31	0.27	0.71	3.53	1.05
01500276	ERGOCALCIFEROL	100122-05	H07	sample	50-14-6	C28H44O	396.66228	antirachitic vitamin; LD50 (rat) 56 mg/kg po	irradiation of ergosterol	USP, INN, BAN, JAN	Steroids 1959:90; Prog Chem Org Nat Prod 27:131 (1969)	-6.43	2.50	0.55	-0.40	-0.95
01500277	ERGONOVINE MALEATE	100122-05	H08	sample	129-51-1, 60-79-7 [ergonovine]	C23H27N3O6	441.48814	oxytocic, 5HT antagonist	ergot and Convolvulvaceae spp	USP, INN, BAN, JAN		-0.48	0.62	0.16	0.24	0.13
01500279	ERYTHROMYCIN ETHYLSUCCINATE	100122-05	H09	sample	1264-62-6, 114-07-8	C43H75NO16	862.0743	antibacterial	semisynthetic	USP, JAN		-2.89	-0.10	1.51	-0.40	-0.47
01500282	ESTRADIOL	100122-05	H10	sample	50-28-2	C18H24O2	272.39078	estrogen	pregnancy urine	USP, INN, BAN		0.60	-0.32	0.93	-0.09	0.28
01500283	ESTRADIOL CYPIONATE	100122-05	H11	sample	313-06-4	C26H36O3	396.57502	estrogen	semisynthetic	USP		-1.91	-0.22	0.07	-0.95	-0.76
	empty	100122-05	H12	sample								NA	NA	NA	NA	NA
	empty	100122-06	A01	sample								NA	NA	NA	NA	NA
01500284	ESTRADIOL VALERATE	100122-06	A02	sample	979-32-8	C23H32O3	356.50969	estrogen	semisynthetic	USP		0.22	0.69	1.01	-0.83	0.27
01500285	ESTRIOL	100122-06	A03	sample	50-27-1, 514-68-1 [as,	C18H24O3	288.39018	estrogen	mammalian hormone	USP, INN, BAN, JAN	JACS 76:2943 (1954); Biochem Prep 12:81 (1968); J	1.04	-0.84	-1.05	1.24	0.10
01500286	ESTRONE	100122-06	A04	sample	53-16-7	C18H22O2	270.37484	estrogen	pregnacy urine, dates and Punica granatum seeds	USP, INN, BAN		-0.39	-2.47	-1.79	-0.65	-1.32
01500287	ETHACRYNIC ACID	100122-06	A05	sample	58-54-8	C13H12Cl2O4	303.14419	diuretic	synthetic	USP, INN, BAN, JAN		0.23	-2.40	-0.51	-1.68	-1.09
01500288	ETHAMBUTOL HYDROCHLORIDE	100122-06	A06	sample	1070-11-7, 74-55-5	C10H26Cl2N2O2	277.23692	antibacterial (tuberculostatic)	synthetic	USP, INN, BAN, JAN		0.73	-0.56	0.11	-1.21	-0.23
01500291	ETHINYL ESTRADIOL	100122-06	A07	sample	57-63-6	C20H24O2	296.41308	estrogen, plus progestogen as oral contraceptive	semisynthetic	USP, INN, BAN, JAN		0.97	-2.10	-1.03	-0.68	-0.71
01500292	ETHIONAMIDE	100122-06	A08	sample	536-33-4	C8H10N2S	166.2463	antibacterial, tuberculostatic	synthetic	USP, INN, BAN, JAN		-0.09	-1.20	-0.18	1.91	0.11
01500293	ETHOPROPAZINE HYDROCHLORIDE	100122-06	A09	sample	1094-08-2, 522-00-9	C19H25ClN2S	348.9415	antiparkinsonian, anticholinergic	synthetic	USP-XXIII, INN, BAN		0.90	-1.12	-0.18	1.42	0.25
01500295	EUCATROPINE HYDROCHLORIDE	100122-06	A10	sample	536-93-6, 100-91-4	C17H26ClNO3	327.85467	anticholinergic (opthalmic)	semisynthetic	USP, INN, BAN		1.55	-0.14	0.72	1.10	0.81
01500296	EUGENOL	100122-06	A11	sample	97-53-0	C10H12O2	164.20594	analgesic (topical), antiseptic, antifungal	common in plant essential oils	USP		1.51	0.13	0.45	0.25	0.58
	0.04% JAK2inhib	100122-06	A12	0.04% jak2inhib								0.63	-0.19	4.35	2.14	1.73
	0.04% DMSO	100122-06	B01	0.04% dmso								-3.15	-5.85	-3.30	5.34	-1.74
01500299	FLUDROCORTISONE ACETATE	100122-06	B02	sample	514-36-3, 127-31-1	C23H31FO6	422.49832	mineralocorticoid	semisynthetic	USP, INN, BAN, JAN		-2.15	-3.63	-1.82	-0.56	-2.04
01500300	FLUMETHAZONE PIVALATE	100122-06	B03	sample	2002-29-1, 2135-17-3	C27H36F2O6	494.58117	glucocorticoid, antiinflammatory	semisynthetic	USP, INN, BAN, JAN		-1.12	-2.38	-2.12	-0.31	-1.48
01500302	FLUOCINOLONE ACETONIDE	100122-06	B04	sample	67-73-2	C24H30F2O6	452.4999	glucocorticoid, antiinflammatory	semisynthetic	USP, INN, BAN, JAN		-0.56	-1.57	0.09	-1.32	-0.84
01500303	FLUOCINONIDE	100122-06	B05	sample	356-12-7	C26H32F2O7	494.53754	antiinflammatory, glucocorticoid	semisynthetic	USP, INN, BAN, JAN		-0.86	-0.89	0.09	-0.54	-0.55
01500304	FLUOROMETHOLONE	100122-06	B06	sample	426-13-1	C22H29FO4	376.47243	glucocorticoid, antiinflammatory	semisynthetic	USP, INN, BAN, JAN		0.28	-0.06	0.27	0.94	0.36
01500305	FLUOROURACIL	100122-06	B07	sample	51-21-8	C4H3FN2O2	130.07911	antineoplastic, pyrimidine antimetabolite	synthetic	USP, INN, BAN, JAN		-0.56	1.11	0.22	3.00	0.94
01500308	FLURBIPROFEN	100122-06	B08	sample	5104-49-4	C15H13FO2	244.26806	antiinflammatory, analgesic	synthetic	USP, INN, BAN, JAN		0.40	-0.29	-0.16	0.32	0.07
01500309	FURAZOLIDONE	100122-06	B09	sample	67-45-8	C8H7N3O5	225.16209	antibacterial	NF-180; synthetic	USP, INN, BAN		-1.84	-3.02	-3.21	-3.47	-2.88
01500310	FUROSEMIDE	100122-06	B10	sample	54-31-9	C12H11ClN2O5S	330.74887	diuretic, antihypertensive	synthetic	USP, INN, BAN, JAN		0.36	0.02	1.26	-0.28	0.34
01500311	FUSIDIC ACID	100122-06	B11	sample	6990-06-3	C31H48O6	516.72461	antibacterial	Fusidium spp	USAN, INN, BAN	Tetrahedron 21: 3505 (1965)	-0.97	0.61	-0.16	0.58	0.01
	0.04% JAK2inhib	100122-06	B12	0.04% jak2inhib								-0.74	-3.38	-0.72	-0.46	-1.33
	0.04% DMSO	100122-06	C01	0.04% dmso								-1.10	-3.87	-4.02	-1.81	-2.70
01500312	GALLAMINE TRIETHIODIDE	100122-06	C02	sample	65-29-2, 153-76-4 [gallamine]	C30H60I3N3O3	891.5442	muscle relaxant (skeletal)	synthetic	USP, INN		-0.58	-3.48	-3.54	-1.20	-2.20
01500313	GEMFIBROZIL	100122-06	C03	sample	25812-30-0	C15H22O3	250.34079	antihyperlipoproteinemic	synthetic	USP, INN, BAN		1.49	-2.41	-1.73	0.50	-0.54
01500314	GENTAMICIN SULFATE	100122-06	C04	sample	1405-41-0, 1403-66-3	C21H45N5O11S	575.6837	antibacterial	Micromonospora spp	USP, INN, BAN, JAN		-0.38	-0.35	2.60	0.58	0.61
01500315	GENTIAN VIOLET	100122-06	C05	sample	548-62-9	C25H30ClN3	407.99095	antibacterial, anthelmintic	synthetic	USP, INN, BAN, JAN		-3.58	-1.76	-1.73	-2.13	-2.30

														Screen Score (z-score)		
Compound ID	MolNAME	plate	position	Content	cas#	Formula	MolWt	Bioactivity	Source	Status	Reference	rep1	rep2	rep3	rep4	Average
01500316	GLUCOSAMINE HYDROCHLORIDE	100122-06	C06	sample	3416-24-8	C6H14ClNO5	215.63518	antiarthritic	polysaccharides in bacteria, fungi, higher plants, invertebrates,	USAN, NF-XXI	Adv Carbohydr Chem 15:159 (1960); Methods Carbohydr	0.76	0.55	-0.38	0.58	0.38
01500319	GRAMICIDIN	100122-06	C07	sample	1405-97-6	C60H92N12O10	1141.47664	antibacterial	Bacillus brevis	USP		-2.65	-0.70	-1.74	-0.13	-1.30
01500321	GUAIFENESIN	100122-06	C08	sample	93-14-1	C10H14O4	198.22068	expectorant	synthetic	USP, INN, BAN, JAN		-0.81	0.37	0.28	0.31	0.04
01500322	GUANABENZ ACETATE	100122-06	C09	sample	23256-50-0	C10H12Cl2N4O2	291.13874	antihypertensive	synthetic	USP, JAN		2.04	1.89	1.46	0.74	1.53
01500323	GUANETHIDINE SULFATE	100122-06	C10	sample	60-02-6, 55-65-2	C10H24N4O4S	296.39118	antihypertensive, mitotic agent	synthetic	USP, INN, BAN		1.08	0.92	0.91	0.57	0.87
01500324	HALAZONE	100122-06	C11	sample	80-13-7	C7H5Cl2NO4S	270.0922	antiinfectant	synthetic	USP, INN		0.65	0.00	2.86	1.45	1.24
	0.11% JAK2inhib	100122-06	C12	0.11% jak2inhib								-1.45	-3.54	-2.08	-1.47	-2.14
	0.11% DMSO	100122-06	D01	0.11% dmso								-0.99	-0.12	3.81	0.93	0.91
01500325	HALOPERIDOL	100122-06	D02	sample	52-86-8	C21H23ClFNO2	375.87436	antidyskinetic, antipsychotic	synthetic	USP, INN, BAN, JAN		-1.75	-1.90	-0.97	-0.02	-1.16
01500327	HETACILLIN POTASSIUM	100122-06	D03	sample	5321-32-4, 3511-16-8	C19H22KN3O4S	427.57089	antibacterial	semisynthetic	USP-XIII, JAN		-0.47	-1.34	-0.58	4.54	0.54
01500328	HEXACHLOROPHENE	100122-06	D04	sample	70-30-4	C13H6Cl6O2	406.90957	antiinfective (topical)	synthetic	USP, INN, BAN		-0.31	1.10	1.92	0.45	0.79
01500330	HEXYLRESORCINOL	100122-06	D05	sample	136-77-6	C12H18O2	194.27606	anthelmintic, topical antiseptic	synthetic	USP, BAN		-0.12	1.34	-0.67	-0.23	0.08
01500331	HISTAMINE DIHYDROCHLORIDE	100122-06	D06	sample	51-45-6 [histamine]	C5H11Cl2N3	184.06952	H receptor agonist, induces edema in mammalian tissues; gastric	Claviceps purpurea, many plants and animals	USAN	JACS 40:1716 (1918); J Biol Chem 180:703 (1949);	0.13	0.66	2.16	-0.02	0.73
01500332	HOMATROPINE BROMIDE	100122-06	D07	sample	51-56-9, 87-00-3 [homatropine]	C16H22BrNO3	356.26264	anticholinergic (opthalmic)	semisynthetic	USP, JAN		0.24	1.02	0.58	1.17	0.75
01500333	HOMATROPINE METHYLBROMIDE	100122-06	D08	sample	80-49-9, 87-00-3 [homatropine]	C17H24BrNO3	370.28973	anticholinergic (opthalmic)	semisynthetic	USP, INN, BAN		0.02	0.80	0.80	0.70	0.58
01500334	HYDRALAZINE HYDROCHLORIDE	100122-06	D09	sample	304-20-1, 86-54-4 [hydralazine]	C8H9ClN4	196.64073	antihypertensive	semisynthetic	USP, INN, BAN		-1.47	-1.03	-0.18	-0.67	-0.84
01500335	HYDROCHLOROTHIAZIDE	100122-06	D10	sample	58-93-5	C7H8ClN3O4S2	297.74051	diuretic	semisynthetic	USP, INN, BAN, JAN		0.38	-0.47	1.40	0.93	0.56
01500338	HYDROCORTISONE ACETATE	100122-06	D11	sample	50-03-3	C23H32O6	404.50789	glucocorticoid, antiinflammatory	semisynthetic	USP, INN, BAN, JAN		1.59	-0.11	1.20	-0.45	0.56
	0.11% JAK2inhib	100122-06	D12	0.11% jak2inhib								-2.36	-3.85	-1.14	0.36	-1.75
	0.11% JAK2inhib	100122-06	E01	0.11% jak2inhib								2.17	0.94	-0.47	-2.11	0.13
01500339	HYDROCORTISONE HEMISUCCINATE	100122-06	E02	sample	83784-20-7, 2203-97-6	C25H34O8	462.54493	glucocorticoid	semisynthetic	USP, JAN		-1.89	0.45	-2.57	2.06	-0.49
01500340	HYDROCORTISONE PHOSPHATE TRIETHYLAMINE	100122-06	E03	sample	3863-59-0 [cortisol 21-	C33H61N2O8P	644.83652	glucocorticoid	semisynthetic	USP, INN, BAN, JAN		-0.60	2.37	-0.84	0.00	0.23
01500341	HYDROFLUMETHIAZIDE	100122-06	E04	sample	135-09-1	C8H8F3N3O4S2	331.29386	antihypertensive, diuretic	synthetic	USP, INN, BAN, JAN		0.12	0.20	-0.21	0.30	0.10
01500343	HYDROXYPROGESTERONE CAPROATE	100122-06	E05	sample	630-56-8, 68-96-2	C27H40O4	428.61745	progestogen	semisynthetic	USP, INN, JAN		0.01	0.38	-2.25	-1.38	-0.81
01500344	HYDROXYUREA	100122-06	E06	sample	127-07-1	CH4N2O2	76.05523	antineoplastic, inhibits ribonucleoside diphosphate reductase	synthetic	USP, INN, BAN		-4.05	-1.04	-2.79	-1.43	-2.32
01500345	HYDROXYZINE PAMOATE	100122-06	E07	sample	10246-75-0, 68-88-2	C44H43ClN2O8	763.29491	anxiolytic, antihistaminic	synthetic	USP, JAN		-1.00	0.94	-0.93	-0.64	-0.41
01500346	HYOSCYAMINE	100122-06	E08	sample	101-31-5	C17H23NO3	289.37776	anticholinergic, analgesic	Atropa, Datura and Hyoscyamus spp	USP, BAN		-0.53	0.60	-1.02	0.25	-0.17
01500347	IBUPROFEN	100122-06	E09	sample	15687-27-1, 58560-75-1 [(+/-)	C13H18O2	206.28721	antiinflammatory	synthetic	USP, INN, BAN, JAN		1.04	0.77	0.23	0.16	0.55
01500348	IMIPRAMINE HYDROCHLORIDE	100122-06	E10	sample	113-52-0, 50-49-7 [imipramine]	C19H25ClN2	316.8775	antidepressant	synthetic	USP, INN, BAN, JAN		0.89	1.29	0.77	0.82	0.94
01500349	INDAPAMIDE	100122-06	E11	sample	26807-65-8	C16H16ClN3O3S	365.84122	diuretic, antihypertensive	synthetic	USP, INN, BAN, JAN		1.96	-0.32	2.31	0.77	1.18
	0.11% DMSO	100122-06	E12	0.11% dmso								0.18	1.70	0.01	0.61	0.63
	0.11% JAK2inhib	100122-06	F01	0.11% jak2inhib								-5.85	-2.47	-3.85	1.43	-2.68
01500350	INDOMETHACIN	100122-06	F02	sample	53-86-1	C19H16ClNO4	357.79667	antiinflammatory, antipyretic, analgesic	synthetic	USP, INN, BAN, JAN		-2.43	-1.95	-2.44	-0.85	-1.92
01500351	INDOPROFEN	100122-06	F03	sample	31842-01-0	C17H15NO3	281.314	analgesic, antiinflammatory	synthetic	USAN, INN, BAN		1.42	0.24	2.34	3.44	1.86
01500352	INOSITOL	100122-06	F04	sample	87-89-8	C6H12O6	180.15894	growth factor	lipotropic polyol widely distributed in plants and animals	NF-XII		0.19	-0.44	0.39	0.00	0.03
01500353	IODOQUINOL	100122-06	F05	sample	83-73-8	C9H5I2NO	396.9551	antiamebic	synthetic	USP, INN, BAN		-0.69	1.17	4.21	0.15	1.21
01500354	IPRATROPIUM BROMIDE	100122-06	F06	sample	66985-17-9, 22254-24-6	C20H30BrNO3	412.371	bronchodilator, antiarrhythmic	synthetic	USAN, INN, BAN, JAN		-0.09	4.67	-0.61	0.21	1.04
01500355	ISONIAZID	100122-06	F07	sample	54-85-3	C6H7N3O	137.14219	antibacterial, tuberculostatic	synthetic	USP, INN, BAN, JAN		3.19	0.29	-0.36	-0.07	0.76
01500356	ISOPROPAMIDE IODIDE	100122-06	F08	sample	71-81-8, 7492-32-2	C23H33IN2O	480.43666	anticholinergic	synthetic	USP, INN, BAN, JAN		-0.22	1.85	2.15	-0.05	0.93
01500357	ISOPROTERENOL HYDROCHLORIDE	100122-06	F09	sample	51-30-9, 7683-59-2	C11H18ClNO3	247.72401	bronchodilator	synthetic	USP, INN, BAN, JAN		0.30	0.63	-0.09	-0.14	0.18
01500358	ISOSORBIDE DINITRATE	100122-06	F10	sample	87-33-2	C6H8N2O8	236.13926	antianginal	semisynthetic	USP, INN, BAN, JAN		1.59	1.33	2.85	1.75	1.88
01500359	ISOXSUPRINE HYDROCHLORIDE	100122-06	F11	sample	579-56-6, 395-28-8	C18H24ClNO3	337.84988	vasodilator	synthetic	USP, INN, BAN, JAN		2.33	1.25	2.47	2.12	2.04
	0.04% DMSO	100122-06	F12	0.04% dmso								1.07	-1.12	1.25	0.84	0.51
	0.04% JAK2inhib	100122-06	G01	0.04% jak2inhib								-4.74	-6.15	-3.45	-2.67	-4.25
01500360	KANAMYCIN A SULFATE	100122-06	G02	sample	25389-94-0, 133-92-6	C18H38N4O15S	582.58536	antibacterial	Streptomyces kanamyceticus	USP, INN, BAN, JAN		-0.01	-3.11	-2.16	-1.38	-1.66
01500362	KETOCONAZOLE	100122-06	G03	sample	65277-42-1	C26H28Cl2N4O4	531.44346	antifungal, PXR/SRC1 & CAR/SRC1 inhibitor	synthetic	USP, INN, BAN, JAN	Oncogene 26:258 (2007)	-2.23	-3.49	-3.16	-2.41	-2.82
01500363	LACTULOSE	100122-06	G04	sample	4618-18-2	C12H22O11	342.30254	laxative	synthetic	USP, INN, BAN, JAN		0.26	-0.48	-0.21	0.47	0.01
01500364	LEUCOVORIN CALCIUM	100122-06	G05	sample	1492-18-8	C20H21CaN7O7	511.51307	antianemic, antidote to folic acid antagonists	synthetic	USP, INN, BAN, JAN		-0.40	0.09	0.57	0.08	0.09
01500365	LEVONORDEFRIN	100122-06	G06	sample	829-74-3, 18829-78-2	C9H13NO3	183.20886	vasoconstrictor	synthetic	USP, INN		-0.18	0.89	0.55	0.10	0.34
01500368	LINCOMYCIN HYDROCHLORIDE	100122-06	G07	sample	7179-49-9, 859-18-7	C18H35ClN2O6S	443.00645	antibacterial	Streptomyces lincolnensis, S espinosus, S variabilis	USAN, INN, BAN		-0.13	0.70	0.38	0.39	0.34
01500379	MEDROXYPROGESTERONE ACETATE	100122-06	G08	sample	71-58-9, 520-85-4	C24H34O4	386.53618	contraceptive	semisynthetic	USP, INN, BAN, JAN		-1.23	0.00	-0.36	-0.74	-0.58
01500383	MEPENZOLATE BROMIDE	100122-06	G09	sample	76-90-4, 25990-43-6	C21H26BrNO3	420.35027	anticholinergic	synthetic	USP-XXIII, INN, BAN		1.05	0.32	0.26	-0.24	0.35
01500387	MERCAPTOPURINE	100122-06	G10	sample	6112-76-1, 50-44-2 [anhydrous]	C5H4N4S	152.17843	antineoplastic, purine antimetabolite	synthetic	USP, INN, BAN, JAN		1.57	0.92	0.22	-0.18	0.63
01500394	METHENAMINE	100122-06	G11	sample	100-97-0	C6H12N4	140.18934	antibacterial (urinary)	synthetic	USP, INN, JAN		1.71	2.36	0.87	1.18	1.53
	0.04% DMSO	100122-06	G12	0.04% dmso								1.57	-0.24	0.24	0.33	0.48
	0.04% JAK2inhib	100122-06	H01	0.04% jak2inhib								-4.33	-6.51	-3.76	-3.84	-4.61
01500395	METHICILLIN SODIUM	100122-06	H02	sample	7246-14-2, 132-92-3	C17H19N2NaO6S	402.40458	antibacterial	semisynthetic	USAN, INN, BAN, JAN		-1.89	-2.36	-1.34	-1.12	-1.68
01500400	METHOXSALEN	100122-06	H03	sample	298-81-7	C12H8O4	216.19516	antipsoriatic, pigmentation agent	amni visnaga	USP, BAN, JAN	Clin Pharmacol Therap 71:153 (2002)	-1.23	-0.70	1.79	-1.91	-0.51
01500404	METHYLERGONOVINE MALEATE	100122-06	H04	sample	57432-61-8, 7054-07-1	C24H29N3O6	455.51523	oxytocic	semisynthetic	USP, INN, BAN, JAN		-0.27	0.50	0.10	-1.05	-0.18
01500408	METHYLTHIOURACIL	100122-06	H05	sample	56-04-2	C5H6N2OS	142.18037	antithyroid agent	synthetic	USP-XXI, INN		-0.96	1.07	0.28	3.30	0.92
01500413	MICONAZOLE NITRATE	100122-06	H06	sample	22832-87-7, 22916-47-8	C18H15Cl4N3O4	479.14995	antifungal (topical)	synthetic	USP, JAN		1.01	-0.65	-1.00	1.97	0.33
01500427	NEOMYCIN SULFATE	100122-06	H07	sample	1405-10-3, 1404-04-2	C23H48N6O17S	712.73301	antibacterial	Streptomyces fradiae	USP, JAN		0.65	0.62	0.86	-0.10	0.51
01500434	NITROFURAZONE	100122-06	H08	sample	59-87-0	C6H6N4O4	198.13912	antiinfective (topical)	synthetic	USP, INN, BAN		-2.86	-0.79	-2.84	-3.01	-2.38
01500435	NITROMIDE	100122-06	H09	sample	121-81-3	C7H5N3O5	211.135	antibacterial, coccidiostat	synthetic	USAN		-0.54	-0.09	-2.39	-2.67	-1.42
01500438	NORETHINDRONE ACETATE	100122-06	H10	sample	51-98-9	C22H28O3	340.46666	Oral contraceptive (in combination with estrogen)	synthetic	USP		0.35	-1.18	-1.32	-1.76	-0.98
01500450	OXIDOPAMINE HYDROCHLORIDE	100122-06	H11	sample	1199-18-4	C8H12ClNO3	205.64274	adrenergic agonist (opthalmic)	synthetic	USAN, INN		1.53	-0.86	0.71	-0.63	0.19
	empty	100122-06	H12	sample								NA	NA	NA	NA	NA
	empty	100122-07	A01	sample								NA	NA	NA	NA	NA
01500456	OXYQUINOLINE HEMISULFATE	100122-07	A02	sample	148-24-3	C9H9NO5S	243.23978	antiinfectant	synthetic	USAN		-0.70	-2.04	0.78	-1.21	-0.79
01500465	PENICILLIN G POTASSIUM	100122-07	A03	sample	69-57-8, 61-33-6 [penicillin, G]	C16H17KN2O4S	372.49089	antibacterial	Penicillium spp	USP, BAN, JAN		-0.18	-1.70	1.17	4.06	0.84
01500467	PENICILLIN V POTASSIUM	100122-07	A04	sample	132-98-9, 87-08-1 [penicillin, V]	C16H17KN2O5S	388.49029	antibacterial	semisynthetic	USP, JAN		-1.06	-1.77	-0.76	-1.99	-1.39
01500480	PHENOLPHTHALEIN	100122-07	A05	sample	77-09-8	C20H14O4	318.33218	cathartic	synthetic	USP-XXIII		0.61	-0.30	0.17	2.04	0.63
01500483	PHENYLEPHRINE HYDROCHLORIDE	100122-07	A06	sample	61-76-7, 59-42-7	C9H14ClNO2	203.67043	mydriatic, decongestant	synthetic	USP, INN, BAN, JAN		-0.17	-0.23	-0.53	-0.55	-0.37
01500484	PHENYLPROPANOLAMINE HYDROCHLORIDE	100122-07	A07	sample	154-41-6	C9H14ClNO	187.67103	vasoconstrictor, decongestant, anorexic	synthetic	USP, INN, BAN		0.06	0.42	-0.41	0.03	0.02
01500486	PHYSOSTIGMINE SALICYLATE	100122-07	A08	sample	57-64-7, 57-47-6	C22H27N3O5	413.47759	cholinergic, anticholinesterase, miotic	Physostigma venenosum	USP, JAN	J Chem Soc 1954: 3651	-0.06	0.87	0.93	-0.18	0.39
01500487	PILOCARPINE NITRATE	100122-07	A09	sample	148-72-1, 92-13-7 [pilocarpine]	C11H17N3O5	271.27524	antiglaucoma agent, miotic	Pilocarpus spp	USP		2.48	3.64	-0.67	2.39	1.96
01500488	PINDOLOL	100122-07	A10	sample	13523-86-9	C14H20N2O2	248.3277	antihypertensive, antianginal, antiarrhythmic, antiglaucoma agent	synthetic	USP, INN, BAN, JAN		1.15	-0.40	0.06	0.38	0.30
01500489	PIPERACILLIN SODIUM	100122-07	A11	sample	59703-84-3, 66258-76-2	C23H26N5NaO7S	539.54677	antibacterial	semisynthetic	USP, INN, BAN, JAN		2.46	1.93	0.65	-1.17	0.97
	0.04% JAK2inhib	100122-07	A12	0.04% jak2inhib								-0.69	-1.89	-1.64	-2.10	-1.58
	0.04% DMSO	100122-07	B01	0.04% dmso								-0.89	-2.06	0.81	-2.54	-1.17
01500490	PIPERAZINE	100122-07	B02	sample	110-85-0, 144-29-6	C4H10N2	86.1377	anthelmintic	synthetic	USP		-1.23	-1.41	-0.57	-0.59	-0.95
01500491	PIROXICAM	100122-07	B03	sample	36322-90-4	C15H13N3O4S	331.35256	antiinflammatory	synthetic	USP, INN, BAN, JAN		-0.44	-1.19	0.18	1.29	-0.04
01500492	POLYMYXIN B SULFATE	100122-07	B04	sample	1405-20-5, 1404-26-8	C56H100N16O17S	1301.5824	antibacterial	Bacillus polymyxa	USP, INN, BAN, JAN		0.41	-0.80	-0.29	-0.65	-0.33
01500494	PRAZIQUANTEL	100122-07	B05	sample	55268-74-1	C19H24N2O2	312.41533	anthelmintic	synthetic	USP, INN, BAN, JAN		-0.29	-0.87	0.09	-0.15	-0.30
01500495	PRAZOSIN HYDROCHLORIDE	100122-07	B06	sample	19237-84-4, 19216-56-9	C19H22ClN5O4	419.87129	antihypertensive	synthetic	USP, INN, BAN, JAN		-0.51	2.10	3.01	-0.90	0.92
01500496	PREDNISOLONE	100122-07	B07	sample	50-24-8 [anhydrous], 52438-85-	C21H28O5	360.45431	glucocorticoid	semisynthetic	USP, INN, BAN, JAN		0.30	0.45	1.29	1.53	0.89
01500497	PREDNISOLONE ACETATE	100122-07	B08	sample	52-21-1	C23H30O6	402.49195	glucocorticoid	semisynthetic	USP, JAN		0.27	0.35	-0.81	-0.07	-0.06
01500499	PREDNISONE	100122-07	B09	sample	53-03-2	C21H26O5	358.43837	glucocorticoid	semisynthetic	USP, INN, BAN		1.11	0.11	0.76	-0.30	0.42
01500500	PRIMAQUINE DIPHOSPHATE	100122-07	B10	sample	63-45-6, 90-34-6 [primaquine]	C15H27N3O9P2	455.34474	antimalarial	synthetic	USP, INN, BAN		1.13	2.77	-0.39	-0.18	0.83
01500501	PRIMIDONE	100122-07	B11	sample	125-33-7	C12H14N2O2	218.25758	anticonvulsant	synthetic	USP, INN, BAN, JAN		1.05	-0.01	0.72	0.57	0.58
	0.04% JAK2inhib	100122-07	B12	0.04% jak2inhib								-0.63	-1.03	-1.10	-1.01	-0.94
	0.04% DMSO	100122-07	C01	0.04% dmso								-1.81	1.97	3.34	-1.76	0.43
01500502	PROBENECID	100122-07	C02	sample	57-66-9	C13H19NO4S	285.36468	uricosuric	synthetic	USP, INN, BAN, JAN		-0.53	0.30	0.89	-0.50	0.04
01500503	PROCAINAMIDE HYDROCHLORIDE	100122-07	C03	sample	614-39-1, 51-06-9	C13H22ClN3O	271.79279	antiarrhythmic	synthetic	USP, INN, BAN, JAN		-0.77	0.06	-0.03	-0.72	-0.36
01500504	PROCAINE HYDROCHLORIDE	100122-07	C04	sample	51-05-8, 59-46-1 [procaine]	C13H21ClN2O2	272.77752	anesthetic (local)	synthetic	USP, JAN		-0.30	-0.91	1.35	0.21	0.09
01500505	PROCHLORPERAZINE EDISYLATE	100122-07	C05	sample	1257-78-9, 84-02-6	C22H30ClN3O6S3	564.1459	antiemetic, antipsychotic, treatment of vertigo	synthetic	USP, JAN		-0.28	-0.91	-0.62	-1.18	-0.75
01500507	PROCYCLIDINE HYDROCHLORIDE	100122-07	C06	sample	1508-76-5, 77-37-2	C19H30ClNO	323.91005	anticholinergic	synthetic	USP, INN, BAN		-1.46	-0.49	0.96	-0.82	-0.45
01500509	PROMAZINE HYDROCHLORIDE	100122-07	C07	sample	53-60-1, 58-40-2 [promazine]	C17H21ClN2S	320.88732	antipsychotic	synthetic	USP, INN, BAN		-0.24	0.55	-0.38	-0.29	-0.09
01500511	PROPANTHELINE BROMIDE	100122-07	C08	sample	50-34-0, 298-50-0	C23H30BrNO3	448.40445	anticholinergic	synthetic	USP, INN, BAN, JAN		-0.48	-0.04	2.76	0.52	0.69
01500514	DEXPROPRANOLOL HYDROCHLORIDE	100122-07	C09	sample	13071-11-9	C16H22ClNO2	295.81224	antihypertensive, antianginal, antiarrhythmic	synthetic	USAN, INN, BAN		-0.11	-0.85	-1.00	0.09	-0.47
01500515	PROPYLTHIOURACIL	100122-07	C10	sample	51-52-5	C7H10N2OS	170.23455	antihyperthyroid	synthetic	USP, INN, BAN, JAN		0.70	-0.20	-0.02	-0.06	0.10
01500516	PSEUDOEPHEDRINE HYDROCHLORIDE	100122-07	C11	sample	345-78-8, 90-82-4	C10H16ClNO	201.69812	decongestant	synthetic	USP, INN, BAN		3.13	0.47	3.59	0.08	1.81
	0.11% JAK2inhib	100122-07	C12	0.11% jak2inhib								-0.59	-4.34	0.83	-2.25	-1.59
	0.11% DMSO	100122-07	D01	0.11% dmso								-1.27	-1.42	-0.50	-2.56	-1.44
01500517	PYRANTEL PAMOATE	100122-07	D02	sample	22204-24-6, 15686-83-6	C34H30N2O6S	594.692	anthelmintic	synthetic	USP, INN, BAN, JAN		0.14	-0.11	0.55	-1.47	-0.22
01500518	PYRAZINAMIDE	100122-07	D03	sample	98-96-4	C5H5N3O	123.1151	antibacterial, tuberculostatic	synthetic	USP, INN, BAN, JAN		-1.24	-0.74	0.40	-0.58	-0.54
01500519	PYRILAMINE MALEATE	100122-07	D04	sample	59-33-6, 91-84-9 [pyrilamine]	C21H27N3O5	401.46644	antihistaminic	synthetic	USP, INN, BAN		0.61	-0.71	0.29	-0.50	-0.08
01500520	PYRIMETHAMINE	100122-07	D05	sample	58-14-0	C12H13ClN4	248.71721	antimalarial	synthetic	USP, INN, BAN, JAN		0.49	-0.41	-0.73	0.13	-0.13
01500521	PYRVINIUM PAMOATE	100122-07	D06	sample	3546-41-6	C49H43N3O6	769.90556	anthelmintic	synthetic	USP, BAN, JAN		-1.96	-1.34	0.15	0.68	-0.62
01500522	QUINACRINE HYDROCHLORIDE	100122-07	D07	sample	6151-30-0, 69-05-6	C23H32Cl3N3O	472.88999	anthelmintic, antimalarial, intercalating agent	synthetic	USP-XXII, INN, BAN		2.72	1.99	1.94	2.47	2.28
01500523	QUINIDINE GLUCONATE	100122-07	D08	sample	7054-25-3, 6591-63-5	C26H36N2O9	520.58482	antiarrhythmic, antimalarial	Cinchona spp	USP		2.70	-0.42	0.17	-0.23	0.55
01500524	QUININE SULFATE	100122-07	D09	sample	6119-70-6, 804-63-7	C20H26N2O6S	422.50402	antimalarial, skeletal muscle relaxant	Cinchona spp	USP, JAN		0.94	0.01	-1.11	-0.03	-0.05
01500525	RACEPHEDRINE HYDROCHLORIDE	100122-07	D10	sample	134-71-4, 90-81-3	C10H16ClNO	201.69812	bronchodilator, decongestant	synthetic	USAN, INN, BAN		0.96	1.06	0.99	0.48	0.87
01500526	RESERPINE	100122-07	D11	sample	50-55-5	C33H40N2O9	608.69475	antihypertensive	Rauwolfia serpentina	USP, INN, BAN, JAN		0.48	-1.02	-1.07	0.15	-0.36
	0.11% JAK2inhib	100122-07	D12	0.11% jak2inhib								-0.34	-4.28	-0.33	-2.11	-1.77
	0.11% JAK2inhib	100122-07	E01	0.11% jak2inhib								-4.34	-4.99	-2.34	-2.64	-3.58
01500527	RESORCINOL	100122-07	E02	sample	108-46-3	C6H6O2	110.11352	keratolytic, antiseborheic	coal tar	USP, JAN		-0.98	-1.66	0.14	-1.13	-0.91
01500529	RIFAMPIN	100122-07	E03	sample	13292-46-1	C43H58N4O12	822.96131	antibacterial (tuberculostatic)	semisynthetic; L-5103, Ba-41166/E, NSC-113926	USP, INN, BAN, JAN		0.40	0.29	0.44	1.36	0.62
01500530	ROXARSONE	100122-07	E04	sample	121-19-7	C6H6AsNO6	263.03942	antibacterial	synthetic	USP, INN, BAN		-0.25	0.05	1.70	-0.23	0.32
01500531	SALICYL ALCOHOL	100122-07	E05	sample	90-01-7	C7H8O2	124.14061	anesthetic (local), antiinflammatory	Populus sieboldii	USAN	J Med Chem 13:957 (1970); Chem Pharm Bull 40:2191	-0.54	0.13	-0.13	-0.42	-0.24
01500532	SALICYLAMIDE	100122-07	E06	sample	65-45-2	C7H7NO2	137.13934	analgesic	synthetic	USP, INN, BAN, JAN		0.95	0.25	-0.28	1.71	0.66
01500533	SODIUM SALICYLATE	100122-07	E07	sample	54-21-7	C7H5NaO3	160.1059	keratolytic	synthetic	USP, JAN		-1.17	-0.42	-0.51	0.41	-0.42
01500536	SISOMICIN SULFATE	100122-07	E08	sample	53179-09-2, 32385-11-8	C19H39N5O11S	545.61358	antibacterial, binds to ribosomes	Micromonospora myoensis	USP, BAN, JAN		1.24	1.64	-0.28	1.36	0.99
01500538	SPECTINOMYCIN HYDROCHLORIDE	100122-07	E09	sample	22189-32-8, 21736-83-4	C14H26Cl2N2O7	405.27852	antibacterial	Streptomyces spectabilis	USP, INN, BAN, JAN		-0.10	0.37	-0.32	4.34	1.07
01500539	SPIRONOLACTONE	100122-07	E10	sample	52-01-7	C24H32O4S	416.58424	diuretic	synthetic	USP, INN, BAN, JAN		1.57	-0.73	-0.56	0.58	0.21
01500541	STREPTOMYCIN SULFATE	100122-07	E11	sample	3810-74-0, 57-92-1	C21H41N7O16S	679.66222	antibacterial (tuberculostatic)	Streptomyces griseus	USP, INN, BAN, JAN		0.75	0.35	-0.25	5.27	1.53
	0.11% DMSO	100122-07	E12	0.11% dmso								0.44	-1.38	-0.50	2.32	0.22
	0.11% JAK2inhib	100122-07	F01	0.11% jak2inhib								-3.73	-3.33	-2.21	-4.24	-3.37

												Screen Score (z-score)				
Compound ID	MolNAME	plate	position	Content	cas#	Formula	MolWt	Bioactivity	Source	Status	Reference	rep1	rep2	rep3	rep4	Average
01500543	STREPTOZOSIN	100122-07	F02	sample	18883-66-4	C8H15N3O7	265.22465	antineoplastic, alkylating agent	synthetic	USAN, INN		-1.15	-1.25	-0.03	-1.23	-0.91
01500544	SULFABENZAMIDE	100122-07	F03	sample	127-71-9	C13H12N2O3S	276.31619	antibacterial	synthetic	USP, INN, BAN		0.69	-0.15	0.02	5.87	1.61
01500545	SULFACETAMIDE	100122-07	F04	sample	144-80-9, 127-56-0	C8H10N2O3S	214.2445	antibacterial	synthetic	USP, INN, BAN		-0.30	0.76	1.28	-0.41	0.33
01500546	SULFADIAZINE	100122-07	F05	sample	68-35-9	C10H10N4O2S	250.2808	antibacterial	synthetic	USP, INN, BAN, JAN		1.58	0.59	-0.51	-0.22	0.36
01500547	SULFAMERAZINE	100122-07	F06	sample	127-79-7	C11H12N4O2S	264.30789	antibacterial	synthetic	USP-XXIII, INN, BAN		0.89	0.59	-0.24	5.24	1.62
01500548	SULFAMETHAZINE	100122-07	F07	sample	57-68-1	C12H14N4O2S	278.33498	antibacterial	synthetic	USP, INN, BAN		-1.62	0.73	-0.89	-0.59	-0.59
01500549	SULFAMETHIZOLE	100122-07	F08	sample	144-82-1	C9H10N4O2S2	270.33365	antibacterial	synthetic	USP, INN, BAN, JAN		-0.82	0.48	0.72	1.86	0.56
01500550	SULFAMETHOXAZOLE	100122-07	F09	sample	723-46-6	C10H11N3O3S	253.28147	antibacterial, antipneumocystis	synthetic	USP, INN, BAN, JAN		0.66	-0.11	2.69	1.30	1.14
01500551	SULFAPYRIDINE	100122-07	F10	sample	144-83-2	C11H11N3O2S	249.29322	antibacterial	synthetic	USP, INN, BAN		1.41	0.29	1.69	0.31	0.92
01500552	SULFASALAZINE	100122-07	F11	sample	599-79-1	C18H14N4O5S	398.40008	anticolitis and Crohn's disease	synthetic	USP, INN, BAN		1.24	0.49	-0.87	-0.29	0.14
	0.04% DMSO	100122-07	F12	0.04% dmso								0.18	-0.25	-0.31	-0.01	-0.10
	0.04% JAK2inhib	100122-07	G01	0.04% jak2inhib								-3.03	-2.72	-0.83	-3.50	-2.52
01500553	SULFATHIAZOLE	100122-07	G02	sample	72-14-0	C9H9N3O2S2	255.31898	antibacterial	synthetic	USP, INN, BAN		-0.80	-1.64	-0.56	-0.69	-0.93
01500554	SULFINPYRAZONE	100122-07	G03	sample	57-96-5	C23H20N2O3S	404.49145	uricosuric	synthetic	USP, INN, BAN, JAN		-0.65	0.29	0.88	-0.51	0.00
01500555	SULFISOXAZOLE	100122-07	G04	sample	127-69-5	C11H13N3O3S	267.30856	antibacterial	synthetic	USP, INN, BAN, JAN		-1.36	-0.96	-0.35	-0.24	-0.73
01500556	SULINDAC	100122-07	G05	sample	38194-50-2	C20H17FO3S	356.41909	antiinflammatory	synthetic	USP, INN, BAN, JAN		-1.53	-0.21	-0.76	-0.41	-0.73
01500557	TAMOXIFEN CITRATE	100122-07	G06	sample	54965-24-1, 10540-29-1	C32H37NO8	563.65359	estrogen antagonist, antineoplastic	synthetic	USP, INN, BAN, JAN		0.53	0.54	0.16	1.21	0.61
01500558	TERBUTALINE HEMISULFATE	100122-07	G07	sample	23031-32-5, 23031-25-6	C12H21NO7S	323.36767	betaadrenergic agonist, bronchodilator	synthetic	USP, INN, BAN, JAN		-0.31	4.48	0.76	-0.06	1.22
01500564	TETRACAINE HYDROCHLORIDE	100122-07	G08	sample	136-47-0, 94-24-6 [tetracaine]	C15H25ClN2O2	300.8317	anesthetic (local)	synthetic	USP, BAN, JAN		-0.73	0.94	-0.60	1.23	0.21
01500566	TETRACYCLINE HYDROCHLORIDE	100122-07	G09	sample	64-75-5, 60-54-8 [tetracycline]	C22H25ClN2O8	480.90615	antibacterial, antiamebic, antirickettsial	Streptomyces spp	USP, INN, BAN, JAN		0.28	-0.38	-0.26	1.30	0.23
01500567	TETRAHYDROZOLINE HYDROCHLORIDE	100122-07	G10	sample	522-48-5, 84-22-0	C13H17ClN2	236.74684	adrenergic agonist, nasal decongestant	synthetic	USP, INN, BAN, JAN		0.35	-1.00	-0.88	0.67	-0.21
01500570	THIABENDAZOLE	100122-07	G11	sample	148-79-8	C10H7N3S	201.25139	anthelmintic	synthetic	USP, INN, BAN, JAN		1.89	3.90	2.31	1.85	2.49
	0.04% DMSO	100122-07	G12	0.04% dmso								0.39	2.25	-0.93	0.11	0.45
	0.04% JAK2inhib	100122-07	H01	0.04% jak2inhib								-0.71	-3.94	-2.27	-4.12	-2.76
01500572	THIMEROSAL	100122-07	H02	sample	54-64-8	C9H9HgNaO2S	404.81468	antiinfective, preservative	synthetic	USP, INN, BAN, JAN		-8.47	-6.08	-6.93	-7.86	-7.34
01500573	THIOGUANINE	100122-07	H03	sample	154-42-7, 5580-03-0	C5H5N5S	167.1931	antineoplastic, purine antimetabolite	synthetic	USP, INN, BAN		-0.44	-0.56	-0.66	-0.04	-0.42
01500575	THIORIDAZINE HYDROCHLORIDE	100122-07	H04	sample	130-61-0, 50-52-2 [thioridazine]	C21H27ClN2S2	407.04374	antipsychotic	synthetic	USP, INN, BAN		-1.98	-1.90	-0.89	3.75	-0.26
01500576	THIOTHIXENE	100122-07	H05	sample	5591-45-7, 3313-26-6 [//Z//]	C23H29N3O2S2	443.63448	antipsychotic	synthetic	USP, INN, BAN, JAN		-5.29	-4.29	-3.73	-4.77	-4.52
01500578	TIMOLOL MALEATE	100122-07	H06	sample	26921-17-5, 91524-16-2	C17H28N4O7S	432.49931	betaadrenergic blocker	synthetic	USP, JAN		-0.98	3.73	-0.23	0.12	0.66
01500579	TOBRAMYCIN	100122-07	H07	sample	32986-56-4	C18H37N5O9	467.52369	antibacterial, inhibits protein synthesis	Streptomyces spp	USP, INN, BAN, JAN		-0.30	0.34	0.75	0.70	0.37
01500580	TOLAZOLINE HYDROCHLORIDE	100122-07	H08	sample	59-97-2, 59-98-3 [tolazoline]	C10H13ClN2	196.68151	adrenergic blocker	synthetic	USP, INN, BAN, JAN		0.59	-0.36	-0.13	1.19	0.32
01500581	TOLBUTAMIDE	100122-07	H09	sample	64-77-7	C12H18N2O3S	270.35286	antidiabetic	synthetic	USP, INN, BAN, JAN		1.08	1.25	0.14	0.74	0.80
01500584	TRANYLCYPROMINE SULFATE	100122-07	H10	sample	13492-01-8, 7081-36-9	C9H13NO4S	231.27226	antidepressant, MAO inhibitor	synthetic	USP-XXI, INN, BAN	Arch Pharmacol Res 10: 50 (1987)	0.20	0.19	0.65	-0.03	0.25
01500585	TRIACETIN	100122-07	H11	sample	102-76-1	C9H14O6	218.20833	antifungal (topical)	synthetic	USP, INN, BAN		0.35	0.27	0.40	0.31	0.33
	empty	100122-07	H12	sample								NA	NA	NA	NA	NA
	empty	100122-08	A01	sample								NA	NA	NA	NA	NA
01500586	TRIAMCINOLONE	100122-08	A02	sample	124-94-7	C21H27FO6	394.44414	glucocorticoid	semisynthetic	USP, INN, BAN, JAN		-1.09	-1.50	-1.06	-2.44	-1.52
01500587	TRIAMCINOLONE ACETONIDE	100122-08	A03	sample	76-25-5	C24H31FO6	434.50947	antiinflammatory	semisynthetic	USP, INN, BAN, JAN		-0.18	-0.72	0.79	1.46	0.34
01500588	TRIAMCINOLONE DIACETATE	100122-08	A04	sample	67-78-7	C25H31FO8	478.51942	antiinflammatory	semisynthetic	USP, JAN		-0.67	-1.57	-0.70	-1.38	-1.08
01500589	TRIAMTERENE	100122-08	A05	sample	396-01-0	C12H11N7	253.26837	diuretic	semisynthetic	USP, INN, BAN, JAN		-1.34	-1.00	-0.24	-0.89	-0.87
01500590	TRICHLORMETHIAZIDE	100122-08	A06	sample	133-67-5	C8H8Cl3N3O4S2	380.65766	diuretic, antihypertensive	synthetic	USP, INN, JAN		-0.08	-1.80	-0.90	-0.66	-0.86
01500591	TRIFLUOPERAZINE HYDROCHLORIDE	100122-08	A07	sample	440-17-5, 117-89-5	C21H26Cl2F3N3S	480.42667	antipsychotic	synthetic	USP, INN, BAN, JAN		0.21	-1.11	0.34	0.02	-0.14
01500592	TRIHEXYPHENIDYL HYDROCHLORIDE	100122-08	A08	sample	52-49-3	C20H32ClNO	337.93714	anticholinergic, antiparkinsonian	synthetic	USP, INN, BAN, JAN		0.21	-1.29	-1.14	0.18	-0.51
01500593	TRIMEPRAZINE TARTRATE	100122-08	A09	sample	4330-99-8, 41375-66-0	C22H28N2O6S	448.54226	antipruritic	synthetic	USP, INN, BAN, JAN		0.36	-0.75	-1.45	-0.45	-0.58
01500595	TRIMETHOPRIM	100122-08	A10	sample	738-70-5	C14H18N4O3	290.32456	antibacterial	synthetic	USP, INN, BAN, JAN		0.29	3.54	-0.24	4.47	2.02
01500596	TRIOXSALEN	100122-08	A11	sample	3902-71-4	C14H12O3	228.24994	melanizing agent, antipsoriatic	synthetic	USP, INN, JAN		0.67	-1.92	-1.07	-1.14	-0.87
	0.04% JAK2inhib	100122-08	A12	0.04% jak2inhib								-1.02	-2.28	-1.65	-2.89	-1.96
	0.04% DMSO	100122-08	B01	0.04% dmso								-1.79	-0.98	-0.28	1.23	-0.45
01500597	TRIPELENNAMINE CITRATE	100122-08	B02	sample	6138-56-3, 91-81-6	C22H29N3O7	447.49233	antihistaminic	synthetic	USP, BAN		-1.15	-0.50	-0.27	-0.40	-0.58
01500598	TRIPROLIDINE HYDROCHLORIDE	100122-08	B03	sample	6138-79-0, 550-70-9	C19H23ClN2	314.86156	antihistaminic	synthetic	USP, INN, BAN, JAN		0.26	0.06	-0.30	0.93	0.24
01500599	TROPICAMIDE	100122-08	B04	sample	1508-75-4	C17H20N2O2	284.36115	anticholinergic (opthalmic)	synthetic	USP, INN, BAN, JAN		0.18	0.64	0.67	0.50	0.50
01500600	TRYPTOPHAN	100122-08	B05	sample	73-22-3 ['L']	C11H12N2O2	204.23049	antidepressant, nutrient; LD50(rat) 1634 mg/kg ip	many plants, animal protein	USP, INN, JAN	Biochem J 29:2256 (1935); JACS 67:36 (1945); Aust J	0.04	-0.66	-0.70	-0.04	-0.34
01500601	TUAMINOHEPTANE SULFATE	100122-08	B06	sample	6411-75-2, 123-82-0	C7H19NO4S	213.29778	adrenergic agonist	synthetic	USP-XX		-0.39	3.30	0.00	-0.49	0.61
01500603	TYROTHRICIN	100122-08	B07	sample	1404-88-2	C66H85N11O15	1272.47805	topical antibacterial (topical)	Bacillus aneurinolyticus; mixture of gramicidins & tyrocidines	USP, INN, BAN		-0.27	-1.02	-1.11	0.03	-0.59
01500604	UREA	100122-08	B08	sample	57-13-6	CH4N2O	60.05583	diuretic	synthetic	USP, JAN		0.09	-0.17	0.00	-0.02	-0.03
01500605	URSODIOL	100122-08	B09	sample	128-13-2	C24H40O4	392.584	anticholelithogenic; LD50(rat) 890 mg/kg ip	bear bile	USP, INN, BAN, JAN	Hoppe Seyler's Z Physiol Chem 244:181 (1936); Drugs	-11.01	-4.11	-1.54	-0.62	-4.32
01500606	VALPROATE SODIUM	100122-08	B10	sample	1069-66-5, 99-66-1 [valproic	C8H15NaO2	166.19735	anticonvulsant	synthetic	USAN, JAN		-2.27	-0.35	-0.73	-1.14	-1.12
01500607	VANCOMYCIN HYDROCHLORIDE	100122-08	B11	sample	1404-93-9, 1404-90-6	C67H77Cl3N8O24	1484.75894	antibacterial	Streptomyces orientalis	USP, INN, BAN, JAN		0.74	-0.01	0.40	1.86	0.75
	0.04% JAK2inhib	100122-08	B12	0.04% jak2inhib								0.07	-2.46	-1.57	-3.50	-1.87
	0.04% DMSO	100122-08	C01	0.04% dmso								1.92	-0.41	-1.19	-1.96	-0.41
01500609	VIDARABINE	100122-08	C02	sample	24356-66-9, 5536-17-4	C10H13N5O4	267.24621	antiviral	synthetic	USP, INN, BAN, JAN		-1.08	-0.29	-1.01	-1.11	-0.87
01500613	WARFARIN	100122-08	C03	sample	81-81-2, 2610-86-8 [warfarin	C19H16O4	308.33697	anticoagulant, rodenticide	synthetic	USP, INN, BAN, JAN	Pharmacol Res Commun 10:445 (1978); Chest 102:312S	0.47	-0.64	-0.59	-0.64	-0.35
01500614	XYLOMETAZOLINE HYDROCHLORIDE	100122-08	C04	sample	1218-35-5, 526-36-3	C16H25ClN2	280.84405	adrenergic agonist, nasal decongestant	synthetic	USP, INN, BAN		-1.12	0.09	0.07	-0.85	-0.45
01500616	ACETARSOL	100122-08	C05	sample	97-44-9	C8H10AsNO5	275.0942	antiprotozoal; diethylamine salt as antiphilitic	synthetic	NF, INN, BAN		-0.76	1.42	0.59	0.01	0.31
01500637	MERBROMIN	100122-08	C06	sample	129-16-8	C20H8Br2HgNa2O6	750.66076	antibacterial	synthetic	NF-XII, INN, BAN		0.33	0.81	0.24	-0.04	0.34
01500642	PHENACETIN	100122-08	C07	sample	62-44-2	C10H13NO2	179.22061	analgesic, antipyretic	synthetic	USP-XX, INN, BAN		0.93	0.01	0.01	-0.80	0.04
01500644	PHENYLMERCURIC ACETATE	100122-08	C08	sample	62-38-4	C8H8HgO2	336.74176	antifungal	synthetic	USAN, NF		2.99	-1.00	0.19	0.47	0.66
01500646	SULFANILAMIDE	100122-08	C09	sample	63-74-1	C6H8N2O2S	172.20686	antibacterial	synthetic	NF-XI, INN		0.70	-0.46	-0.41	-0.62	-0.20
01500648	AZELAIC ACID	100122-08	C10	sample	123-99-9	C9H16O4	188.22547	antiacne, antiproliferative agent	rancid fats and Lycopodium spp	USAN, INN		1.21	0.14	0.32	5.59	1.81
01500643	PHENETHICILLIN POTASSIUM	100122-08	C11	sample	132-93-4, 147-55-7	C17H19KN2O5S	402.51738	antibacterial	semisynthetic	USP-XX, INN, BAN, JAN		1.46	-1.83	-1.38	1.66	-0.02
	0.11% JAK2inhib	100122-08	C12	0.11% jak2inhib								-1.81	-2.40	-2.95	-2.20	-2.34
	0.11% DMSO	100122-08	D01	0.11% dmso								-1.37	-0.78	-0.80	-2.60	-1.39
01500649	THEOBROMINE	100122-08	D02	sample	83-67-0	C7H8N4O2	180.16741	diuretic, bronchodilator, cardiotonic	Camelia, Theobroma, Cola spp	NF-XII, BAN		2.03	0.16	1.42	-1.21	0.60
01500651	STRYCHNINE	100122-08	D03	sample	57-24-9	C21H22N2O2	334.42169	central stimulant	Strychnos nux-vomica and other Strychnos spp	NF-X		-1.35	-0.56	-1.39	-0.92	-1.05
01500655	ACONITINE	100122-08	D04	sample	302-27-2	C34H47NO11	645.75379	anesthetic (gastric), antipyretic, and cardiotoxin	Aconitum spp	USP-X, BP-1934	Neuropharmacol 34: 1139 (1995); Biochem Pharmacol 55:	0.65	0.31	3.11	0.39	1.12
01500663	YOHIMBINE HYDROCHLORIDE	100122-08	D05	sample	65-19-0	C21H27ClN2O3	390.91394	alpha adrenergic blocker, mydriatic, antidepressant	Corynanthe spp	USP	J Chem Soc 1950: 1534; Alkaloids 2: 406 (1952);	-0.13	0.35	1.14	1.04	0.60
01500667	ADENOSINE PHOSPHATE	100122-08	D06	sample	61-19-8	C10H14N5O7P	347.22618	vasodilator, neuromodulator	widespread in living tissue	USAN, INN, BAN		-0.44	0.04	0.31	-0.04	-0.03
01500668	KETOTIFEN FUMARATE	100122-08	D07	sample	34580-14-8, 34580-13-7	C23H23NO5S	425.50746	antiasthmatic	synthetic	USAN, INN, BAN, JAN		-0.26	-0.25	4.27	0.64	1.10
01500670	BETAHISTINE HYDROCHLORIDE	100122-08	D08	sample	5579-84-0, 5638-76-6	C8H14Cl2N2	209.12018	vasodilator	synthetic	USAN, INN, BAN, JAN		1.52	0.83	-0.08	4.13	1.60
01500673	MOLSIDOMINE	100122-08	D09	sample	25717-80-0	C9H14N4O4	242.23633	antianginal	synthetic	USAN, INN, BAN, JAN		0.13	-0.31	-0.67	0.20	-0.16
01500674	MYCOPHENOLIC ACID	100122-08	D10	sample	24280-93-1	C17H20O6	320.34535	immune suppressant, antineoplastic, antiviral	Penicillium brevicompactum and other Penicillium spp	USAN, INN, BAN	Biochem J 26:1411 (1932); J Antibiot 22:358 (1969);	0.90	-0.79	1.36	1.78	0.81
01500675	OLEANDOMYCIN PHOSPHATE	100122-08	D11	sample	3922-90-5 (base)	C35H64NO16P	785.87123	antibacterial	Streptomyces antibioticus	NF-XIII, INN, BAN, JAN		-1.58	-0.33	-4.42	-3.43	-2.44
	0.11% JAK2inhib	100122-08	D12	0.11% jak2inhib								-1.79	-3.26	-2.96	-3.04	-2.76
	0.11% JAK2inhib	100122-08	E01	0.11% jak2inhib								-3.61	-2.94	-2.30	-2.82	-2.92
01500676	OUABAIN	100122-08	E02	sample	11018-89-6, 630-60-4	C29H44O12	584.66683	antiarrhythmic, cardiotonic, hypertensive, Na/K ATPase inhibitor	Acokanthera and Strophanthus spp	USP-XX, JAN	Ann Rev Biochem 15: 155 (1946); Helv Chim Acta 42: 1418	-0.44	0.52	-0.41	0.24	-0.02
01500677	ALBUTEROL (+/-)	100122-08	E03	sample	18559-94-9	C13H21NO3	239.31722	bronchodilator, tocolytic	synthetic	USP, INN, BAN		-1.22	0.34	-1.22	0.35	-0.44
01500680	ARECOLINE HYDROBROMIDE	100122-08	E04	sample	300-08-3, 63-75-2 [arecoline]	C8H14BrNO2	236.11028	anthelmintic (Cestodes), hypotensive, cathartic	betel nuts (Arica catechu)	NF-XIII	Ber 21: 3404 (1888); Biochem J 122: 503 (1971)	-0.87	-1.00	2.47	0.17	0.19
01500682	CAPTOPRIL	100122-08	E05	sample	62571-86-2	C9H15NO3S	217.2888	antihypertensive	synthetic	USP, INN, BAN, JAN		-1.44	-0.27	0.09	0.63	-0.25
01500684	CIMETIDINE	100122-08	E06	sample	51481-61-9	C10H16N6S	252.34322	antiulcer	synthetic	USP, INN, BAN, JAN		0.21	0.19	0.11	0.39	0.23
01500685	CLOZAPINE	100122-08	E07	sample	5786-21-0	C18H19ClN4	326.83193	antipsychotic	synthetic	USP, INN, BAN		-0.84	-0.41	-0.11	3.34	0.50
01500687	HYDRASTINE (1R, 9S)	100122-08	E08	sample	118-08-1	C21H21NO6	383.40462	antihypertensive, sedative, antibacterial	Corydalis spp	USP-IX	J Nat Prod 45:105 (1982)	1.14	0.90	0.12	-0.28	0.47
01500689	LIDOCAINE HYDROCHLORIDE	100122-08	E09	sample	6108-05-0, 73-78-9	C14H23ClN2O	270.80521	anesthetic (local), antiarrhythmic	synthetic	USP, INN, JAN		-0.04	-0.38	2.24	0.06	0.47
01500691	PHENTOLAMINE HYDROCHLORIDE	100122-08	E10	sample	73-05-2, 50-60-2	C17H20ClN3O	317.82145	antihypertensive	synthetic	USP-XX, INN, BAN		0.91	-0.92	3.33	-1.14	0.54
01500767	BUTAMBEN	100122-08	E11	sample	94-25-7	C11H15NO2	193.2477	anesthetic (local)	synthetic	USP		1.44	-0.62	-0.22	-0.54	0.01
	0.11% DMSO	100122-08	E12	0.11% dmso								1.31	-2.44	-0.48	-1.92	-0.88
	0.11% JAK2inhib	100122-08	F01	0.11% jak2inhib								-2.44	-1.86	-0.33	-2.99	-1.90
01500771	CEFACLOR	100122-08	F02	sample	70356-03-5, 53994-73-3	C15H14ClN3O4S	367.81353	antibacterial	semisynthetic	USP, INN, BAN, JAN		-0.40	0.93	-1.30	1.72	0.24
01500772	IODIPAMIDE	100122-08	F03	sample	606-17-7, 2618-26-0	C12H11I3N2O4	627.94567	radioopaque agent	synthetic; L-isomer: spectrum	USAN, INN, BAN, JAN		-0.14	1.17	2.54	-0.01	0.89
01500778	LIOTHYRONINE	100122-08	F04	sample	6893-02-3	C15H12I3NO4	650.98039	thyroid hormone blocker	semisynthetic	USP, BAN, JAN		-0.10	0.21	0.66	0.44	0.30
01500801	ALLANTOIN	100122-08	F05	sample	97-59-6	C4H6N4O3	158.11742	wound healing agent	allantoic fluid; widely distributed in plants	USP, BAN, JAN		-29.23	-23.29	-25.52	-22.66	-25.17
01500804	ALTHIAZIDE	100122-08	F06	sample	5588-16-9	C11H14ClN3O4S3	383.89693	diuretic	synthetic	USAN, INN		0.19	1.01	1.86	-0.99	0.52
01500807	ADENINE	100122-08	F07	sample	73-24-5	C5H5N5	135.1291	Vitamin B4	widespread in nature	USP, JAN	Ber 18: 79, 1928 (1885)	0.76	0.90	1.97	4.25	1.97
01500810	AMINACRINE	100122-08	F08	sample	90-45-9, 134-50-9 [aminacrine	C13H10N2	194.23805	local antiseptic	synthetic	USAN, IN, BAN		-0.11	-0.64	-0.44	-0.49	-0.42
01500812	BEKANAMYCIN SULFATE	100122-08	F09	sample	4696-76-8 (base)	C18H39N5O14S	581.60063	antibacterial	semisynthetic; Streptomyces kanamyceticus; NK-1006	USP, INN, BAN, JAN	JACS 80:2911 (1958); 85:1547 (1963)	1.12	-0.55	-0.49	-0.28	-0.05
01500813	BUDESONIDE	100122-08	F10	sample	51333-22-3 [11(gr b), 16(gr a)]	C25H34O6	430.54613	antiinflammatory	semisynthetic	USAN, INN, BAN, JAN		0.42	0.66	-1.43	-0.53	-0.22
01500822	BRUCINE	100122-08	F11	sample	4845-99-2, 357-57-3 [brucine]	C23H26N2O4	394.47467	central stimulant	Strychnos nux-vomica	NF-IX	Progress in Organic Chemistry 1: 1 (1952)	1.47	-0.66	0.35	-1.08	0.02
	0.04% DMSO	100122-08	F12	0.04% dmso								4.26	2.22	3.15	-1.39	2.06
	0.04% JAK2inhib	100122-08	G01	0.04% jak2inhib								-2.12	-0.64	-1.55	-1.81	-1.53
01500828	CANRENOIC ACID, POTASSIUM SALT	100122-08	G02	sample	2181-04-6, 4138-96-9	C22H29KO4	396.57603	aldosterone antagonist, diuretic	semisynthetic	USAN, INN, BAN, JAN		-0.89	0.78	-0.86	-1.12	-0.52
01500837	CHENODIOL	100122-08	G03	sample	474-25-9	C24H40O4	392.584	anticholithogenic, antilipemic agent	human bile	USAN, INN, BAN, JAN	Helv Chim Acta 43: 1595 (1960)	-0.51	0.67	-0.42	0.07	-0.05
01500838	CHOLECALCIFEROL	100122-08	G04	sample	67-97-0	C27H44O	384.65113	vitamin D3	fish oils	USP, INN, BAN, JAN	J Biol Chem 247: 2328 (1972)	-0.36	2.10	1.41	0.85	1.00
01500839	CINCHONIDINE	100122-08	G05	sample	485-71-2	C19H22N2O	294.39999	antimalarial	Cinchona spp	NF-IX		-0.21	0.61	0.60	0.07	0.27
01500841	CINCHONINE	100122-08	G06	sample	118-10-5	C19H22N2O	294.39999	antimalarial	Cinchona spp	NF-IX		-0.44	-3.24	-1.55	0.09	-1.28
01500844	COENZYME B12	100122-08	G07	sample	13870-90-1	C72H104CoN18O17P	1583.64908	vitamin	Vitamin B12; cyanocobalamin	USP-XXI		0.35	0.23	0.66	1.08	0.58
01500847	CHOLESTEROL	100122-08	G08	sample	57-88-5	C27H46O	386.66707	emulsifying agent	common animal sterol	USAN, NF		-0.26	0.19	-0.18	-0.34	-0.15
01500873	PIPERINE	100122-08	G09	sample	94-62-2	C17H19NO3	285.34588	analeptic, antibacterial	black pepper (Piper nigrum L.)	USP-VIII	Tet Lett 1979: 1043	0.42	1.06	-0.07	0.57	0.50
01500903	ETOPOSIDE	100122-08	G10	sample	33419-42-0	C29H32O13	588.57059	antineoplastic	semisynthetic	USP, INN, BAN, JAN		1.23	-0.72	-0.33	-1.06	-0.22
01500907	DEHYDROCHOLIC ACID	100122-08	G11	sample	81-23-2	C24H34O5	402.53558	choleretic	derivative of cholic acid	USP, INN, BAN, JAN		1.83	0.60	2.40	-0.73	1.02
	0.04% DMSO	100122-08	G12	0.04% dmso								1.48	2.85	-0.92	-0.97	0.61
	0.04% JAK2inhib	100122-08	H01	0.04% jak2inhib								-3.12	-1.04	-1.89	-2.63	-2.17
01500992	FLUMEQUINE	100122-08	H02	sample	42835-25-6	C14H12FNO3	261.25504	antibacterial	synthetic	USAN, INN, BAN		-1.21	1.17	1.01	-0.58	0.10
01500993	FLUNARIZINE HYDROCHLORIDE	100122-08	H03	sample	30484-77-6, 52468-60-7	C26H28Cl2F2N2	477.42926	vasodilator	synthetic	USAN, INN, BAN, JAN		0.14	1.14	0.40	0.09	0.44
01500994	FLUPHENAZINE HYDROCHLORIDE	100122-08	H04	sample	146-56-5	C22H28Cl2F3N3OS	510.45316	H1 antihistamine	synthetic	USP, BAN, JAN		-0.48	1.15	-0.21	-2.40	-0.48
01500995	FLUTAMIDE	100122-08	H05	sample	13311-84-7	C11H11F3N2O3	276.21712	antiandrogen, antineoplastic, Nuclear Hormone receptor	synthetic	USP, INN, BAN		-0.41	1.35	0.22	3.91	1.27
01501002	DROPERIDOL	100122-08	H06	sample	548-73-2	C22H22FN3O2	379.43794	neuroleptic	synthetic	USP, INN, BAN, JAN		-1.07	-0.77	1.57	-0.27	-0.13
01501003	FAMOTIDINE	100122-08	H07	sample	76824-35-6	C8H15N7O2S3	337.44645	H2 antihistamine	synthetic	USP, INN, BAN, JAN		-0.64	1.41	0.71	1.08	0.64
01501005	ETODOLAC	100122-08	H08	sample	41340-25-4	C17H21NO3	287.36182	antiinflammatory	synthetic	USP, INN, BAN		0.66	0.95	0.14	0.18	0.48
01501007	FENOTEROL HYDROBROMIDE	100122-08	H09	sample	13392-18-2	C17H22BrNO4	384.27319	betaadrenergic agonist	synthetic	USAN, INN, BAN, JAN		1.46	0.54	0.50	0.35	0.71

														Screen Score (z-score)		
Compound ID	MolNAME	plate	position	Content	cas#	Formula	MolWt	Bioactivity	Source	Status	Reference	rep1	rep2	rep3	rep4	Average
01501008	FENBUFEN	100122-08	H10	sample	36330-85-5	C16H14O3	254.28818	antiinflammatory	synthetic	USAN, INN, BAN, JAN		0.16	0.80	4.36	0.40	1.43
01501117	MEBEVERINE HYDROCHLORIDE	100122-08	H11	sample	2753-45-9, 3625-06-7	C25H36ClNO5	466.02237	muscle relaxant (smooth)	synthetic	USAN, INN, BAN		0.59	-0.35	-0.08	-0.65	-0.12
	empty	100122-08	H12	sample								NA	NA	NA	NA	NA
	empty	100122-09	A01	sample								NA	NA	NA	NA	NA
01501124	ACECLIDINE	100122-09	A02	sample	827-61-2	C9H15NO2	169.2254	cholinergic	synthetic	USAN, INN	Mol Pharm 21:594 (1982); Eur J Pharmacol 238:343 (1993);	-1.06	-1.39	-1.71	-1.15	-1.33
01501128	CAPSAICIN	100122-09	A03	sample	404-86-4	C18H27NO3	305.42079	analgesic (topical), depletes Substance P, neurotoxic	Capsicum spp	USP	J Chem Soc 1955: 1025; Life Sci 25: 1273 (1979); Trends	-0.51	0.87	-0.40	0.47	0.11
01501130	FAMPRIDINE	100122-09	A04	sample	504-24-5	C5H6N2	94.11697	K channel blocker; multiple sclerosis therapy	synthetic	USAN, INN	J Pharmacol Exp Ther 275: 864 (1995)	-0.08	-1.38	0.47	-0.34	-0.33
01501133	NICERGOLINE	100122-09	A05	sample	27848-84-6	C24H26BrN3O3	484.39712	vasodilator	synthetic	USAN, INN, BAN, JAN		-0.89	-0.66	-0.72	0.22	-0.51
01501152	SPIPERONE	100122-09	A06	sample	749-02-0	C23H26FN3O2	395.48097	antipsychotic	synthetic; R-5147	USAN, INN, BAN, JAN		-0.07	-1.85	0.26	0.58	-0.27
01501176	ERYTHROMYCIN ESTOLATE	100122-09	A07	sample	134-36-1, 114-07-8	C52H97NO18S	1056.41279	antibacterial	Streptomyces erythreus	USP, INN, BAN, JAN		-2.45	-2.85	-2.26	-1.05	-2.15
01501179	ESTRADIOL PROPIONATE	100122-09	A08	sample	113-38-2	C21H28O3	328.45551	estrogen	semisynthetic	NF-XIV, JAN		-0.65	-0.34	-0.69	2.13	0.11
01501182	ESTRADIOL BENZOATE	100122-09	A09	sample	50-50-0	C25H28O3	376.50011	estrogen	semisynthetic	USP-XX, INN, BAN, JAN		0.25	-1.25	-0.41	-0.60	-0.50
01501203	RETINOL	100122-09	A10	sample	68-26-8	C20H30O	286.4615	vitamin A	fish & liver oils, eggs, milk	USP, INN, BAN		2.09	-0.92	4.16	-0.14	1.30
01502013	ISOTRETINON	100122-09	A11	sample	4759-48-2	C20H28O2	300.44496	antiacne, antineoplastic	semisynthetic	USP, INN, BAN	Science 195: 487 (9177); N Eng J Med 300: 329 (1979)	0.77	2.23	0.75	-0.46	0.82
	0.04% JAK2inhib	100122-09	A12	0.04% jak2inhib								-2.73	-3.20	-1.00	-2.27	-2.30
	0.04% DMSO	100122-09	B01	0.04% dmso								-0.36	-1.10	-0.99	-0.36	-0.70
01502014	MESNA	100122-09	B02	sample	19767-45-4, 3375-50-6 [2-	C2H5NaO3S2	164.17815	mucolytic	synthetic	USAN, INN, BAN		-0.33	0.06	-0.45	0.29	-0.11
01502016	TRETINON	100122-09	B03	sample	302-79-4	C20H28O2	300.44496	keratolytic	semisynthetic	USP, INN, BAN	Science 195: 487 (9177); N Eng J Med 300: 329 (1979)	0.32	-0.30	-0.03	0.84	0.21
01502018	BRETYLIUM TOSYLATE	100122-09	B04	sample	61-75-6, 59-41-6 [bretylium]	C18H24BrNO3S	414.36488	inhibitor of norepinephrine release	synthetic	USP, INN, BAN		0.27	0.28	0.15	0.96	0.41
01502019	FOSCARNET SODIUM	100122-09	B05	sample	63585-09-1	CNa3O5P	191.95135	antiviral	synthetic	USAN, INN, BAN		-0.31	-0.75	-0.18	0.68	-0.14
01502029	CEFSULODIN SODIUM	100122-09	B06	sample	52152-93-9, 62587-73-9	C22H19N4NaO8S2	554.53653	antibacterial	semisynthetic	USAN, INN, BAN, JAN		-0.11	-0.58	-0.35	-0.70	-0.44
01502039	FOSFOMYCIN CALCIUM	100122-09	B07	sample	26472-47-9, 23112-90-5(acid)	C3H5CaO4P	176.1247	antibacterial	Streptomyces spp	USAN, INN, BAN		0.59	-0.47	0.83	0.86	0.46
01502041	CEFAMANDOLE NAFATE	100122-09	B08	sample	42540-40-9, 34444-01-4	C19H17N6NaO6S2	512.50174	antibacterial	semisynthetic	USP, BAN		-0.10	-1.13	0.52	0.15	-0.14
01502047	LIOTHYRONINE (L- isomer) SODIUM	100122-09	B09	sample	55-06-1, 6893-02-3	C15H11I3NNaO4	672.96222	thyroid hormone	synthetic; L-isomer	USP, BAN, JAN		0.58	-0.88	2.52	0.34	0.64
01502053	ALRESTATIN	100122-09	B10	sample	51411-04-2, 51876-97-2	C14H9NO4	255.23213	aldose reductase inhibitor	synthetic; AY-22284A	USAN, INN		0.82	-1.11	0.72	-0.12	0.08
01502084	PROADIFEN HYDROCHLORIDE	100122-09	B11	sample	78997-40-7	C23H32ClNO2	389.96999	cytochrome P450 inhibitor, Ca antagonist	synthetic; SKF-525A	USAN, INN		0.19	0.20	1.61	-0.58	0.36
	0.04% JAK2inhib	100122-09	B12	0.04% jak2inhib								-0.28	-3.89	-1.40	-1.82	-1.85
	0.04% DMSO	100122-09	C01	0.04% dmso								-0.08	-1.30	-1.29	-0.82	-0.87
01502106	CARBOPLATIN	100122-09	C02	sample	41575-94-4	C6H12N2O4Pt	371.26354	antineoplastic, convulsant	synthetic	USP, INN, BAN, JAN		-0.01	-1.01	-0.64	-0.17	-0.46
01502107	CISPLATIN	100122-09	C03	sample	15663-27-1	H6Cl2N2Pt	300.05722	antineoplastic, convulsant	synthetic	USP, INN, BAN, JAN		-0.81	-0.63	-0.45	-0.46	-0.58
01502109	ZIDOVUDINE [AZT]	100122-09	C04	sample	30516-87-1	C10H13N5O4	267.24621	RT transferase inhibitor, antiviral	synthetic	USP, INN, BAN, JAN		-0.17	3.57	-0.58	0.02	0.71
01502111	AZACITIDINE	100122-09	C05	sample	320-67-2	C8H12N4O5	244.20864	antineoplastic, pyrimidine antimetabolite	synthetic; U-18496; NSC-102816	USAN, INN		0.62	-0.15	0.16	0.57	0.30
01502112	CYCLOHEXIMIDE	100122-09	C06	sample	66-81-9	C15H23NO4	281.35486	protein synthesis inhibitor	synthetic	USAN, INN		0.59	0.40	4.27	-0.24	1.26
01502127	TINIDAZOLE	100122-09	C07	sample	19387-91-8	C8H13N3O4S	247.27451	antiprotozoal	synthetic	USP, INN, BAN, JAN		-0.07	-0.82	0.04	-0.22	-0.27
01502150	CARBIDOPA	100122-09	C08	sample	38821-49-7, 28860-95-9	C10H14N2O4	226.23408	decarboxylase inhibitor, antiparkinsonism	synthetic	USP, INN, BAN, JAN		1.47	-0.08	0.58	-0.41	0.39
01502196	ETHOSUXIMIDE	100122-09	C09	sample	77-67-8	C7H11NO2	141.17122	anticonvulsant	synthetic	USP, INN, BAN, JAN		2.35	-0.83	0.42	-0.20	0.44
01502197	PIPERIDOLATE HYDROCHLORIDE	100122-09	C10	sample	129-77-1, 82-98-4	C21H26ClNO2	359.89987	antispasmodic	synthetic	USP-XX, INN, BAN, JAN		-0.88	-1.95	4.18	-1.61	-0.06
01502198	ANISINDIONE	100122-09	C11	sample	117-37-3	C16H12O3	252.27224	anticoagulant	synthetic	NF-XIII, INN, BAN		-0.26	-1.32	-0.24	-0.37	-0.55
	0.11% JAK2inhib	100122-09	C12	0.11% jak2inhib								-2.77	-4.45	-1.65	-1.45	-2.58
	0.11% DMSO	100122-09	D01	0.11% dmso								-1.53	-0.06	-2.80	-1.75	-1.54
01502202	CYCLOSPORINE	100122-09	D02	sample	59865-13-3	C62H111N11O12	1202.64247	immunosuppressant	Tolypocladium inflatum	USP, INN, BAN, JAN	Helv Chim Acta 60: 1568 (1977)	1.75	0.41	0.60	-0.84	0.48
01502230	ASCORBIC ACID	100122-09	D03	sample	50-81-7	C6H8O6	176.12706	antiscorbutic, antiviral	Vitamin C	USP, INN, BAN, JAN		0.93	1.10	-0.41	1.52	0.78
01502254	MENADIONE	100122-09	D04	sample	58-27-5	C11H8O2	172.18521	prothrombogenic agent	Asplenium and Juglans spp.	USP, BAN	J Nat Prod 47: 901 (1984)	0.36	-0.31	-0.38	-0.18	-0.13
01502255	SALICIN	100122-09	D05	sample	138-52-3	C13H18O7	286.28421	analgesic, antipyretic	Salix spp	USP-IX		2.68	0.17	3.27	-0.09	1.51
01502258	MONENSIN SODIUM (monensin A is shown)	100122-09	D06	sample	22373-78-0, 17090-79-8	C37H63NaO10	690.89846	antibacterial	Streptomyces cinnamonensis	USP, INN, BAN	J Am Chem Soc 89: 5737 (1967)	0.77	0.38	-0.61	-0.20	0.09
01502260	ABAMECTIN	100122-09	D07	sample	65195-55-3	C48H72O14	873.10064	antiparisitic (avermectin A1a shown)	Streptomyces avermitilis	USP, INN, BAN		1.55	0.20	0.27	0.93	0.74
01503001	BENZOIC ACID	100122-09	D08	sample	65-85-0	C7H6O2	122.12467	antifungal	synthetic	USP, JAN		-0.13	1.28	-0.37	1.57	0.59
01503002	BENZYL BENZOATE	100122-09	D09	sample	120-51-4	C14H12O2	212.25054	scabicide	synthetic	USP, JAN		2.47	0.40	0.76	-0.77	0.72
01503004	BENZOYL PEROXIDE	100122-09	D10	sample	94-36-0	C14H10O4	242.2334	keratolytic	synthetic; NSC-675	USP		1.30	3.39	0.37	-0.25	1.20
01503007	BETAINE HYDROCHLORIDE	100122-09	D11	sample	590-46-5, 141-58-2 [replaced],	C5H12ClNO2	153.60989	antiarteriosclerotic, hypolipaemic, hepatoprotectant	widespread in fungi and plants	USP		1.57	0.28	0.99	-0.27	0.64
	0.11% JAK2inhib	100122-09	D12	0.11% jak2inhib								-3.07	-3.55	-2.89	-2.74	-3.06
	0.11% JAK2inhib	100122-09	E01	0.11% jak2inhib								-3.64	-2.93	-4.01	-1.90	-3.12
01503009	BIOTIN	100122-09	E02	sample	58-85-5	C10H16N2O3S	244.31462	vitamin B complex	Vitamin B complex	USP, INN, JAN		-0.62	-0.80	-1.67	2.40	-0.17
01503014	AKLOMIDE	100122-09	E03	sample	3011-89-0	C7H5ClN2O3	200.5825	antiprotozoal, coccidiostat	synthetic	USAN, INN, BAN	JACS 75: 4675 (1953)	0.04	0.60	-0.19	0.00	0.11
01503038	NICOTINYL ALCOHOL TARTRATE	100122-09	E04	sample	100-55-0	C10H13NO7	259.21761	vasodilator	synthetic	USAN, JAN		0.48	1.08	-0.21	1.94	0.82
01503059	FLOXURIDINE	100122-09	E05	sample	50-91-9	C9H11FN2O5	246.19682	antineoplastic, antimetabolite	synthetic	USP		0.35	0.29	-0.16	-0.50	0.00
01503065	ALTRETAMINE	100122-09	E06	sample	645-05-6	C9H18N6	210.28401	antineoplastic	synthetic	USP, INN, BAN		0.57	-0.36	-1.11	1.16	0.07
01503069	AMINOHIPPURIC ACID	100122-09	E07	sample	61-78-9, 94-16-6	C9H10N2O3	194.19165	renal function diagnosis	synthetic	USP		-0.58	-0.81	0.36	0.38	-0.16
01503070	MEFLOQUINE	100122-09	E08	sample	53230-10-7	C17H17ClF6N2O	414.78124	antimalarial	synthetic	USAN, INN, BAN		-0.06	1.76	0.32	5.41	1.86
01503073	ADIPHENINE HYDROCHLORIDE	100122-09	E09	sample	50-42-0, 64-95-9 [adiphenine]	C20H26ClNO2	347.88872	muscle relaxant (smooth)	synthetic	USAN, INN		-0.47	0.74	-0.07	-0.48	-0.07
01503076	QUINAPRIL HYDROCHLORIDE	100122-09	E10	sample	82586-55-8, 85441-61-8	C25H31ClN2O5	474.98922	antihypertensive, ACE inhibitor	synthetic	USP, INN, BAN		-8.06	-1.88	-0.42	-0.39	-2.69
01503081	AMIFOSTINE	100122-09	E11	sample	20537-88-6	C5H15N2O3PS	214.2247	radioprotectant	synthetic	USP, INN, BAN		0.39	0.14	0.69	-0.19	0.26
	0.11% DMSO	100122-09	E12	0.11% dmso								-0.60	-1.08	-0.22	-2.19	-1.02
	0.11% JAK2inhib	100122-09	F01	0.11% jak2inhib								-3.11	-0.30	-2.56	-3.19	-2.29
01503083	AMIPRILOSE	100122-09	F02	sample	56824-20-5, 60414-06-4	C14H28ClNO6	341.83536	immunomodulator, antiinflammatory	semisynthetic	USAN, INN		-0.17	2.84	-1.28	-0.07	0.33
01503086	TIAPRIDE HYDROCHLORIDE	100122-09	F03	sample	51012-32-9	C15H25ClN2O4S	364.8945	neuroleptic	synthetic	USAN, INN, BAN		-0.38	0.25	-0.88	0.12	-0.22
01503102	BACAMPICILLIN HYDROCHLORIDE	100122-09	F04	sample	37661-08-8, 50972-17-3	C21H28ClN3O7S	501.99021	antibacterial	semisynthetic	USP, INN, BAN, JAN		-0.02	0.93	-0.52	1.26	0.41
01503104	BENDROFLUMETHIAZIDE	100122-09	F05	sample	73-48-3	C15H14F3N3O4S2	421.41973	diuretic, antihypertensive	synthetic	USP, INN, BAN, JAN		0.51	0.99	-0.80	-0.45	0.06
01503106	BEPRIDIL HYDROCHLORIDE	100122-09	F06	sample	74764-40-2	C24H35ClN2O	403.01235	antiarrhythmic	synthetic; CERM-1978	USAN, INN, BAN, JAN		-0.26	0.56	-0.76	0.00	-0.12
01503107	BROMHEXINE HYDROCHLORIDE	100122-09	F07	sample	611-75-6, 3572-43-8	C14H21Br2ClN2	412.59787	expectorant	synthetic	USAN, INN, BAN, JAN		0.71	5.10	0.50	0.84	1.79
01503110	CARMUSTINE	100122-09	F08	sample	154-93-8	C5H9Cl2N3O2	214.05238	antineoplastic, alkylating agent	synthetic	USAN, INN, BAN	Toxicol Appl Pharmacol 21: 405 (1972)	2.39	0.42	0.71	3.75	1.82
01503111	CEFTRIAXONE SODIUM TRIHYDRATE	100122-09	F09	sample	104376-79-6, 73384-59-5	C18H22N8Na2O10S3	652.59524	antibacterial	semisynthetic; RO-13-9904	USP, INN, BAN, JAN		0.27	0.54	0.53	-0.39	0.24
01503117	TRIMIPRAMINE MALEATE	100122-09	F10	sample	521-78-8, 739-71-9	C24H30N2O4	410.5177	antidepressant	synthetic	USAN, JAN		0.67	-0.56	0.22	3.61	0.98
01503118	TRIFLUPROMAZINE HYDROCHLORIDE	100122-09	F11	sample	1098-60-8, 146-54-3	C18H20ClF3N2S	388.8857	antipsychotic	synthetic	USP, INN, BAN		0.59	-0.91	-0.67	2.76	0.44
	0.04% DMSO	100122-09	F12	0.04% dmso								0.33	-0.40	0.50	-0.27	0.04
	0.04% JAK2inhib	100122-09	G01	0.04% jak2inhib								-2.54	-3.09	-2.67	-1.95	-2.56
01503121	TRAZODONE HYDROCHLORIDE	100122-09	G02	sample	25332-39-2, 19794-93-5	C19H23Cl2N5O	408.33406	antidepressant	synthetic	USP, INN, BAN, JAN		-0.48	-0.35	-1.25	-0.79	-0.72
01503134	MENTHOL(-)	100122-09	G03	sample	1490-04-6	C10H20O	156.2703	analgesic (topical), antipruritic agent	Mentha piperita and other Mentha spp	USP, JAN		0.71	0.47	1.39	0.35	0.73
01503135	THONZYLAMINE HYDROCHLORIDE	100122-09	G04	sample	63-56-9, 91-85-0	C16H23ClN4O	322.84091	antihistamine	synthetic	USAN, INN, BAN		-0.31	-0.06	0.03	0.09	-0.06
01503136	THIAMPHENICOL	100122-09	G05	sample	15318-45-3	C12H15Cl2NO5S	356.22705	antibacterial	synthetic	USAN, INN, BAN, JAN		0.71	2.17	-0.24	0.41	0.76
01503142	TENOXICAM	100122-09	G06	sample	59804-37-4	C13H11N3O4S2	337.37832	antiinflammatory	synthetic	USAN, INN, BAN, JAN		-0.52	-0.14	-1.08	-0.12	-0.46
01503202	CHLOROXINE	100122-09	G07	sample	773-76-2	C9H5Cl2NO	214.0523	chelating agent	synthetic	USAN		0.17	1.27	0.18	3.16	1.20
01503203	CHLORPROTHIXENE HYDROCHLORIDE	100122-09	G08	sample	113-59-7	C18H19Cl2NS	352.32883	antipsychotic	synthetic	USP-XIII, INN, BAN, JAN	J Med Chem 36: 2219 (1993)	0.72	0.28	1.17	0.45	0.66
01503204	CINNARAZINE	100122-09	G09	sample	298-57-7	C26H28N2	368.52646	H1 antihistamine	synthetic	USAN, INN, BAN, JAN		-0.11	0.71	0.09	0.14	0.21
01503209	DANTROLENE SODIUM	100122-09	G10	sample	24868-20-0, 14663-23-1	C14H9N4NaO5	336.24143	muscle relaxant (skeletal)	synthetic	USAN, INN, BAN, JAN		-0.37	-0.75	-0.31	-1.74	-0.79
01503210	BETAMETHASONE 17,21-DIPROPIONATE	100122-09	G11	sample	5593-20-4	C28H37FO7	504.60129	glucocorticoid, antiinflammatory	semisynthetic	USP, INN, BAN, JAN		0.49	0.15	1.50	0.79	0.73
	0.04% DMSO	100122-09	G12	0.04% dmso								-0.24	-0.34	1.72	-1.48	-0.09
	0.04% JAK2inhib	100122-09	H01	0.04% jak2inhib								-2.49	-2.97	-1.98	-2.40	-2.46
01503212	DOBUTAMINE HYDROCHLORIDE	100122-09	H02	sample	49745-95-1, 34368-04-2	C18H24ClNO3	337.84988	cardiotonic	synthetic	USP, INN, BAN		-0.53	0.96	-0.32	0.11	0.05
01503214	EDOXUDINE	100122-09	H03	sample	15176-29-1	C11H16N2O5	256.26057	antiviral	synthetic; EDU; EUDR; ORF-15817; RWJ-15817	USAN, INN		0.91	-0.33	-0.29	1.21	0.38
01503215	ENOXACIN	100122-09	H04	sample	74011-58-8	C15H17FN4O3	320.32614	antibacterial	synthetic	USAN, INN, BAN, JAN		-0.33	-0.69	0.77	-0.35	-0.15
01503221	ETHISTERONE	100122-09	H05	sample	434-03-7	C21H28O2	312.45611	progestogen	synthetic; NSC-9565	NF-XIII, INN, BAN		-0.52	-0.13	-0.85	-0.20	-0.43
01503223	PARAROSANILINE PAMOATE	100122-09	H06	sample	7232-51-1, 569-61-9	C42H33N3O6	675.74781	anthelmintic, antischistosomal	synthetic	USAN, INN		-2.47	-1.64	-4.32	-4.54	-3.24
01503227	PERHEXILINE MALEATE	100122-09	H07	sample	6724-53-4, 6621-47-2	C23H39NO4	393.57158	coronary vasodilator	synthetic	USAN, INN, BAN	hepatotoxic at clinical dose (300mg)	0.01	0.63	-0.37	0.81	0.27
01503228	PAROMOMYCIN SULFATE	100122-09	H08	sample	1263-89-4, 7542-37-2	C23H47N5O18S	713.71774	antibacterial, antiamebic	Streptomyces rimosis paramomycinus	USP, INN, BAN		-0.04	0.06	0.17	0.20	0.10
01503229	METHAPYRILENE HYDROCHLORIDE	100122-09	H09	sample	135-23-9, 91-80-5	C14H20ClN3S	297.8526	H1 antihistamine	synthetic	USP-XX, INN, BAN		-0.45	1.20	0.09	-0.40	0.11
01503234	BETA-PROPIOLACTONE	100122-09	H10	sample	57-57-8	C3H4O2	72.06413	antiinfective	synthetic; NSC-21626	USAN, INN		-0.18	-0.15	0.66	0.31	0.16
01503237	HALCINONIDE	100122-09	H11	sample	3093-35-4	C24H32ClFO5	454.97104	glucocorticoid, antiinflammatory	synthetic	USP, INN, BAN, JAN		-1.29	-1.46	0.03	-1.35	-1.02
	empty	100122-09	H12	sample								NA	NA	NA	NA	NA
	empty	100122-10	A01	sample								NA	NA	NA	NA	NA
01503239	HYCANTHONE	100122-10	A02	sample	3105-97-3	C20H24N2O2S	356.49048	anthelmintic, hepatotoxic	synthetic	USAN, INN		-0.86	-1.09	-0.91	-0.20	-0.77
01503240	PYRIDOSTIGMINE BROMIDE	100122-10	A03	sample	101-26-8, 155-97-5	C9H13BrN2O2	261.12016	cholinergic	synthetic	USP, INN, BAN, JAN		-0.46	1.49	-0.78	0.43	0.17
01503242	ISOXICAM	100122-10	A04	sample	34552-84-6	C14H13N3O5S	335.34081	antiinflammatory	synthetic	USAN, INN, BAN		-1.07	-1.73	-0.54	-0.53	-0.96
01503243	LABETALOL HYDROCHLORIDE	100122-10	A05	sample	32780-64-6, 36894-69-6	C19H25ClN2O3	364.8757	adrenergic blocker	synthetic	USP, INN, BAN, JAN		-0.81	-1.06	0.54	0.60	-0.18
01503245	LEVAMISOLE HYDROCHLORIDE	100122-10	A06	sample	16595-80-5, 14769-73-4	C11H13ClN2S	240.75666	immunomodulator	synthetic	USP, INN, BAN		-0.26	-0.66	0.86	0.52	0.12
01503250	MEPHENTERMINE SULFATE	100122-10	A07	sample	1212-72-2, 6190-60-9	C11H19NO4S	261.34238	vasoconstrictor	synthetic	USP-XXIII, INN, BAN		-0.81	-0.97	-0.53	-0.94	-0.81
01503251	METARAMINOL BITARTRATE	100122-10	A08	sample	33402-03-8	C13H19NO8	317.29828	antihypotensive	synthetic	USP, INN, BAN, JAN		0.60	-0.43	0.94	0.70	0.45
01503252	METHAZOLAMIDE	100122-10	A09	sample	554-57-4	C5H8N4O3S2	236.27251	carbonic anhydrase inhibitor	synthetic	USP, INN, BAN, JAN		-0.17	-0.69	-0.06	-0.56	-0.37
01503253	METHYLBENZETHONIUM CHLORIDE	100122-10	A10	sample	1320-44-1, 25155-18-4	C28H44ClNO2	462.12138	antiinfective	synthetic	USP, INN, BAN		0.85	-0.37	-0.19	4.04	1.08
01503255	METHYLPREDNISOLONE SODIUM SUCCINATE	100122-10	A11	sample	2375-03-3	C26H33NaO8	496.53791	glucocorticoid, antiinflammatory	semisynthetic	USP, JAN		-0.08	-1.14	3.60	-1.12	0.32
	0.04% JAK2inhib	100122-10	A12	0.04% jak2inhib								-2.24	-3.03	-2.20	-3.54	-2.75
	0.04% DMSO	100122-10	B01	0.04% dmso								-1.21	-2.17	-1.65	-1.63	-1.67
01503256	AMSACRINE	100122-10	B02	sample	51264-14-3	C22H20N2O3S	392.4803	antineoplastic, immune suppressive	synthetic	USAN, INN, BAN	Pharmacother 5: 78 (1985)	-4.36	-4.39	-3.64	-3.95	-4.09
01503257	MIDODRINE HYDROCHLORIDE	100122-10	B03	sample	3092-17-9, 42794-76-3	C12H19ClN2O4	290.74923	antihypertensive, vasoconstrictor	synthetic	USAN, INN, BAN, JAN		-0.25	-0.96	0.29	1.56	0.16
01503260	NADOLOL	100122-10	B04	sample	42200-33-9	C17H27NO4	309.40904	betaadrenergic blocker	synthetic	USP, INN, BAN, JAN		0.01	0.12	1.50	0.84	0.62
01503262	NALTREXONE HYDROCHLORIDE	100122-10	B05	sample	16676-29-2, 16590-41-3	C20H23NO4	341.41061	morphine antagonist	synthetic	USP		-0.61	0.22	0.26	0.84	0.18
01503263	CYCLOTHIAZIDE	100122-10	B06	sample	2259-96-3	C14H16ClN3O4S2	389.88232	diuretic	synthetic	USP XXII, INN, BAN		0.31	1.38	0.29	0.67	0.67
01503265	NICLOSAMIDE	100122-10	B07	sample	50-65-7	C13H8Cl2N2O4	327.12571	anthelmintic, teniacide	synthetic; BAY-2353	USAN, INN, BAN		0.40	0.36	3.31	0.92	1.25
01503267	NOMIFENSINE MALEATE	100122-10	B08	sample	32795-47-4, 24526-64-5	C20H22N2O4	354.40934	antidepressant, dopamine uptake inhibitor	synthetic	USAN, INN, BAN	J Clin Psychiatry 45: 12 (1984); Int Pharmacopsychiatry 17:	0.33	-0.21	0.25	-0.71	-0.08
01503269	PERGOLIDE MESYLATE	100122-10	B09	sample	66104-23-2, 66104-22-1	C20H30N2O3S2	410.6017	dopamine receptor agonist	semisynthetic	USP, INN, BAN		1.29	-0.96	-1.11	-0.40	-0.29
01503270	PRILOCAINE HYDROCHLORIDE	100122-10	B10	sample	1786-81-8, 721-50-6	C13H21ClN2O	256.77812	anesthetic (local)	synthetic	USAN, INN, BAN		0.70	4.97	0.73	-0.09	1.58
01503273	HYDROCORTISONE BUTYRATE	100122-10	B11	sample	13609-67-1	C25H36O6	432.56207	glucocorticoid, antiinflammatory	semisynthetic	USP, INN, BAN, JAN		-0.05	-1.05	0.86	-0.35	-0.15
	0.04% JAK2inhib	100122-10	B12	0.04% jak2inhib								-0.77	-3.38	-1.77	-1.57	-1.87
	0.04% DMSO	100122-10	C01	0.04% dmso								-1.27	-1.33	-1.58	-1.20	-1.34
01503276	ROXITHROMYCIN	100122-10	C02	sample	80214-83-1	C41H76N2O15	837.06727	antibacterial	semisynthetic; RU-28965, RU-965	USAN, INN, JAN		1.58	0.62	-0.55	1.20	0.71
01503278	MITOXANTHRONE HYDROCHLORIDE	100122-10	C03	sample	70476-82-3, 65271-80-9	C22H30Cl2N4O6	517.4136	antineoplastic	semisynthetic	USP, INN, BAN, JAN		-0.57	-0.53	-0.32	2.65	0.31
01503279	OXETHAZAINE	100122-10	C04	sample	126-27-2	C28H41N3O3	467.65727	anesthetic (local)	synthetic	USAN, INN, BAN, JAN		-0.56	-0.56	1.56	0.54	0.24
01503298	DIPYRONE	100122-10	C05	sample	5907-38-0, 68-89-3	C13H16N3NaO4S	333.34397	analgesic, antipyretic	synthetic	USAN, INN, BAN, JAN		-0.54	0.38	0.52	0.38	0.19

														Screen Score (z-score)		
Compound ID	MolNAME	plate	position	Content	cas#	Formula	MolWt	Bioactivity	Source	Status	Reference	rep1	rep2	rep3	rep4	Average
01503301	SULFANILATE ZINC	100122-10	C06	sample	31884-76-1	C12H12N2O6S2Zn	409.73724	antibacterial	synthetic	USAN		-0.31	0.22	0.32	1.37	0.40
01503304	URETHANE	100122-10	C07	sample	51-79-6	C3H7NO2	89.09474	antineoplastic, cytotoxic	synthetic; NSC-746	NF-XIII, INN		-0.47	0.27	0.34	0.04	0.05
01503322	THIRAM	100122-10	C08	sample	137-26-8	C6H12N2S4	240.43194	antifungal	synthetic	USAN, INN		-3.80	-5.76	-2.37	-5.84	-4.44
01503324	THIOTEPA	100122-10	C09	sample	52-24-4	C6H12N3PS	189.22044	antineoplastic, alkylating agent	synthetic	USP, INN, BAN, JAN		1.09	0.31	3.78	-0.21	1.24
01503330	TETROQUINONE	100122-10	C10	sample	319-89-1	C6H4O6	172.09518	keratolytic	synthetic	USAN, INN		1.78	-0.17	0.26	-0.61	0.31
01503339	SULFANITRAN	100122-10	C11	sample	122-16-7	C14H13N3O5S	335.34081	antibacterial	synthetic; NSC-77120	USAN, INN, BAN		-0.56	-1.07	-0.74	-0.57	-0.73
	0.11% JAK2inhib	100122-10	C12	0.11% jak2inhib								-0.64	-3.62	-2.49	-0.64	-1.85
	0.11% DMSO	100122-10	D01	0.11% dmso								-1.89	-1.49	-3.03	-0.89	-1.83
01503373	OXIBENDAZOLE	100122-10	D02	sample	20559-55-1	C12H15N3O3	249.27165	anthelmintic	synthetic	USAN, INN, BAN	Am J Vet Res 38:809 (1977); J Parasitol 66:929 (1980)	1.31	0.03	1.04	1.25	0.91
01503393	PIPOBROMAN	100122-10	D03	sample	54-91-1	C10H16Br2N2O2	356.05922	antineoplastic, alkylating agent	synthetic	USP-XXII, INN		1.20	0.27	-0.12	5.49	1.71
01503412	ETANIDAZOLE	100122-10	D04	sample	22668-01-5	C7H10N4O4	214.18215	antineoplastic, hypoxic cell radiosensitizer	synthetic	USAN, INN		0.69	2.64	-0.37	0.53	0.87
01503419	NAFRONYL OXALATE	100122-10	D05	sample	3200-06-4, 31329-57-4	C26H35NO7	473.57135	vasodilator	synthetic	USAN, INN, BAN		0.02	-0.25	0.35	2.92	0.76
01503420	QUIPAZINE MALEATE	100122-10	D06	sample	5786-68-5, 4774-24-7	C17H19N3O4	329.35868	antidepressant, oxytocic	synthetic	USAN, INN		0.76	-0.03	-0.37	5.43	1.45
01503421	RITANSERIN	100122-10	D07	sample	87051-43-2	C27H25F2N3OS	477.5806	antiserotonin	synthetic	USAN, INN, BAN		0.39	1.26	3.31	0.81	1.44
01503422	SEMUSTINE	100122-10	D08	sample	13909-09-6	C10H18ClN3O2	247.72686	antineoplastic	synthetic	USAN, INN		0.47	4.90	1.18	0.26	1.70
01503423	SPIRAMYCIN	100122-10	D09	sample	8025-81-8	C43H74N2O14	843.07423	antibacterial	Streptomyces ambofaciens	USAN, INN, BAN	J Am Chem Soc 91: 3401 (1969)	1.36	0.31	0.96	-0.05	0.65
01503429	CLOFIBRATE	100122-10	D10	sample	637-07-0	C12H15ClO3	242.70455	antihyperlipidemic	synthetic	USP, INN, BAN, JAN		1.07	0.05	-0.75	-0.51	-0.04
01503500	RESORCINOL MONOACETATE	100122-10	D11	sample	102-29-4	C8H8O3	152.15116	antiseborrheic, antipruritic	synthetic	USP		0.93	0.21	1.41	-0.48	0.52
	0.11% JAK2inhib	100122-10	D12	0.11% jak2inhib								-2.00	-4.27	0.63	-4.22	-2.47
	0.11% JAK2inhib	100122-10	E01	0.11% jak2inhib								-3.67	-3.01	-3.71	-1.88	-3.07
01503600	NIMODIPINE	100122-10	E02	sample	66085-59-4	C21H26N2O7	418.45057	vasodilator	synthetic	USP, INN, BAN		-0.68	-1.50	-3.05	-1.18	-1.60
01503603	ACYCLOVIR	100122-10	E03	sample	59277-89-3	C8H11N5O3	225.20857	antiviral	synthetic	USP, INN, BAN, JAN		-0.27	0.41	-0.71	1.48	0.23
01503604	RETINYL PALMITATE	100122-10	E04	sample	79-81-2	C36H60O2	524.8784	provitamin, antixerophthalamic	semisynthetic; Vitamin A palmitate	USP, JAN		-9.56	-0.62	-0.83	-0.54	-2.88
01503607	THALIDOMIDE	100122-10	E05	sample	50-35-1	C13H10N2O4	258.23565	hypnotic	synthetic	USP, INN, BAN		-0.50	0.35	0.29	1.01	0.29
01503609	NITRENDIPINE	100122-10	E06	sample	84845-75-0	C18H20N2O6	360.3699	antihypertensive	synthetic	USAN, INN, BAN, JAN		-0.55	-0.49	0.44	-0.36	-0.24
01503610	BENZALKONIUM CHLORIDE	100122-10	E07	sample	8001-54-5	C22H40ClN	354.0238	antiinfective (topical)	synthetic	USAN, NF, INN, BAN, JAN		0.69	-0.28	1.67	2.80	1.22
01503614	CIPROFLOXACIN	100122-10	E08	sample	85721-33-1	C17H18FN3O3	331.34971	antibacterial, fungicide	synthetic	USP, INN, BAN		2.61	1.73	0.11	0.22	1.17
01503678	CELECOXIB	100122-10	E09	sample	169590-42-5	C17H14F3N3O2S	381.37923	antiarthritic, cyclooxygenase2 inhibitor	synthetic; SC-58635	USAN, INN, BAN		0.17	-0.12	0.62	-0.57	0.03
01503679	AZITHROMYCIN	100122-10	E10	sample	83905-01-5, 117772-70-0	C38H72N2O12	749.00374	antibacterial	semisynthetic	USP, INN, BAN		-6.05	0.15	0.39	0.66	-1.21
01503705	ANETHOLE	100122-10	E11	sample	4180-23-8	C10H12O	148.20654	expectorant, gastric stimulant, insecticide	anise, fennel and other plant oils	USAN, NF		-5.60	-1.20	-0.80	-0.13	-1.93
	0.11% DMSO	100122-10	E12	0.11% dmso								-0.60	-1.67	-0.18	-1.23	-0.92
	0.11% JAK2inhib	100122-10	F01	0.11% jak2inhib								-3.30	-3.72	-3.29	-3.59	-3.48
01503708	TERFENADINE	100122-10	F02	sample	50679-08-8	C32H41NO2	471.68907	H1 antihistamine, nonsedating	synthetic	USP XXIII, INN, BAN, JAN		-5.66	-2.37	-2.45	3.21	-1.82
01503710	CLOPIDOGREL SULFATE	100122-10	F03	sample	113665-84-2	C16H18ClNO6S2	419.90596	platelet aggregation inhibitor	synthetic	USP, INN, BAN		-3.36	0.38	-1.23	-0.66	-1.22
01503712	LORATADINE	100122-10	F04	sample	79794-75-5	C22H23ClN2O2	382.89381	H1 antihistamine	synthetic	USP, INN, BAN		-0.17	0.39	-0.25	0.60	0.14
01503720	SELAMECTIN	100122-10	F05	sample	165108-07-6	C43H63NO11	769.98166	antiparasitic, antimite	semisynthetic	USAN, INN		-0.25	1.15	-0.73	0.42	0.14
01503801	NAPROXOL	100122-10	F06	sample	26159-36-4	C14H16O2	216.28242	antinflammatory, analgesic, antipyretic	synthetic	USAN, INN		-0.12	0.71	1.07	0.22	0.47
01503804	COLFORSIN	100122-10	F07	sample	66575-29-9	C22H34O7	410.51208	adenylate cyclase activator, antiglaucoma, hypotensive,	Coleus forskohlii; HL-362; L-75-1362B	USAN, INN	J Biol Chem 258:2960 (1983); J Med Chem 26:436, 486	-1.15	-1.11	-1.34	-0.75	-1.09
01503807	ISOSORBIDE MONONITRATE	100122-10	F08	sample	16051-77-7	C6H9NO6	191.14173	antianginal	semisynthetic; BM-22145, IS-5-MN, AHR-4698	USAN, INN, BAN, JAN		0.30	-0.08	-0.36	0.03	-0.03
01503816	AMCINONIDE	100122-10	F09	sample	51022-69-6	C28H35FO7	502.58535	glucocorticoid, antiinflammatory	synthetic	USP, INN, BAN, JAN		0.20	0.66	-0.16	-0.17	0.13
01503818	BUPIVACAINE HYDROCHLORIDE	100122-10	F10	sample	14252-80-3, 2180-92-9	C18H29ClN2O	324.89763	anesthetic (local)	synthetic	USP, INN, BAN, JAN		1.04	-1.45	-0.06	-1.09	-0.39
01503903	ALBENDAZOLE	100122-10	F11	sample	54965-21-8	C12H15N3O2S	265.33625	anthelmintic	synthetic	USP		1.28	0.49	0.18	-0.59	0.34
	0.04% DMSO	100122-10	F12	0.04% dmso								-0.02	-1.37	2.92	-1.35	0.04
	0.04% JAK2inhib	100122-10	G01	0.04% jak2inhib								-2.91	-2.24	-3.23	-3.24	-2.90
01503908	PACLITAXEL	100122-10	G02	sample	33069-62-4	C47H51NO14	853.92882	antineoplastic	Taxus breviofolia	USP, INN, BAN	J Am Chem Soc 93: 2325 (1971)	-3.93	-3.92	-4.85	-4.08	-4.19
01503914	BUTACAINE	100122-10	G03	sample	149-15-5, 149-16-6 [butacaine]	C18H30N2O2	306.452	anesthetic (local)	synthetic	USP-XX, INN, BAN		-0.33	-1.25	-2.10	-1.12	-1.20
01503918	CLOBETASOL PROPIONATE	100122-10	G04	sample	25122-46-7, 25122-41-2	C25H32ClFO5	466.98219	glucocorticoid, antiinflammatory	synthetic	USP, INN, BAN, JAN		0.03	0.62	0.02	1.47	0.54
01503923	IOPANIC ACID	100122-10	G05	sample	96-83-3	C11H12I3NO2	570.93699	radioopaque agent	synthetic	USP, INN, BAN, JAN		0.41	0.20	-1.27	1.73	0.27
01503925	KETOROLAC TROMETHAMINE	100122-10	G06	sample	74103-07-4, 74103-06-3	C19H24N2O6	376.41293	antiinflammatory	synthetic	USP, INN, BAN		0.44	0.13	-0.83	-0.79	-0.26
01503926	LANSOPRAZOLE	100122-10	G07	sample	103577-45-3	C16H14F3N3O2S	369.36808	antiulcer, proton pump inhibitor	synthetic	USP, INN, BAN		0.27	-0.28	0.28	0.70	0.24
01503929	MEXILETINE HYDROCHLORIDE	100122-10	G08	sample	5370-01-4, 31828-71-4	C11H18ClNO	215.72521	antiarrhythmic	synthetic; KO-1173	USP, INN, BAN, JAN		0.85	0.10	0.12	0.00	0.27
01503931	MORANTEL CITRATE	100122-10	G09	sample	26155-31-7, 20574-50-9	C18H24N2O7S	412.46518	anthelmintic	synthetic	USAN, INN, BAN		0.52	0.31	-0.41	-0.83	-0.10
01503934	PERPHENAZINE	100122-10	G10	sample	58-39-9	C21H26ClN3OS	403.97787	antipsychotic	synthetic	USP, INN, BAN, JAN		1.42	0.04	0.80	-0.59	0.42
01503938	RIBAVIRIN	100122-10	G11	sample	36791-04-5	C8H12N4O5	244.20864	antiviral	synthetic	USP, INN, BAN		1.10	-0.34	1.33	-0.74	0.34
	0.04% DMSO	100122-10	G12	0.04% dmso								0.45	-0.38	-0.37	-1.21	-0.38
	0.04% JAK2inhib	100122-10	H01	0.04% jak2inhib								1.20	-1.88	-2.57	1.26	-0.50
01503968	TACROLIMUS	100122-10	H02	sample	109581-93-3, 104987-11-3	C44H69NO12	804.04003	immune suppressant, antifungal	Streptomyces tsukubaensis	USAN, INN, BAN, JAN		-9.14	-3.31	-3.47	-2.82	-4.68
01503985	BROMPHENIRAMINE MALEATE	100122-10	H03	sample	980-71-2, 86-22-6	C20H23BrN2O4	435.32131	H1 antihistamine	synthetic	USP, INN, BAN		-1.58	-0.42	-0.82	-0.62	-0.86
01504008	SIROLIMUS	100122-10	H04	sample	53123-88-9	C51H79NO13	914.19718	immunosuppressant, antineoplastic; rapamycin	Streptomyces hygroscopicus	USAN, INN, BAN		0.35	-0.38	-0.17	1.31	0.28
01504085	PAROXETINE HYDROCHLORIDE	100122-10	H05	sample	61869-08-7	C19H21ClFNO3	365.83552	antidepressant	synthetic	USP, INN, BAN		-6.61	-1.28	-0.79	-1.44	-2.53
01504088	ETHYLNOREPINEPHRINE HYDROCHLORIDE	100122-10	H06	sample	3198-07-0, 536-24-3	C10H16ClNO3	233.69692	bronchodilator	synthetic	USP-XXII, INN		-0.01	0.87	-0.29	0.37	0.24
01504136	ALAPROCLATE	100122-10	H07	sample	60719-82-6	C13H18ClNO2	255.74691	antidepressant	synthetic	USAN, INN		0.30	1.53	-0.48	3.63	1.24
01504142	ACETRIAZOIC ACID	100122-10	H08	sample	85-36-9, 129-63-5 [acetrizoate	C9H6I3NO3	556.86627	X-ray contrast medium	synthetic	USP	JACS 74: 4365 (1952); J Pharmacol Exp Therap 116: 394	-0.13	0.16	-0.02	0.00	0.00
01504171	VENLAFAXINE	100122-10	H09	sample	99300-78-4, 93413-69-5	C17H27NO2	277.41024	antidepressant	synthetic	USAN, INN, BAN		-0.93	0.62	1.23	-0.78	0.04
01504172	CITALOPRAM	100122-10	H10	sample	59729-33-8	C20H21FN2O	324.40157	antidepressant, 5HT reuptake inhibitor	synthetic	USAN		1.51	0.80	1.20	-0.17	0.83
01504173	FLUOXETINE	100122-10	H11	sample	54910-89-3	C17H19ClF3NO	345.79528	antidepressant	synthetic	USAN, INN, BAN		0.98	-0.98	0.14	-1.13	-0.25
	empty	100122-10	H12	sample								NA	NA	NA	NA	NA
	empty	100122-11	A01	sample								NA	NA	NA	NA	NA
01504174	BUPROPION	100122-11	A02	sample	31677-93-7, 34911-55-2	C13H19Cl2NO	276.20848	antidepressant	synthetic	USP, INN, BAN		-2.30	-1.08	-2.17	-1.20	-1.69
01504175	CEFUROXIME AXETIL	100122-11	A03	sample	64544-07-6, 55268-75-2	C20H22N4O10S	510.48314	antibacterial	synthetic	USP, BAN, JAN		0.90	-1.35	2.65	-0.36	0.46
01504179	FEXOFENADINE HYDROCHLORIDE	100122-11	A04	sample	138452-21-8	C32H40ClNO4	538.1329	nonsedating H1-antihistamine	synthetic	USAN, INN, BAN		-0.88	-2.02	-1.57	3.00	-0.37
01504183	TRIFLURIDINE	100122-11	A05	sample	70-00-8	C10H11F3N2O5	296.20477	antiviral (opthalmic)	synthetic	USP, INN		-5.02	-5.92	-5.88	-6.78	-5.90
01504188	PIRENPERONE	100122-11	A06	sample	75444-65-4	C23H24FN3O2	393.46503	5HT2 receptor antagonist	synthetic	USAN, INN, BAN	Psychopharmacol 119: 222 (1995)	-0.92	1.19	-0.79	-0.64	-0.29
01504190	AVOBENZONE	100122-11	A07	sample	70356-09-1	C20H22O3	310.39654	sunscreen	synthetic	USP, INN		0.06	0.82	-0.77	-0.65	-0.14
01504210	ATOVAQUONE	100122-11	A08	sample	95233-18-4	C22H19ClO3	366.84793	antipneumocystic, antimalarial	synthetic	USP, INN, BAN		3.49	1.57	2.34	0.78	2.04
01504214	TRIMETOZINE	100122-11	A09	sample	635-41-6	C14H19NO5	281.31123	sedative, neurosedative	synthetic	USAN, INN	J Med Pharm Chem 5: 800 (1962)	0.55	1.09	-0.32	-1.02	0.08
01504216	ZOXAZOLAMINE	100122-11	A10	sample	61-80-3	C7H5ClN2O	168.5837	muscle relaxant, antirrheumatic	synthetic	NF-XI, INN, BAN		1.97	0.69	0.43	1.04	1.03
01504226	CYSTEAMINE HYDROCHLORIDE	100122-11	A11	sample	60-23-1	C2H8ClNS	113.60976	antiurolithic, depigmentation, radiation protectant	cysteamine; CL-9148	USAN, INN, BAN		0.71	-0.40	-0.79	-0.08	-0.14
	0.04% JAK2inhib	100122-11	A12	0.04% jak2inhib								-0.84	-0.26	-1.67	-2.55	-1.33
	0.04% DMSO	100122-11	B01	0.04% dmso								-2.86	-3.54	-2.36	-2.38	-2.78
01504235	ROFECOXIB	100122-11	B02	sample	162011-90-7	C17H14O4S	314.36273	COX2 inhibitor, antiinflammatory, antiarthritic	synthetic	USAN, INN, BAN		-2.92	-3.19	-2.28	-1.52	-2.48
01504236	SIMVASTATIN	100122-11	B03	sample	79902-63-9	C25H38O5	418.57861	antihyperlipidemic, HMGCoA reductase inhibitor	synthetic	USP, INN, BAN		7.09	3.08	1.56	1.54	3.32
01504243	OXCARBAZEPINE	100122-11	B04	sample	28721-07-5	C15H12N2O2	252.27509	antipsychotic	synthetic	USAN, INN, BAN		2.74	-1.94	-0.16	-0.46	0.05
01504150	MELOXICAM SODIUM	100122-11	B05	sample	71125-38-7	C14H12N3NaO4S2	373.38724	antiinflammatory	synthetic	USAN, INN, BAN	Neuropharmacol 39: 1653 (2000)	-1.09	-0.28	0.20	-0.36	-0.38
01504257	CARVEDILOL	100122-11	B06	sample	72956-09-3	C24H26N2O4	406.48582	beta-adrenergic blocker	synthetic	USAN, INN, BAN, JAN		0.44	-0.71	-1.05	-1.69	-0.75
01504259	IRBESARTAN	100122-11	B07	sample	138402-11-6	C25H28N6O	428.54151	angiotensin 2 receptor antagonist	synthetic	USP, INN, BAN		1.70	-1.85	-0.34	-1.91	-0.60
01504260	LEVOFLOXACIN	100122-11	B08	sample	138199-71-0	C18H20FN3O4	361.3762	antibacterial	synthetic	USAN, INN, BAN, JAN		0.92	0.48	-0.29	0.78	0.47
01504269	LITHIUM CITRATE	100122-11	B09	sample	6080-58-6, 919-16-4	C6H5Li3O7	209.91955	antidepressant	synthetic	USP		0.59	-0.07	-0.61	0.14	0.01
01504272	GATIFLOXACIN	100122-11	B10	sample	160738-57-8	C19H22FN3O4	375.40329	antibacterial	synthetic; BMS-206584-01, AM-1155	USAN, INN		1.25	0.65	1.48	2.62	1.50
01504273	MIGLITOL	100122-11	B11	sample	72432-03-2	C8H17NO5	207.22839	alpha-glucosidase inhibitor, antidiabetic	synthetic; BAYm-1099	USAN, INN, BAN		-1.74	0.51	1.30	1.79	0.46
	0.04% JAK2inhib	100122-11	B12	0.04% jak2inhib								0.25	-0.11	0.11	-2.15	-0.47
	0.04% DMSO	100122-11	C01	0.04% dmso								-0.98	-2.19	-3.39	-2.34	-2.23
01504300	ORLISTAT	100122-11	C02	sample	96829-58-2	C29H53NO5	495.74946	reversible lipase inhibitor, antiobesity	synthetic	USAN, INN, BAN		-4.16	-3.71	-2.26	-1.68	-2.95
01504303	MOXIFLOXACIN HYDROCHLORIDE	100122-11	C03	sample	186826-86-8	C23H29ClFN3O4	465.95668	antibacterial	synthetic	USAN		0.39	-1.13	-0.11	-0.60	-0.36
01504401	PIOGLITAZONE HYDROCHLORIDE	100122-11	C04	sample	111025-46-8 (pioglitazone)	C19H21ClN2O3S	392.90782	antidiabetic	synthetic	USAN, INN, BAN		-0.06	0.17	0.79	-0.14	0.19
01504403	DONEPEZIL HYDROCHLORIDE	100122-11	C05	sample	142057-77-0	C24H30ClNO3	415.9646	acetylcholinesterase inhibitor (reversible), cognitive enhancer	synthetic; E-2020, BNAG	USAN, INN, BAN		-11.77	-3.00	-0.80	-1.32	-4.22
01504911	FLUVASTATIN	100122-11	C06	sample	93957-54-1	C24H26FNO4	411.47752	antihyperlipidemic, HMGCoA reductase inhibitor	synthetic; XU-62-320	USAN, INN, BAN		4.74	6.36	6.76	6.02	5.97
01505003	PIZOTYLINE MALATE	100122-11	C07	sample	5189-11-7	C23H27NO5S	429.53934	5HT antagonist, antimigraine	synthetic; PIZOTIFEN; BC-105	USAN, INN, BAN	J Cardiovas Pharmacol 8:805 (1986); Eur Neurol 38:31	-1.82	0.14	0.50	0.25	-0.23
01505012	EXEMESTANE	100122-11	C08	sample	107868-30-4	C20H24O2	296.41308	antineoplastic, aromatase inhibitor	synthetic; PNU-155971, FCE-24304	USAN, INN, BAN	J Steroid Biochem 30:391 (1988)	-0.11	0.26	0.30	-0.44	0.00
01505112	TILMICOSIN	100122-11	C09	sample	108050-54-0	C46H80N2O13	869.1561	antibacterial	semisynthetic	USP, INN, BAN		-1.76	0.84	0.23	0.45	-0.06
01505113	FLUNIXIN MEGLUMINE	100122-11	C10	sample	42461-84-7	C21H28F3N3O7	491.46841	analgesic, antiinflammatory	synthetic; SCH-14714	USP		1.85	3.19	1.05	1.57	1.92
01505115	CLORSULON	100122-11	C11	sample	60200-06-8	C8H8Cl3N3O4S2	380.65766	antiparasitic, fasciolicide	synthetic; MK-401	USP, INN, BAN		1.36	0.04	-0.23	1.28	0.61
	0.11% JAK2inhib	100122-11	C12	0.11% jak2inhib								-2.13	-0.23	-1.69	-2.16	-1.55
	0.11% DMSO	100122-11	D01	0.11% dmso								-0.09	-2.90	-2.90	-2.71	-2.15
01505116	ESTROPIPATE	100122-11	D02	sample	7280-37-7	C22H32N2O5S	436.57474	estrogen	semisynthetic	USP, BAN		-0.54	-0.05	-1.75	0.38	-0.49
01505124	CLAVULANATE LITHIUM	100122-11	D03	sample	58001-44-8(acid)	C8H10LiNO5	207.1116	beta-lactamase inhibitor	semisynthetic	USP		0.71	0.93	0.01	0.82	0.62
01505125	ALCLOMETAZONE DIPROPIONATE	100122-11	D04	sample	66734-13-2	C28H37ClO7	521.05589	antiinflammatory, glucocorticoid	semisynthetic; SCH-22219	USP, INN, JAN		-1.29	-0.98	-0.69	-0.94	-0.98
01505166	ALENDRONATE SODIUM	100122-11	D05	sample	121268-17-5	C4H19NNaO10P2	326.13413	bone resorption inhibitor, farnesyldiphosphate synthetase inhibitor,	synthesis	USAN	Nature 223:211 (1969); Cancer Res 62:2708 (2002); Mol	-1.22	-1.52	-0.27	-1.26	-1.07
01505172	ACARBOSE	100122-11	D06	sample	56180-94-0	C25H43NO18	645.61736	alpha-glucosidase & saccharase inhibitor, antidiabetes,	Actinoplanes spp	USAN, INN, BAN	Naturwissenschaften 64:535 (1977); Carbohydr Res	-1.58	-0.92	0.65	0.02	-0.46
01505178	ROPINIROLE	100122-11	D07	sample	91374-21-9	C16H24N2O	260.38248	dopamine receptor agonist, antiParkinsonian	synthetic; SK&F-101468	USAN, INN, BAN	J Med Chem 28:1533 (1985)	-1.87	0.11	0.43	1.42	0.02
01505187	QUETIAPINE	100122-11	D08	sample	111974-69-7	C21H25N3O2S	383.5163	antipsychotic: 5HT antagonist, dopamine antagonist, H1-	synthetic; ICI-204636, ZD-5077, ZM-204639	USAN, INN, BAN		-1.01	0.60	1.38	3.34	1.08
01505189	RIZATRIPTAN BENZOATE	100122-11	D09	sample	144034-80-0	C22H25N5O2	391.47685	5HT-1B/1D agonist, antimigraine	synthetic; MK-0462 (benzoate)	USAN, INN, BAN		0.54	0.82	0.37	-0.41	0.33
01505201	FAMCICLOVIR	100122-11	D10	sample	104227-87-4	C14H19N5O4	321.33863	antiviral	synthetic	USAN, INN, BAN		-1.51	1.68	1.08	0.45	0.42
01505202	AMLODIPINE BESYLATE	100122-11	D11	sample	111470-99-6	C26H31ClN2O8S	567.06257	Ca channel blocker	synthetic	USAN, INN, BAN, JAN		-6.82	-0.34	-1.41	0.27	-2.07
	0.11% JAK2inhib	100122-11	D12	0.11% jak2inhib								1.40	2.55	-3.01	-2.27	-0.33
	0.11% JAK2inhib	100122-11	E01	0.11% jak2inhib								-4.25	-4.72	-3.69	-0.06	-3.18
01505203	EZETIMIBE	100122-11	E02	sample	163222-33-1	C24H21F2NO3	409.43667	sterol absorption inhibitor	synthetic	USAN, INN, BAN		-1.18	-1.16	-1.81	-0.71	-1.22
01505205	OLMESARTAN MEDOXOMIL	100122-11	E03	sample	144689-63-4	C29H30N6O6	558.59905	Angiotensin II inhibitor prodrug, antihypertensive	synthetic	USAN, INN, BAN		-3.57	-2.28	-1.79	-1.25	-2.22
01505207	CEFTIBUTEN	100122-11	E04	sample	97519-39-6	C15H14N4O6S2	410.43003	antibacterial	semisynthetic	USAN, INN, BAN		-0.19	-0.75	2.75	-0.15	0.42
01505208	CEFDINIR	100122-11	E05	sample	91832-40-5	C14H13N5O5S2	395.41821	antibacterial	semisynthetic; CL-983, FK-482	USAN, INN, BAN		-0.53	0.18	0.14	0.22	0.00
01505210	SIBUTRAMINE HYDROCHLORIDE	100122-11	E06	sample	84485-00-7; 106650-56-0	C17H27Cl2N	316.31744	anorexic, antidepressant, uptake inhibitor (5HT, norepinephrine,	synthetic	USAN, INN, BAN		-0.87	-1.26	0.04	-0.52	-0.65
01505212	PERINDOPRIL ERBUMINE	100122-11	E07	sample	107133-36-8; 82834-16-0	C23H43N3O5	441.61626	antihypertensive, ACE inhibitor	synthetic; S9490-3, McN-A2833-109	USAN		0.06	1.05	1.98	0.70	0.95
01505213	ROSUVASTATIN CALCIUM	100122-11	E08	sample	287714-14-4, 147098-20-2(Ca	C22H27CaFN3O6S	520.61939	antihyperlipidemic	synthetic	USAN, INN, BAN		10.66	7.99	5.77	7.05	7.87
01505214	RAMIPRIL	100122-11	E09	sample	87333-19-5	C23H32N2O5	416.52189	antihypertensive, ACE inhibitor	synthetic; HOE-498	USP, INN, BAN		0.56	0.70	0.44	-0.49	0.30
01505216	ESCITALOPRAM OXALATE	100122-11	E10	sample	219861-08-2	C22H23FN2O5	414.43741	antidepressant, 5HT reuptake inhibitor	synthetic; LU26-054-0	USAN		-6.72	-0.51	1.15	-0.67	-1.69
01505222	DERACOXIB	100122-11	E11	sample	169590-41-4	C17H14F3N3O3S	397.37863	antiinflammatory, antiarthritic, COX-2 inhibitor	synthetic	USAN, INN		1.32	-0.61	-0.72	-2.04	-0.51
	0.11% DMSO	100122-11	E12	0.11% dmso								0.62	1.44	-0.33	-1.21	0.13
	0.11% JAK2inhib	100122-11	F01	0.11% jak2inhib								-4.06	-5.17	-5.12	-4.27	-4.66

												Screen Score (z-score)				
Compound ID	MolNAME	plate	position	Content	cas#	Formula	MolWt	Bioactivity	Source	Status	Reference	rep1	rep2	rep3	rep4	Average
01505230	CILOSTAZOL	100122-11	F02	sample	73963-72-1	C20H27N5O2	369.47049	phosphodiesterase inhibitor	synthetic	USAN, INN, BAN, JAN		-1.21	-2.36	-0.97	-0.46	-1.25
01505244	CITICOLINE	100122-11	F03	sample	987-78-0	C14H26N4O11P2	488.33112	cognition enhancer, phosphocholine cytidyltransferase activator	cytidine-5'-diphosphocholine	USAN, INN, JAN	J Neurochem 65: 889 (1995); Neurosci Lett 273: 163 (1999)	-0.13	-0.69	-1.08	-0.59	-0.62
01505249	APRAMYCIN	100122-11	F04	sample	37321-09-8	C21H41N5O11	539.58782	antibacterial; LD50(iv) 280mg/kg(mouse)	Streptomyces tenebrarius, Saccharomyces porispora hiltus	USAN, INN, BAN	J Org Chem 41: 2087 (1976); 43: 1430 (1978); Eur J	-0.13	1.49	-0.46	1.25	0.54
01505262	SERTRALINE HYDROCHLORIDE	100122-11	F05	sample	79559-97-0; 79617-96-2(base)	C17H18Cl3N	342.69871	antidepressant, 5HT uptake inhibitor	synthetic	USAN, INN, BAN		1.13	1.10	0.53	0.80	0.89
01505263	ALFLUZOSIN	100122-11	F06	sample	81403-80-7	C19H27N5O4	389.45814	alpha(1)-adrenergic blocker	synthetic; SL-77499	USAN, INN, BAN		-6.54	0.37	-0.27	1.58	-1.22
01505265	TELITHROMYCIN	100122-11	F07	sample	191114-48-4	C43H65N5O10	812.025	antibacterial	semisynthetic; HMR-3647	USAN, INN, BAN		-3.05	0.29	-0.21	-0.68	-0.91
01505267	OXAPROZIN	100122-11	F08	sample	21256-18-8	C18H15NO3	293.32515	antiinflammatory	synthetic; WY-21743	USP, INN, BAN, JAN		0.38	2.48	0.82	-0.05	0.91
01505296	OXFENDAZOLE	100122-11	F09	sample	53716-50-0	C15H13N3O3S	315.35316	anthelmintic	synthetic	USP, INN, BAN	Am J Vet Res 38:465 (1977)	0.29	-0.28	-0.27	-0.92	-0.30
01505299	AMITRAZ	100122-11	F10	sample	33089-61-1	C19H23N3	293.41526	scabicide	synthetic; U-36059	USP, INN, BAN	Toxicol Appl Pharmacol 73:411 (1984); Toxicol Lett 28:99	1.26	1.26	1.20	0.83	1.14
01505305	PEFLOXACINE MESYLATE	100122-11	F11	sample	149676-40-4	C18H24FN3O6S	429.47088	antibacterial, antiproliferative	synthetic	USAN, INN, BAN	J Med Chem 31:221 (1988); J Pharmacol Exp Ther 248:415	1.45	3.69	2.00	5.61	3.19
	0.04% DMSO	100122-11	F12	0.04% dmso								0.39	1.76	0.90	1.24	1.07
	0.04% JAK2inhib	100122-11	G01	0.04% jak2inhib								-4.63	-5.02	-4.09	-1.52	-3.82
01505308	CHLOROPHYLLIDE Cu COMPLEX Na SALT	100122-11	G02	sample	15611-43-5	C34H28CuN4Na2O5	682.15166	antineoplastic	water soluble derivative of chlorophyll	USP, JAN	Mutat Res 308:191 (1994)	-2.69	-1.77	-1.85	-0.88	-1.80
01505309	BIFONAZOLE	100122-11	G03	sample	60628-96-8	C22H18N2	310.40216	antifungal, calmodulin antagonist	synthetic	USAN, INN, BAN, JAN	Eur J Pharmacol 313:265 (1996)	-0.88	-1.47	-1.44	-1.26	-1.26
01505312	TYLOSIN TARTRATE	100122-11	G04	sample	1405-54-5, 1401-69-0(base)	C46H77NO17	916.12309	antibacterial	Streptomyces fradiae	USP, INN, BAN	Chem Ber 108:2481 (1975); Infection 5:183 (1977);	0.09	-0.94	-0.17	-0.82	-0.46
01505314	SARAFLOXACIN HYDROCHLORIDE	100122-11	G05	sample	91296-87-6	C20H18ClF2N3O3	421.83456	antibacterial	synthetic	USAN, INN, BAN	J Med Chem 28:1558 (1985); J Antimicrob Chemother	1.05	-0.02	-0.01	-0.15	0.22
01505319	CLOPIDOL	100122-11	G06	sample	2971-90-6	C7H7Cl2NO	192.04594	coccidiostat, antiplatelet	synthetic	USAN, INN, BAN	Clin Appl Thromb Hemost 8:169 (2002)	0.79	0.86	0.59	0.30	0.64
01505327	CHLORMADINONE ACETATE	100122-11	G07	sample	302-22-7	C23H29ClO4	404.93818	progestin, antiandrogen	semisynthetic	USAN, INN, BAN, JAN	Cancer Metastasis Rev 6:615 (1987); Endocrin J 42:505	-1.64	-3.05	-3.09	0.64	-1.78
01505330	OXICONAZOLE NITRATE	100122-11	G08	sample	64211-46-7	C18H14Cl4N4O4	492.14868	antifungal	synthetic	USAN, INN, BAN, JAN	Arzneimittelforschung 32:17 (1992)	0.06	0.20	0.36	0.70	0.33
01505332	AZAPERONE	100122-11	G09	sample	1649-18-9	C19H22FN3O	327.40509	tranquilizer, neuroleptic, alpha adrenergic blocker	synthetic; R-1929	USP, INN, BAN	J Vet Pharmacol Ther 9:164 (1986); Psychopharmacol	1.33	0.38	0.07	0.35	0.53
01505333	TRANILAST	100122-11	G10	sample	53902-12-8	C18H17NO5	327.33989	antiallergic, mast cell degranulation inhibitor, angiogenesis blocker	synthetic; MK-341	USAN, INN, JAN	Br J Pharmacol 118:915 (1996); 122:1061 (1997); Eur J	2.34	0.31	0.60	0.29	0.89
01505340	AZELASTINE HYDROCHLORIDE	100122-11	G11	sample	58581-89-8	C22H25Cl2N3O	418.37005	H1 antihistamine (nonsedating); leucotriene synthesis blocker	synthetic	USAN, INN, BAN, JAN	Int Arch Allergy Appl Immun 83:284 (1987)	1.16	-0.01	-0.38	-2.06	-0.32
	0.04% DMSO	100122-11	G12	0.04% dmso								3.58	1.54	1.11	-0.93	1.33
	0.04% JAK2inhib	100122-11	H01	0.04% jak2inhib								-4.05	-3.93	-3.88	-3.93	-3.95
01505346	KETANSERIN TARTRATE	100122-11	H02	sample	83846-83-7, 74050-98-9(base)	C22H22FN3O3	395.43734	5HT2/5HT1C seritonin antagonist	synthetic; R41468	USAN, INN, BAN	J Pharmacol Exp Therap 218:217 (1981); Drug Dev Res	0.29	-0.82	-1.14	0.55	-0.28
01505354	FIPRONIL	100122-11	H03	sample	120068-37-3	C12H4Cl2F6N4OS	437.15228	GABA Cl channel agonist, antiparasitic	synthetic; RM-1601, MB-46030	USAN, BAN		-3.48	-1.60	-0.75	-0.59	-1.61
01505356	DECOQUINATE	100122-11	H04	sample	18507-89-6	C24H35NO5	417.55025	coccidiostat	synthetic; M&B-15497, HC-1528	USP, INN, BAN		-7.38	-2.46	-0.28	-0.02	-2.53
01505360	CEFDITORIN PIVOXIL	100122-11	H05	sample	117467-28-4	C25H28N6O7S3	620.72991	antibacterial	semisynthetic	USAN, INN, JAN		-1.62	-1.28	-0.81	1.42	-0.57
01505368	VALACYCLOVIR HYDROCHLORIDE	100122-11	H06	sample	124832-27-5	C13H21ClN6O4	360.80312	antiviral	synthetic; BW-256-U-87	USAN, INN, BAN		-6.65	-0.62	0.63	0.26	-1.59
01505387	DULOXETINE HYDROCHLORIDE	100122-11	H07	sample	136434-34-9	C18H20ClNOS	333.8832	antidepressant	synthetic; LY-248686	USAN, INN, BAN		0.53	-0.49	0.32	0.04	0.10
01505390	NISOLDIPINE	100122-11	H08	sample	63675-72-9	C20H24N2O6	388.42408	vasodilator (coronary)	synthetic; bayk-5552	USAN, INN, BAN, JAN		0.93	0.63	0.59	4.89	1.76
01505391	MONTELUKAST SODIUM	100122-11	H09	sample	151767-02-1	C34H33ClNNaO3S	594.15381	leucotriene antagonist, antiasthmatic	synthetic; MK-476	USAN, INN, BAN		-6.49	0.01	1.07	0.27	-1.29
01505410	BENURESTAT	100122-11	H10	sample	38274-54-3	C9H9ClN2O3	228.63668	urease inhibitor	synthetic; EU-2826	USAN, INN		1.36	2.94	0.53	0.06	1.22
01505411	BENZOXIQUINE	100122-11	H11	sample	86-75-9	C16H11NO2	249.27157	antiinfective	synthetic; NSC-3951	USAN, INN		2.09	1.88	0.74	-0.78	0.98
	empty	100122-11	H12	sample								NA	NA	NA	NA	NA
	empty	100122-12	A01	sample								NA	NA	NA	NA	NA
01505412	BISMUTH SUBSALICYLATE	100122-12	A02	sample	14882-18-9	C7H5BiO4	362.0955	antidiarrheal, antacid, antiulcer	synthetic	USP, JAN		-0.43	-0.77	-0.50	-0.47	-0.54
01505413	BENZOYLPAS	100122-12	A03	sample	13898-58-3	C14H11NO4	257.24807	antibacterial, tuberculostatic	synthetic	USP-XX		-0.71	-0.56	-1.81	0.15	-0.73
01505414	BROMINDIONE	100122-12	A04	sample	1146-98-1	C15H9BrO2	301.14178	anticoagulant	synthetic	USAN, INN, BAN	Farmaco Ed Sci 10:710 (1955)	-0.95	-0.83	-2.14	1.86	-0.51
01505417	CAPOBENIC ACID	100122-12	A05	sample	21434-91-3	C16H23NO6	325.36481	antiarrhythmic	synthetic; C-3	USAN, INN		-0.25	0.84	-0.81	-0.48	-0.18
01505425	ACETOHEXAMIDE	100122-12	A06	sample	968-81-0	C15H20N2O4S	324.40165	antidiabetic	synthetic; 33006	USP, INN, BAN, JAN		0.02	-1.53	-1.12	-1.75	-1.10
01505426	ETHOXZOLAMIDE	100122-12	A07	sample	452-35-7	C9H10N2O3S2	258.31965	carbonic anhydrase inhibitor, antiulcer, antiglaucoma	synthetic	USP-XX		0.58	0.91	-0.34	-1.15	0.00
01505429	FLUCYTOSINE	100122-12	A08	sample	2022-85-7	C4H4FN3O	129.09438	antifungal	synthetic; RO-2-9915	USP, INN, BAN, JAN		-0.77	0.48	-0.77	2.35	0.32
01505432	FOMEPIZOLE HYDROCHLORIDE	100122-12	A09	sample	56010-888	C4H7ClN2	118.56679	alcohol dehydrogenase inhibitor	synthetic; 4-MP	USAN, INN, BAN		0.61	0.06	-0.61	-0.25	-0.05
01505433	GLIPIZIDE	100122-12	A10	sample	29094-61-9	C21H27N5O4S	445.54444	antidiabetic	synthetic; CP-28720, K-4024	USP, INN, BAN		0.66	0.90	-0.54	-0.89	0.03
01505435	GUANFACINE	100122-12	A11	sample	29110-47-2	C9H9Cl2N3O	246.09758	antihypertensive	synthetic; BS-100-141	USP, INN, BAN, JAN		2.48	0.50	-0.16	-0.65	0.54
	0.04% JAK2inhib	100122-12	A12	0.04% jak2inhib								-2.58	-1.23	-1.11	-1.11	-1.51
	0.04% DMSO	100122-12	B01	0.04% dmso								0.49	-0.86	-0.99	1.54	0.05
01505436	D-LACTITOL MONOHYDRATE	100122-12	B02	sample	81025-04-9, 585-86-4	C12H26O12	362.33382	sweetener, treatment of portoencephalopathy	semisynthetic	USAN, NF, INN, BAN	Zhonghua Yi Xue Za Zhi 55:31 (1995)	-0.38	-0.54	-1.79	0.78	-0.48
01505437	LEVOCARNITINE	100122-12	B03	sample	541-15-1	C7H15NO3	161.2025	cofactor for fatty acid metabolism, replenisher in arterial disease	semisynthetic	USP, INN, BAN, JAN		-0.11	-0.23	2.01	-0.05	0.41
01505440	LOBENDAZOLE	100122-12	B04	sample	6306-71-4	C10H11N3O2	205.21807	anthelmintic	synthetic; SK&F-24529; NSC-42044	USAN, INN		-1.13	-1.83	-2.14	-1.40	-1.63
01505444	METHYLENE BLUE	100122-12	B05	sample	7220-79-3	C16H20ClN3OS	337.8743	antimethemoglobinemic, cyanide antidote	synthetic	USP, INN, BAN		-3.22	-3.83	-4.25	-2.67	-3.49
01505445	METHYLATROPINE NITRATE	100122-12	B06	sample	52-88-0	C18H26N2O6	366.41772	anticholinergic	semisynthetic	USAN, INN, BAN, JAN		0.05	-0.18	0.05	-0.56	-0.16
01505448	NITHIAMIDE	100122-12	B07	sample	140-40-9	C5H5N3O3S	187.1779	antibacterial	synthetic; CI-5279	USAN, INN, BAN		-0.41	0.46	-0.37	-0.32	-0.16
01505449	PRALIDOXIME CHLORIDE	100122-12	B08	sample	51-15-0	C7H9ClN2O	172.61558	cholesterase agonist	synthetis; 2-PAM	USP, INN, BAN		-0.25	-0.51	-0.58	-1.02	-0.59
01505450	PREDNISOLONE HEMISUCCINATE	100122-12	B09	sample	2920-86-7	C25H32O8	460.52899	antiinflammatory, glucocorticoid	semisynthetic	USP, JAN		0.48	0.15	0.40	0.25	0.32
01505453	PYRIDOXINE	100122-12	B10	sample	65-23-6	C8H11NO3	169.18177	vitamin B6, enzyme cofactor	rice husks, wheat germ, yeast	USP, INN, BAN, JAN		1.75	1.00	0.03	-0.29	0.62
01505460	RIMANTADINE HYDROCHLORIDE	100122-12	B11	sample	1501-84-4	C12H22ClN	215.76884	antiviral	synthetic; EXP-126	USP, INN, BAN		0.87	1.38	0.45	4.80	1.87
	0.04% JAK2inhib	100122-12	B12	0.04% jak2inhib								0.15	1.01	-1.31	-0.12	-0.07
	0.04% DMSO	100122-12	C01	0.04% dmso								1.23	-1.72	-1.97	-0.43	-0.72
01505461	SULFISOXAZOLE ACETYL	100122-12	C02	sample	80-74-0	C13H15N3O4S	309.3462	antibacterial	synthetic	USP		0.22	1.06	0.50	1.47	0.81
01505463	TAURINE	100122-12	C03	sample	107-35-7	C2H7NO3S	125.14699	neuroprotectant, inhibitory neurotransmitter, GABA agonist	widespread in animal tissue	USP, INN, JAN	Am J Clin Nutrition 47:660 (1988); Amino Acids 23:381	-0.24	-0.77	0.32	-0.08	-0.19
01505464	THIAMINE	100122-12	C04	sample	67-03-8	C12H18Cl2N4OS	337.27346	vitamin B1, enzyme cofactor	rice husks, wheat germ, yeast	USP, INN, BAN, JAN		0.32	-0.88	0.38	3.78	0.90
01505465	TRICLOSAN	100122-12	C05	sample	3380-34-5	C12H7Cl3O2	289.54739	antiinfective	synthetic; CH-3565	USP, INN, BAN		-0.42	2.99	-0.32	0.74	0.75
01505466	TRIMETHADIONE	100122-12	C06	sample	127-48-0	C6H9NO3	143.14353	anticonvulsant	synthetic	USP-XXIII, INN, BAN, JAN		0.53	0.44	0.13	0.62	0.43
01505467	ZINC UNDECYLENATE	100122-12	C07	sample	557-08-4	C22H38O4Zn	431.91576	antifungal	synthetic	USP, JAN		0.62	-1.10	-0.04	3.96	0.86
01505468	UNDECYLENIC ACID	100122-12	C08	sample	112-38-9	C11H20O2	184.28085	antifungal	semisynthetic	USP, JAN		0.21	-0.61	0.09	0.49	0.04
01505470	CLINDAMYCIN PALMITATE HYDROCHLORIDE	100122-12	C09	sample	25507--04-4	C34H64Cl2N2O6S	699.86898	antibacterial, inhibits protein synthesis	semisynthetic; U-25179E	USP, JAN		-0.44	1.30	0.58	0.35	0.45
01505474	CEFONICID SODIUM	100122-12	C10	sample	71420-79-6	C18H17N6NaO8S3	564.55339	antibacterial	semisynthetic; SK&F-D-75073-Z	USAN, INN, BAN		1.22	1.03	-0.02	-0.44	0.45
01505480	IFOSFAMIDE	100122-12	C11	sample	3778-73-2	C7H15Cl2N2O2P	261.0896	antineoplastic	synthetic; MJF-9325, Z-4942, NSC-109724	USP, INN, BAN, JAN		0.84	1.06	4.99	0.45	1.84
	0.11% JAK2inhib	100122-12	C12	0.11% jak2inhib								-2.80	-3.38	-1.85	-1.97	-2.50
	0.11% DMSO	100122-12	D01	0.11% dmso								-1.37	0.03	-1.11	1.37	-0.27
01505482	NETILMICIN SULFATE	100122-12	D02	sample	56391-56-1	C21H45N5O15S2	671.7453	antibacterial	semisynthetic; SCH-20569	USP, INN, BAN, JAN		3.19	0.58	-0.60	0.59	0.94
01505483	DOXORUBICIN	100122-12	D03	sample	23214-92-8	C27H29NO11	543.53228	antineoplastic	semisynthetic	USAN, INN, BAN		-7.58	-9.64	-6.87	-3.89	-6.99
01505492	METHYSERGIDE MALEATE	100122-12	D04	sample	129-49-7	C25H31N3O6	469.54232	vasoconstrictor, antimigraine	semisynthetic	USP		-0.57	0.24	0.28	0.06	0.00
01505497	SOLIFENACIN SUCCINATE	100122-12	D05	sample	242478-37-1	C27H32N2O6	480.56589	muscarinic M3 antagonist	synthetic; YM-905, YM-67905	USAN, INN		-10.04	-0.18	3.00	-0.33	-1.89
01505498	ACEPROMAZINE MALEATE	100122-12	D06	sample	3598-37-6	C23H26N2O5S	442.53807	sedative	synthetic	USP,INN, BAN		0.14	-0.27	0.20	2.85	0.73
01505514	BIPERIDEN	100122-12	D07	sample	514-65-8	C21H29NO	311.47138	anticholinergic, antiparkinsonian	synthetic	USP, INN, BAN, JAN		-1.04	-0.19	0.94	-0.08	-0.09
01505530	DEXCHLORPHENIRAMINE MALEATE	100122-12	D08	sample	2438-32-6	C20H23ClN2O4	390.87031	antihistamine	synthetic	USP, INN, BAN, JAN		0.36	0.09	0.50	0.68	0.41
01505532	DILOXANIDE FUROATE	100122-12	D09	sample	3736-81-0	C14H11Cl2NO4	328.15407	amoebicide	synthetic	USP		-0.17	0.87	0.09	-0.31	0.12
01505537	ETIDRONATE DISODIUM	100122-12	D10	sample	7414-83-7	C2H6Na2O7P2	249.99312	bone resorption inhibitor	synthetic	USP, BAN, JAN		0.53	0.30	0.61	1.30	0.68
01505560	NATAMYCIN	100122-12	D11	sample	7681-93-8	C33H47NO13	665.74144	antibacterial	Steptomyces natalensis; CL-12625, A-5283	USP, INN, BAN, JAN		-6.17	2.01	1.15	1.45	-0.39
	0.11% JAK2inhib	100122-12	D12	0.11% jak2inhib								-2.32	-2.45	2.06	-0.97	-0.92
	0.11% JAK2inhib	100122-12	E01	0.11% jak2inhib								-3.68	-3.04	-4.02	-3.00	-3.43
01505562	NORGESTIMATE	100122-12	E02	sample	35189-28-7	C23H31NO3	369.50842	progestin	semisynthetic	USP, INN, BAN		-0.25	-0.90	-1.58	0.79	-0.48
01505576	TERAZOSIN HYDROCHLORIDE	100122-12	E03	sample	70024-40-7	C19H26ClN5O4	423.90317	antihypertensive	synthetic; Abbott-45975	USP, INN, BAN, JAN		-0.70	-1.17	-0.21	-0.03	-0.53
01505581	TIOCONAZOLE	100122-12	E04	sample	65899-73-2	C16H13Cl3N2OS	387.71781	antifungal	synthetic; UK-20349	USP, INN, BAN, JAN		-1.00	-0.83	-0.77	-0.11	-0.68
01505584	ERGOTAMINE TARTRATE	100122-12	E05	sample	379-79-3	C38H43N5O10	729.79391	analgesic, antimigraine	Claviceps purpurea	USP, INN, BAN, JAN		-2.83	-1.15	-0.84	-2.06	-1.72
01505590	ANAGRELIDE HYDROCHLORIDE	100122-12	E06	sample	58579-51-4	C10H8Cl3N3O	292.55376	antithrombotic	synthetic; BL-4162a	USAN, INN, BAN		0.58	0.73	0.36	0.36	0.51
01505599	ETOMIDATE	100122-12	E07	sample	33125-97-2	C14H16N2O2	244.29582	sedative	synthetic	USAN, INN, BAN		-0.97	-1.40	0.17	-0.99	-0.80
01505610	LAMOTRIGINE	100122-12	E08	sample	84057-84-1	C9H7Cl2N5	256.09564	anticonvulsant	synthetic; BW-430C	USAN, INN, BAN		-0.57	0.42	1.91	0.59	0.59
01505622	RALOXIFENE HYDROCHLORIDE	100122-12	E09	sample	82640-04-8	C28H28ClNO4S	510.05666	antiestrogen	synthetic-LY-156758	USAN, INN, BAN		-0.12	-0.08	-0.67	1.19	0.08
01505637	CEFPODOXIME PROXETIL	100122-12	E10	sample	87239-81-4	C21H27N5O9S2	557.60544	antibacterial	semisynthetic; U-76252, CS-807	USP, INN, BAN, JAN		-5.69	-0.31	0.10	0.94	-1.24
01505639	TADALAFIL	100122-12	E11	sample	171596-29-5	C22H19N3O4	389.41443	erectile dysfunction therapy	synthetic; IC-351	USAN, INN		-8.01	-0.39	-0.15	0.27	-2.07
	0.11% DMSO	100122-12	E12	0.11% dmso								0.17	-0.32	-0.65	-0.71	-0.38
	0.11% JAK2inhib	100122-12	F01	0.11% jak2inhib								-3.06	-3.22	-3.78	-2.16	-3.05
01505650	AMINOPENTAMIDE	100122-12	F02	sample	119793-66-7	C19H26N2O5S	394.49347	antispasmodic, antiemetic	synthetic; NND-1962	USP		-0.90	-1.22	-1.45	-0.25	-0.96
01505652	ARSANILIC ACID	100122-12	F03	sample	98-50-0	C6H8AsNO3	217.05716	antibacterial	synthetic; AS-101	USP, INN, BAN		1.12	-0.24	-0.38	0.40	0.22
01505656	PANTHENOL	100122-12	F04	sample	16485-10-2	C9H19NO4	205.25608	vitamin B5 precursor, radioprotectant	various plants	USP, INN, BAN, JAN		-0.33	1.10	0.60	0.72	0.52
01505660	PHENTERMINE	100122-12	F05	sample	122-09-8	C10H15N	149.23775	anorexic	synthetic	USAN, INN, BAN		-0.58	-1.50	-0.41	-0.24	-0.68
01505675	TRIENTINE HYDROCHLORIDE	100122-12	F06	sample	38260-01-4	C6H20Cl2N4	219.1591	chelating agent	synthetic; MK-0681	USP, INN, BAN		1.08	-0.21	3.37	-0.47	0.94
01505677	TICLOPIDINE HYDROCHLORIDE	100122-12	F07	sample	53885-35-1	C14H15Cl2NS	300.25235	PAF inhibitor	synthetic; 53-32C, 4-C-32	USAN, INN, BAN, JAN		0.11	0.07	-0.03	-0.58	-0.11
01505678	TICARCILLIN DISODIUM	100122-12	F08	sample	4697-14-7	C15H14N2Na2O6S2	428.39623	antibacterial	synthetic	USP, INN, BAN		0.56	0.62	0.74	0.01	0.48
01505679	TETRAMIZOLE HYDROCHLORIDE	100122-12	F09	sample	5086-74-8	C11H13ClN2S	240.75666	anthelmintic	synthetic; R-8299, McN-JR-8299-11	USAN, INN, BAN		-0.25	0.24	0.76	0.91	0.41
01505681	TOLTRAZURIL	100122-12	F10	sample	69004-03-1	C18H14F3N3O4S	425.38918	coccidiostat	synthetic; BAY-Vi-9142	USAN, INN, BAN		0.06	0.34	-0.91	-0.01	-0.13
01505682	TOREMIPHENE CITRATE	100122-12	F11	sample	89778-27-8	C32H36ClNO8	598.09862	antineoplastic, anti-estrogen	synthetic; FC-1157a	USAN, INN, BAN		1.06	0.62	0.26	0.47	0.60
	0.04% DMSO	100122-12	F12	0.04% dmso								0.70	0.60	0.51	0.36	0.54
	0.04% JAK2inhib	100122-12	G01	0.04% jak2inhib								-2.11	-2.50	0.54	-1.58	-1.41
01505683	ROLIPRAM	100122-12	G02	sample	61413-54-5	C16H21NO3	275.35067	tranquilizer	synthetic; ZK-62711	USAN, INN		0.23	-0.88	-1.06	-0.13	-0.46
01505684	ROLITETRACYCLINE	100122-12	G03	sample	751-97-3	C27H33N3O8	527.57936	antibacterial	semisynthetic; SQ-15659	USP-XXII, INN, BAN, JAN		1.26	0.01	1.03	0.67	0.74
01505690	PIPAMPERONE	100122-12	G04	sample	1893-33-0	C21H30FN3O2	375.49055	antipsychotic	synthetic; R-3345	USAN, INN, BAN		0.54	-0.68	-0.94	-1.37	-0.61
01505692	PANCURONIUM BROMIDE	100122-12	G05	sample	15500-66-0	C35H60Br2N2O4	732.68745	neuromuscular blocker	synthetic; ORG-NA-97	USAN, INN, BAN, JAN		-0.39	-0.30	0.02	-0.01	-0.17
01505701	FUMAZENIL	100122-12	G06	sample	78755-81-4	C15H14FN3O3	303.29553	benzodiazepine antagonist	synthetic; Ro-15-1788/000	USP, INN, BAN		0.39	-0.01	0.02	-0.19	0.05
01505703	ALTRENOGEST	100122-12	G07	sample	850-52-2	C21H26O2	310.44017	progestinantineoplastic	synthetic; RU-2267, A-35957	USAN, INN, BAN		-0.06	-0.19	-0.47	-0.14	-0.22
01505704	BISOPROLOL FUMARATE	100122-12	G08	sample	104344-23-2	C22H35NO8	441.52615	beta-blocker, antihypertensive	synthetic; CL-297939	USP, JAN		0.41	2.81	-0.32	0.13	0.76
01505705	FLUDARABINE PHOSPHATE	100122-12	G09	sample	75607-67-9	C10H13FN5O7P	365.21661	antineoplastic	synthetic;	USP, INN, BAN		0.71	3.78	0.27	0.13	1.22
01505706	MUPIROCIN	100122-12	G10	sample	12650-69-0	C26H44O9	500.63518	antibacterial, antimycoplasmal, isoleucyl-tRNA synthetase inhibitor	Pseudomonas fluorescens; BRL-4910A	USP, INN, BAN	J Antibiotics 31:330 (1978); Biochem J 176:305 (1978);	0.66	1.22	0.87	0.01	0.69
01505707	TEICOPLANIN [A(2-1) shown]	100122-12	G11	sample	61036-62-2	C88H95Cl2N9O33	1877.68485	antibacterial	Actinoplanes teichomyceticus; MDL-507	USAN, INN, BAN		1.15	1.55	0.80	-0.04	0.87
	0.04% DMSO	100122-12	G12	0.04% dmso								0.91	2.70	-0.19	0.22	0.91
	0.04% JAK2inhib	100122-12	H01	0.04% jak2inhib								-2.94	-2.46	-3.48	-4.68	-3.39
01505708	EPIRUBICIN HYDROCHLORIDE	100122-12	H02	sample	56390-09-1	C27H30ClNO11	579.99325	antineoplastic	synthetic; IMI-28	USAN, INN, BAN, JAN		-11.12	-16.09	-12.37	-5.86	-11.36
01505709	VECURONIUM BROMIDE	100122-12	H03	sample	50700-72-6	C34H57BrN2O4	637.74839	neuromuscular blocker	synthetic; ORG-NC-45	USAN, INN, BAN, JAN		-0.02	-0.57	-0.57	-0.39	-0.39
01505710	ALISKIREN HEMIFUMARATE	100122-12	H04	sample	173334-57-1	C34H57N3O10	667.84749	renin inhibitor	synthetic	USAN, INN		-5.92	-1.53	3.00	1.89	-0.64
01505711	ACAMPROSATE CALCIUM	100122-12	H05	sample	77337-73-6	C10H20CaN2O8S2	400.4875	alcohol antagonist	synthetic	USAN, INN, BAN		0.46	0.82	-0.60	-0.38	0.07
01505712	PREDNISOLONE SODIUM PHOSPHATE	100122-12	H06	sample	125-02-0	C21H27Na2O8P	484.39794	antiinflammatory, glucocorticoid	semisynthetic	USP, JAN		0.24	0.74	-0.64	0.24	0.15
01505713	PREGNENOLONE SUCCINATE	100122-12	H07	sample	4598-67-8	C25H36O5	416.56267	glucocortcoid, antiinflammatory	semisynthetic	USAN, INN, BAN		-5.05	-0.28	0.79	-0.16	-1.18
01505714	DARIFENACIN HYDROBROMIDE	100122-12	H08	sample	133099-07-7	C28H31BrN2O2	507.47547	M3 muscarinic antagonist, bladder suppressant	synthetic; UK-88525-04	USAN, INN, BAN		-8.25	-1.46	-0.50	-0.50	-2.68
01505722	DESOXYMETASONE	100122-12	H09	sample	382-67-2	C22H29FO4	376.47243	antiinflammatory	semisynthetic; HOE-304, R-2113, A-41-304	USP		1.73	1.63	0.70	1.79	1.46

												Screen Score (z-score)				
Compound ID	MolNAME	plate	position	Content	cas#	Formula	MolWt	Bioactivity	Source	Status	Reference	rep1	rep2	rep3	rep4	Average
01505723	BETAMETHASONE ACETATE	100122-12	H10	sample	987-24-6	C24H31FO6	434.50947	antiinflammatory	semisynthetic	USP, BAN, JAN		1.34	1.22	1.12	1.09	1.19
01505751	ERYTHROSINE SODIUM	100122-12	H11	sample	16423-68-0	C20H6I4Na2O5	879.86502	color additive	synthetic	USP-XXII	JACS 49:1594 (1927); Food Cosmet Toxicol 14:525 (1976)	2.54	0.22	1.95	0.48	1.29
	empty	100122-12	H12	sample								NA	NA	NA	NA	NA
	empty	100122-13	A01	sample								NA	NA	NA	NA	NA
01505724	ISOFLUPREDNONE ACETATE	100122-13	A02	sample	338-98-7	C23H29FO6	420.48238	antiinflammatory	semisynthetic; U-6013	USP		0.60	0.80	-0.82	-0.22	0.09
01505725	BETAMETHAZONE SODIUM PHOSPHATE	100122-13	A03	sample	151-73-5	C22H28FNa2O8P	516.41546	antiinflammatory, glucocorticoid	semisynthetic	USP, BAN, JAN		1.84	-1.34	-1.03	0.31	-0.05
01505727	MELENGESTROL ACETATE	100122-13	A04	sample	2919-66-6	C25H32O4	396.53139	antineoplastic, progestin	semisynthetic; BDH-1921, 5373, NSC-70968	USAN, INN, BAN		-0.38	0.46	-0.42	-0.83	-0.29
01505728	PHTHALYLSULFACETAMIDE	100122-13	A05	sample	131-69-1	C16H14N2O6S	362.36378	antibacterial	synthetic	NF-XIII, BAN	J Indian Chem Soc 26:130 (1949)	-0.47	-0.27	-0.62	-1.59	-0.74
01505765	TRICHLORFON	100122-13	A06	sample	52-68-6	C4H8Cl3O4P	257.43876	anthelmintic	synthetic; Bayer-L-1359, DETF	USAN	Biochem Pharmacol 24:717 (1975); Arch Toxicol 41:31	-0.79	-0.72	0.04	-1.14	-0.66
01505778	BEPHENIUM HYDROXYNAPTHOATE	100122-13	A07	sample	3818-50-6	C28H29NO4	443.54763	anthelmintic	synthetic	USP-XXI, INN, BAN	Experientia 30:1265 (1974)	-1.21	0.01	0.41	-0.82	-0.40
01505780	DIPERODON HYDROCHLORIDE	100122-13	A08	sample	537-12-2	C22H28ClN3O4	433.93916	analgesic, anesthetic	synthetic	USP-XXII, INN, BAN		-0.10	-1.26	-0.49	-0.14	-0.50
01505777	DIATRIZOIC ACID	100122-13	A09	sample	117-96-4	C11H9I3N2O4	613.91858	radiopaque agent	synthetic	USP, BAN, JAN	Anal Profiles Drug Subst 4:137 (1975)	-0.45	-1.20	0.72	-0.53	-0.37
01505715	PANTOTHENIC ACID(d) Na salt	100122-13	A10	sample	63409-48-3	C9H16NNaO5	241.22137	vitamin B5	rice bran, queen bee jelly	USP, INN, JAN	Biophys Res Commun 38:181 (1970)	-0.29	-0.03	3.48	0.57	0.93
01505726	DESONIDE	100122-13	A11	sample	638-94-8	C24H32O6	416.51904	antiinflammatory, glucocorticoid	semisynthetic; D-2083	USAN, INN, BAN		-0.74	-3.03	-2.82	-3.05	-2.41
	0.04% JAK2inhib	100122-13	A12	0.04% jak2inhib								-1.68	-2.18	-2.38	-1.07	-1.83
	0.04% DMSO	100122-13	B01	0.04% dmso								-0.59	-0.74	-2.58	-1.22	-1.29
01505753	GLYCOPYRROLATE	100122-13	B02	sample	596-51-0	C19H28BrNO3	398.34391	anticholinergic	synthetic; AHR-504	USAN, INN, BAN, JAN	J Pharm Pharmacol 26:352 (1974); Toxicol Appl Pharmacol	0.03	-0.32	-1.55	3.24	0.35
01505756	ITRACONAZOLE	100122-13	B03	sample	84625-61-6	C35H38Cl2N8O4	705.65031	antifungal	synthetic; R51211	USAN, INN, BAN, JAN		-0.85	-0.57	2.24	0.34	0.29
01505760	OCTISALATE	100122-13	B04	sample	118-60-5	C15H22O3	250.34079	sunscreen	synthetic	USP, INN		0.40	0.80	0.01	0.69	0.48
01505763	RIBOFLAVIN 5-PHOSPHATE SODIUM	100122-13	B05	sample	130-40-5	C17H20N4NaO9P	478.33395	vitamin, enzyme cofactor	wdiespread in plants and animals	USP, JAN	Helv Chim Acta 35:457 (1952)	1.84	-0.21	-1.01	-0.42	0.05
01505781	SELEGILINE HYDROCHLORIDE	100122-13	B06	sample	14611-52-0	C13H18ClN	223.74811	antidepressant, MAO inhibitor, antiparkinsonian	synthetic	USP, INN, BAN	J Org Chem 42:2637 (1977); Am J Psychiatry 150:321	0.01	-0.45	1.93	0.00	0.37
01505782	CEFTAZIDIME	100122-13	B07	sample	72558-82-8	C22H22N6O7S2	546.58464	antibacterial	semisynthetic	USP, INN, BAN, JAN	J Antimicrobial Chemother 12(suppl A):1 (1983); Drugs	-0.77	-0.67	0.74	0.20	-0.13
01505805	GABAPENTIN	100122-13	B08	sample	60142-96-3	C9H17NO2	171.24134	anticonvulsant	synthetic; CI-945; GOE-3450	USAN, INN, BAN		-0.72	0.12	0.84	-0.03	0.05
01505819	ELETRIPTAN HYDROBROMIDE	100122-13	B09	sample	177834-92-3, 143322-58-	C22H27BrN2O2S	463.44069	5-HT agonist, anti-migrane	synthetic; UK-116044-04	USAN, INN, BAN		-4.08	-1.71	0.52	2.39	-0.72
01505851	ARIPIPRAZOLE	100122-13	B10	sample	129722-12-9	C23H27Cl2N3O2	448.39654	antipsychotic, 5HT2A antagonist	synthetic; OPC-14597, OPC-31	USAN, INN, BAN	J Med Chem 41:658 (1998); J Clin Psychiatry 63:763	-7.58	-2.90	-0.98	-0.78	-3.06
01505906	ZILEUTON	100122-13	B11	sample	111406-87-2	C11H12N2O2S	236.29449	5-lipoxygenase inhibitor	synthetic; Abbott-64077	USP, INN, BAN		1.24	-0.76	0.53	0.71	0.43
	0.04% JAK2inhib	100122-13	B12	0.04% jak2inhib								0.81	-3.14	0.95	-1.03	-0.60
	0.04% DMSO	100122-13	C01	0.04% dmso								-0.34	-0.59	-2.73	0.60	-0.76
01505907	METHYLPHENIDATE HYDROCHLORIDE	100122-13	C02	sample	298-59-9, 113-45-1(base)	C14H20ClNO2	269.774	CNS stimulant	synthetic	USP, INN, BAN, JAN	Helv Chim Acta 27:1748 (1944); J Pharmacol Exp Therap	-0.78	1.90	1.14	1.20	0.86
01505943	RABEPRAZOLE SODIUM	100122-13	C03	sample	117976-90-6	C18H20N3NaO3S	381.4322	gastric acid secretion inhibitor	synthetic; LY-307640, E-3810	USAN, INN, BAN		0.40	0.76	-0.66	-0.70	-0.05
01505944	RISEDRONATE SODIUM HYDRATE	100122-13	C04	sample	115436-72-1	C7H16NNaO10P2	359.14367	calcium regulator	synthetic; NE-58095	USAN		-3.53	-0.33	-0.46	2.59	-0.43
01505953	SUCRALOSE	100122-13	C05	sample	56038-13-2	C12H19Cl3O8	397.63943	sweetener	semisynthetic	NF, BAN	Arch Oral Biol 27:693 (1982); Trends Biochem Sci 3:61	1.43	-0.41	0.02	0.02	0.26
01505955	COLISTIN SULFATE	100122-13	C06	sample	1264-72-8	C52H102N16O21S2	1351.61534	antibacterial	Bacillus polymyxa colistinus; polymyxin E	USP, INN, BAN, JAN	J Biochem (Tokyo) 54:25, 173, 412 (1963); J Chem Soc	-4.17	-0.61	-1.46	-0.20	-1.61
01505957	ARSENIC TRIOXIDE	100122-13	C07	sample	1327-53-3	As4O6	395.6828	antineoplastic, antileukemia	mineral	USAN, JAN	Pharmacol Ther 76:141 (1997); Blood 93:268 (1999); N	0.59	-0.44	0.48	0.22	0.21
01505958	CLONAZEPAM	100122-13	C08	sample	1622-61-3	C15H10ClN3O3	315.71825	anticonvulsant, sedative	synthetic	USAN	Pediatr Neurol 9:465 (1993); J Forensic Sci 40:382 (1995);	-7.28	-1.78	-0.72	-1.03	-2.70
01505971	BENZBROMARONE	100122-13	C09	sample	117976-90-6	C17H12Br2O3	424.09139	uricosuric	synthetic; MJ-10061, L-2214	USAN, INN, BAN		-0.62	0.00	0.08	-0.64	-0.29
01505972	BROMPERIDOL	100122-13	C10	sample	10457-90-6	C21H23BrFNO2	420.32536	antipsychotic	synthetic; R-11333	USAN, INN, BAN, JAN		1.19	0.80	1.37	0.30	0.92
01505973	CYPROHEPTADINE HYDROCHLORIDE	100122-13	C11	sample	41354-29-4	C21H22ClN	323.86919	H1-antihistamine, antipruritic	synthetic	USP, INN, BAN, JAN		-2.16	-2.01	0.54	1.77	-0.46
	0.11% JAK2inhib	100122-13	C12	0.11% jak2inhib								-0.04	-4.47	0.73	-3.66	-1.86
	0.11% DMSO	100122-13	D01	0.11% dmso								-1.62	-1.66	-2.50	-2.23	-2.00
01505974	CLOFAZIMINE	100122-13	D02	sample	2030-63-9	C27H22Cl2N4	473.40919	antibacterial, antilepretic, antituberculosis	synthetiNSC-141046c; G-30320	USP, INN, BAN		0.78	1.62	-0.77	5.31	1.73
01505975	BENZYDAMINE HYDROCHLORIDE	100122-13	D03	sample	132-69-4	C19H24ClN3O	345.87563	analgesic, antipyretic, antiinflammatory	synthetic; AF-864	USAN, INN, BAN, JAN		-1.43	2.78	-1.33	3.98	1.00
01505976	DOXAZOSIN MESYLATE	100122-13	D04	sample	77883-43-3	C24H29N5O8S	547.59143	antihypertensive	synthetic; UK-33274-27	USAN, INN, BAN, JAN		2.46	0.09	-1.52	2.32	0.84
01505977	ISOETHARINE MESYLATE	100122-13	D05	sample	7279-75-6	C14H25NO6S	335.42245	bronchodilator	synthetic	USP		-0.18	-0.31	0.50	-0.03	-0.01
01505978	FLORFENICOL	100122-13	D06	sample	76639-94-6	C12H14Cl2FNO4S	358.21808	antibacterial	synthetic; SCH-25298	USAN, INN, BAN		-0.02	0.66	1.77	0.40	0.70
01505979	ETHYNODIOL DIACETATE	100122-13	D07	sample	297-76-7	C24H32O4	384.52024	progestin	semisynthetic; SC-11800	USP, INN, BAN, JAN		0.33	-0.29	-0.42	-0.73	-0.28
01505981	ORNIDAZOLE	100122-13	D08	sample	16773-42-5	C7H10ClN3O3	219.62905	antiinfective	synthetic; 16773-42-5	USAN, INN		0.82	0.42	0.42	-0.49	0.29
01505982	OXANTEL PAMOATE	100122-13	D09	sample	68813-55-8	C36H32N2O7	604.66564	anthelmintic	synthetic; CP-14445-16	USAN, INN, BAN		0.21	0.03	0.79	1.48	0.63
01505984	PROTRYPTYLINE HYDROCHLORIDE	100122-13	D10	sample	1225-55-4	C19H22ClN	299.84689	antidepressant	synthetic; MK-240	USP, INN, BAN	J Med Chem 11:325 (1968); N Engl J Med 307:1037 (1982)	-0.86	-0.55	0.13	-0.48	-0.44
01505485	PHYTONADIONE	100122-13	D11	sample	84-80-0	C31H46O2	450.71107	vitamin	semisynthetic	USP, INN, BAN		-4.03	-1.53	0.48	-0.82	-1.47
	0.11% JAK2inhib	100122-13	D12	0.11% jak2inhib								-1.96	-2.90	-2.01	-2.28	-2.29
	0.11% JAK2inhib	100122-13	E01	0.11% jak2inhib								-3.06	-3.64	-5.68	2.56	-2.45
01505987	DENATONIUM BENZOATE	100122-13	E02	sample	3734-33-6	C28H34N2O3	446.59478	denaturing agent, bitter principle	synthetic	USAN, NF, INN, BAN	Chem Ind 22:721 (1988)	-0.07	0.28	0.99	0.44	0.41
01505993	MESALAMINE	100122-13	E03	sample	89-57-6	C7H7NO3	153.13874	antiinflammatory	synthetic; 5-ASA	USP, INN, BAN	Gastroenterology 83:1062 (1982); Am J Gastroenterol	-0.19	-0.86	-1.16	-0.53	-0.69
01505994	ETHAMIVAN	100122-13	E04	sample	304-84-7	C12H17NO3	223.27419	CNS & respiratory stimulant	synthetic; NCS-406087	USAN, USP-XX, INN, BAN	Compt Rendu 243:609 (1956)	0.84	0.39	-0.52	0.97	0.42
01505996	AZTREONAM	100122-13	E05	sample	78110-38-0	C13H21N5O7S2	423.46962	antibacterial	synthetic; SQ-26776	USPXX11, USAN, INN, BAN		0.52	1.23	0.32	-0.46	0.40
01506053	TYLOXAPOL	100122-13	E06	sample	25301-02-4	C70H124O13	1173.76098	polymeric nonionic detergent	synthetic	USP, INN, BAN		0.94	0.51	-0.04	-0.13	0.32
01900002	THIAMYLAL SODIUM	100122-13	E07	sample	337-47-3, 77-27-0 [thiamylal]	C12H17N2NaO2S	276.33529	anesthetic	synthetic	USP-XXII, JAN		0.65	-0.70	-0.46	-0.67	-0.29
01900004	CHLORDIAZEPOXIDE	100122-13	E08	sample	438-41-5, 58-25-3	C16H14ClN3O	299.76248	minor tranquilzer, sedative	synthetic	USP, INN, BAN, JAN		-0.57	0.73	0.32	0.67	0.29
02300094	ASTEMIZOLE	100122-13	E09	sample	68844-77-9	C28H31FN4O	458.58387	H1 antihistamine (nonsedating)	synthetic	USP, INN, BAN, JAN		-0.56	-0.55	0.84	0.16	-0.03
02300154	ACECAINIDE HYDROCHLORIDE	100122-13	E10	sample	34118-92-8, 32795-44-1	C15H24ClN3O2	313.83043	antiarrhythmic	synthetic	USAN, INN		-0.21	0.20	0.70	0.52	0.30
01505430	FLUROTHYL	100122-13	E11	sample	333-36-8	C4H4F6O	182.06628	central stimulant, convulsant	synthetic; SK&F-6539	USP-XXI, INN, BAN	Anesth Analag 95:1611 (2002); 96:1010 (2003)	0.97	-0.06	0.80	0.62	0.58
	0.11% DMSO	100122-13	E12	0.11% dmso								-0.42	-0.57	-0.52	-0.80	-0.58
	0.11% JAK2inhib	100122-13	F01	0.11% jak2inhib								-1.61	-2.85	-3.79	-2.53	-2.69
02300166	ALPRENOLOL	100122-13	F02	sample	13655-52-2, 13707-88-5	C15H24ClNO2	285.81703	betaadrenergic blocker	synthetic	USAN, INN, BAN, JAN		0.18	0.00	-0.87	0.07	-0.16
02300165	AMIODARONE HYDROCHLORIDE	100122-13	F03	sample	1951-25-3	C25H30ClI2NO3	681.78455	adrenergic agonist, coronary vasodilator, Ca channel blocker	synthetic	USAN, INN, BAN, JAN	Adv Drug Res 16: 309 (1987)	0.86	0.84	-0.91	0.40	0.30
02300173	BUSPIRONE HYDROCHLORIDE	100122-13	F04	sample	33386-08-2, 36505-84-7	C21H32ClN5O2	421.97449	5HT1a receptor agonist, anxiolytic	synthetic	USP, INN, BAN		0.29	0.79	-0.16	0.54	0.36
02300242	LOXAPINE SUCCINATE	100122-13	F05	sample	27833-64-3, 1977-10-2	C22H24ClN3O5	445.90668	antipsychotic	synthetic	USP		-0.14	0.61	-0.05	-0.11	0.08
02300206	DIAZOXIDE	100122-13	F06	sample	364-98-7	C8H7ClN2O2S	230.67419	antihypertensive, diuretic, activates K channels and AMPA	synthetic; SCH-6783; NSC-64198	USP, INN, BAN		0.19	-0.59	-1.19	2.96	0.34
02300214	DILTIAZEM HYDROCHLORIDE	100122-13	F07	sample	33286-22-5, 42399-41-7	C22H27ClN2O4S	450.98849	Ca channel blocker, coronary vasodilator	synthetic	USP, INN, BAN, JAN		-0.01	0.58	0.54	-0.42	0.17
02300229	GLYBURIDE	100122-13	F08	sample	10238-21-8	C23H28ClN3O5S	494.01371	antihyperglycemic	synthetic	USP, INN, BAN, JAN		-0.24	1.01	0.15	-0.24	0.17
02300292	MIANSERIN HYDROCHLORIDE	100122-13	F09	sample	21535-47-7, 24219-97-4	C18H21ClN2	300.83447	5HT antagonist	synthetic	USAN, INN, BAN, JAN		0.45	-0.11	1.79	3.41	1.38
02300309	VESAMICOL HYDROCHLORIDE	100122-13	F10	sample	120447-62-3	C17H26ClNO	295.85587	acetylcholine transport blocker (vesicular)	synthetic	USAN		0.01	0.10	0.57	-1.03	-0.09
01505985	NIZATIDINE	100122-13	F11	sample	76963-41-2	C12H21N5O2S2	331.46147	antiulcer, H2 antagonist	synthetic; LY-139037	USP, INN, BAN, JAN	Clin Pharmacol Ther 37:162 (1985); Arzneim Forsch 39:240	0.82	0.25	1.20	0.05	0.58
	0.04% DMSO	100122-13	F12	0.04% dmso								0.48	-0.78	0.38	-0.23	-0.04
	0.04% JAK2inhib	100122-13	G01	0.04% jak2inhib								-1.11	-1.75	-3.41	-0.95	-1.81
02300347	PENTYLENETETRAZOL	100122-13	G02	sample	54-95-5	C6H10N4	138.1734	analeptic, circulation stimulant	synthetic	NF-XIII, INN, BAN		-0.13	0.38	-0.65	-0.79	-0.30
02300259	NICOTINE DITARTRATE	100122-13	G03	sample	65-31-6	C18H26N2O12	462.41412	nicotinyl acetylcholine receptor agonist, ectoparasiticide	Nicotiana tabacum	USAN		0.06	1.21	-0.91	0.04	0.10
02300104	TACRINE HYDROCHLORIDE	100122-13	G04	sample	1684-40-8	C13H15ClN2	234.7309	anticholinesterase, cognitive adjuvant, K channel blocker	synthetic; CL-970	USP		0.67	0.72	-0.41	0.77	0.44
01500252	DIMERCAPROL	100122-13	G05	sample	59-52-9	C3H8OS2	124.22461	chelating agent (As, Au, Hg antidote)	synthetic	USP, INN, BAN, JAN		0.57	0.03	0.03	0.46	0.27
02300325	METOLAZONE	100122-13	G06	sample	17560-51-9	C16H16ClN3O3S	365.84122	diuretic, antihypertensive	synthetic	USP, INN, BAN, JAN		1.12	0.29	0.24	-0.12	0.38
02300161	AMOXAPINE	100122-13	G07	sample	14028-44-5	C17H16ClN3O	313.78957	antidepressant, inhibits norepinephrine uptake	synthetic	USP, INN, BAN, JAN		-2.73	-2.77	-2.58	-2.06	-2.54
01505995	BUTYL PARABEN	100122-13	G08	sample	94-26-8	C11H14O3	194.23243	antifungal, preservative	synthetic	USAN, NF, BAN		-0.80	0.19	-0.18	3.27	0.62
01505991	DECAMETHONIUM BROMIDE	100122-13	G09	sample	541-22-0	C16H38Br2N2	418.30266	neuromuscular blocker	synthetic	USP-XX, INN, BAN		0.68	-0.46	-0.02	-0.47	-0.07
01505294	CARBADOX	100122-13	G10	sample	6804-07-5	C11H10N4O4	262.22675	antibacterial	synthetic	USAN, INN, BAN	J Vet Med Sci 65:1275 (2003)	0.23	-0.29	0.55	-1.13	-0.16
01503721	ENROFLOXACIN	100122-13	G11	sample	93106-60-6	C19H22FN3O3	359.40389	antibacterial	synthetic	USAN, INN, BAN		0.74	3.39	0.40	-0.08	1.11
	0.04% DMSO	100122-13	G12	0.04% dmso								0.90	-0.01	1.05	0.16	0.52
	0.04% JAK2inhib	100122-13	H01	0.04% jak2inhib								-1.26	-1.42	-3.17	-2.76	-2.15
01505420	DEXPANTHENOL	100122-13	H02	sample	81-13-0	C9H19NO4	205.25608	cholinergic	semisynthetic	USP, INN, BAN		-0.01	-0.05	-1.28	-0.31	-0.41
01505292	NONOXYNOL-9	100122-13	H03	sample	26027-38-3	C33H60O10	616.84015	spermatocide, contraceptive	synthetic	USP, INN	J Steroid Biochem Mol Biol 65:143 (1998)	-1.57	-1.94	-2.31	-2.11	-1.98
01505729	DOCOSANOL	100122-13	H04	sample	661-19-8	C22H46O	326.61132	antiviral	Pygeum africanum	USAN	Antiviral Res 40:85 (1998)	-0.45	-0.16	-0.87	-0.16	-0.41
01505447	OCTODRINE	100122-13	H05	sample	543-82-8	C8H19N	129.24733	vasoconstrictor, anesthetic (local)	synthetic; SK&F-51	USAN, INN		-0.16	-0.56	-0.90	-0.93	-0.64
01503078	ANIRACETAM	100122-13	H06	sample	72432-10-1	C12H13NO3	219.24231	cognitive enhancer	synthetic	USAN, INN, JAN		1.90	0.03	-0.76	-0.37	0.20
01503611	PENTOXIFYLLINE	100122-13	H07	sample	6493-05-6	C13H18N4O3	278.31341	PDE inhibitor, bronchodilator, vasodilator	synthetic	USP, INN, BAN, JAN		0.02	1.09	-0.35	3.19	0.99
01505122	AZTREONAM	100122-13	H08	sample	78110-38-0	C13H17N5O8S2	435.43714	antibiotic	semisynthetic; SQ-26776	USP, INN, BAN, JAN		0.41	1.05	1.29	0.00	0.69
01500164	CEFAZOLIN SODIUM	100122-13	H09	sample	27164-46-1, 25953-19-9	C14H13N8NaO4S3	476.49271	antibacterial	semisynthetic	USP, INN, BAN		0.39	0.92	-0.13	1.07	0.56
01500602	TUBOCURARINE CHLORIDE	100122-13	H10	sample	6989-98-6, 57-94-3	C37H41ClN2O6	645.20212	muscle relaxant (skeletal)	Chondodendron spp	USP, INN, BAN, JAN	Nature 158: 515 (1946); Chem Commun 1970: 1020	1.54	0.76	0.69	1.33	1.08
01500582	TOLMETIN SODIUM	100122-13	H11	sample	64490-92-2, 26171-23-3	C15H14NNaO3	279.27353	antiinflammatory	synthetic	USP, JAN		-1.06	-1.88	-0.01	5.97	0.76
	empty	100122-13	H12	sample								NA	NA	NA	NA	NA
	empty	100122-14	A01	sample								NA	NA	NA	NA	NA
01505990	CLEMIZOLE HYDROCHLORIDE	100122-14	A02	sample	1163-36-6, 442-52-4(base)	C19H21Cl2N3	362.30532	H1-antihistamine	synthetic; AL-20	INN, BAN, JAN	Ann 575:162 (1952)	-1.67	-1.50	-0.97	-1.15	-1.32
01500632	HEXESTROL	100122-14	A03	sample	5635-50-7	C18H22O2	270.37484	estrogen, antineoplastic (hormonal)	synthetic; NSC-9894	NF-XI, INN		-0.78	-0.50	-1.56	-0.72	-0.89
01504523	FTAXILIDE	100122-14	A04	sample	19368-18-4	C16H15NO3	269.30285	antiulcer	synthetic; MP-12	INN		-1.34	-1.35	-1.17	-1.30	-1.29
01503045	ACEXAMIC ACID	100122-14	A05	sample	57-08-9	C8H15NO3	173.21365	wound healing agent	synthetic	INN, BAN		0.43	-0.75	-0.70	-0.34	-0.34
01503032	DIPYROCETYL	100122-14	A06	sample	486-79-3	C11H10O6	238.19875	antirheumatic, analgesic	synthetic	INN	Bul Chem Soc Japan 16: 284 (1941); J Pharm Pharmacol	-0.37	-0.72	-1.16	-1.06	-0.83
01505117	ESTRAGOLE	100122-14	A07	sample	140-67-0	C10H12O	148.20654	insect atttractant, skin irritant, carcinogen	numerous plant essential oils	experimental	JACS 55:1556 (1933); Food Chem Toxicol 28:537 (1990)	-0.84	-0.26	-0.45	1.91	0.09
01503017	AMINOTHIAZOLE	100122-14	A08	sample	96-50-4	C3H4N2S	100.14273	antithyroid agent	synthetic; RP-2921	INN		0.79	-0.15	0.62	-1.14	0.03
01503105	BENFOTIAMINE	100122-14	A09	sample	22457-89-2	C19H23N4O6PS	466.45616	vitamin B1	Vitamin B1; 8088-CB	INN, JAN		0.08	0.29	-1.40	-0.88	-0.48
01500679	AMINOPTERIN	100122-14	A10	sample	54-62-6, 58602-66-7	C19H20N8O5	440.42185	antineoplastic, antirheumatic, folic acid antagonist	synthetic; NSC-739	INN, BAN		-6.65	-7.74	-7.80	-10.61	-8.20
00201606	RUTILANTINONE	100122-14	A11	sample	21288-61-9	C22H20O9	428.3993	coccidiostat	Streptomyces species	experimental	Chem Ber 92: 1880 (1959); 100: 2561 (1967)	0.10	0.57	-0.60	-1.16	-0.27
	0.04% JAK2inhib	100122-14	A12	0.04% jak2inhib								0.84	-1.06	-1.25	-2.86	-1.08
	0.04% DMSO	100122-14	B01	0.04% dmso								-1.73	-2.03	-0.23	-1.03	-1.26
01500195	CLOFIBRIC ACID	100122-14	B02	sample	882-09-7	C10H11ClO3	214.65037	antihyperlipoproteinemic	synthetic	INN		-0.92	-0.91	-0.85	2.48	-0.05
01505807	AVOCADENOFURAN	100122-14	B03	sample	25346-24-1	C17H28O	248.41211		Persea spp	undetermined activity	Tetrahedron 25:4617(1969); An Acad Brasil Cienc 42	-0.52	-0.34	-0.07	-0.02	-0.24
00330086	DIBUTYL PHTHALATE	100122-14	B04	sample	84-74-2	C16H22O4	278.35134	plasticiser, suspect endocrine disruptor	synthetic	NF		-0.03	-1.16	-1.37	-0.94	-0.87
01503320	TIMONACIC	100122-14	B05	sample	444-27-9	C4H7NO2S	133.16989	hepatoprotectant	synthetic	INN		-0.94	0.13	-1.69	-0.95	-0.86
00210925	METHYL ORSELLINATE	100122-14	B06	sample	3187-58-4	C9H10O4	182.17765		lichens and lichen acids	undetermined activity	Ber 56: 2556 (1923); JACS 88: 2053 (1966); Tetrahedron	-0.14	-0.55	-0.03	-1.02	-0.43
01503114	DIBEKACIN	100122-14	B07	sample	34493-98-6	C18H39N5O12S	549.60183	antibacterial	semisynthetic	INN, BAN		0.50	0.29	0.75	0.61	0.54
01503920	CLOPERASTINE HYDROCHLORIDE	100122-14	B08	sample	3703-76-2	C20H25Cl2NO	366.33435	antitussive	synthetic; HT-11	INN, JAN		-1.41	-0.05	-1.67	-1.87	-1.25
01504104	DIHYDROJASMONIC ACID	100122-14	B09	sample	98674-52-3	C12H20O3	212.2914	plant growth regulator	Jasminium spp and Vicia faba	experimental	Helv Chim Acta 57: 1301 (1974); Tet Lett 3413 (1974);	0.76	0.27	0.01	-0.24	0.20
01500641	PENTAMIDINE ISETHIONATE	100122-14	B10	sample	100-33-4	C23H36N4O10S2	592.69217	antiprotozoal, inhibits nucleic acid & protein synthesis	synthetic; MB-800, RP-2512	INN, BAN, JAN		-0.98	-1.53	-0.67	-1.54	-1.18
00310006	HYDRASTININE HYDROCHLORIDE	100122-14	B11	sample	4884-68-8, 6592-85-4	C11H14ClNO3	243.69213	cardiotonic, uterine hemostatic	derivative of hydrastine (01501009)	INN, NF-VIII		0.91	0.15	-1.45	-2.07	-0.62
	0.04% JAK2inhib	100122-14	B12	0.04% jak2inhib								0.11	-0.57	-1.57	-1.66	-0.92
	0.04% DMSO	100122-14	C01	0.04% dmso								-2.04	-1.52	-0.85	-0.30	-1.18
01500281	ERYTHROMYCIN STEARATE	100122-14	C02	sample	643-22-1; 114-07-8(base)	C55H103NO15	1018.43186	antibacterial	Streptomyces erythreus	INN, BAN, JAN		-7.55	-2.98	-1.38	0.62	-2.82
01300042	SODIUM TETRADECYL SULFATE	100122-14	C03	sample	139-88-8	C14H29NaO4S	316.43863	sclerosing agent	synthetic	INN	J Ind Hyg Toxicol 30:63 (1948)	2.55	-0.07	-0.59	-0.57	0.33
01505256	SILIBININ	100122-14	C04	sample	22888-70-6	C25H22O10	482.44809	hepatoprotective agent, antioxidant	Silybum marianum	INN	Arzneim-Forsch 20: 1248 (1970); Chem Ber 108: 790, 1482	-2.12	-1.02	-1.89	-2.06	-1.77
01500633	HEXETIDINE	100122-14	C05	sample	141-94-6	C21H45N3	339.6129	antifungal	synthetic	BAN		-0.12	1.16	0.93	1.08	0.76

Compound ID	MolNAME	plate	position	Content	cas#	Formula	MolWt	Bioactivity	Source	Status	Reference	Screen Score (z-score) rep1	rep2	rep3	rep4	Average
01503222	FIPEXIDE HYDROCHLORIDE	100122-14	C06	sample	34161-24-5	C20H22Cl2N2O4	425.31534	psychostimulant	synthetic	INN	hepatotoxic at clinical dose (600mg)	1.89	-0.44	-0.98	-0.44	0.01
01500898	EMODIN	100122-14	C07	sample	518-82-1	C15H10O5	270.24395	antibacterial, antineoplastic, cathartic, tyrosine kinase inhibitor	Cascara, Rheum and Rhammnus species	experimental	Oncogene 17: 913 (1998); Planta Med 65: 9 (1999); Clin	1.63	-0.10	-0.20	-0.26	0.27
01505168	ETHACRIDINE LACTATE	100122-14	C08	sample	1837-57-6; 6402-23-9 (hydrate)	C18H21N3O4	343.38577	antiseptic, abortifaceant	synthetic	INN, BAN, JAN	Clin Obster Gynacol 11:573 (1984)	-0.99	-0.53	-0.82	0.00	-0.58
01506049	meta-CRESYL ACETATE	100122-14	C09	sample	122-46-3	C9H10O2	150.17885	antiseptic (topical)	synthetic	INN		0.02	0.38	0.33	0.38	0.28
01505300	PURPURIN	100122-14	C10	sample	81-54-9	C14H8O5	256.21686	xanthin oxidase inhibitor, irritant	Rubia and Gallium spp	experimental	J Chem Soc 1928:229; 1929:1399; 1931:3206; Anticancer	-0.13	1.65	1.08	3.05	1.41
01502012	FOSFOSAL	100122-14	C11	sample	6064-83-1	C7H7O6P	218.10404	analgesic, antiinflammatory	synthetic	INN		1.24	2.23	3.78	0.00	1.81
	0.11% JAK2inhib	100122-14	C12	0.11% jak2inhib								-1.91	-1.34	-1.15	3.03	-0.34
	0.11% DMSO	100122-14	D01	0.11% dmso								-2.09	-0.60	-2.10	-0.74	-1.38
01503080	AMBROXOL HYDROCHLORIDE	100122-14	D02	sample	23828-92-4, 18683-91-5	C13H19Br2ClN2O	414.57018	expectorant	synthetic	INN, BAN, JAN		-1.35	-1.32	-1.11	0.76	-0.75
01505276	CAPSANTHIN	100122-14	D03	sample	465-42-9	C40H56O3	584.89052	antineoplastic	Capsicum annum	experimental		-0.35	0.79	1.80	1.46	0.93
01505155	3-HYDROXYTYRAMINE	100122-14	D04	sample	62-31-7	C8H11NO2	153.18237	dopaminergic	synthetic	experimental	Am J Physiol 267:R628 (1994); FEBS Lett 325:215 (1993)	-1.01	-0.94	-0.33	-1.19	-0.87
00201664	CELASTROL	100122-14	D05	sample	34157-83-0	C29H38O4	450.62381	antineoplastic, antiinflamatory, NO synthesis inhibitor, chaperone	Celastrus scandens & Tripterygium wilfordii	experimental	J Chem Soc 1963: 2884; 1972: 330; J Org Chem 30: 1729	0.03	1.85	0.98	0.27	0.78
01505293	DIALLYL SULFIDE	100122-14	D06	sample	592-88-1	C6H10S	114.2106	antibacterial, antifungal, antineoplastic, antihypercholesterolemic,	Allium spp, Wasabia japonica	experimental	Arch Biochem Biophys 107:137 (1964); JACS 104:312	-0.04	-0.21	-1.50	-0.01	-0.44
00100528	PEUCENIN	100122-14	D07	sample	578-72-3	C15H16O4	260.29237		Peucedanum ostruthium, Ptaeroxylon obliquum	undetermined activity	Ber 74:1851 (1941); J Chem Soc (C) 1967:145	-0.06	0.19	-0.01	-1.32	-0.30
00107108	5alpha-ANDROSTAN-3,17-DIONE	100122-14	D08	sample		C19H28O2	288.43381	androgen	human urine & adrenal cortex	experimental		1.22	0.84	0.09	2.39	1.13
00212151	CHLORQUINALDOL	100122-14	D09	sample	72-80-0	C10H7Cl2NO	228.07939	antiinfectant, antifungal	synthetic	INN, BAN		1.56	1.17	0.43	-0.49	0.67
01503711	ORBIFLOXACIN	100122-14	D10	sample	113617-63-3	C19H20F3N3O3	395.38475	antibacterial	synthetic	INN		0.89	0.98	-0.16	-0.89	0.21
01501199	ZAPRINAST	100122-14	D11	sample	37762-06-4	C13H13N5O2	271.28086	cGMP phosphodiesterase inhibitor	synthetic; M&B-22948	INN, BAN		0.38	0.57	0.28	0.18	0.35
	0.11% JAK2inhib	100122-14	D12	0.11% jak2inhib								-2.14	-0.83	-1.51	-3.94	-2.10
	0.11% JAK2inhib	100122-14	E01	0.11% jak2inhib								-3.69	-2.92	-4.31	-2.30	-3.30
00102058	OLEANOLIC ACID ACETATE	100122-14	E02	sample	4339-72-4	C32H50O4	498.7529		birch bark	undetermined activity	J Chem Soc 1939: 1047	-2.60	-3.35	1.67	-2.43	-1.68
00210658	DEHYDROVARIABILIN	100122-14	E03	sample		C17H14O4	282.29873		Dalbergia variabilis	undetermined activity	Phytochemistry 17: 1417 (1978)	0.22	0.28	-0.05	0.75	0.30
00211475	4'-METHOXYCHALCONE	100122-14	E04	sample	22966-19-4	C16H14O2	238.28878		Citrus limon	undetermined activity	Acta Cryst C 48:741 (1992)	-0.95	0.32	-0.86	0.64	-0.21
00200141	2-ETHOXYCARBONYL-2-	100122-14	E05	sample		C24H24O9	456.45348		mp 111-114 C	undetermined activity		-0.46	0.81	0.70	1.40	0.61
01506073	IDAZOXAN HYDROCHLORIDE	100122-14	E06	sample	79944-58-4	C11H13ClN2O2	240.69146	alpha2-adrenergic blocker	synthetic	INN, BAN	Ann NY Acad Sci 881:272 (1999); CNS Drug Reviews	-0.02	-0.44	0.46	0.36	0.09
01505827	BECLAMIDE	100122-14	E07	sample	501-68-8	C10H12ClNO	197.66624	anticonvulsant, antiepileptic	synthetic; mp 94 deg C	INN, BAN	J Org Chem 16:1283 (1951)	-0.34	0.98	-0.14	-0.60	-0.02
00231043	1,3,5-TRIMETHOXYBENZENE	100122-14	E08	sample	621-23-8	C9H12O3	168.19419	spasmolytic	synthetic	experimental		-0.22	0.38	0.73	0.20	0.27
00270043	3beta-HYDROXY-23,24-BISNORCHOL-5-ENIC ACID	100122-14	E09	sample		C22H34O3	346.51448		semisynthetic	undetermined activity		1.17	2.88	1.40	0.91	1.59
01504070	PHYSCION	100122-14	E10	sample	521-61-9	C16H12O5	284.27104	antibacterial, cathartic	Xanthoria lichens, Rumex spp and various Aspergillus spp.	experimental	Helv Chim Acta 8: 140 (1925); Pharmacology 14: 1 (1976)	0.12	1.19	1.16	-0.16	0.58
01503939	RIBOSTAMYCIN SULFATE	100122-14	E11	sample	25546-65-0	C17H36N4O14S	552.55887	antibacterial	Streptomyces ribosidificus	INN, BAN, JAN		1.71	0.16	-0.88	-0.13	0.21
	0.11% DMSO	100122-14	E12	0.11% dmso								0.81	1.69	0.10	-1.97	0.16
	0.11% JAK2inhib	100122-14	F01	0.11% jak2inhib								-4.15	-3.28	-3.35	-3.41	-3.55
02300345	NIPECOTIC ACID	100122-14	F02	sample	498-95-3	C6H11NO2	129.16007	GABA uptake inhibitor	synthetic	experimental	J Neurochem 25: 797 (1975); Life Sci 19: 701 (1976)	-1.55	-1.53	-0.31	0.49	-0.73
02300348	1-(2-METHOXYPHENYL)PIERAZINE HYDROCHLORIDE	100122-14	F03	sample		C11H17ClN2O	228.72394	5HT1 receptor agonist	synthetic	experimental	Biochem Pharmacol 29: 833 (1980); J Med Chem 30: 1	0.21	0.00	-1.68	-1.15	-0.66
01505870	4-HYDROXYANTIPYRINE	100122-14	F04	sample	1672-63-5	C11H12N2O2	204.23049	analgesic	synthetic	experimental		1.38	1.41	0.04	-0.03	0.70
01400010	IPRIFLAVONE	100122-14	F05	sample	35212-22-7	C18H16O3	280.32642	anabolic	synthetic	INN, JAN		-0.45	0.97	1.64	0.29	0.61
01502085	CYCLOCREATINE	100122-14	F06	sample	35404-50-3	C5H9N3O2	143.14638	regulator of creatine biosynthesis	heart & muscle tissue	experimental		0.16	0.58	1.04	0.70	0.62
01502096	alpha-CYANO-4-HYDROXYCINNAMIC ACID	100122-14	F07	sample		C10H7NO3	189.17219	inhibitor of mitochondrial pyruvate transport	synthetic	experimental		0.72	0.24	0.57	0.27	0.45
01506052	TROLOX	100122-14	F08	sample	53188-07-1	C14H18O4	250.29716	antioxidant	synthetic	experimental	Food Chem Toxicol 42:45 (2004); Bioorg Med Chem Letts	1.05	0.33	0.70	0.07	0.53
00330009	SODIUM FLUOROACETATE	100122-14	F09	sample	62-74-8	C2H2FNaO2	100.02524	inhibits citric acid cycle	synthetic	experimental		0.35	1.43	2.07	0.59	1.11
01505899	HYDROQUININE HYDROBROMIDE HYDRATE	100122-14	F10	sample	207386-86-5	C20H29BrN2O3	425.36973	depigmentor	semisynthetic; derivatice of quinine	experimental		0.61	2.00	1.34	0.26	1.05
01505761	OXYTHIAMINE CHLORIDE HYDROCHLORIDE	100122-14	F11	sample	614-05-1	C12H17Cl2N3O2S	338.25819	thiamine antagonist	synthesis	experimental	Science 110:162 (1949); Proc Soc Exp Med 72:165 (1949)	0.95	1.37	0.88	0.06	0.81
	0.04% DMSO	100122-14	F12	0.04% dmso								1.43	5.50	-0.06	-0.64	1.56
	0.04% JAK2inhib	100122-14	G01	0.04% jak2inhib								-1.67	-1.99	-0.30	-1.21	-1.29
00307123	ESTRONE BENZOATE	100122-14	G02	sample		C25H26O3	374.48417		semisynthetic	undetermined activity		0.16	-1.05	-1.05	-0.40	-0.58
01501126	5-AMINOPENTANOIC ACID HYDROCHLORIDE	100122-14	G03	sample		C5H12ClNO2	153.60989	GABAb antagonist	synthetic	experimental	Biochem Pharmacol 30: 1105 (1981)	0.83	2.15	0.96	0.90	1.21
01500699	ACETYLTRYPTOPHANAMIDE	100122-14	G04	sample		C13H15N3O2	245.2834	antidepressant, nutrient	synthetic	experimental		-0.79	-0.64	0.79	0.30	-0.09
01500702	ACETYLTRYPTOPHAN	100122-14	G05	sample	1218-34-4	C13H14N2O3	246.26813	antidepressant	synthetic	experimental		0.83	0.01	-0.41	0.59	0.26
01500762	ROSOLIC ACID	100122-14	G06	sample		C19H14O3	290.32163	diagnostic aid	synthetic	experimental		0.26	-0.01	0.41	0.98	0.41
01505862	2-AMINOBENZENESULFONAMIDE	100122-14	G07	sample		C6H8N2O2S	172.20686	antibacterial	synthetic	experimental		0.28	-0.36	-0.09	-0.17	-0.09
01504617	ISAXONINE	100122-14	G08	sample	4214-72-6	C7H11N3	137.18582	nerve growth stimulant	synthetic	INN	hepatotoxic at clinical dose (1500mg)	-0.29	1.22	1.05	2.59	1.14
00210515	PYROGALLIN	100122-14	G09	sample		C11H8O4	204.18401	antiinfectant	synthetic	experimental	aq KIO4 + catechol + pyrogallol	-0.37	-0.45	0.27	-0.33	-0.22
00201505	METHYL 7-DESHYDROXYPYROGALLIN-4-	100122-14	G10	sample	77-41-8	C13H10O6	262.22105		synthetic	undetermined activity		2.15	-0.57	0.54	0.63	0.69
01503207	CYCLOBENZAPRINE HYDROCHLORIDE	100122-14	G11	sample	6202-23-9, 303-53-7	C20H22ClN	311.85804	muscle relaxant (skeletal)	synthetic	USP, INN		-0.80	-0.87	-0.03	-1.00	-0.67
	0.04% DMSO	100122-14	G12	0.04% dmso								1.30	0.94	0.66	0.21	0.78
	0.04% JAK2inhib	100122-14	H01	0.04% jak2inhib								-2.55	-2.34	-2.77	1.77	-1.47
00300563	TRICHLORMETHINE	100122-14	H02	sample	555-77-1	C6H13Cl4N	240.98921	antineoplastic, cytotoxic	synthetic	INN, BAN		-0.12	-1.08	-1.13	1.75	-0.15
01500736	3,6-DIMETHOXYFLAVONE	100122-14	H03	sample		C17H14O4	282.29873		semisynthetic	undetermined activity		-0.56	-1.28	-0.22	0.22	-0.46
01500734	3,4'-DIMETHOXYFLAVONE	100122-14	H04	sample		C17H14O4	282.29873		derivative Millettia flowers	undetermined activity		-0.52	-0.05	0.04	0.11	-0.11
01505770	FURALTADONE	100122-14	H05	sample	139-91-3	C13H16N4O6	324.29567	antibacterial	synthetic; NF-260	INN, BAN		-1.41	-1.54	1.21	-0.89	-0.66
01300010	ARGININE HYDROCHLORIDE	100122-14	H06	sample	1119-34-2	C6H15ClN4O2	210.66505	ammonia detoxicant, diagnostic aid	widespread in nature	USP		0.73	1.87	0.40	1.03	1.01
01505241	SPARTEINE HYDROIODIDE	100122-14	H07	sample		C15H27IN2	362.30024	oxytocic	Lupinus spp and other Leguminosae	experimental	J Chem Soc 1949: 663	0.74	-0.68	0.01	1.14	0.30
00100114	3alpha-HYDROXY-3-DEOXYANGOLENSIC ACID	100122-14	H08	sample		C27H36O7	472.58377		Meliaceae spp	undetermined activity		-0.27	-0.17	1.02	-0.24	0.09
01300019	OXIGLUTATIONE DISODIUM SALT	100122-14	H09	sample	103239-24-3; 27025-41-8(acid)	C20H30N6Na2O12S2	656.6027	antioxidant	synthetic	JAN		0.41	0.52	0.04	0.17	0.28
00201580	POMIFERIN	100122-14	H10	sample	572-03-2	C25H24O6	420.46643	antioxidant	Maclura pomifera	experimental	JACS 61:2832 (1939); 62:651 (1940); 63:1253 (1941);	0.19	0.00	2.21	-0.66	0.44
01505895	GLYCOCHOLIC ACID	100122-14	H11	sample	475-31-0	C26H43NO6	465.63571		mammalian bile	undetermined activity		0.46	0.67	1.19	0.16	0.62
	empty	100122-14	H12	sample								NA	NA	NA	NA	NA
	empty	100122-15	A01	sample								NA	NA	NA	NA	NA
01500623	BROXYQUINOLINE	100122-15	A02	sample	521-74-4	C9H5Br2NO	302.9543	antiinfectant, disinfectant	synthetic	INN		-1.00	-1.66	-2.18	-0.89	-1.43
01503424	ALFAXALONE	100122-15	A03	sample	23930-19-0	C21H32O3	332.48739	anesthetic	semisynthetic	INN, BAN, JAN		-1.26	-0.86	-1.42	-0.11	-0.91
01500999	DROFENINE HYDROCHLORIDE	100122-15	A04	sample	1679-76-1	C20H32ClNO2	353.93654	antispasmodic	synthetic	INN		-1.82	-3.70	-2.21	-3.23	-2.74
01501000	ETHAVERINE HYDROCHLORIDE	100122-15	A05	sample	985-13-7, 486-47-5	C24H30ClNO4	431.964	antispasmodic	synthetic	INN		-0.84	0.18	-0.88	-0.80	-0.58
01501026	FENDILINE HYDROCHLORIDE	100122-15	A06	sample	113042-18-7	C23H26ClN	351.92337	coronary vasodilator	synthetic	INN		-0.43	-0.78	0.68	-0.94	-0.37
01501114	PIMETHIXENE MALEATE	100122-15	A07	sample	314-03-4	C23H23NO4S	409.50806	H1 antihistamine	synthetic; BP-400	INN		-1.48	-2.31	-3.02	-3.19	-2.50
01501159	SULMAZOLE	100122-15	A08	sample	73384-60-8	C14H13N3O2S	287.34261	cardiotonic	synthetic	INN		-0.10	0.01	0.48	-1.40	-0.25
01501170	ACETAMINOSALOL	100122-15	A09	sample	118-57-0	C15H13NO4	271.27516	analgesic, antipyretic	synthetic	INN		-0.01	1.02	1.59	-1.06	0.39
01501188	EBSELEN	100122-15	A10	sample	60940-34-3	C13H9NOSe	274.18278	antioxidant, lipoxygenase inhibitor, inhibits oxidation of LDL	synthetic	INN		-0.39	-0.64	0.29	-1.18	-0.48
01502001	ACETYL-L-LEUCINE	100122-15	A11	sample	99-15-0	C8H15NO3	173.21365	antivertigo	synthetic	INN		0.96	0.79	0.22	2.47	1.11
	0.04% JAK2inhib	100122-15	A12	0.04% jak2inhib								0.01	-1.44	-2.93	-3.84	-2.05
	0.04% DMSO	100122-15	B01	0.04% dmso								-0.08	2.03	-1.31	-0.45	0.05
01800031	URSOLIC ACID	100122-15	B02	sample	77-52-1	C30H48O3	456.71526	diuretic, antineoplastic, antiulcer	Rhododendron spp, Epigaea asiatica, surface wax of fruits	experimental	Phytochemistry 10:3279 (1971)	-0.91	-0.61	-1.53	-0.79	-0.96
00300062	METAMECONINE	100122-15	B03	sample		C10H10O4	194.1888		Accacia crombei	undetermined activity		-0.55	-1.22	-0.47	-0.09	-0.58
01503085	PYRITHYLDIONE	100122-15	B04	sample	77-04-3	C9H13NO2	167.20946	hypnotic, sedative	synthetic	INN		0.15	-0.63	-0.46	-0.02	-0.24
00201182	IRIGENOL	100122-15	B05	sample	4935-93-7	C15H10O8	318.24215		Iris spp	undetermined activity		0.62	-0.97	3.93	2.35	1.48
01503381	PASINIAZID	100122-15	B06	sample	2066-89-9	C13H14N4O4	290.28093	antibacterial (tuberculostatic)	synthetic; RD-325	INN		0.80	-0.57	0.50	-0.52	0.05
01505864	3-ACETYLCOUMARIN	100122-15	B07	sample		C11H8O3	188.18461		synthetic	experimental		0.78	-0.06	0.52	-0.77	0.12
01505863	3-ACETAMIDOCOUMARIN	100122-15	B08	sample		C11H9NO3	203.19928		synthetic	experimental		0.52	1.06	1.87	0.37	0.96
01503648	GABOXADOL HYDROCHLORIDE	100122-15	B09	sample	64603-91-4	C6H9ClN2O2	176.60383	GABAa agonist, GABAc antagonist	synthetic; THIP hydrochloride	INN	J Med Chem 24: 1377 (1981); 26: 895 (1983); Drugs of the	0.06	0.87	0.39	1.09	0.60
01503822	RAMIFENAZONE	100122-15	B10	sample	3615-24-5	C14H20ClN3O	281.788	analgesic, antipyretic, antiinflammatory	synthetic	INN		0.75	1.76	0.06	-0.27	0.57
01504100	METHOPRENE (S)	100122-15	B11	sample	40596-69-8	C19H34O3	310.48133	ectoparasiticide	synthetic	INN		-5.34	-3.30	-5.12	-4.46	-4.55
	0.04% JAK2inhib	100122-15	B12	0.04% jak2inhib								0.50	0.70	-1.76	-1.64	-0.55
	0.04% DMSO	100122-15	C01	0.04% dmso								-2.02	0.01	-2.13	-1.65	-1.45
01504187	TELENZEPINE HYDROCHLORIDE	100122-15	C02	sample	80880-90-6	C19H23ClN4O2S	406.93776	antiulcer, M1 muscarinic antagonist	synthetic	INN	Gen Pharmacol 21: 17 (1990)	-0.78	-1.17	-0.43	0.11	-0.56
01505865	6-HYDROXYTROPINONE	100122-15	C03	sample		C8H13NO2	155.19831		synthetic	experimental		-0.45	-0.55	0.36	6.26	1.41
01505779	DICHLORISONE ACETATE	100122-15	C04	sample	79-61-8	C23H28Cl2O5	455.38261	antipruretic	semisynthetic; R-25788	INN	JACS 81:2191 (1959)	2.04	-1.15	-0.46	0.14	0.14
01505788	NIFUROXAZIDE	100122-15	C05	sample	965-52-6	C12H9N3O5	275.22263	antiseptic	synthetic	INN	Ann Pharm Fr 21:287 (1963)	-1.19	-1.81	-0.24	-1.25	-1.12
00300104	2-METHYLENE-5-(2,5-DIOXOTETRAHYDROFURAN-3-	100122-15	C06	sample		C18H24O4	304.38958		derivative	undetermined activity		-1.00	-0.15	0.01	0.59	-0.14
00300105	beta-CARYOPHYLLENE ALCOHOL	100122-15	C07	sample		C15H26O	222.37387		palmarosa oil, Cymbopogon martini	undetermined activity	Aust J Chem 41: 1755 (1988)	0.08	-0.33	0.14	-0.43	-0.13
01505892	FUSARIC ACID	100122-15	C08	sample	536-69-6	C9H11NO2	165.19352	antiproliferative, dopamine beta-hydroxylase inhibitor	Fusarium spp	experimental		-0.04	-0.48	0.95	3.29	0.93
00300106	3-AMINO-beta-PINENE	100122-15	C09	sample		C10H18ClN	187.71466		derivative	undetermined activity	Helv Chim Acta 46: 1480 (1963)	2.16	-0.01	0.34	5.70	2.05
00210505	PURPUROGALLIN	100122-15	C10	sample	569-77-7	C11H8O5	220.18341	xanthine oxidase inhibitor, antioxidant	gall of Dryophanta divisa	experimental	J Chem Soc 1949: 3271; Biotechnol Biochem 61: 890	0.51	-0.01	2.25	-0.79	0.49
00300565	METERGOLINE	100122-15	C11	sample	17692-51-2	C25H29N3O2	403.52878	analgesic, antipyretic	synthetic; FI-6337, MCE	INN, BAN		0.01	-0.29	3.02	-0.56	0.54
	0.11% JAK2inhib	100122-15	C12	0.11% jak2inhib								0.39	-1.67	-2.48	5.76	0.50
	0.11% DMSO	100122-15	D01	0.11% dmso								-1.91	-2.14	-1.71	-2.29	-2.01
01300030	OMEGA-3-ACID ESTERS (EPA shown)	100122-15	D02	sample	86227-47-6	C22H34O2	330.51508	hypolipidemic	semisynthetic; K-85	USAN		-5.26	-1.61	-1.50	-1.19	-2.39
01500626	DICHLOROPHENE	100122-15	D03	sample	97-23-4	C13H10Cl2O2	269.12945	anthelmintic	synthetic	INN, BAN		-1.14	-0.91	-0.01	0.25	-0.45
01505876	CHICAGO SKY BLUE	100122-15	D04	sample	2610-05-1	C34H24N6Na4O16S4	992.81618	inhibitor of aminoacid uptake	synthetic	experimental	J.Neurochem. 63 Suppl 1:S49A (1994); 65:96 (1995)	-0.50	-0.01	-0.54	1.45	0.10
01500636	MECYSTEINE HYDROCHLORIDE	100122-15	D05	sample	2485-62-3, 18598-63-5	C4H10ClNO2S	171.6468	mucolytic	synthetic	INN, BAN		0.48	0.54	0.46	1.34	0.71
01505992	BUFLOMEDIL HYDROCHLORIDE	100122-15	D06	sample	35543-24-9, 55837-25-7(base)	C17H26ClNO4	343.85407	vasodilator (peripheral)	synthetic	INN, BAN	Therapie 30:207, 259 (1975); Drugs 33:430 (1987)	0.33	0.81	0.00	-0.96	0.04
01503628	PRONETALOL HYDROCHLORIDE	100122-15	D07	sample	54-80-8	C15H20ClNO	265.78575	beta adrenergic agonist, antiarrhythmic	synthetic; ICI-38174, AY-6204	INN, BAN		0.21	0.37	0.17	0.79	0.39
01505898	HEMICHOLINIUM BROMIDE	100122-15	D08	sample	312-45-8	C24H34Br2N2O4	574.35758	acetylcholine antagonist	synthetic	experimental		0.96	0.06	0.16	-0.20	0.24
01503806	HOMIDIUM BROMIDE	100122-15	D09	sample	1239-45-8	C21H20BrN3	394.31765	antiprotozoal, intercalcate with DNA	synthetic	INN, BAN	J Mol Biol 13: 269 (1965); 27: 87 (1967)	-0.24	0.58	1.57	0.31	0.56
01500645	PREGNENOLONE	100122-15	D10	sample	145-13-1, 4598-67-8	C21H32O2	316.48799	glucocortcoid, antiinflammatory	semisynthetic	INN, BAN		-0.33	0.46	0.97	-0.17	0.23
01504098	PHENOTHRIN	100122-15	D11	sample	26002-80-2	C23H26O3	350.46187	ectoparasiticide	synthetic; S-2539-F	INN, BAN		-0.64	0.51	2.58	0.44	0.72
	0.11% JAK2inhib	100122-15	D12	0.11% jak2inhib								-1.62	1.52	-2.89	-4.26	-1.81
	0.11% JAK2inhib	100122-15	E01	0.11% jak2inhib								-3.98	-3.55	-3.47	-3.58	-3.64
01500669	BENFLUOREX HYDROCHLORIDE	100122-15	E02	sample	23602-78-0	C19H21ClF3NO2	387.83292	antihyperlipoproteinemic	synthetic	INN, BAN		-0.99	0.30	0.26	-1.91	-0.58
01505180	6,2'-DIMETHOXYFLAVONE	100122-15	E03	sample		C17H14O4	282.29873		derivative	undetermined activity		2.77	0.20	-0.67	-0.02	0.57
01504116	FORMESTANE	100122-15	E04	sample	566-48-3	C20H28O2	300.44496	antineoplastic, aromatase inhibitor	synthetic; CGP-32349	INN, BAN	Steroids 65: 171 (2000)	-0.11	-0.04	-0.32	0.59	0.03
01505173	BAMBUTEROL HYDROCHLORIDE	100122-15	E05	sample	81732-46-9	C18H30ClN3O5	403.9099	beta adrenergic agonist, bronchodilator, cholinesterase inhibitor	synthetic	INN, BAN	Acta Anaeth Scand 34:595 (1990); Chest c103:771 (1993)	1.02	0.17	2.89	1.58	1.42
01501004	DROPROPIZINE	100122-15	E06	sample	17692-31-8	C13H20N2O2	236.31655	antitussive	synthetic; UCB-1967	INN, BAN		1.31	1.51	2.34	2.45	1.90
01503036	DINITOLMIDE	100122-15	E07	sample	148-01-6	C8H7N3O5	225.16209	antiprotozoal	synthetic	INN, BAN		0.47	-1.22	1.90	-1.00	0.04
01505245	TROXERUTIN	100122-15	E08	sample	7085-55-4	C33H42O19	742.69129	vasoprotectant	derivative; rutin 7,3',4'-trihydroxyethyl ether; Z-6000, THR	INN, BAN		0.39	0.21	1.52	1.07	0.80
01503053	PIPENZOLATE BROMIDE	100122-15	E09	sample	125-51-9, 13473-38-6	C22H28BrNO3	434.37736	spasmolytic	synthetic	INN, BAN		0.20	0.64	-0.97	1.22	0.28
01505323	CARBIMAZOLE	100122-15	E10	sample	22232-54-8	C7H10N2O2S	186.23395	antithyroid	synthetic	INN, BAN	Clin Endocrinol 10:655 (1979)	1.52	3.36	-0.23	-0.33	1.08
01503230	NIFENAZONE	100122-15	E11	sample	2139-47-1	C17H16N4O2	308.34267	analgesic, antiinflammatory	synthetic	INN, BAN		1.73	0.47	1.21	0.50	0.98
	0.11% DMSO	100122-15	E12	0.11% dmso								-0.39	-0.28	-0.15	0.23	-0.15
	0.11% JAK2inhib	100122-15	F01	0.11% jak2inhib								-3.82	-4.34	-4.39	-4.75	-4.32

												Screen Score (z-score)				
Compound ID	MolNAME	plate	position	Content	cas#	Formula	MolWt	Bioactivity	Source	Status	Reference	rep1	rep2	rep3	rep4	Average
01505355	LUFENURON	100122-15	F02	sample	103055-07-8	C17H8Cl2F8N2O3	511.15811	molt inhibitor, chitin synthesis inhibitor; insecticide	synthetic; CGA-184699	INN, BAN	Pakistan J Biol Sci 6:1125 (2003)	-5.29	-1.64	-2.27	-1.88	-2.77
01503231	NIMESULIDE	100122-15	F03	sample	51803-78-2	C13H12N2O5S	308.31499	antiinflammatory	synthetic; R-805	INN, BAN		-0.89	-0.31	-1.17	-1.45	-0.95
01505755	IDEBENONE	100122-15	F04	sample	58186-27-9	C19H30O5	338.44795	cognition enhancer, nootropic	synthetic	INN, BAN	Chem Pharm Bull 30:2797 (1982); Biochemistry 21:4096	0.11	1.04	-0.16	0.28	0.32
01505783	OXYPHENONIUM BROMIDE	100122-15	F05	sample	50-10-2	C21H34BrNO3	428.41403	anticholinergic, anticonvulsant	synthetic; BA-5473, C-5473	INN, BAN		-0.05	-0.09	2.67	-0.28	0.56
01503383	PEMPIDINE TARTRATE	100122-15	F06	sample	79-55-0	C14H27NO6	305.37439	ganglionic blocker, antihypertensive	synthetic	INN, BAN		0.67	0.71	-0.50	-0.35	0.13
01500634	IPRONIAZID SULFATE	100122-15	F07	sample	54-92-2, 305-33-9 [as	C9H15N3O5S	277.301	monoamine oxidase inhibitor, antidepressant	synthetic	INN, BAN		0.24	0.05	2.32	0.08	0.67
01503974	DIMETHYL 4,4-o-PHENYLENE-BIS (3-THIOPHANATE)	100122-15	F08	sample		C12H14N4O4S2	342.39778	antifungal (systemic plant)	synthetic	agricultural use		0.75	1.00	0.20	0.39	0.59
01504021	LUPININE	100122-15	F09	sample	486-70-4	C10H19NO	169.26903	antifeedant, antiinflammatory, oxytoxic	Anabasis aphylla, Lupinus spp	experimental	Helv Chim Acta 11:1062 (1928); JACS 56:2434 (1934)	-0.07	-0.53	0.09	0.02	-0.12
01504101	TETRACHLOROISOPHTHALONITRILE	100122-15	F10	sample		C8Cl4N2	265.9146	antifungal	synthetic	experimental		-1.93	-1.48	-1.58	-0.83	-1.45
00100568	SARMENTOSIDE B	100122-15	F11	sample		C34H48O13	664.75386		Strophanthus sarmentosis; mp 193-195	undetermined activity	Helv Chim Acta 31: 1719 (1948); 40: 980 (1957); 41: 736	0.74	1.74	-0.14	0.47	0.70
	0.04% DMSO	100122-15	F12	0.04% dmso								0.95	1.18	-0.13	-1.72	0.07
	0.04% JAK2inhib	100122-15	G01	0.04% jak2inhib								-2.28	-2.60	-2.89	-2.51	-2.57
01503206	CLOFOCTOL	100122-15	G02	sample	37693-01-9	C21H26Cl2O	365.34677	antibacterial	synthetic	INN		-0.57	0.42	-0.30	0.55	0.03
01300048	XYLOSE	100122-15	G03	sample	58-86-6	C5H10O5	150.13245	diagnostic aid	many woods, straw, corncobs, cottonseed hulls and pecan shells.	undetermined activity		0.40	-0.35	-1.86	-1.22	-0.76
01505786	TRICLABENDAZOLE	100122-15	G04	sample	68786-66-3	C14H9Cl3N2OS	359.66363	anthelmintic	synthetic	INN, BAN	Vet Parasitol 13:145 (1983); Vet Rec 113:315 (1983)	-0.06	1.75	-1.13	0.46	0.25
01502034	METAMPICILLIN SODIUM	100122-15	G05	sample	6489-97-0	C17H18N3NaO4S	383.40451	antibacterial	semisynthetic	INN		-0.33	0.55	-0.66	0.73	0.07
01506039	ETICLOPRIDE HYDROCHLORIDE	100122-15	G06	sample	97612-24-3, 84226-12-0 (base)	C17H26Cl2N2O3	377.31437	dopamine D2 blocker	synthetic	INN	Eur J Pharmacol 111:191 (1985); Physiol Behav 61:563	-0.11	0.32	-1.19	-1.39	-0.59
01600654	CHAULMOSULFONE	100122-15	G07	sample	473-32-5	C48H76N2O4S	777.21592	antilepretic	synthetic	INN		0.13	1.43	1.19	0.77	0.88
01504512	DEFERIPRONE	100122-15	G08	sample	30652-11-0	C7H9NO2	139.15528	iron chelating agent	synthetic	INN, BAN		-1.03	0.14	0.49	1.04	0.16
01506047	TIRATRICOL	100122-15	G09	sample	51-24-1	C14H9I3O4	621.93863	thyroid agent	synthetic	INN		2.32	-0.26	-0.16	0.14	0.51
01504504	CARSALAM	100122-15	G10	sample	2037-95-8	C8H5NO3	163.13395	analgesic	synthetic	INN, BAN		0.66	-0.58	-1.11	0.20	-0.21
01504501	beta-NAPHTHOL	100122-15	G11	sample	135-19-3	C10H8O	144.17466	anthelmintic, antiseptic	synthetic	INN, NF-XI	Austral J Chem 27:2385 (1974); Tetrahedron Letts 31:6977	0.63	0.72	0.30	-0.36	0.32
	0.04% DMSO	100122-15	G12	0.04% dmso								1.21	2.51	-0.09	0.02	0.91
	0.04% JAK2inhib	100122-15	H01	0.04% jak2inhib								-2.53	-2.52	-3.07	1.82	-1.57
01504511	CHLORALOSE	100122-15	H02	sample	158-79-93-3	C8H11Cl3O6	309.53227	anesthetic	synthetic	INN, DCF		-0.75	-0.09	-1.51	0.27	-0.52
01300099	CYSTINE	100122-15	H03	sample	56-89-3	C6H12N2O4S2	240.30154	nutrient	widespread in plants and animals	INN		1.04	-0.56	-1.81	-0.94	-0.57
01504073	UVAOL	100122-15	H04	sample	545-46-0	C30H50O2	442.7318	antineoplastic	Arctostaphylos spp, Leucothoe keiskei, Crataegus cuneata,	experimental	Bull Chem Soc Jpn 39:2313 (1966); J Pharm Sci 55:1378	-0.41	0.42	-1.81	1.51	-0.07
01506065	CLORGILINE HYDROCHLORIDE	100122-15	H05	sample	17780-72-2 (base)	C13H16Cl3NO	308.63757	MAO-A inhibitor, antidepressant, antiparkinsonian	synthetic	INN, BAN	J Neural Transm Suppl. 52:39 (1998)	-0.45	0.32	1.56	1.69	0.78
01504520	EPIESTRIOL	100122-15	H06	sample	547-81-9	C18H24O3	288.39018	estrogen	semisynthetic	INN, BAN		0.37	0.16	-0.38	0.40	0.14
01300018	GLUTAMINE (L)	100122-15	H07	sample	56-85-9	C5H10N2O3	146.14705	dietary suppliment	beetroot; widely distributed in plants	USP, INN		-0.96	1.05	0.00	-0.22	-0.03
01506027	OXALAMINE CITRATE	100122-15	H08	sample	959-14-8 (base)	C20H27N3O8	437.45349	antitussive	synthetic; SKF-9976, AF-438	INN		-0.01	-0.18	-0.37	-0.67	-0.31
01503100	URAPIDIL HYDROCHLORIDE	100122-15	H09	sample	34661-75-1	C20H30ClN5O3	423.9468	antihypertensive	synthetic	INN, BAN, JAN		0.48	3.05	0.26	1.08	1.21
01504506	TOSYLCHLORAMIDE SODIUM	100122-15	H10	sample	127-65-1	C7H7ClNNaO2S	227.64614	antiseptic, disinfestant, antiproliferative	synthetic	INN, BAN		0.50	-0.25	-0.06	-0.08	0.03
01504508	CINCHOPHEN	100122-15	H11	sample	132-60-5	C16H11NO2	249.27157	analgesic, antipyretic, antiinflammatory	synthetic	INN		1.23	0.18	-0.05	2.30	0.92
	empty	100122-15	H12	sample								NA	NA	NA	NA	NA
	empty	100122-16	A01	sample								NA	NA	NA	NA	NA
01504510	CHINIOFON	100122-16	A02	sample	8002-90-2	C9H5INNaO4S	373.1027	antiprotozoal, amebicide	synthetic	INN, DCF, NF-XI		-0.82	-0.82	-0.24	1.85	-0.01
01506031	PIRIBEDIL HYDROCHLORIDE	100122-16	A03	sample	3605-01-4	C16H18N4O2	298.34746	dopamine agonist	synthetic; EU-4200, ET-495	INN		-1.06	-0.70	-0.54	0.26	-0.51
01502015	NIFLUMIC ACID	100122-16	A04	sample	4394-00-7	C13H9F3N2O2	282.22408	analgesic, antiinflammatory	synthetic; UP-83	INN		1.27	-0.86	-0.77	-0.22	-0.15
01506077	METICRANE	100122-16	A05	sample	1084-65-7	C10H13NO4S2	275.34741	diuretic, antihypertensive	synthetic; SD-17102	INN, JAN		-1.78	-2.11	-1.85	-2.23	-1.99
01500938	PROTOVERATRINE B	100122-16	A06	sample	124-97-0	C41H63NO15	809.95696	antihypertensive, emetic; LD50(mouse) 0.21 mg/kg sc	Veratrum album, V viride	INN	J Biol Chem 149:271 (1943); JACS 82:2242, 2252 (1960);	0.12	-0.80	-0.13	5.76	1.24
01500618	ACRIFLAVINIUM HYDROCHLORIDE	100122-16	A07	sample	8018-07-3	C14H14ClN3	259.74078	antiinfective, intercalating agent	synthetic	NF, INN		1.20	0.53	1.27	-2.38	0.16
01505037	3-BROMO-4-METHYL-3,4-HEXAMETHYLENE-3,4-	100122-16	A08	sample		C7H11BrN2O2	235.08192	guanyl cyclase activator, NO donor, vasodilator	synthetic; DD2	experimental		0.30	0.77	0.84	-0.53	0.35
01503094	TIOXOLONE	100122-16	A09	sample	4991-65-5	C7H4O3S	168.17213	antiseborrhoic	synthetic	INN, BAN		0.26	2.39	-0.47	-0.14	0.51
00100566	DEOXYCHOLIC ACID	100122-16	A10	sample	88-44-3	C24H40O4	392.584		bile constituent	undetermined activity	J Biol Chem 238: 3846 (1963)	0.52	-0.15	-0.44	-0.87	-0.23
01504507	CHLORINDIONE	100122-16	A11	sample	1146-99-2	C15H9ClO2	256.69078	antcoagulant, Vitamine K antagonist	synthetic; G25766	INN, BAN		0.39	0.00	-1.15	2.88	0.53
	0.04% JAK2inhib	100122-16	A12	0.04% jak2inhib								-1.83	-0.70	-3.39	-2.44	-2.09
	0.04% DMSO	100122-16	B01	0.04% dmso								-1.43	-1.02	-1.39	2.76	-0.27
01503917	CLENBUTEROL HYDROCHLORIDE	100122-16	B02	sample	37148-27-9	C12H19Cl3N2O	313.65703	bronchodilator, beta2 adrenergic agonist	synthetic; NAB-365	INN, BAN, JAN		-1.01	-0.05	-1.44	-0.86	-0.84
01503606	CYPERMETHRIN	100122-16	B03	sample	52315-07-8	C22H19Cl2NO3	416.30763	insecticide	synthetic	BAN		0.45	-1.27	-1.35	-1.41	-0.90
01506079	MOXISYLYTE HYDROCHORIDE	100122-16	B04	sample	54-32-0	C16H25NO3	279.38255	alpha-adrenergic blocker	synthetic	INN, BAN, JAN	J Auton Nerv Syst 79: 191 (2000); hepatotoxic at clinical	-0.38	-1.67	-1.73	-1.26	-1.26
01900040	BARBITAL	100122-16	B05	sample	57-44-3	C8H12N2O3	184.19644	sedative	synthetic	INN	Therapeutische Monatsh 17:208 (1903)	-0.28	-0.88	-0.49	-0.62	-0.57
01506085	OXEDRINE	100122-16	B06	sample	94-07-5	C9H13NO2	167.20946	anti-obesity	citrus aurantium; SYNEPHRINE	BAN		-0.01	0.70	0.03	1.30	0.50
01506024	NAFTOPIDIL DIHYDROCHLORIDE	100122-16	B07	sample	57149-07-2	C24H30Cl2N2O3	465.4243	antihypertensive	synthetic	INN		0.57	0.41	0.31	0.21	0.38
01506056	ZARDAVERINE	100122-16	B08	sample	101975-10-4	C12H10F2N2O3	268.2219	PDE III & IV inhibitor, antiallergic	synthetic	INN	Eur Repir J 5:982 (1992); J. Pharmacol Exp Ther 270:250	0.54	0.10	-0.26	-1.24	-0.22
01505082	CYCLANDELATE	100122-16	B09	sample	456-59-7	C17H24O3	276.37903	vasodilator	synthetic; BS-572	INN, BAN, JAN	Arzneim Forsch 2:165 (1952); 3:503 (1953)	0.07	-0.33	1.80	-0.67	0.22
01505434	HALOTHANE	100122-16	B10	sample	151-67-7	C2HBrClF3	197.38247	anesthetic	synthesis	USP, INN, BAN, JAN		0.86	0.71	0.34	-0.21	0.43
01500647	VINCAMINE	100122-16	B11	sample	1617-90-9	C21H26N2O3	354.45297	vasodilator	Vinca minor	INN, BAN		0.22	1.23	0.48	-1.65	0.07
	0.04% JAK2inhib	100122-16	B12	0.04% jak2inhib								-1.22	-1.66	-2.55	-1.46	-1.72
	0.04% DMSO	100122-16	C01	0.04% dmso								-0.43	-1.37	-0.61	-0.18	-0.65
00270029	PRASTERONE ACETATE	100122-16	C02	sample	53-43-0 (praesterone)	C21H30O3	330.47145	adrenocortical hormone, antidepressant	semisynthetic	INN		-1.51	-0.78	-1.83	-1.17	-1.32
00212064	HYDROXYTOLUIC ACID	100122-16	C03	sample	83-40-9	C8H8O3	152.15116	analgesic, antiseptic	synthetic; 3-MS	INN, BAN		-0.62	0.01	-0.53	0.44	-0.18
00100424	XYLOCARPUS A	100122-16	C04	sample		C31H38O11	586.64191		Meliaceae spp	undetermined activity	Adv Med Phytochemistry 1986: 179	-3.33	-2.65	-4.47	-2.24	-3.17
01504517	DOXIFLURIDINE	100122-16	C05	sample	3094-09-5	C9H11FN2O5	246.19682	antineoplastic	synthetic	INN, JAN		0.01	3.40	2.00	-0.20	1.31
01504518	EFLOXATE	100122-16	C06	sample	119-41-5	C19H16O5	324.33637	coronary vasodilator	synthetic	INN, JAN		1.98	3.68	-0.04	3.02	2.16
01504502	BITOSCANATE	100122-16	C07	sample	4044-65-9	C8H4N2S2	192.26248	anthelmintic	synthetic; 16842	INN, MI		4.45	0.07	-0.03	2.29	1.70
01504515	DIACETAMATE	100122-16	C08	sample	2623-33-8	C10H11NO3	193.20407	analgesic, antiinflammatory	synthetic	INN, BAN		0.49	0.62	1.40	-1.19	0.33
00211175	METACETAMOL	100122-16	C09	sample	621-42-1	C8H9NO2	151.16643	analgesic	synthetic; BS-749	INN, BAN		1.03	0.93	1.51	5.18	2.16
00100447	DEACETOXY(7)-7-OXOKHIVORINIC ACID	100122-16	C10	sample		C27H36O10	520.58197		derivative	undetermined activity		-0.05	-0.36	-0.73	-1.19	-0.58
01503416	MIZORIBINE	100122-16	C11	sample	50924-49-7	C9H13N3O6	259.22046	immunosuppressant	synthetic	INN, JAN		0.62	0.72	4.16	-0.51	1.25
	0.11% JAK2inhib	100122-16	C12	0.11% jak2inhib								-0.67	-0.94	-3.76	-3.61	-2.24
	0.11% DMSO	100122-16	D01	0.11% dmso								1.94	-1.43	-1.38	-0.77	-0.41
00310050	QUININE ETHYL CARBONATE	100122-16	D02	sample	83-75-0	C23H28N2O4	396.49061	antimalarial	semisynthetic	JAN, NF-VIII		-1.80	-0.69	-1.62	-0.53	-1.16
01503200	CETRIMONIUM BROMIDE	100122-16	D03	sample	57-09-0, 6899-10-1	C19H42BrN	364.45729	antiinfectant	synthetic	NF, INN, BAN		1.16	1.48	1.16	-0.98	0.71
00100583	EUPHOL ACETATE	100122-16	D04	sample		C32H52O2	468.77004		Euphorbia spp	undetermined activity	Helv Chim Acta 37: 2306 (1954)	-4.29	-1.16	-0.54	-0.28	-1.57
00330008	2,4-DINITROPHENOL	100122-16	D05	sample	51-28-5	C6H4N2O5	184.10918	uncouples oxidative phosphorylation	synthetic	Martindale 28		-0.08	0.00	0.44	1.33	0.42
01300044	TARTARIC ACID	100122-16	D06	sample	87-69-4	C4H6O6	150.08882	pharmaceutical aid	common plant constituent	NF, JAN		0.14	2.00	-0.02	0.62	0.68
01505772	BUCETIN	100122-16	D07	sample	1083-57-4	C12H17NO3	223.27419	analgesic	synthetic	INN, BAN, JAN	Arnzneim Forsch 15:727 (1965); Chem Pharm Bull 16:1726,	0.06	0.69	2.09	0.38	0.80
01505310	REBAMIPIDE	100122-16	D08	sample	90098-04-7	C19H15ClN2O4	370.7954	antiulcer, antioxidant	synthetic	INN, JAN	Chem Pharm Bull39:2906 (1991); Dig Dis Sci 43:35S	0.15	1.27	0.68	-0.31	0.45
01300046	TYROSINE	100122-16	D09	sample	60-18-4	C9H11NO3	181.19292		widespread in nature	USP		1.03	-0.36	-0.27	-0.30	0.03
01504181	PRISTIMERIN	100122-16	D10	sample	1258-84-0	C30H40O4	464.6509	antineoplastic, antiinflammatory	Celastrus and Maytenus spp	experimental		-18.99	-4.61	-4.08	-0.96	-7.16
01505740	LIPOAMIDE	100122-16	D11	sample	3206-73-3	C8H15NOS2	205.34285	hepatoprotectant	semisynthetic; thioctic acid amide	JAN		0.07	-0.23	0.19	-0.93	-0.23
	0.11% JAK2inhib	100122-16	D12	0.11% jak2inhib								-2.40	-3.60	-3.64	-5.34	-3.74
	0.11% JAK2inhib	100122-16	E01	0.11% jak2inhib								-3.80	-3.58	-4.63	-3.06	-3.77
01502045	PIROMIDIC ACID	100122-16	E02	sample	19562-30-2	C14H16N4O3	288.30862	antibacterial	synthetic; PD-93	INN, JAN		-0.45	-0.69	3.13	-0.27	0.43
01503084	AMRINONE	100122-16	E03	sample	60719-84-8	C10H9N3O	187.20273	cardiac stimulant	synthetic	experimental		-0.16	-0.43	-0.24	0.28	-0.14
01505005	L-DEOXYALLIIN	100122-16	E04	sample	21593-77-1	C6H11NO2S	161.22407	antineoplastic	Allium spp	experimental	J Cancer Res 50:5084 (1990)	-1.42	0.00	0.62	0.59	-0.05
01506043	SOTALOL HYDROCHLORIDE	100122-16	E05	sample	959-24-0	C12H21ClN2O3S	308.82977	beta-adrenergic agonist	synthetic; MJ-1999	USP		-0.64	-0.64	0.54	0.91	0.04
01505688	PROPARACAINE HYDROCHLORIDE	100122-16	E06	sample	575-06-98	C16H27ClN2O3	330.85819	local anesthetic	synthetic	USP, INN, BAN		-0.85	0.17	0.51	3.78	0.90
01506064	CHLOROPYRAMINE HYDROCHLORIDE	100122-16	E07	sample	6170-42-9, 59-32-5 (base)	C16H21Cl2N3	326.27187	antihistamine	synthetic	INN, BAN		1.05	-0.71	0.09	1.23	0.41
01505315	6-AMINONICOTINAMIDE	100122-16	E08	sample	329-89-5	C6H7N3O	137.14219	antineoplastic, apoptosis inducer	synthetic	experimental	Biochem Pharmacol 50:1943 (1995); Cancer Chemother	1.06	1.67	2.38	0.38	1.37
01502102	QUINOLINIC ACID	100122-16	E09	sample		C7H5NO4	167.1222	differentiates between cerebral and forebrain NMDA receptors	synthetic	experimental		0.97	0.18	-0.63	1.04	0.39
01503205	CITIOLONE	100122-16	E10	sample	1195-16-0	C6H9NO2S	159.20813	hepatoprotectant	synthetic	INN		0.91	0.85	0.58	0.47	0.70
00100749	STROPHANTHIDINIC ACID LACTONE ACETATE	100122-16	E11	sample		C25H32O7	444.52959		semisynthetic	undetermined activity	J Biol Chem 64: 123; 65: 491 (1925)	-2.15	-2.65	-3.42	-3.33	-2.89
	0.11% DMSO	100122-16	E12	0.11% dmso								0.08	-0.25	-0.48	-1.18	-0.46
	0.11% JAK2inhib	100122-16	F01	0.11% jak2inhib								0.75	-3.61	-3.24	-1.33	-1.86
00270078	5alpha-CHOLESTANOL	100122-16	F02	sample	80-97-7	C27H48O	388.68301		eggs, faces, gall stones, adrenal tissue	undetermined activity		-3.56	-1.47	-0.93	-0.04	-1.50
01504912	OLSELTAMIVIR PHOSPHATE	100122-16	F03	sample	196618-13-0	C15H29N2O8P	396.38078	antiviral	synthetic	USAN	N Engl J Med 353:2667 (2005); Lancet 369:1056 (2007)	-6.06	-0.91	-1.15	2.72	-1.35
01300028	MANNITOL	100122-16	F04	sample	69-65-8	C6H14O6	182.17488	diuretic, sweetener, diagnostic aid	manna and seaweeds; exudates of olive and plane trees	USP		1.13	-0.54	1.10	0.78	0.62
00210242	THEAFLAVIN MONOGALLATES	100122-16	F05	sample		C36H28O16	716.61496		mixed isomers black tea (3' shown)	undetermined activity	Tet Lets 1970: 5237	0.08	-0.09	-0.15	1.72	0.39
01505762	PYRITINOL	100122-16	F06	sample	1098-97-1	C16H20N2O4S2	368.4768	cognition enhancer, nootropic	synthetic; pyrithioxin	INN, BAN, JAN	Chem Pharm Bull 25:1335 (1977; Arzniem) Forsch 19:5	0.32	-0.81	0.00	0.16	-0.08
01501111	PROTOPORPHYRIN IX	100122-16	F07	sample	553-12-8	C34H34N4O4	562.67448	hepatoprotectant	mamalian feces, avian pigment	JAN		-0.32	3.85	4.36	-0.56	1.84
01503973	2-THIOURACIL	100122-16	F08	sample	141-90-2	C4H4N2OS	128.15328	thyroid depressant	synthetic	BP-1948		-0.60	1.49	0.52	0.97	0.59
00100595	COTARNINE CHLORIDE	100122-16	F09	sample	10018-19-6, 82-54-2	C12H14ClNO3	255.70328	vasoconstrictor	Papaver pseudo-orientale	experimental	J Indian Chem Soc 12: 421 (1935); J Nat Prod 53:1302	-0.01	1.18	0.68	0.15	0.50
00100655	DIHYDROGEDUNIC ACID, METHYL ESTER	100122-16	F10	sample		C26H36O8	476.57202		derivative	undetermined activity		2.40	-1.00	0.28	-1.25	0.11
01505372	SUMATRIPTAN	100122-16	F11	sample	103628-46-2	C14H21N3O2S	295.40637	5HT agonist	synthetic; GR-43175	USAN, INN, BAN		0.91	0.40	1.83	1.63	1.19
	0.04% DMSO	100122-16	F12	0.04% dmso								1.62	1.09	-0.81	-0.38	0.38
	0.04% JAK2inhib	100122-16	G01	0.04% jak2inhib								-1.27	-0.28	-1.62	0.75	-0.61
01300043	SORBITOL	100122-16	G02	sample	50-70-4	C6H14O6	182.17488	sweetening agent and humectant	fruits of Sorbus and Crataegus spp	NF, JAN		-1.08	-0.23	1.59	-0.12	0.04
01503108	BROMOPRIDE	100122-16	G03	sample	4093-35-0	C14H22BrN3O2	344.25434	antiemetic	synthetic	INN		-1.09	-0.64	-1.77	0.09	-0.85
01505108	L-BUTHIONINE SULFOXIMINE	100122-16	G04	sample	83730-53-4	C8H18N2O3S	222.30826	inhibitor of gamma-glutamylcysteine synthetase	synthetic	experimental	J Biol Chem 254:7558 (1979); 257:13704 (1982); Bone	-0.45	1.25	1.69	3.11	1.40
01503074	ALEXIDINE HYDROCHLORIDE	100122-16	G05	sample	22573-93-9, 22782-69-0	C26H58Cl2N10	581.72516	antibacterial	synthetic	INN		-2.88	-5.07	-2.51	-1.49	-2.99
00240929	AVOCADYNE ACETATE	100122-16	G06	sample	24607-06-5	C19H34O4	326.48043	antifungal	Persea spp	experimental	Ann Acad Brasil Cienc 42 (suplemento): 45 (1970);	-0.05	0.35	2.36	-0.24	0.61
01500833	CANAVANINE	100122-16	G07	sample	543-38-4	C5H12N4O3	176.17639	NO synthase inhibitor	Canavalia ensiformis	experimental	Phytochemistry 6: 863 (1967); Eur J Pharmacol 269: 269;	0.12	-0.10	0.71	0.66	0.35
01502010	DIACERIN	100122-16	G08	sample	13739-02-1	C19H12O8	368.30269	antiinflammatory	synthetic	INN		-0.14	1.63	1.46	0.04	0.75
01401414	CRESOPIRINE	100122-16	G09	sample	4386-39-4	C10H10O4	194.1888	antiinflammatory, antpyretic	synthetic	INN		1.58	1.08	0.54	0.93	1.03
00380004	DIHYDROCELASTRYL DIACETATE	100122-16	G10	sample		C33H44O6	536.71503	chaperone stimulant	celastrol derivative	undetermined activity	J Org Chem 30: 1729 (1965)	-23.45	-1.52	-0.59	-1.41	-6.74
01504616	MEPIROXOL	100122-16	G11	sample	6968-72-5	C6H7NO2	125.12819	antihyperlipemic	synthetic	INN		0.11	2.04	0.91	0.73	0.95
	0.04% DMSO	100122-16	G12	0.04% dmso								0.97	0.80	-0.63	0.10	0.31
	0.04% JAK2inhib	100122-16	H01	0.04% jak2inhib								-3.34	-3.24	-2.28	0.92	-1.98
01300029	MEGLUMINE	100122-16	H02	sample	6284-40-8	C7H17NO5	195.21724	diagnostic aid	semisynthetic	USP, INN, BAN, JAN		-1.03	-0.98	-1.17	-0.30	-0.87
02300218	EFAROXAN HYDROCHLORIDE	100122-16	H03	sample	89197-32-0	C13H17ClN2O	252.74624	insulin secretagogue, alpha2 adrenorecetor antagonist	synthetic	INN, BAN	Naunyn Schmiedebergs Arch Pharmacol 359: 262 (1999)	-0.64	-0.46	-0.13	0.77	-0.11
01503044	CHROMOCARB	100122-16	H04	sample	4940-39-0	C10H6O4	190.15692	vascular protectant	synthetic	INN		-0.17	0.08	3.33	0.28	0.88
01502024	PIPEMIDIC ACID	100122-16	H05	sample	51940-44-4	C14H17N5O3	303.32329	antibacterial	synthetic	INN, BAN, JAN		-0.12	1.26	0.00	-0.06	0.27
01504505	CARZENIDE	100122-16	H06	sample	138-41-0	C7H7NO4S	201.20214	diuretic, carbonic anhydrase inhibitor	synthetic	INN		-0.01	1.50	-0.33	1.79	0.74
01504082	DIHYDROCELASTROL	100122-16	H07	sample		C29H40O4	452.63975		celastrol derivative	undetermined activity	J Org Chem 30: 1729 (1965)	-0.77	1.16	0.13	1.02	0.39
00100612	TOTAROL-19-CARBOXYLIC ACID, METHYL ESTER	100122-16	H08	sample		C21H30O3	330.47145		Podocarpus spp	undetermined activity	J Chem Soc 1963: 1553	1.55	0.50	-0.79	-1.03	0.06
00310016	CHAULMOOGRIC ACID	100122-16	H09	sample	502-30-7	C18H32O2	280.45454	antibacterial (mycobacteria)	Hydnocarpus and Oncola spp	experimental	J Chromatography 39: 182 (1969); Am Rev Respir Dis	-5.97	2.06	-0.42	0.20	-1.03

												Screen Score (z-score)				
Compound ID	MolNAME	plate	position	Content	cas#	Formula	MolWt	Bioactivity	Source	Status	Reference	rep1	rep2	rep3	rep4	Average
00201716	NORSTICTIC ACID	100122-16	H10	sample	571-67-5	C18H12O9	372.29094	antibacterial	Lobaria pulmonaria, Usnea japonica, Lecanora radiosa, Parmelia	experimental	Z Naturforsch 59c: 384 (2004)	1.02	1.07	1.36	0.53	0.99
00200413	2,6-DIMETHOXYQUINONE	100122-16	H11	sample	35069-70-6	C8H8O4	168.15056	antibacterial, induces dermatitis, mutagen	Picrasmus & Ailanthus spp	experimental		3.25	0.00	-0.21	-1.97	0.27
	empty	100122-16	H12	sample								NA	NA	NA	NA	NA
	empty	100122-17	A01	sample								NA	NA	NA	NA	NA
00201538	DECAHYDROGAMBOGIC ACID	100122-17	A02	sample		C38H54O8	638.84928		derivative	undetermined activity	Tetrahedron 21: 1453 (1965)	-5.44	-1.23	-3.08	-2.90	-3.16
00201595	OSAJIN	100122-17	A03	sample	482-53-1	C25H24O5	404.46703		Maclura pomifera	undetermined activity	J Am Chem Soc 63: 1253 (1941); 68: 406 (1946)	0.04	0.93	-0.87	-1.49	-0.35
00201605	BISANHYDRORUTILANTINONE	100122-17	A04	sample	749-18-8	C22H16O7	392.36862	antibacterial	Streptomyces spp; also rutilantinone	experimental	Naturwissenschaften 52: 539 (1965); Chem Ber 100: 256	-0.99	-0.82	-0.30	-1.03	-0.79
00205071	HAEMATOMMIC ACID, ETHYL ESTER	100122-17	A05	sample	39503-14-5	C11H12O5	224.21529		various lichens, e.g. Evernia spp, Parmelia spp	undetermined activity	J Org Chem 42: 2526 (1977); Helv Chim Acta 72: 1061	1.39	-0.67	-0.54	0.12	0.07
00201696	LANOSTEROL ACETATE	100122-17	A06	sample		C32H52O2	468.77004		derivative of lanosterol	undetermined activity	J Chem Soc 1953: 571	-7.93	-1.99	-0.78	-2.18	-3.22
01505746	7-AMINOCEPHALOSPORANIC ACID	100122-17	A07	sample	957-68-6	C10H12N2O5S	272.28154		derivative of Cephalosporin C	undetermined activity	Biochem J 79:48 (1961); JACS 84:3400 (1962)	0.74	-0.46	0.02	0.25	0.14
00201539	GARCINOLIC ACID	100122-17	A08	sample		C38H46O9	646.78492		Garcinia spp.; also hydrolysis product of gambogic acid	undetermined activity	Ann Chim 56: 232 (1966)	0.28	0.23	6.15	-0.53	1.53
00210369	GALLIC ACID	100122-17	A09	sample	149-91-7	C7H6O5	170.12287	antineoplastic, astringent, antibacterial	insect galls	INN, NF-VII	J Chem Soc 1961:1854; Agric Biol Chem 30:617 (1966);	0.66	-0.44	0.66	-0.65	0.06
00100006	BUSSEIN	100122-17	A10	sample	41060-14-4	C43H54O18	858.89903		Entandrophragma species	undetermined activity	Chem Commun 1965:302	-0.84	-0.11	-0.24	-2.29	-0.87
01502248	GLUTATHIONE	100122-17	A11	sample	70-18-8	C10H17N3O6S	307.32749	antioxidant	plant and animal tissue	JAN	Pharm Biol 11: 539 (1968)	1.14	-0.38	1.30	-0.20	0.47
	0.04% JAK2inhib	100122-17	A12	0.04% jak2inhib								-1.05	-1.69	-1.14	-2.24	-1.53
	0.04% DMSO	100122-17	B01	0.04% dmso								-1.08	-0.62	-1.38	-1.26	-1.08
00100049	KHAYANTHONE	100122-17	B02	sample	25279-68-9	C32H42O9	570.68614		Khaya species	undetermined activity	Chem Commun 1967:379	-8.16	-0.51	-2.26	1.51	-2.36
00200463	BRAZILEIN	100122-17	B03	sample	600-76-0	C16H12O5	284.27104		Caesalpinia spp	undetermined activity	Phytochemistry 46: 177 (1997)	0.12	0.40	-1.69	0.13	-0.26
00100008	CARAPIN	100122-17	B04	sample	3463-88-5	C27H32O7	468.55189		Carapa and Cedrela species; mp 180-185	undetermined activity	Chem Commun 1965: 302	-4.95	-4.19	-4.00	-1.81	-3.74
01502256	SHIKIMIC ACID	100122-17	B05	sample	138-59-0	C7H10O5	174.15475		common constituent in plants	undetermined activity	Helv Chim Acta 20: 705 (1937)	0.05	-0.13	1.50	-0.96	0.11
01504412	PODOPHYLLIN ACETATE	100122-17	B06	sample	1180-34-3	C24H24O9	456.45348		derivative	undetermined activity		-8.29	-7.27	-9.07	-8.36	-8.25
00300423	DIFUCOL HEXAMETHYL ETHER	100122-17	B07	sample	14262-07-8	C18H22O6	334.37244		derivative	undetermined activity	J Chem Soc 1969: 2403; Phytochemistry 31: 279	0.48	0.77	0.52	-0.09	0.42
00100005	ANTHOTHECOL	100122-17	B08	sample	10410-83-0	C28H32O7	480.56304		also as 11-ACETOXYCEDRELONE	undetermined activity	J Chem Soc 1963:983; 1970: 205; Nature 207:1101(1965)	-16.35	-24.62	-23.30	-13.70	-19.49
00100009	CEDRELONE	100122-17	B09	sample	1254-85-9	C26H30O5	422.526		Cedrela species	undetermined activity	J Chem Soc 1963: 2506, 2515	-21.11	-9.87	-6.22	-3.16	-10.09
00100303	ALLOPREGNANOLONE	100122-17	B10	sample		C21H34O2	318.50393		semisynthetic	undetermined activity	J Am Chem Soc 59: 616 (1937)	1.93	0.08	2.88	-0.22	1.17
00100012	DEACETYLGEDUNIN	100122-17	B11	sample		C26H32O6	440.54134		Khaya, Azadirachta and other West African timbers; mp 264-266	undetermined activity	J Chem Soc 1961: 3705; Phytochemistry 10: 1845 (1971)	-0.99	-0.77	0.04	1.21	-0.13
	0.04% JAK2inhib	100122-17	B12	0.04% jak2inhib								0.53	1.20	-0.25	-0.87	0.15
	0.04% DMSO	100122-17	C01	0.04% dmso								-1.23	0.82	-1.60	2.99	0.24
00100013	3-DEACETYLKHIVORIN	100122-17	C02	sample		C30H40O9	544.6479		Meliaceae spp	undetermined activity		-1.39	-0.69	-2.20	-0.04	-1.08
00100014	7-DEACETYLKHIVORIN	100122-17	C03	sample		C30H40O9	544.6479		Khaya species and other West African timbers	undetermined activity	J Chem Soc 1970: 1710	-0.38	1.37	-0.95	0.32	0.09
00100358	1,7-DIDEACETOXY-1,7-DIOXO-3-DEACETYLKHIVORIN	100122-17	C04	sample		C26H32O7	456.54074		Meliaceae spp	undetermined activity		0.31	0.81	0.74	-0.65	0.30
00100346	PICROTIN	100122-17	C05	sample	21416-53-5	C15H18O7	310.30651	GABAa receptor antagonist	nontoxin component of PICROTOXIN	experimental	J Chem Soc 1952: 1042; 1958: 2987	-0.09	0.54	0.41	-1.30	-0.11
00100222	3-DEOXO-3beta-HYDROXYMEXICANOLIDE 16-ENOL	100122-17	C06	sample		C28H36O7	484.59492		derivative	undetermined activity		-1.81	-1.73	-2.58	-1.36	-1.87
00100173	EPOXYGEDUNIN	100122-17	C07	sample		C28H34O8	498.57838		Meliaceae spp	undetermined activity		-2.67	-3.34	-2.35	3.33	-1.26
00100101	PRENYLETIN	100122-17	C08	sample	15870-91-4	C14H14O4	246.26528		Ptaeroxylon obliquum	undetermined activity	J Chem Soc (C) 1967:145; Tet Lett 1967:2147	0.99	-1.31	0.53	0.27	0.12
00100060	MEXICANOLIDE	100122-17	C09	sample	1915-67-9	C27H32O7	468.55189		Meliaceae spp	undetermined activity	Tetrahedron 24: 1489 (1968)	0.47	1.25	1.40	0.37	0.87
00100315	TIGOGENIN	100122-17	C10	sample	77-60-1	C27H44O3	416.64993		Agavaceae, Dioscoreaceae, Solanaceae, Scrophulariaceae,	undetermined activity	Chem Ind 1954:1320	2.26	-0.70	0.80	-0.55	0.45
00100513	PTAEROXYLIN	100122-17	C11	sample	14729-11-4	C15H14O4	258.27643		Ptaeroxylon obliquum, Cedrelopsis grevei	undetermined activity	J Chem Soc (C) 1966:114; 1967:145; Tet Lett 1967:3459	0.37	-0.13	2.94	1.06	1.06
	0.11% JAK2inhib	100122-17	C12	0.11% jak2inhib								-2.28	-3.35	-1.16	-1.35	-2.03
	0.11% DMSO	100122-17	D01	0.11% dmso								-1.77	-1.10	-1.21	-1.38	-1.37
00100223	1,2alpha-EPOXYDEACETOXYDIHYDROGEDUNIN	100122-17	D02	sample		C26H32O7	456.54074		Meliaceae spp	undetermined activity		-6.80	-1.65	-0.16	-1.73	-2.59
00100105	8beta-HYDROXYCARAPIN, 3,8-HEMIACETAL	100122-17	D03	sample		C27H32O8	484.55129		derivative	undetermined activity		-0.20	0.33	0.97	0.35	0.36
00100359	3,16-DIDEOXYMEXICANOLIDE-3beta-DIOL	100122-17	D04	sample		C27H36O7	472.58377		derivative	undetermined activity	Taylor (unpublished)	0.04	1.95	-0.91	1.19	0.57
00100267	TOTAROL ACETATE	100122-17	D05	sample		C22H32O2	328.49914		Podocarpus totara	undetermined activity	J Chem Soc 1951: 2979	0.14	0.15	2.80	0.11	0.80
00100117	HYDROLYSIS PRODUCT OF BUSSEIN	100122-17	D06	sample		C32H40O14	648.6672		structure tentative	undetermined activity		1.92	1.01	0.05	-0.79	0.55
00100550	OLEANOIC ACID	100122-17	D07	sample	508-02-1	C30H48O3	456.71526		leaves of Olea europea and Viscum album L.	undetermined activity	J Chem Soc (C) 1939: 1047	0.69	0.25	0.07	1.94	0.74
00100520	LUNARINE	100122-17	D08	sample	24185-51-1	C25H31N3O4	437.54352		Lunaria spp	undetermined activity	Bull Soc Chim France 1956:1840; Naturwissenschaften	-0.61	2.06	0.42	-0.01	0.47
00100551	FRIEDELIN	100122-17	D09	sample	559-74-0	C30H50O	426.7324		Ceratopetalum apetalum D. Don, Cunoniaceae	undetermined activity	J Chem Soc 1954: 473	0.51	1.26	0.57	0.33	0.67
00100096	3-DEOXY-3beta-HYDROXYANGOLENSIC ACID METHYL	100122-17	D10	sample		C27H36O7	472.58377		Meliaceae spp	undetermined activity		3.19	-0.94	-0.42	0.02	0.46
00100081	UTILIN	100122-17	D11	sample	31218-22-1	C41H52O17	816.86139		Entandrophragma utile	undetermined activity	J Chem Soc 1960:3827; Chem Commun 1970:1388	-0.04	-1.52	-1.40	-0.59	-0.89
	0.11% JAK2inhib	100122-17	D12	0.11% jak2inhib								0.15	-2.77	-2.08	-2.59	-1.82
	0.11% JAK2inhib	100122-17	E01	0.11% jak2inhib								-3.55	-2.58	-0.17	-1.87	-2.04
00100201	3-DEOXO-3beta-ACETOXYDEOXYDIHYDROGEDUNIN	100122-17	E02	sample		C30H40O7	512.6491		Meliaceae spp	undetermined activity		0.54	-0.24	-0.12	2.95	0.79
00100455	MEROGEDUNIN	100122-17	E03	sample		C21H28O4	344.45491		derivative	undetermined activity	J Chem Soc 1966: 506	-0.74	1.69	-0.23	-0.64	0.02
00100517	ENTANDROPHRAGMIN	100122-17	E04	sample	11013-05-1	C43H56O17	844.91557		Entangrophragma spp.	undetermined activity	J. Chem. Res. Synop. 1977: 154	1.62	0.38	1.87	-0.51	0.84
00100355	1,3-DIDEACETYL-7-DEACETOXY-7-OXOKHIVORIN	100122-17	E05	sample		C26H34O7	458.55668		Meliaceae spp	undetermined activity		-2.53	-2.20	-2.98	-1.07	-2.19
00100497	3beta-ACETOXYDEOXODIHYDROGEDUNIN	100122-17	E06	sample		C30H40O8	528.6485		Meliaceae spp	undetermined activity		-8.19	-2.99	-1.93	-1.18	-3.57
00100305	3beta-HYDROXYISOALLOSPIROST-9(11)-ENE	100122-17	E07	sample		C27H42O3	414.63399		semisynthetic	undetermined activity		-0.44	-0.14	-0.80	1.57	0.05
01505959	DIPTERYXIN	100122-17	E08	sample	53948-01-9	C17H14O6	314.29753		Dipteryx odorata	undetermined activity	Phytochemistry 13:1943 (1974)	0.79	0.58	-0.47	0.78	0.42
00100286	PODOTOTARIN	100122-17	E09	sample		C40H58O2	570.90706		Podocarpus spp	undetermined activity	Tetrahedron 19: 209 (1963); Phytochemistry 6: 883 (1967)	-3.50	0.56	0.48	1.46	-0.25
00100360	beta-AMYRIN	100122-17	E10	sample	559-70-6	C30H50O	426.7324		widespread in plants	undetermined activity	Phytochemistry 9: 1669 (1970)	-3.52	-0.32	0.64	1.16	-0.51
00100129	8-HYDROXYCARAPINIC ACID	100122-17	E11	sample		C26H30O8	470.5242		Meliaceae spp	undetermined activity		1.01	-0.59	2.23	3.98	1.66
	0.11% DMSO	100122-17	E12	0.11% dmso								-0.35	0.58	0.49	0.39	0.28
	0.11% JAK2inhib	100122-17	F01	0.11% jak2inhib								-2.67	-3.09	-3.10	-3.24	-3.02
00100024	DIHYDROGEDUNIN	100122-17	F02	sample		C28H36O7	484.59492		Meliaceae spp.	undetermined activity		-2.11	-3.36	-1.89	-3.29	-2.66
00100287	TOTAROL	100122-17	F03	sample	511-15-9	C20H30O	286.4615		Podocarpus spp, Dacrydium cupressinum, Tetraclinis articulata	undetermined activity	Aust J Chem 48: 883 (1995)	-1.88	-0.42	-1.99	-1.53	-1.45
00100031	FISSINOLIDE	100122-17	F04	sample	1915-69-1	C29H36O8	512.60547		Cedrela fissilis, Khaya grandifoliola	undetermined activity	Tetrahedron 26: 219 (1970); Tet Letters 1970: 2797; J Chem	0.47	0.67	-0.13	1.86	0.72
00100432	DEOXYGEDUNIN	100122-17	F05	sample		C28H34O6	466.57958		Meliaceae spp	undetermined activity		-0.61	0.76	1.09	-0.22	0.26
00100139	DEOXYKHIVORIN	100122-17	F06	sample		C32H42O9	570.68614		Meliaceae spp	undetermined activity		-1.65	-3.20	-3.28	0.59	-1.89
00100298	SMILAGENIN ACETATE	100122-17	F07	sample		C29H46O4	458.68757		Smilax ornata, Agave & Yucca spp	undetermined activity		-6.57	-0.48	-1.16	-0.94	-2.29
00100047	DEACETOXY-7-OXOGEDUNIN	100122-17	F08	sample		C26H30O6	438.5254		Meliaceae spp	undetermined activity		2.86	2.84	1.13	-0.10	1.68
00100162	KHIVORIN	100122-17	F09	sample	2524-38-1	C32H42O10	586.68554		Khaya and other West African timbers	undetermined activity	J Chem Soc 1962: 768	0.06	-1.46	-1.85	-1.56	-1.20
01504524	ORNITHINE	100122-17	F10	sample	70-26-8	C5H12N2O2	132.16359	hepatoprotectant, anticholesteremic	widespread in nature	INN		1.11	0.87	0.40	0.37	0.69
01505484	LUPEOL	100122-17	F11	sample	545-47-1	C30H50O	426.7324	antineoplastic	widespread in plants	experimental	Bull Soc Chim Belg 70:585 (1961)	-5.92	4.21	0.73	1.16	0.04
	0.04% DMSO	100122-17	F12	0.04% dmso								2.12	-0.87	0.59	-0.18	0.41
	0.04% JAK2inhib	100122-17	G01	0.04% jak2inhib								-1.00	-1.23	-2.44	-2.10	-1.69
01500733	ALPINETIN METHYL ETHER	100122-17	G02	sample	36052-66-1	C17H16O4	284.31467		Eucalyptus spp	undetermined activity		-0.52	-0.30	-0.35	1.01	-0.04
01505488	SALIDROSIDE	100122-17	G03	sample	10338-51-9	C15H22O7	314.33839		Salix triandra, Rhodiola spp	undetermined activity	Naturwissenschaften 51:360 (1964)	3.13	1.44	0.89	-0.22	1.31
01400242	N-METHYLBENZYLAMINE HYDROCHLORIDE	100122-17	G04	sample	13426-94-3, 103-67-3(base)	C8H12ClN	157.64454		Ephedra sp.	undetermined activity		-0.72	-0.09	1.06	0.62	0.22
01504036	3-DESHYDROXYSAPPANOL TRIMETHYL ETHER	100122-17	G05	sample		C19H22O5	330.38419		derivative	undetermined activity		-0.52	0.94	-0.31	0.02	0.03
01504030	beta-ESCIN	100122-17	G06	sample	6805-41-0	C55H86O24	1131.28427	membrane permeabilizer	principal saponin Aesculus hippocastanum	INN	Arzneimittel-Forsch 13: 385 (1963)	-1.25	2.97	0.12	2.73	1.14
01500741	6,4'-DIMETHOXYFLAVONE	100122-17	G07	sample	54401-47-7	C17H14O4	282.29873		derivative Cassia spectabilis	undetermined activity		0.05	0.07	0.72	0.70	0.38
01504120	CARNOSIC ACID	100122-17	G08	sample	3650-09-7	C20H28O4	332.44376		Salvia spp, Rosmarinus officinalis	undetermined activity		0.39	0.38	-0.23	0.81	0.34
01504206	BOVINOCIDIN (3-nitropropionic acid)	100122-17	G09	sample	504-88-1	C3H5NO4	119.0776	antineoplastic	Aspergillus, Streptomyces spp & other microorganisms	experimental	Nature 164: 575 (1949)	0.87	1.37	1.02	1.65	1.23
01500709	CHRYSIN	100122-17	G10	sample	480-40-0	C15H10O4	254.24455	diuretic	Ulnus sieboldiana, Flourensia resinosa, Oroxylum indicum, Pinus	experimental	J Chem Soc 1938:1320; Biochem J 59:58 (1955)	1.02	1.27	2.70	0.47	1.37
01503006	BENZYL ISOTHIOCYANATE	100122-17	G11	sample	622-78-6	C8H7NS	149.21569	antineoplastic, antibacterial, antifungal	Tropaeolum majus, Lepidium sativum and other Cruciferae	INN	J Agr Food Chem 12: 127 ((1964)	0.14	0.50	0.06	2.34	0.76
	0.04% DMSO	100122-17	G12	0.04% dmso								0.33	2.52	0.86	-0.39	0.83
	0.04% JAK2inhib	100122-17	H01	0.04% jak2inhib								-4.02	-2.08	-2.64	-3.00	-2.94
01503042	ARTEMISININ	100122-17	H02	sample	63968-64-9	C15H22O5	282.33959	antimalarial	Artemisia annua	INN	Science 228:1049 (1985); Drugs 52:818 (1996); Trends	-2.06	0.06	-0.40	0.01	-0.60
01500721	7,4'-DIHYDROXYFLAVONE	100122-17	H03	sample	2196-14-7	C15H10O4	254.24455	antioxidant	Pterocarpus marsupium	experimental	Phytochemistry 4: 523 (1965); J Nat Prod 47:179 (1984)	-0.36	2.50	-0.31	-0.10	0.43
01500758	LOBELINE HYDROCHLORIDE	100122-17	H04	sample	90-69-7	C22H28ClNO2	373.92696	antiasthmatic, respiratory stimulant	Lobelia spp	INN, BAN, JAN		-0.55	-0.54	-0.02	-0.18	-0.32
01505349	4-O-METHYLPHLORACETOPHENONE	100122-17	H05	sample	7507-89-3	C9H10O4	182.17765	antifungal	Prunus domesticus	experimental		-0.75	-0.06	-0.78	-0.06	-0.41
01500706	ARCAINE SULFATE	100122-17	H06	sample	36587-93-6	C6H18N6O4S	270.31216		Arca noae	undetermined activity	Chem Abstracts 41: 4455 (1947)	-0.33	0.48	-0.80	0.81	0.04
01505274	3,4-DIHYDROXYCARANE	100122-17	H07	sample		C10H18O2	170.25376		monoterpene	undetermined activity		0.16	1.37	0.53	0.05	0.53
01700330	MUNDULONE ACETATE	100122-17	H08	sample		C28H28O7	476.53116		derivative	undetermined activity		5.82	7.49	8.19	8.93	7.61
01500656	AJMALINE	100122-17	H09	sample	4360-17-7	C20H26N2O2	326.44242	antiarrhythmic (Class Ia): inhibits glucose uptake by mitochondria,	Rauwolfia spp., Melodinus balansae, Tonduzia longifolia	JAN	J Chem Soc 1954: 1242; Angew Chem., 68: 13 (1956)	0.09	2.54	0.08	0.69	0.85
01503219	DIOSMIN	100122-17	H10	sample	520-27-4	C28H32O15	608.55824	vascular protectant	Zanthoxylum avicennae	INN, BAN		0.95	0.71	0.10	-0.25	0.38
01500760	HECOGENIN	100122-17	H11	sample	467-55-0	C27H42O4	430.63339	antiinflammatory	Agave and Yucca spp	experimental	Planta Med 63:199 (1997)	0.89	-0.23	1.02	-0.33	0.34
	empty	100122-17	H12	sample								NA	NA	NA	NA	NA
	empty	100122-18	A01	sample								NA	NA	NA	NA	NA
01500872	PALMATINE CHLORIDE	100122-18	A02	sample	10605-02-4	C21H22ClNO4	387.86679	antibacterial, antimalarial, uterine contractant	Jateorhiza palmata (Calumba root)	experimental	J Nat Prod 28: 73 (1965); Can J Chem 58: 2770 (1980)	1.04	1.08	-1.65	1.09	0.39
01505812	CRYPTOTANSHINONE	100122-18	A03	sample	35825-57-1	C19H20O3	296.36945	inhibits angiogenesis	Salvia miltiorrhiza, Rosmarinus officinalis	experimental	Chem Pharm Bull 10:112 (1962); Planta Med 24:190	-0.66	-1.55	-2.91	-1.75	-1.72
01500754	XANTHURENIC ACID	100122-18	A04	sample	59-00-7	C10H7NO4	205.17159	caspase activator, guanylyl cyclase stimulant	pathological metabolite of tryptophan and kynurenine	experimental	Mol Microbiol 42:553 (2001); BMC Physiology 1:7 (2001)	0.05	-1.58	-1.77	-2.05	-1.34
01505177	RUBESCENSIN A	100122-18	A05	sample	28957-04-2	C20H28O6	364.44256	antibacterial, antineoplastic, insect growth inhibitor	Isodon trichocarpus, I japonicus, Rabdosia spp	experimental	Chem Pharm Bull 18:871 (1970); 20:1752 (1972); 24:2118	-1.77	-1.24	-1.35	4.38	0.00
01501017	FERULIC ACID	100122-18	A06	sample	1135-24-6	C10H10O4	194.1888	antineoplastic, choleretic, food preservative	widely distributed in plants	experimental	J Org Chem 16: 216 (1951)	-1.62	-1.32	-0.68	-2.48	-1.52
01504019	GOSSYPOL	100122-18	A07	sample	303-45-7	C30H30O8	518.5688	antispermatogenic, antineoplastic, antiHIV	Gossypium spp	experimental	J Am Chem Soc 60: 2193 (1938)	2.41	0.99	0.52	0.28	1.05
01500811	BERBERINE CHLORIDE	100122-18	A08	sample	633-65-8, 2086-83-1	C20H18ClNO4	371.82376	antiarrhythmic, alpha2 agonist, cholinesterase, anticonvulsant,	Berberis and Mahonia spp	JAN	Coll Czech Chem Comm 35:124 (1970); J Nat Prod 40:384	-1.31	-2.72	-2.76	-4.18	-2.74
01503982	AGMATINE SULFATE	100122-18	A09	sample	2482-00-0	C5H16N4O4S	228.27167	NMDA blocker, alpha-2 adrenergic agonist; NO synthase inhibitor	Ambrosia entemisifolia	experimental	JACS 41:681 (1919); Science 263:966 (1994); Biochem J	-0.31	0.75	0.16	1.07	0.42
01503602	SALINOMYCIN, SODIUM	100122-18	A10	sample	53003-10-4	C42H69NaO11	773.00143	antibacterial	Streptomyces albus	INN, BAN	J Antibiotics 27: 814 (1954)	-3.33	-2.60	0.14	-3.28	-2.27
01505038	KASUGAMYCIN HYDROCHLORIDE	100122-18	A11	sample	19408-46-9, 6980-18-3 (base)	C14H26ClN3O9	415.83102	antifungal	Streptomyces kasugaensis	agricultural use	J Antibiot Ser A 18:101 (1965); Tet Lett 1966:1239	-0.01	0.66	-1.31	-3.09	-0.93
	0.04% JAK2inhib	100122-18	A12	0.04% jak2inhib								-1.66	-0.79	-0.69	-2.13	-1.32
	0.04% DMSO	100122-18	B01	0.04% dmso								0.86	-1.11	-1.33	2.51	0.23
01505920	PANTETHINE	100122-18	B02	sample	16816-67-4	C22H42N4O8S2	554.73004	antilipemic	semisynthetic	JAN	J Chem Soc 1954:1171; Atherosclerosis 50:73 (1984)	-0.18	-0.82	0.02	-1.80	-0.70
01500944	CARNOSINE	100122-18	B03	sample	305-84-0	C9H14N4O3	226.23693		mammalian skeletal muscle	undetermined activity	Science 184: 909 (1974); Proc Soc Exp Biol Med 158: 402	2.75	-0.33	1.82	0.26	1.13
01500987	GLUTAMINE (D)	100122-18	B04	sample	6899-04-3	C5H10N2O3	146.14705		widely distributed in plants	undetermined activity		1.12	-0.20	-0.07	-0.66	0.05
01502227	CADAVERINE TARTRATE	100122-18	B05	sample	462-94-2(base)	C7H16N2O6	224.21537		putrifaction of lysine; sectrtion of the fox Vulpes vulpes	undetermined activity		-0.73	-0.49	-0.58	-2.25	-1.01
01502245	ELLAGIC ACID	100122-18	B06	sample	476-66-4	C14H6O8	302.19912	hemostatic, antineoplastic, antimutagenic	widely distributed in higher plants	INN		1.47	2.03	1.83	-0.12	1.31
01502232	CAMPTOTHECIN	100122-18	B07	sample	7689-03-4	C20H16N2O4	348.36152	antineoplastic	Camptotheca acuminata	experimental	J Am Chem Soc 88: 3888 (1966)	0.10	2.14	1.69	1.47	1.35
01504002	BAICALEIN	100122-18	B08	sample	491-67-8	C15H10O5	270.24395	antiviral (HIV)	Scutellaria baicalensis	experimental	Gazz Chim acta 49 (II): 47 (1919); Acta Phytochim 1: 109	-1.53	4.84	-0.65	-0.99	0.42
01500819	BERGENIN	100122-18	B09	sample	477-90-7	C14H18O10	346.29356	hepatoprotectant	Bergenia spp.	JAN	J Chem Soc 1958: 2231; Org Biomol Chem. 6:2713 (2008)	0.17	0.92	1.05	-0.03	0.53
01500906	LITHOCHOLIC ACID	100122-18	B10	sample	434-13-9	C24H40O3	376.5846	LD50(mouse) 3900 mg/kg po	mammalian bile and gallstones, faecal matter	experimental	JACS 71:4126 (1949)	-4.92	0.16	2.45	-0.82	-0.78
01500901	AESCULIN	100122-18	B11	sample	531-75-9	C15H16O9	340.28937	antiinflammatory	Aesculus hippocastanum, Fraxinus spp	experimental	Naturwiss 29: 650 (1941); Phytochemistry 11: 657 (1972)	-0.35	-0.03	-1.60	-1.13	-0.78
	0.04% JAK2inhib	100122-18	B12	0.04% jak2inhib								0.93	0.93	2.45	-1.18	0.78
	0.04% DMSO	100122-18	C01	0.04% dmso								0.60	-1.46	1.58	-0.54	0.04
01501204	LAPACHOL	100122-18	C02	sample	84-79-7	C15H14O3	242.27703	antineoplastic, antifungal	heartwood of Bignoniaceae	experimental	Phytochemistry 14: 1829 (1975)	0.43	-0.78	-0.62	0.06	-0.23
01503904	PATULIN	100122-18	C03	sample	149-29-1	C7H6O4	154.12347	antibacterial	Aspergillus clavatus, Penicillium patulum	experimental	J Chem Soc 1944: 415	-13.10	-12.18	-8.02	-5.32	-9.65
01500990	ENOXOLONE	100122-18	C04	sample	471-53-4	C30H46O4	470.69872	antitussive, antiinflammatory, antibacterial	derivative	INN, BAN, JAN		-2.20	-1.17	-0.44	-0.09	-0.98
01505824	TANSHINONE IIA	100122-18	C05	sample	568-72-9	C19H18O3	294.35351	antineoplastic, bone resorption inhibitor, antiproliferative, apoptosis	Salvia miltiorrhiza	experimental	Chem Pharm Bull 10:112 (1962); Phytochemistry 24:815	-0.75	-1.46	-2.50	-2.00	-1.67

														Screen Score (z-score)		
**Compound ID**	**MolNAME**	**plate**	**position**	**Content**	**cas#**	**Formula**	**MolWt**	**Bioactivity**	**Source**	**Status**	**Reference**	**rep1**	**rep2**	**rep3**	**rep4**	**Average**
01505810	PEONIFLORIN	100122-18	C06	sample	23180-57-6	C23H30O11	482.48895	antiinflammatory, antispasmodic, antihypertensive, antidiuretic	Peonia spp	experimental	Tetrahedron 25:1825 (1969)	-1.24	-0.67	1.09	0.05	-0.19
00100146	7-DESACETOXY-6,7-DEHYDROGEDUNIN	100122-18	C07	sample		C26H30O5	422.526		derivative of gedunin	undetermined activity		-0.60	-0.04	-0.91	-0.49	-0.51
01500867	HARMINE	100122-18	C08	sample	442-51-3	C13H12N2O	212.25339	antiparkinsonian, CNS stimulant	Peganium harmala	experimental	Phytochemistry 7: 503 (1968)	4.31	3.06	3.47	2.80	3.41
00100102	CARAPIN-8(9)-ENE	100122-18	C09	sample		C27H30O7	466.53595		Carapa and Cedrela species	undetermined activity	Chem Commun 1965: 302	5.05	6.11	5.60	1.38	4.54
01504525	OROTIC ACID	100122-18	C10	sample	65-86-1	C5H4N2O4	156.09863	hepatoprotectant, uricosuric agent	widespread in animals	INN	J Biol Chem 202:91 (1953)	0.16	4.13	3.05	-0.65	1.67
00300047	ANISODAMINE HYDROBROMIDE	100122-18	C11	sample	17659-49-3	C17H23NO4	305.37716	anticholinergic, antispasmodic	Scopolia tanguticus	CHINA	Naturwissenschaften 49: 281 (1962)	0.05	2.85	0.34	0.88	1.03
	0.11% JAK2inhib	100122-18	C12	0.11% jak2inhib								-3.22	-2.23	-1.75	-2.48	-2.42
	0.11% DMSO	100122-18	D01	0.11% dmso								-0.49	-2.96	-0.95	-2.49	-1.72
01503630	SPAGLUMIC ACID	100122-18	D02	sample	4910-46-7	C11H16N2O8	304.25877	neurotransmitter; mGluR3 receptors	brain tissue	INN	PNAS 80:1116 (1983); Eur J Pharmacol 98:193 (1984); J	1.61	-0.57	-0.99	-1.35	-0.32
00201508	7-DESHYDROXYPYROGALLIN-4-CARBOXYLIC ACID	100122-18	D03	sample		C12H8O6	248.19396		synthetic	undetermined activity		1.15	-1.35	-0.36	-1.48	-0.51
01506011	6-METHOXYHARMALAN	100122-18	D04	sample	3589-73-9	C13H14N2O	214.26933	convulsant, halucinogen	Peganum spp	experimental	Int J Biochem 14:581 (1982); Neurosci Let 189:121 (1995);	0.79	0.85	0.48	1.01	0.78
00107113	ANDROSTERONE ACETATE	100122-18	D05	sample	1164-95-0	C21H32O3	332.48739	androgen	semisynthetic	experimental		0.26	0.20	-0.29	1.52	0.42
00270049	alpha-HYDROXYDEOXYCHOLIC ACID	100122-18	D06	sample	83-49-8	C24H40O4	392.584		pig bile	undetermined activity	J Chem Soc 1990: 1	0.01	0.30	-0.02	1.22	0.38
10100003	BIOCHANIN A	100122-18	D07	sample	491-80-5	C16H12O5	284.27104	phytoestrogen	widely distributed in Leguminosae	experimental	J Chem soc 1953: 1852	0.94	-0.25	0.45	0.02	0.29
00270051	METHYL DEOXYCHOLATE	100122-18	D08	sample	3245-38-3	C25H42O4	406.61109		rabbit bile & feces	undetermined activity	Helv Chim Acta 25:797 (1942); J Biol Chem 238: 3846	-2.29	-2.50	-1.20	-0.49	-1.62
00300038	JUGLONE	100122-18	D09	sample	481-39-0	C10H6O3	174.15752	antineoplastic, antifungal	leaves and nuts of Juglans spp., Carya spp and Pterocarya spp.	experimental		-3.48	0.37	1.12	1.74	-0.06
01504051	STIGMASTEROL	100122-18	D10	sample		C29H48O	412.70531		soya and calabar beans; widely distributed in plant oils	undetermined activity		-4.79	-0.36	1.08	-0.06	-1.03
01506004	LACTOBIONIC ACID	100122-18	D11	sample	96-82-2	C12H22O12	358.30194	food additive	semisynthetic	undetermined activity		-0.29	4.84	-0.06	0.08	1.14
	0.11% JAK2inhib	100122-18	D12	0.11% jak2inhib								-3.57	-1.47	1.54	-2.33	-1.46
	0.11% JAK2inhib	100122-18	E01	0.11% jak2inhib								-2.71	-4.03	-4.01	-1.51	-3.07
01600025	2,6-DIHYDROXY-4-METHOXYTOLUENE	100122-18	E02	sample		C8H10O3	154.1671		synthetic	undetermined activity		0.54	1.94	-0.15	0.33	0.66
01600075	QUERCETIN PENTAMETHYL ETHER	100122-18	E03	sample		C20H20O7	372.3782		derivative	undetermined activity		-0.54	-1.07	-0.34	-0.44	-0.60
01600919	3-METHOXYCATECHOL	100122-18	E04	sample	934-00-9	C7H8O3	140.14001		Machaerium kuhlmanni	undetermined activity	Phytochemistry 17: 1383, 1401 (1990)	0.18	-0.49	-1.48	0.60	-0.30
01506091	MENAQUINONE-4	100122-18	E05	sample		C21H24O2	308.42423	antioxidant, alkaline phosphatase enhancer	synthetic; VITAMIN K2	experimental	Biochem J 162:297 (1977); J Vasc Res 40:531 (2003)	-4.62	-0.71	-0.30	0.36	-1.32
01505896	GRAMINE	100122-18	E06	sample	87-52-5	C11H14N2	174.24763		Arundo, Hordeum, Phalaris spp	experimental	J Org Chem 24:1285 (1959); Phytochemistry 33:741 (1993)	0.25	0.37	0.62	-0.49	0.19
01505884	ESEROLINE FUMARATE	100122-18	E07	sample	469-22-7	C17H22N2O5	334.37529	μ-opioid receptor agonist, analgesic	Coynanthe yohimbe	experimental	Can J Physiol Pharmacol 59:307 (1981); Eur J Pharmacol	-0.47	-0.92	0.65	-1.15	-0.47
00100032	GEDUNIN	100122-18	E08	sample	2753-30-2	C28H34O7	482.57898	antifeedant; heat shock inducer	numerous Meliaceae species	experimental	J Chem Soc 1960:3827; 1962:5095; 1967:1026; 1969:864;	-4.10	-0.80	-2.68	-2.65	-2.56
00100618	3alpha-HYDROXY-4,4-BISNOR-8,11,13-	100122-18	E09	sample		C15H20O	216.32605		semisynthetic	undetermined activity		0.31	0.06	0.03	-0.93	-0.13
00100291	STROPHANTHIDIN	100122-18	E10	sample	66-28-4	C23H32O6	404.50789	cardiotonic	Strophanthus kombe	experimental	J Soc Chem Ind 53: 956 (1934)	-2.61	-1.36	-1.98	-2.85	-2.20
00100318	DIOSGENIN	100122-18	E11	sample	512-04-9	C27H42O3	414.63399	antiinflammatory, estrogen; LD50(rat) 4872 mg/kg ip. LD50	Clintonia, Dioscorea and Solanum spp, Trillium erectum, Balanites	experimental	Konstitution und Vorkommen der Organischen	-1.56	1.33	0.84	0.11	0.18
	0.11% DMSO	100122-18	E12	0.11% dmso								0.39	1.26	0.59	0.08	0.58
	0.11% JAK2inhib	100122-18	F01	0.11% jak2inhib								-2.50	-3.77	-3.85	-3.13	-3.31
00100375	alpha-DIHYDROGEDUNOL	100122-18	F02	sample		C28H38O7	486.61086		derivative	undetermined activity	J Chem Soc 1961: 3705	-0.22	-3.79	-2.55	-1.01	-1.89
00100434	DIHYDRODEOXYGEDUNIN	100122-18	F03	sample		C28H36O6	468.59552		Meliaceae spp	undetermined activity		2.10	2.27	1.50	2.44	2.08
00270088	CHOLESTAN-3-ONE	100122-18	F04	sample	566-88-1	C27H46O	386.66707		minor mammalian sterol	undetermined activity		-1.29	0.46	0.55	1.55	0.32
01505764	SPERMINE	100122-18	F05	sample	71-44-3	C10H26N4	202.34552	immune modulator	all animal tissue, fungi	experimental	Differentiation 19:1 (1981); Cancer Res 42:3248 (1982)	-0.29	-1.28	-0.18	0.90	-0.21
00100529	HETEROPEUCENIN, METHYL ETHER	100122-18	F06	sample	26213-95-6	C16H18O4	274.31946		Ptaeroxylon obliquum, Harrisonia perforate ,Neochamaelea	undetermined activity	Tetrahedron Letters 1967:2737	0.34	-0.98	0.22	-1.76	-0.54
00200115	PECTOLINARIN	100122-18	F07	sample	28978-02-1	C29H34O15	622.58533		Cirsium and Linaria spp, Kickxia elatine, Duranta plumieri	undetermined activity	Phytochemistry 12:421 (1973); Chem Pharm Bull 26:2036	0.10	0.33	-0.12	0.89	0.30
00200139	ISOTECTORIGENIN, 7-METHYL ETHER	100122-18	F08	sample		C18H16O6	328.32462		Dalbergia spp	undetermined activity		0.11	-0.49	0.65	5.16	1.36
00200258	2',4'-DIHYDROXYCHALCONE 4'-GLUCOSIDE	100122-18	F09	sample		C21H22O8	402.40469	anthelmintic & antiulcerocenic	aglycone Flamingia chappar, Acacia neovernicosa	experimental	Phytochemistry 21: 1063 (1982); Planta Medica 58: 389	-0.06	2.04	5.09	1.39	2.12
00200004	12a-HYDROXY-9-DEMETHYLMUNDUSERONE-8-	100122-18	F10	sample		C19H16O9	388.33397		derivative	undetermined activity		-0.06	0.91	2.62	0.25	0.93
00200208	ASARYLALDEHYDE	100122-18	F11	sample	4460-86-0	C10H12O4	196.20474	fly attractant	Daucus carota, Acorus calamus, Asarum europaeum	experimental	J Nat Prod 39: 412 (1976)	0.10	1.04	-0.77	-1.26	-0.22
	0.04% DMSO	100122-18	F12	0.04% dmso								-0.46	1.66	1.12	-0.02	0.57
	0.04% JAK2inhib	100122-18	G01	0.04% jak2inhib								0.44	-2.00	1.17	-1.61	-0.50
00200215	2-METHYL-5,7,8-TRIMETHOXYISOFLAVONE	100122-18	G02	sample		C19H18O5	326.35231		derivative	undetermined activity		1.22	-0.24	0.57	1.58	0.78
00200243	GRISEOFULVIC ACID	100122-18	G03	sample	469-54-5	C16H15ClO6	338.74735		semisynthetic	undetermined activity		0.42	0.50	-0.62	-0.45	-0.04
00200412	2,3,4'-TRIHYDROXY-4-METHOXYBENZOPHENONE	100122-18	G04	sample		C14H12O5	260.24874		derivative	undetermined activity		1.10	1.37	-0.14	0.50	0.71
00200422	KOPARIN	100122-18	G05	sample	65048-75-1	C16H12O6	300.27044		Castanospermum australe	undetermined activity	Aust J Chem 30:1827 (1977)	-0.16	-0.19	0.98	0.78	0.35
00200425	2,3-DIHYDROXY-4-METHOXY-4'-	100122-18	G06	sample		C16H16O5	288.30292		derivative	undetermined activity		-0.07	-0.97	-0.68	2.60	0.22
00200090	OBTUSAQUINONE	100122-18	G07	sample	21105-15-7	C16H14O3	254.28818		Dalbergia retusa	undetermined activity	Phytochemistry 17: 1395 (1978)	-0.67	0.37	-0.95	0.40	-0.21
00200427	PISCIDIC ACID	100122-18	G08	sample	35388-57-9	C11H12O7	256.21409		Piscidia piscipula	undetermined activity	J Chem Soc 1954: 3981; Acta Chem Scand 18: 1979	0.04	0.09	0.24	0.21	0.14
00200110	ANTIAROL	100122-18	G09	sample	642-71-7	C9H12O4	184.19359		Antiaris toxicaria	undetermined activity	Helv Chim Acta 45: 1183, 1206, 2285 (1992)	1.56	1.40	0.18	-1.21	0.48
00200111	THEAFLAVIN	100122-18	G10	sample	4670-05-7	C29H24O12	564.50743	antioxidant	pigment in black tea	experimental	Tet Lett 1966:1193, 4024; Tetrahedron 29:125 (1973)	0.25	1.15	2.43	-0.02	0.96
00200010	HAEMATOXYLIN	100122-18	G11	sample	517-28-2	C16H14O6	302.28638		Haemotoxylon campechianum	undetermined activity	Bul Soc Chim Fr 1972: 3292	-0.73	0.89	1.31	0.41	0.47
	0.04% DMSO	100122-18	G12	0.04% dmso								0.22	4.10	1.52	0.13	1.49
	0.04% JAK2inhib	100122-18	H01	0.04% jak2inhib								-1.51	-3.15	-1.61	-2.02	-2.07
00200011	MUNDULONE	100122-18	H02	sample	481-94-7	C26H26O6	434.49352		Mundulea sericea	undetermined activity	Tet Letters 1963, 281	6.03	7.66	5.40	8.33	6.86
00200012	BRAZILIN	100122-18	H03	sample	474-07-7	C16H14O5	286.28698		Haematoxylin campechianum	undetermined activity	J Chem Soc 1928, 1504	-0.15	-1.56	-1.59	1.79	-0.38
00200054	FUMARPROTOCETRARIC ACID	100122-18	H04	sample	489-50-9	C22H16O12	472.36562		Cetraria islandica	undetermined activity	Chem Ber 67: 411, 766 (1934)	0.76	-0.43	0.60	0.25	0.30
00200035	GANGALEOIDIN	100122-18	H05	sample	55365-63-4	C18H14Cl2O7	413.21408		Lecanora gangaleoides	undetermined activity	J Chem Soc 1986: 1491	0.98	0.55	0.27	1.25	0.76
00200070	LECANORIC ACID	100122-18	H06	sample	480-56-8	C16H14O7	318.28578		Common constituent of lichens	undetermined activity	Monatsh Chem 61:147 (1932)	0.28	-0.08	0.54	-0.10	0.16
00100584	GITOXIGENIN DIACETATE	100122-18	H07	sample	5996-03-2	C27H38O7	474.59971		aglycon of GITOXIN	undetermined activity	Helv Chim Acta 33: 76 (1950)	-4.38	-3.20	-6.13	-5.36	-4.76
00100599	2-METHYL GRAMINE	100122-18	H08	sample		C12H16N2	188.27472		derivative	undetermined activity		0.17	-0.60	-1.42	-1.09	-0.74
00100609	XANTHYLETIN	100122-18	H09	sample	553-19-5	C14H12O3	228.24994		Brosimum rubescens, Ruta, Boenninghausenia, Flindersia,	undetermined activity	J Chem Soc (C) 1969:33; Phytochemistry 11:3479 (1972)	0.24	1.37	1.20	2.24	1.26
00100615	CHUKRASIN METHYL ETHER	100122-18	H10	sample		C43H58O16	830.93211		Meliaceae	undetermined activity	Helv Chim Acta 61: 1814 (1978)	-2.47	0.03	-0.74	-0.37	-0.89
00100616	ANGOLENSIN (R)	100122-18	H11	sample	4842-48-2	C16H16O4	272.30352		Pericopsis and Pterocarpus spp	undetermined activity	J Chem Soc 1959: 2679; 1963: 5573	-0.28	1.78	0.62	-0.19	0.48
	empty	100122-18	H12	sample								NA	NA	NA	NA	NA
	empty	100122-19	A01	sample								NA	NA	NA	NA	NA
00200428	ROCCELLIC ACID	100122-19	A02	sample	22139-54-4	C17H32O4	300.44219		Lecanora and Roccella spp	undetermined activity	J Chem Soc 1971: 1318	-1.78	-2.12	-1.48	-1.78	-1.79
00200446	METHYLXANTHOXYLIN	100122-19	A03	sample	23121-32-6	C11H14O4	210.23183		Acradenia franklinii, Eugenia jambolana	undetermined activity	J Chem Soc 1955:105	-0.76	-0.97	-0.20	-0.03	-0.49
00200457	ANHYDROBRAZILIC ACID	100122-19	A04	sample		C12H10O5	234.2105		derivative	undetermined activity		-0.80	-0.03	-0.77	-0.40	-0.50
00200484	DEOXYSAPPANONE B 7,4'-DIMETHYL ETHER	100122-19	A05	sample		C18H18O5	314.34116		Caesalpinia sappan	undetermined activity	Chem Pharm Bull 34: 2506 (1986); 35: 3597 (1987)	-1.44	0.06	-0.42	-0.47	-0.57
00200523	XANTHONE	100122-19	A06	sample	90-47-1	C13H8O2	196.20751		gentian and other flowers	undetermined activity	Phytochemistry 4: 411 (1968)	0.67	0.26	1.16	-0.90	0.30
00200640	3-METHYLORSELLINIC ACID	100122-19	A07	sample	4707-46-4	C9H10O4	182.17765		Aspergillus terreus	undetermined activity	Helv Chim Acta 11: 864 (1928)	-0.68	0.11	-0.84	-0.85	-0.57
00200690	2-BENZOYL-5-METHOXYBENZOQUINONE	100122-19	A08	sample		C14H10O4	242.2334		synthetic dalbergione analog	undetermined activity		0.16	0.59	-0.46	-0.42	-0.03
00200743	ERGOSTEROL	100122-19	A09	sample	57-87-4	C28H44O	396.66228		yeast	undetermined activity	J Biol Chem 80: 15 (1928)	-2.98	-1.34	-0.03	-1.46	-1.45
00200744	ERGOSTEROL ACETATE	100122-19	A10	sample	2418-45-3	C32H50O2	466.7541		derivative; mp 179-181 C	undetermined activity		-6.19	-1.55	-0.24	-1.25	-2.31
00200488	NORSTICTIC ACID PENTAACETATE	100122-19	A11	sample		C28H24O15	600.49448		derivative of norstictic acid (00240916)	undetermined activity		1.22	-0.69	0.68	-0.93	0.07
	0.04% JAK2inhib	100122-19	A12	0.04% jak2inhib								-0.76	-0.55	1.06	-2.43	-0.67
	0.04% DMSO	100122-19	B01	0.04% dmso								-2.51	-1.41	1.06	-1.44	-1.07
00200759	IRETOL	100122-19	B02	sample		C7H8O4	156.13941		semisynthetic	undetermined activity	J Chem Soc 1927: 3028	-1.37	-0.85	-0.82	-0.30	-0.83
00200763	IRIGENIN, 7-BENZYL ETHER	100122-19	B03	sample		C25H22O8	450.44929		derivative of irigenin from Iris spp.	undetermined activity		-0.80	-0.45	-0.80	-0.12	-0.54
00200793	IRIDIN	100122-19	B04	sample	491-74-7	C24H26O13	522.46702		Iris spp.	undetermined activity	J Chem Soc 1928:1022; Phytochemistry 22:2061 (1983);	-1.63	-0.75	-0.31	-1.77	-1.12
00200798	DALBERGIONE, 4-METHOXY-4'-HYDROXY-	100122-19	B05	sample		C16H14O4	270.28758		Dalbergia spp	undetermined activity	Chem Commun 1968: 1392; mp 174-176 C (blackens)	-0.57	-0.66	0.52	-0.33	-0.26
00200833	ACACETIN DIACETATE	100122-19	B06	sample	5892-39-7	C20H16O7	368.34632		derivative of acacetin	undetermined activity	J Chem Soc 128: 2344 (1926)	0.86	0.30	0.10	-0.85	0.10
00200846	APIGENIN	100122-19	B07	sample	520-36-5	C15H10O5	270.24395	antispasmodic, antineoplastic, topoisomerase I inhibitor	parsley seed	experimental	J Chem Soc 1963: 1477; Eur J Cancer 32A: 146 (1996); J	-1.37	0.86	0.09	1.41	0.25
00200848	DEOXYSAPPANONE B 7,3'-DIMETHYL ETHER	100122-19	B08	sample		C18H18O5	314.34116		Caesalpinia sappan	undetermined activity	Chem Pharm Bull 34: 2506 (1986); 35: 3597 (1987)	-0.18	0.43	0.52	-0.08	0.17
00200873	IRIGENIN TRIMETHYL ETHER	100122-19	B09	sample		C21H22O8	402.40469		derivative	undetermined activity	Prog Chem Org Nat Prod 43: 1 (1983)	-0.26	-1.35	0.42	0.29	-0.23
00201136	SAPPANONE A DIMETHYL ETHER	100122-19	B10	sample		C18H16O5	312.32522		Caesalpinia sappan	undetermined activity	Chem Pharm Bull 34: 2506 (1986); 35: 3597 (1987)	1.90	0.71	0.38	-0.20	0.70
00201078	VIOLASTYRENE	100122-19	B11	sample	19034-96-9	C17H18O3	270.33121		Dalbergia miscolobium	undetermined activity	Chem Commun 1968:1390; Phytochemistry 17:1375,1395	1.29	0.64	0.70	-0.10	0.63
	0.04% JAK2inhib	100122-19	B12	0.04% jak2inhib								-0.58	-1.41	-0.46	-2.08	-1.13
	0.04% DMSO	100122-19	C01	0.04% dmso								-0.21	-1.67	-0.47	-0.75	-0.77
00201154	DEHYDROROTENONE	100122-19	C02	sample	30990-44-4	C23H20O6	392.41225		Derris spp; rotenone derivative	undetermined activity	Chem Rev 12: 181 (1933)	0.36	-0.04	2.33	1.28	0.98
00201177	DUARTIN (-)	100122-19	C03	sample	52305-04-1	C18H20O6	332.3565		Dalbergia variabilis	undetermined activity	Phytochemistry 17: 1401 (1978)	0.45	-0.14	-0.50	0.86	0.17
00201181	IRIGENIN, DIBENZYL ETHER	100122-19	C04	sample		C32H28O8	540.57516		derivative of Irigenin	undetermined activity		0.51	-1.66	0.72	0.28	-0.04
00201227	NONIC ACID	100122-19	C05	sample		C9H16O4	188.22547		hydroysis product of actinonin	undetermined activity		0.33	-0.89	-1.27	-0.62	-0.61
00201281	DALBERGIONE	100122-19	C06	sample		C15H12O2	224.26169		Dalbergia spp	undetermined activity		1.54	0.09	0.82	-1.16	0.32
00201310	2'-METHOXYFORMONETIN	100122-19	C07	sample		C17H14O5	298.29813		Eschscholtzia californica	undetermined activity	Phytochemistry 41: 661 (1996)	0.90	-0.15	-0.21	1.40	0.49
00201331	DEOXYSAPPANONE B TRIMETHYL ETHER	100122-19	C08	sample		C19H20O5	328.36825		Caesalpinia sappan	undetermined activity	Chem Pharm Bull 34: 2506 (1986); 35: 3597 (1987)	1.34	2.16	0.85	0.49	1.21
00201342	DEOXYSAPPANONE B 7,3'-DIMETHYL ETHER	100122-19	C09	sample		C20H20O6	356.3788		derivative Caesalpinia sappan	undetermined activity	Chem Pharm Bull 34: 2506 (1986); 35: 3597 (1987)	0.23	0.03	-0.03	-1.87	-0.41
00201364	DUARTIN, DIMETHYL ETHER	100122-19	C10	sample		C20H24O6	360.41068		derivative of DUARTIN (00201177)	undetermined activity		0.47	2.02	2.40	2.70	1.90
00201448	4,4'-DIMETHOXYDALBERGIONE	100122-19	C11	sample		C17H16O4	284.31467		Dalbergia nigra	undetermined activity	Tetrahedron 21: 2683 (1965)	0.17	-0.95	0.07	4.54	0.95
	0.11% JAK2inhib	100122-19	C12	0.11% jak2inhib								-3.39	-3.58	3.03	-3.78	-1.93
	0.11% DMSO	100122-19	D01	0.11% dmso								-1.52	-2.51	1.49	-0.66	-0.80
00201477	MUNDOSERONE	100122-19	D02	sample	3564-85-0	C19H18O6	342.35171		Mundulea suberosa; mp 156-161 C	undetermined activity	Proc Chem Soc 1960:176; 1969:177	4.06	3.79	3.83	4.02	3.93
00201602	PACHYRRHIZIN	100122-19	D03	sample	10091-01-7	C19H12O6	336.30389	insecticide	Pachyrrhizus erosus; mp 206-207 C	experimental	J Am Chem Soc 67: 1609 (1945)	-0.78	-2.03	-1.28	-2.28	-1.59
00202130	PERSEITOL	100122-19	D04	sample	527-06-0	C7H16O7	212.20137		Persia spp.	undetermined activity	J Am Chem Soc 61: 339 (1939); Carbohydr Res 150: 35	-0.46	-0.55	0.62	0.16	-0.06
00202178	EPIAFZELECHIN (2R,3R)(-)	100122-19	D05	sample	24808-04-6	C15H14O5	274.27583		Larix sibirica, Actinidia chinensis, Juniperus communis, Cassia	undetermined activity	J Chem Soc 1955: 2948; Aust. J. Chem 13: 390 (1960);	-0.42	0.36	-0.70	-0.74	-0.37
00201727	EVERNINIC ACID	100122-19	D06	sample	570-10-5	C8H8O5	184.14996		oak moss lichen	undetermined activity	Ber 56: 2556 (1923); JACS 88: 2053 (1966); Tetrahedron	0.75	0.93	1.27	0.72	0.92
00210211	EPIAFZELECHIN TRIMETHYL ETHER	100122-19	D07	sample		C18H20O5	316.3571		derivative	undetermined activity		0.26	0.23	0.70	0.33	0.38
00210296	GENISTEIN	100122-19	D08	sample	446-72-0	C15H10O5	270.24395		widely distributed in Leguminoseae	undetermined activity	Phytochemistry 7: 791 (1968)	0.36	1.00	0.20	0.58	0.54
01504166	ISOOSAJIN	100122-19	D09	sample	5745-54-0	C25H24O5	404.46703		Maclura pomifera	undetermined activity	J Am Chem Soc 61: 2832 (1939)	1.09	0.03	1.10	0.31	0.63
00211949	APOTOXICAROL	100122-19	D10	sample		C18H14O7	342.30808		derivative	undetermined activity		-0.88	-0.36	-0.21	0.08	-0.34
00107023	SITOSTERYL ACETATE	100122-19	D11	sample		C31H52O2	456.75889		widespread in plants	undetermined activity	J Am Chem Soc 48: 2987 (1926)	-2.47	-1.77	0.26	-2.30	-1.57
	0.11% JAK2inhib	100122-19	D12	0.11% jak2inhib								-2.26	-2.49	-1.28	-4.34	-2.59
	0.11% JAK2inhib	100122-19	E01	0.11% jak2inhib								-3.37	-4.33	-3.29	-2.23	-3.30
00300001	ORSELLINIC ACID	100122-19	E02	sample	480-64-8	C8H8O4	168.15056		common lichen constituent	undetermined activity		-0.09	1.68	-0.74	1.45	0.58
00300003	SOLIDAGENONE	100122-19	E03	sample	23534-56-7	C20H28O3	316.44436		Solidago canadensis L.; mp 133-134 C	undetermined activity	Tetrahedron 25, 2233 (1969)	-0.31	-0.59	-0.70	-0.03	-0.41
00300005	SPHONDIN	100122-19	E04	sample	483-66-9	C12H8O4	216.19516		Heracleum maximum root; mp 191-192 C	undetermined activity	Ber 74:595 (1941); Monatsh Chem 84:217 (1953)	1.55	0.63	0.46	1.82	1.11
00300007	EUPARIN	100122-19	E05	sample	532-48-9	C13H12O3	216.23879		Eupatorium macculatum	undetermined activity	J Chem Soc 1939:925	0.28	1.16	0.87	0.03	0.58
00300006	STICTIC ACID	100122-19	E06	sample	549-06-4	C19H14O9	386.31803		numerous lichens, e.g. Parmelia sp.	undetermined activity	Ber 66, 943 (1933); Tetrahedron 24, 2707 (1966)	-0.43	0.55	-0.32	0.12	-0.02
00300010	AGELASINE (stereochemistry of diterpene unknown)	100122-19	E07	sample		C26H40ClN5	458.0952	cytotoxic, antineoplastic	Agelas dispar	experimental	Can J Chem 53:1690 (1975)	-0.52	1.46	-0.32	0.63	0.31
00300012	ISOPIMPINELLIN	100122-19	E08	sample	482-27-9	C13H10O5	246.22165		Hercaleum maximum; mp 150-153 C dimorphic (142-145 C)	undetermined activity	J Chem Soc 1945: 540	-1.90	-0.17	-1.99	-0.27	-1.08
00300032	ISOBERGAPTENE	100122-19	E09	sample	482-48-4	C12H8O4	216.19516		Heracleum and other Umbelliferaceae; mp 223-224 C	undetermined activity	Ber 70: 1253 (1937)	-0.94	-0.62	0.84	-1.42	-0.54
00300021	GIBBERELLIC ACID	100122-19	E10	sample	77-06-5	C19H22O6	346.38359		Gibberella fujikuroi	undetermined activity	J Chem Soc 1954: 1066	1.72	0.03	0.71	1.76	1.05
00300013	PIMPINELLIN	100122-19	E11	sample	131-12-4	C13H10O5	246.22165	GABA receptor antagonist, phototoxin	Heracleum maximum root; mp 118-119 C	experimental	Monatsh 59: 161 (1932); J Org Chem 35: 2294 (1970)	0.44	-1.33	-0.84	-2.28	-1.00
	0.11% DMSO	100122-19	E12	0.11% dmso								-0.22	0.92	-0.13	-1.15	-0.14
	0.11% JAK2inhib	100122-19	F01	0.11% jak2inhib								-4.01	-3.83	-3.35	-3.20	-3.60

												Screen Score (z-score)				
Compound ID	MolNAME	plate	position	Content	cas#	Formula	MolWt	Bioactivity	Source	Status	Reference	rep1	rep2	rep3	rep4	Average
00300020	1-MONOPALMITIN	100122-19	F02	sample	542-44-9	C19H38O4	330.51231		Asparagus, Momodica spp; widespread in plants	undetermined activity		-1.13	-0.61	1.79	0.05	0.03
00300058	EPI(13)TORULOSOL	100122-19	F03	sample	3650-30-4	C20H34O2	306.49278		Cryptomeria japonica and Larix sibirica	undetermined activity	J Org Chem 29: 1554 (1964); Phytochemistry 13:471 (1974)	-1.05	-0.62	0.03	1.77	0.03
00300055	CADIN-4-EN-10-OL	100122-19	F04	sample		C15H26O	222.37387		Chamaecyparis spp and Juniperus spp.	undetermined activity	Tet Lett 1968: 1913	-0.17	2.27	0.69	1.16	0.99
00300056	LARIXOL	100122-19	F05	sample		C20H34O2	306.49278		Larix sibiricia	undetermined activity	Tet Letters 1965: 3523; 1967: 219	0.28	0.67	-0.25	2.41	0.78
00300057	LARIXOL ACETATE	100122-19	F06	sample		C22H36O3	348.53042		Larix europaea	undetermined activity	Tet Letters 1965: 3523; 1967: 220	0.22	0.82	-0.73	-0.16	0.04
00300146	VULPINIC ACID	100122-19	F07	sample	521-52-8	C19H14O5	322.32043	antiinflammatory, antibacterial, plant growth inhibitor	numerous lichens, e.g. Letharia vulpina	experimental	J Am Chem Soc 72:1824 (1950)	-0.12	0.74	-0.44	0.31	0.12
00300110	3-NOR-3-OXOPANASINSAN-6-OL	100122-19	F08	sample		C14H22O2	222.33024		derivative Panax ginseng	undetermined activity	Chem Pharm Bull 35: 1975 (1987)	1.40	-0.19	1.13	0.08	0.60
00300111	2-METHOXY-5 (6)EPOXY-	100122-19	F09	sample		C16H28O2	252.40036		derivative	undetermined activity		-0.08	-0.29	0.23	-1.04	-0.30
00300117	ISOKOBUSONE	100122-19	F10	sample	24173-72-6	C14H22O2	222.33024		Cyperus rotundus, Sindora sumatrana	undetermined activity	Chem Pharm Bull 17:1390 (1969); 42:138 (1994)	0.55	0.61	3.57	1.27	1.50
00300118	3,7-EPOXYCARYOPHYLLAN-6-OL	100122-19	F11	sample		C15H26O2	238.37327		derivative Lippia spp	undetermined activity		-2.74	0.20	3.88	-0.85	0.12
	0.04% DMSO	100122-19	F12	0.04% dmso								1.43	0.20	1.55	-0.31	0.72
	0.04% JAK2inhib	100122-19	G01	0.04% jak2inhib								-2.05	-2.61	-2.78	-1.47	-2.23
00300119	2-HYDROXY-5 (6)EPOXY-	100122-19	G02	sample		C15H26O2	238.37327		derivative	undetermined activity		-0.87	-0.34	-0.15	0.28	-0.27
00300132	3,7-EPOXYCARYOPHYLLAN-6-ONE	100122-19	G03	sample		C15H24O2	236.35733		derivative Lippia spp	undetermined activity		0.34	1.36	-0.08	0.77	0.60
00300133	CLOVANEDIOL DIACETATE	100122-19	G04	sample		C19H30O4	322.44855		derivative Dipterocarpus pilosus, Salvia canariensis, Viguiera	undetermined activity	Chem Pharm Bull 42: 138 (1994)	-1.44	-1.33	-0.65	-1.24	-1.16
00307047	3-OXOURSAN (28-13)OLIDE	100122-19	G05	sample		C30H44O3	452.68338		semisynthetic	undetermined activity		0.03	2.51	-0.50	1.49	0.88
00307050	DEHYDROABIETAMIDE	100122-19	G06	sample		C20H29NO	299.46023		derivative	undetermined activity		-4.53	-3.47	-3.72	-2.96	-3.67
00307056	MUUROLLADIE-3-ONE	100122-19	G07	sample		C15H22O	218.34199		derivative of muurolene	undetermined activity	Coll Czech Chem Comm 31: 3373 (1966)	-0.31	-0.04	0.24	-0.06	-0.04
00300160	3-PINANONE OXIME	100122-19	G08	sample		C10H17NO	167.25309		derivative	undetermined activity		0.77	0.95	0.72	3.41	1.46
00300531	MELEZITOSE	100122-19	G09	sample		C18H32O16	504.44614		honey & plant exudates	undetermined activity	J Org Chem 11: 610 (1946)	2.09	1.17	0.72	0.83	1.20
00300539	ARBUTIN	100122-19	G10	sample	497-76-7	C12H16O7	272.25712		Berginia crassifolia; also in Pyrus and Vaccinium spp.	undetermined activity	Pharmazie 15: 650 (1960)	0.76	0.41	0.94	3.43	1.39
00300533	ISOSAFROLE	100122-19	G11	sample	120-58-1	C10H10O2	162.19		Illicium religiosum	undetermined activity	J Chem Soc 1927:2489; J Biol Chem 255:7941 (1980); J	1.40	0.23	0.47	3.70	1.45
	0.04% DMSO	100122-19	G12	0.04% dmso								0.29	0.32	0.40	0.18	0.30
	0.04% JAK2inhib	100122-19	H01	0.04% jak2inhib								-2.68	-2.12	-0.13	1.06	-0.97
00300540	HYMECROMONE METHYL ETHER	100122-19	H02	sample	2555-28-4	C11H10O3	190.20055		Dalbergia volubilis, Eupatorium pauciflorum	undetermined activity	Indian J Chem 15:94, 492 (1977)	-0.62	-0.93	-2.12	0.15	-0.88
00300537	XANTHOPTERIN	100122-19	H03	sample	119-44-8	C6H5N5O2	179.13905	cell proliferation inhibitor	human urine, butterfly wing pigment	experimental	J Het Chem 29:583 (1992); Int J Biochem 25:1873 (1993)	2.46	-0.04	-0.76	0.52	0.55
00300542	SANTONIN	100122-19	H04	sample	481-06-1	C15H18O3	246.30891		Artemisia spp.	undetermined activity	J Chem Soc 1930: 1110	-1.26	-0.67	-1.26	-0.17	-0.84
00300547	PHLORIDZIN	100122-19	H05	sample	60-81-1	C21H24O10	436.41943	induces experimental glucosuria, antifeedant	Rosaceae spp	experimental	J Chem Soc 1933: 1170; Physiol Rev 25: 255 (1945)	-1.48	0.35	-1.13	-0.31	-0.64
00300558	GEDUNOL	100122-19	H06	sample		C28H36O7	484.59492		Meliaceae spp	undetermined activity		1.07	-1.14	-0.51	-0.26	-0.21
00300566	CHRYSANTHEMYL ALCOHOL	100122-19	H07	sample	5617-92-5	C10H18O	154.25436		reduction product of pyrrethrin constituent	undetermined activity		-1.14	-0.35	-0.52	-1.23	-0.81
00300564	MENTHONE	100122-19	H08	sample	14073-97-3	C10H18O	154.25436		pennyroyal and peppermint oils	undetermined activity	J Org Chem 25: 1434 (1960)	0.66	2.21	1.06	0.25	1.05
00300556	CHRYSAROBIN	100122-19	H09	sample	491-58-7	C15H12O3	240.26109		Andira araroba (glacial AcOH)	undetermined activity	J Am Chem Soc 53: 4114 (1931)	0.67	1.41	2.98	1.72	1.69
00300553	PELLETIERINE HYDROCHLORIDE	100122-19	H10	sample		C8H16ClNO	177.67582		Punica granatum	undetermined activity	Bull Soc Chim Fr 1961: 1993	0.94	-0.26	-0.30	-1.77	-0.35
00300554	PHLORETIN	100122-19	H11	sample	60-82-2	C15H14O5	274.27583		Prunus spp	undetermined activity	Ann Rev Biochem 20: 4955 (1951)	-0.03	-1.08	-0.52	-2.24	-0.97
	empty	100122-19	H12	sample								NA	NA	NA	NA	NA
	empty	100122-20	A01	sample								NA	NA	NA	NA	NA
00210220	CATECHIN TETRAMETHYLETHER	100122-20	A02	sample		C19H22O6	346.38359		derivative	undetermined activity		-2.32	-2.28	-0.88	-1.30	-1.70
00200499	ACACETIN	100122-20	A03	sample	480-44-4	C16H12O5	284.27104	antiinflammatory, spasmolytic agent, antioxidant	Robinia pseudoacacia	experimental	J Pharm Soc Japan 73: 481 (1953); Experientia 47: 195	1.30	-0.98	-1.12	-1.99	-0.70
01504256	EPICATECHIN PENTAACETATE	100122-20	A04	sample		C25H24O11	500.46343		derivative	undetermined activity		-2.10	-1.79	-0.18	0.00	-1.02
00205113	EPIGALLOCATECHIN	100122-20	A05	sample	970-74-1	C15H14O7	306.27463		green tea	undetermined activity		2.32	-1.48	0.05	-1.14	-0.06
01600537	CATECHIN PENTAACETATE	100122-20	A06	sample		C25H24O11	500.46343		derivative	undetermined activity		-1.88	-1.25	-0.85	-0.72	-1.17
00201513	EPIGALLOCATECHIN 3,5-DIGALLATE	100122-20	A07	sample		C29H22O15	610.48969		tea pigment	undetermined activity		-0.45	-1.61	-0.38	3.52	0.27
00210206	EPICATECHIN	100122-20	A08	sample	490-46-0	C15H14O6	290.27523	antioxidant	tea and cocoa constituent	experimental	J Chem Soc 1969: 1824; Acta Cryst C 40: 2068 (1984)	-1.51	0.65	-1.44	-1.85	-1.04
00210239	EPIGALLOCATECHIN-3-MONOGALLATE	100122-20	A09	sample	989-51-5	C22H18O11	458.38216		tea pigment	undetermined activity		3.06	-0.26	0.86	0.43	1.02
00201507	2',2'-BISEPIGALLOCATECHIN DIGALLATE	100122-20	A10	sample		C44H34O22	914.74838		tea pigment	undetermined activity		-0.01	0.24	4.90	0.00	1.28
00210238	EPICATECHIN MONOGALLATE	100122-20	A11	sample	1257-08-5	C22H18O10	442.38276		tea pigment	undetermined activity		0.18	0.38	0.82	2.89	1.07
	0.04% JAK2inhib	100122-20	A12	0.04% jak2inhib								-1.18	-0.12	-0.31	-2.59	-1.05
	0.04% DMSO	100122-20	B01	0.04% dmso								-2.60	-4.61	3.44	-3.90	-1.92
00310041	VISNAGIN	100122-20	B02	sample	82-57-5	C13H10O4	230.22225		Ammi visnaga	undetermined activity	Tetrahedron 3: 230 (1958)	-2.22	-2.74	3.95	-1.20	-0.55
00310298	GLUCOSAMINIC ACID	100122-20	B03	sample		C6H13NO6	195.17361		oxidation product of glucosamine (01500316)	undetermined activity		1.09	-1.83	-0.10	-0.11	-0.24
00310030	RHODINYL ACETATE	100122-20	B04	sample		C12H22O2	198.30794		common constituent of plant essential oils	undetermined activity		3.94	-0.67	-0.59	2.72	1.35
00310010	HELENINE	100122-20	B05	sample	546-43-0	C15H20O2	232.32545	anthelmintic, antibacterial, antineoplastic	Inula spp	experimental	J Am Chem Soc 79: 5721 (1957); 88: 3408 (1966); J Org	-0.32	-1.02	-0.59	-0.60	-0.63
00310012	HESPERETIN	100122-20	B06	sample	520-33-2	C16H14O6	302.28638		aglycone of hesperidin (00310011)	undetermined activity	J Chem Soc 1956: 632	-1.42	0.14	-0.92	-0.58	-0.70
00310009	EPIANDROSTERONE	100122-20	B07	sample		C19H30O2	290.44975		in normal human urine	undetermined activity	J Am Chem Soc 75: 2275 (9153)	-0.52	0.27	0.09	-0.55	-0.18
00310008	DJENKOLIC ACID	100122-20	B08	sample	498-59-9	C7H14N2O4S2	254.32863		djenkol bean (Pithecolobium lobatum)	undetermined activity	J Biol Chem 168: 373 (1947)	-0.48	0.73	0.65	4.80	1.42
00310015	CEDRYL ACETATE	100122-20	B09	sample	77-54-3	C17H28O2	264.41151		semisynthetic	undetermined activity		-0.41	0.98	1.65	-0.40	0.46
00310023	HYPOXANTHINE	100122-20	B10	sample	68-94-0	C5H4N4O	136.11383		widely distributed in the plant and animal kingdom	undetermined activity		2.03	0.59	1.31	-0.08	0.96
00310021	PHYTOL	100122-20	B11	sample		C19H38O	282.51411		nettles	undetermined activity	J Chem Soc 1966: 2144	-6.25	-1.35	-0.65	-1.83	-2.52
	0.04% JAK2inhib	100122-20	B12	0.04% jak2inhib								-1.62	-0.43	-0.25	-2.91	-1.30
	0.04% DMSO	100122-20	C01	0.04% dmso								-4.08	-0.94	-1.77	-3.50	-2.57
00310019	CHRYSANTHEMIC ACID, ETHYL ESTER	100122-20	C02	sample		C12H20O2	196.292	insecticide	pyrethrum flowers	experimental	J Chem Soc 1945: 283	-1.14	-2.08	-0.97	2.19	-0.50
00310004	ANABASINE HYDROCHLORIDE	100122-20	C03	sample	13078-04-1 (anabasine)	C10H15ClN2	198.69745	insecticide	Anabasis aphylla & Nicotiana spp	agricultural use	J Am Chem Soc 57: 959 (9154)	0.10	-1.42	-0.61	0.37	-0.39
00310001	ACONITIC ACID	100122-20	C04	sample	585-84-2	C6H6O6	174.11112		Aconitum and Achillea spp	undetermined activity	Ind Eng Chem 33: 637 (1941)	3.20	-0.37	0.02	3.59	1.61
00310011	HESPERIDIN	100122-20	C05	sample	520-26-3	C28H34O15	610.57418	capillary protectant	Citrus spp.	experimental		0.02	2.12	0.91	1.33	1.09
00310025	LARIXINIC ACID	100122-20	C06	sample	118-71-8	C6H6O3	126.11292		Larix decidua	undetermined activity	Ber 27: 3115 (1984); 34: 1804 (1901); 43: 2398 (1910)	-0.76	0.63	0.60	0.25	0.18
00310018	QUINIC ACID	100122-20	C07	sample	77-95-2	C7H12O6	192.17009		Cinchona spp	undetermined activity	Ber 65: 1009 (1932)	3.20	1.70	-0.21	2.49	1.79
00310028	QUASSIN	100122-20	C08	sample	76-78-8	C22H28O6	388.46486	insecticide, antiamoebic	Quassia amara, Picrasma excelsa and Ailanthus glandulosa	experimental	JACS 60:1146 (1938); 72:375 (1950); Tetrahedron 15:100	1.51	0.16	0.50	-0.93	0.31
00350025	APIIN	100122-20	C09	sample	26544-34-3	C26H28O14	564.50466		parsley seed; flowers of Anthemis nobilis. 80% + other glycosides	undetermined activity	Chem Ind 1953: 85; Can J Chem 43: 2085 (1965)	1.13	0.72	-0.32	-0.60	0.23
10101011	BIXIN	100122-20	C10	sample	39937-23-0	C25H30O4	394.51545		Bixa orellana seeds	undetermined activity	Helv Chim Acta 40: 1242 (1957); J Chem Soc 1961: 1625;	-3.94	-0.20	0.55	-0.68	-1.07
00203008	JUAREZIC ACID	100122-20	C11	sample	1552-94-9	C11H10O2	174.20115		propolis and Populus spp	undetermined activity	JACS 108: 3016 (1986); Phytochemistry 29: 2551 (1990)	0.72	0.64	0.99	1.71	1.02
	0.11% JAK2inhib	100122-20	C12	0.11% jak2inhib								-2.13	2.31	-1.62	-3.91	-1.34
	0.11% DMSO	100122-20	D01	0.11% dmso								0.32	-0.38	-2.96	-3.53	-1.64
01600759	4-HYDROXY-6-METHYLPYRAN-2-ONE	100122-20	D02	sample	675-10-5	C6H6O3	126.11292		Pennicillium stipitatum	undetermined activity	J Am Chem Soc 89: 676, 681 (1967)	1.10	-1.80	0.66	-0.56	-0.15
01400164	2-HYDROXY-3,4-DIMETHOXYBENZOIC ACID	100122-20	D03	sample		C9H10O5	198.17705	prostaglandin synthetase inhibitor	Dalbergia odorifera	experimental		1.14	-1.07	-0.56	-0.22	-0.18
01400131	HAEMATOMMIC ACID	100122-20	D04	sample	479-25-4	C9H8O5	196.16111		Alectoria spp, Haematomma spp and Lethariella spp	undetermined activity	J Org Chem 42: 2526 (1977); Helv Chim Acta 72: 1061	-0.35	-1.57	-0.05	-0.47	-0.61
01400156	ISOPEONOL	100122-20	D05	sample	493-33-4	C9H10O3	166.17825		Paeonia spp	undetermined activity		-0.85	-0.05	0.17	0.46	-0.07
01400136	N-METHYLISOLEUCINE	100122-20	D06	sample	5125-98-8	C7H15NO2	145.2031		Phasealus vulgaris	undetermined activity	Phytochemistry 42: 443 (1996)	0.15	0.12	0.09	-0.13	0.06
01600300	CREATININE	100122-20	D07	sample	60-27-5	C4H7N3O	113.11989	metabolic enhancer	metabolite in muscle and urine; renal metabolite	undetermined activity	J Biol Chem 81:73 (1929); JACS 93:5552 (1971)	-0.18	-0.38	0.51	0.58	0.13
01601000	MEVASTATIN	100122-20	D08	sample	73573-88-3	C23H34O5	390.52443	antihyperlipidemic, HMGCoA reductase inhibitor	Penicillium brevicompactum; Compactin	INN		0.68	0.00	0.78	1.60	0.76
01600964	N-METHYLANTHRANILIC ACID	100122-20	D09	sample	119-68-6	C8H9NO2	151.16643		grapefruit peel oil	undetermined activity	Ber 42: 3194 (1909)	0.69	4.87	0.89	0.01	1.61
01600480	TETRAHYDROSAPPANONE A TRIMETHYL ETHER	100122-20	D10	sample		C19H22O5	330.38419		derivative Caesalpinia sappan	undetermined activity	Chem Pharm Bull 34: 2506 (1986); 35: 3597 (1987)	0.99	0.24	-0.07	0.38	0.38
00300610	ACETOSYRINGONE	100122-20	D11	sample	2478-38-8	C10H12O4	196.20474	insect attractant, plant hormone	several commercial woods	experimental	Can J Chem 34: 1562 (1956)	2.71	1.06	1.08	3.35	2.05
	0.11% JAK2inhib	100122-20	D12	0.11% jak2inhib								-2.88	-1.21	-1.80	-3.45	-2.33
	0.11% JAK2inhib	100122-20	E01	0.11% jak2inhib								-5.31	-1.33	-4.36	-2.93	-3.48
01601021	PAEONOL	100122-20	E02	sample	552-41-0	C9H10O3	166.17825	antibacterial	Paeonia montan, Xanthorrhoea spp	experimental		-0.56	-1.98	-1.78	-0.70	-1.25
01503218	HEPTAMINOL HYDROCHLORIDE	100122-20	E03	sample	543-15-7, 372-66-7	C8H20ClNO	181.7077	vasodilator	synthetic	INN, BAN		1.19	-0.55	-0.90	0.35	0.02
00270083	5alpha-CHOLESTAN-3beta-OL-6-ONE	100122-20	E04	sample		C27H46O2	402.66647		Mandevilla pentlandiana	undetermined activity		-0.11	0.10	0.95	-0.31	0.16
00240437	PERSEITOL HEPTAACETATE	100122-20	E05	sample	19147-10-5	C21H30O14	506.46485		derivative of perseitol	undetermined activity		1.17	-0.18	0.53	2.44	0.99
00240429	3,4-DIDESMETHYL-5-DESHYDROXY-3'-	100122-20	E06	sample		C15H14O5	274.27583		synthetic	undetermined activity	Tetrahedron 21: 2697 (1965)	-0.62	0.02	-0.33	0.13	-0.20
00500580	PIPERONYLIC ACID	100122-20	E07	sample	94-53-1	C8H6O4	166.13462		Piper longum, Cinnamumum spp	undetermined activity	J Chem Soc 1950: 2376	-0.20	-0.78	0.38	0.95	0.08
00501332	PHENACYLAMINE HYDROCHLORIDE	100122-20	E08	sample	5468-37-1, 613-89-8(base)	C8H10ClNO	171.628		Castanopsis cuspidata, Vitis spp	undetermined activity	Planta Med 22: 434 (1972)	3.24	2.75	1.42	0.14	1.89
00500123	CYCLOVERATRYLENE	100122-20	E09	sample		C27H30O6	450.53655		Halopythus spp	undetermined activity	Tet Lett 1978: 1699; 33: 5846 (1992)	0.10	0.36	0.50	-0.25	0.18
00501000	d,l-threo-3-HYDROXYASPARTIC ACID	100122-20	E10	sample		C4H7NO5	149.10409	L-aspartate beta-carboxylase inhibitor	Streptomyces spp, Arthrinium phaeospermum,	experimental		1.74	0.63	1.28	-0.84	0.70
00390001	APIOLE	100122-20	E11	sample	523-80-8	C12H14O4	222.24298	antipyretic, diuretic, insecticide	Petroselimum spp, Anethum graveolens	experimental	Phytochemistry 15: 1773 (1976); Pharmazie 34: 426 (1979)	-2.30	-0.03	-0.56	3.01	0.03
	0.11% DMSO	100122-20	E12	0.11% dmso								0.07	0.39	0.12	-1.44	-0.22
	0.11% JAK2inhib	100122-20	F01	0.11% jak2inhib								-5.81	-2.11	-4.33	-5.27	-4.38
01401406	DERRUSNIN	100122-20	F02	sample	14736-62-0	C19H16O7	356.33517		Derris robusta	undetermined activity	J Chem Soc 1969:365	-0.49	-2.02	3.35	4.21	1.26
01401419	DERRUSTONE	100122-20	F03	sample	2204-59-3	C18H14O6	326.30868		Derris robusta	undetermined activity	J Chem Soc 1959: 2679; Phytochemistry 7: 701 (1968); 11:	-1.66	-1.59	-0.11	1.40	-0.49
01401401	METHYL ROBUSTONE	100122-20	F04	sample		C22H18O6	378.38516		Derris spp	undetermined activity	J Indian Chem Soc 18: 471 (9179)	-3.24	-3.33	-3.41	-4.56	-3.64
00100048	7-DEACETOXY-7-OXOKHIVORIN	100122-20	F05	sample	15004-51-0	C30H38O9	542.63196		Khaya senegalensis and other Meliaceae; mp 225	undetermined activity	J Chem Soc 1963: 980	0.25	-0.58	-0.39	0.13	-0.15
00100650	DESACETYL (7)KHIVORINIC ACID, METHYL ESTER	100122-20	F06	sample		C28H40O10	536.625		semisynthetic	undetermined activity		-0.70	-0.82	-0.41	-0.32	-0.56
00100572	FRAXIDIN METHYL ETHER	100122-20	F07	sample		C12H12O5	236.22644		derivative	undetermined activity		0.51	0.39	0.05	0.11	0.27
00100652	DIHYDROXY (3alpha,12alpha)PREGNAN-20-ONE	100122-20	F08	sample		C21H34O3	334.50333		semisynthetic	undetermined activity		-0.05	0.20	5.29	-0.78	1.17
00200033	LEOIDIN	100122-20	F09	sample	105350-54-7	C18H14Cl2O7	413.21408		lichen metabolite	undetermined activity	J Chem Soc 1986: 1491	1.11	1.04	1.09	-0.07	0.79
00300147	USNIC ACID	100122-20	F10	sample	125-46-2	C18H16O7	344.32402	antibacterial	Usnea, Cladonia and other lichen spp	experimental	J Chem Soc, 1953, 603; Antibiotics I (Verlag), 1967, p 611	0.60	0.19	-0.05	0.06	0.20
00307059	CEDROL	100122-20	F11	sample	77-53-2	C15H26O	222.37387	acaricide	Common constitutent in the Family Cupressaceae	experimental	J Org Chem 43: 1964 (1978)	1.15	2.51	0.59	-0.08	1.04
	0.04% DMSO	100122-20	F12	0.04% dmso								1.03	0.56	1.23	-0.47	0.59
	0.04% JAK2inhib	100122-20	G01	0.04% jak2inhib								-4.78	-5.54	-3.89	-0.54	-3.69
00300384	5,7,4'-TRIMETHOXYFLAVONE	100122-20	G02	sample	5631-70-9	C18H16O5	312.32522		Cassia siamia, Citrus reticulata	undetermined activity	Phytochemistry 22: 625 (1983)	-2.90	-2.84	0.54	-0.04	-1.31
00300604	PHLORACETOPHENONE	100122-20	G03	sample	480-66-0	C8H8O4	168.15056		Prunus domesticus	undetermined activity	JACS 54:2452 (1932); J Chem Soc 1953:1241;	-0.47	0.52	0.64	-0.16	0.13
01500624	CARNITINE (dl) HYDROCHLORIDE	100122-20	G04	sample	461-06-3	C7H16ClNO3	197.66347	antihyperlipoproteinemic, gastric/ pancreatic secretion stimulant	striated muscle, liver; also in Pisum sativum	INN, JAN		0.63	0.42	-0.27	0.21	0.25
01500672	QUERCETIN	100122-20	G05	sample	117-39-5	C15H10O7	302.24275	capillary protectant, antioxidant. antineoplastic, anti-HIV;	Solanaceae, Rhamnaceae, Passifloraceae, Umbelliferae genera	experimental	Chem Ber 103:3674 (1970); Experientia 28:380 (1972);	-2.17	-2.59	-1.10	1.40	-1.11
01500678	gamma-AMINOBUTYRIC ACID	100122-20	G06	sample	56-12-2	C4H9NO2	103.12183	antihypertensive	widely distributed in higher plants	JAN		0.00	-0.20	-0.35	1.15	0.15
01500690	MELATONIN	100122-20	G07	sample	73-31-4	C13H16N2O2	232.28467	sleep induction, modifies circadian rhythm	pineal gland	experimental		0.00	1.06	-0.32	-0.19	0.14
01500707	CITROPTEN	100122-20	G08	sample	487-06-9	C11H10O4	206.19995	photosensitizing agent	Bergamot oil	experimental	Indian J Chem 13: 835 (1975)	-0.22	-0.15	-0.81	-1.72	-0.72
01500717	6,4'-DIHYDROXYFLAVONE	100122-20	G09	sample	63046-09-3	C15H10O4	254.24455	antihaemorrhagic	Cassia spp as glycoside	experimental		1.96	1.38	3.84	3.95	2.78
01500719	7,2'-DIHYDROXYFLAVONE	100122-20	G10	sample	77298-66-9	C15H10O4	254.24455	antihaemorrhagic	Primula spp	experimental		1.45	1.15	0.14	-0.19	0.64
01500729	4-METHYLESCULETIN	100122-20	G11	sample		C10H8O4	192.17286		analog of esculetin	undetermined activity		0.70	2.17	3.15	0.04	1.51
	0.04% DMSO	100122-20	G12	0.04% dmso								0.87	1.10	0.27	-0.34	0.48
	0.04% JAK2inhib	100122-20	H01	0.04% jak2inhib								-5.13	-5.01	-3.99	-5.75	-4.97
01500737	3,7-DIMETHOXYFLAVONE	100122-20	H02	sample	20950-52-1	C17H14O4	282.29873		Pongamia pinnata	undetermined activity		-1.18	-3.33	-1.33	-2.72	-2.14
01500730	4-METHYLDAPHNETIN	100122-20	H03	sample		C10H8O4	192.17286		analog of daphnetin	undetermined activity		-0.36	0.23	-0.03	2.10	0.48
01500739	CHRYSIN DIMETHYL ETHER	100122-20	H04	sample	21392-57-4	C17H14O4	282.29873		Boesenburgia pandurata, Leptosermum scoparium	undetermined activity		-0.19	-0.65	-1.10	-0.06	-0.50
01500746	NARINGENIN	100122-20	H05	sample	480-41-1	C15H12O5	272.25989	antiulcer, gibberellin antagonist	widely distributed in plants	experimental		-0.55	0.03	-0.44	1.24	0.07
01500752	QUERCITRIN	100122-20	H06	sample	522-12-3	C21H20O11	448.38695	antihemmorrhagic	widespread in plants	experimental		-5.42	-2.53	-0.02	-0.44	-2.10
01500764	KINETIN	100122-20	H07	sample	525-79-1	C10H9N5O	215.21613	auxin, plant growth regulator, plant cell division promotor	yeast	experimental	JACS 77: 1392, 2662 (1955); 78: 1375, 3693 (1956); 82:	0.30	0.11	-0.45	-1.79	-0.46
01500765	NARINGIN	100122-20	H08	sample	10236-47-2	C27H32O14	580.54769	antihaemorrhagic, antiinflammatory	Citrus spp	experimental	JACS 61: 175 (1939); Tetrahedron 19: 773 (1963)	0.04	0.00	-0.30	0.54	0.07
01500814	CANTHARIDIN	100122-20	H09	sample	56-25-7	C10H12O4	196.20474	irritant	active principle of cantharides and other insects	experimental	J Am Chem Soc 63: 3167 (1941)	-1.03	-0.97	1.91	-2.14	-0.56

												Screen Score (z-score)				
Compound ID	MolNAME	plate	position	Content	cas#	Formula	MolWt	Bioactivity	Source	Status	Reference	rep1	rep2	rep3	rep4	Average
01500815	BETULIN	100122-20	H10	sample	473-98-3	C30H50O2	442.7318		Betula spp	undetermined activity	J Am Chem Soc 78: 2312 (1956)	0.50	0.73	-0.55	0.00	0.17
01500817	CARMINIC ACID	100122-20	H11	sample	1260-17-9	C22H20O14	508.3963		Dactylopius coccus (cochineal)	undetermined activity		1.38	0.47	5.74	0.99	2.15
	empty	100122-20	H12	sample								NA	NA	NA	NA	NA
	empty	100122-21	A01	sample								NA	NA	NA	NA	NA
01500821	BICUCULLINE (+)	100122-21	A02	sample	485-49-4	C20H17NO6	367.36159	GABAa antagonist	Dicentra cucullaria, Corydalis spp	experimental	Coll Czech Chem Commun, 29, 2328 (1964); Nature	-1.75	-1.34	1.25	-2.26	-1.03
01500832	CARYLOPHYLLENE OXIDE	100122-21	A03	sample	1139-30-6	C14H22O	206.33084		clove, cinnamon and many other oils	undetermined activity		-1.20	-2.17	-2.82	-2.24	-2.11
01500835	URSOCHOLANIC ACID	100122-21	A04	sample	546-18-9	C24H40O2	360.5852		Abrus pecatorius	undetermined activity	Steroids 23:357 (1974)	-12.11	-4.31	-2.60	-3.50	-5.63
01500836	CEPHALOSPORIN C SODIUM	100122-21	A05	sample	61-24-5	C16H20N3NaO8S	437.4069	antibacterial	Cephalosprium acremonium	BAN		-0.60	-1.63	-0.95	-1.38	-1.14
01500842	CARYOPHYLLENE [t(-)]	100122-21	A06	sample	87-44-5	C14H22	190.33144		clove, cinnamon and many other oils	undetermined activity		-6.70	-1.40	-1.04	2.95	-1.55
01500849	CHOLEST-5-EN-3-ONE	100122-21	A07	sample	601-54-7	C27H44O	384.65113		animal fats; mp 79-80 C	undetermined activity	J Am Chem Soc 75: 3500 (1953)	-4.24	-1.61	-1.29	-3.20	-2.59
01500851	CHOLEST-4,6-DIEN-3-ONE	100122-21	A08	sample	566-93-8	C27H42O	382.63519		Pinus spp	undetermined activity	Hoppe Seyler's Z Physiol Chem 245:80 (1936); J Chem Soc	-8.09	0.06	-1.17	0.14	-2.27
01500854	7-OXOCHOLESTEROL	100122-21	A09	sample	566-28-9	C27H44O2	400.65053		Cliona copiosa	undetermined activity	Annalen 543, 240 (1940); JACS 71:2226 (1949); J Chem	-6.99	-0.54	-0.63	1.29	-1.72
01500855	CITRULLINE	100122-21	A10	sample	627-77-0	C6H13N3O3	175.18881		Citrullis vulgaris and the alga Grateloupia filicina	undetermined activity	Tetrahedron 40: 235 (1984)	2.29	-0.14	0.50	-0.36	0.57
01500856	CHOL-11-ENIC ACID	100122-21	A11	sample		C24H36O3	372.55272		semisynthetic	undetermined activity		1.10	-0.93	3.61	-2.32	0.36
	0.04% JAK2inhib	100122-21	A12	0.04% jak2inhib								-0.39	-2.55	-1.85	-2.90	-1.92
	0.04% DMSO	100122-21	B01	0.04% dmso								-2.41	-1.29	-1.34	1.19	-0.96
01500857	BILIRUBIN	100122-21	B02	sample	635-65-4	C33H36N4O6	584.67807		pigment mamalian gallstones, blood and urine	undetermined activity		-1.01	-0.75	0.90	-0.52	-0.34
01500860	S-ISOCORYDINE (+)	100122-21	B03	sample	475-67-2	C20H23NO4	341.41061	sedative, cholinergic	Dicentra canadensis, Artabotyris suaveoleus and Cordyalis	experimental	J Org Chem 19:1774 (1954); Aust J Chem 20: 1277 (1967)	-1.31	-1.45	-2.45	-2.63	-1.96
01500861	CORALYNE CHLORIDE	100122-21	B04	sample	38989-38-7	C22H22ClNO4	399.87794	cytostatic, intercalating agent	semisynthetic	experimental	Int J Immunopharmacol 6: 587 (1984); Nucleic Acids Res	-0.67	-2.06	-1.70	-1.91	-1.59
01500862	BOLDINE	100122-21	B05	sample	476-70-0	C19H21NO4	327.38352		Peumus boldus	undetermined activity	J Chem Soc Perkin I 1977: 706	-0.31	1.22	-0.29	-2.58	-0.49
01500864	HARMALINE	100122-21	B06	sample	304-21-2	C13H14N2O	214.26933	CNS stimulant, antiparkinsonian agent	Peganium harmala	experimental	Ber 63: 120 (1930)	-0.02	0.02	-0.49	-2.60	-0.78
01500866	HARMANE	100122-21	B07	sample	486-84-0	C12H10N2	182.2269	intercalating agent, sedative	Arariba rubra.	experimental	J Am Chem Soc 76: 2792 (1954)	0.93	0.08	0.65	-2.13	-0.12
01500869	MIMOSINE	100122-21	B08	sample		C8H10N2O4	198.1799	depilatory agent	Mimosa and Leucena spp	experimental	Z Physiol Chem 244: 153 (1936)	0.75	3.22	2.07	-2.03	1.00
01500871	NORHARMAN	100122-21	B09	sample	244-63-3	C11H8N2	168.19981	plant growth inhibitor; mutagen	Chrysophyllum and Nocardia spp	experimental	J Chem Soc 119: 1602 (1921); 1929: 2926; Phytochemistry	1.30	0.74	-0.65	-3.58	-0.55
01500876	CORYNANTHINE	100122-21	B10	sample	123333-62-0	C21H26N2O3	354.45297		bark of Pseudocinchona africana Chev.	undetermined activity	Helv Chim Acta 9: 1207 (1951)	2.33	-0.86	-0.71	-2.66	-0.48
01500877	KYNURAMINE	100122-21	B11	sample		C9H12N2O	164.20879		urine of various animals	undetermined activity	Z Naturforsch 8b: 454 (1953)	1.97	-0.03	0.10	-0.98	0.27
	0.04% JAK2inhib	100122-21	B12	0.04% jak2inhib								-0.02	-2.47	-1.74	-1.12	-1.34
	0.04% DMSO	100122-21	C01	0.04% dmso								-2.25	-1.22	-1.33	-2.01	-1.70
01500879	KYNURENINE	100122-21	C02	sample		C10H12N2O3	208.21874		mamalian urine	undetermined activity	J Am Chem Soc 71: 4158 (1955)	-1.15	-0.91	1.34	-1.13	-0.46
01500899	ESCULETIN	100122-21	C03	sample	305-01-1	C9H6O4	178.14577	antifungal	Aesculus and Solanaceae spp	experimental		-2.31	-0.30	-0.88	-0.58	-1.02
01500904	CHOLIC ACID, METHYL ESTER	100122-21	C04	sample	1448-36-8	C25H42O5	422.61049		acid as primary bile constituent	undetermined activity		2.27	0.41	0.85	0.03	0.89
01500984	GINKGOLIDE A	100122-21	C05	sample	15291-75-5	C20H24O11	440.40768	antibacterial	Ginkgo biloba	experimental	J Am Chem Soc 93: 3546 (1971)	2.78	0.50	-0.04	-0.47	0.69
01500986	GITOXIN	100122-21	C06	sample	4562-36-1	C41H64O14	780.95883	cardiotonic	Digitalis spp.	experimental	Chem Pharm Bull 18: 1080 (1970); Mol Pharmacol 11:653	-5.35	-2.90	-3.56	-3.25	-3.77
01500989	18alpha-GLYCYRRHETINIC ACID	100122-21	C07	sample		C30H46O4	470.69872	antiinflammatory	epimer of aglycone Glycyrrhiza glabra	experimental		-2.35	-0.97	-0.40	0.42	-0.82
01501012	3-HYDROXYFLAVONE	100122-21	C08	sample	577-85-5	C15H10O3	238.24515		cabbage	undetermined activity		0.95	0.04	-0.10	0.61	0.37
01501019	BERBAMINE HYDROCHLORIDE	100122-21	C09	sample	478-61-5 (berbamine)	C37H42Cl2N2O6	681.66309	antihypertensive, skeletal muscle relaxant	Berberis spp	experimental	J Indian Chem Soc 28: 225 (1951); 30: 705 (1953); Aust J	0.87	1.61	-0.43	-1.08	0.25
01501107	PICROTOXININ	100122-21	C10	sample	17617-45-7	C15H16O6	292.29117	convulsant, GABA receptor antagonist, ichthyotoxin	Anamirta cocculus, Menispermum cocculus	experimental	JACS 57: 1111 (1935); 79: 5550 (1957); Chem Rev 67: 441	1.91	2.22	0.80	-1.76	0.79
01504027	CYTISINE	100122-21	C11	sample	485-35-8	C11H14N2O	190.24703	antiinflammatory, respiratory stimulant	Thermopsis lanceolata	INN	J Chem Soc 1932: 2778; Martindale 28th Ed, 1700 (1982)	2.29	0.50	0.43	-1.23	0.50
	0.11% JAK2inhib	100122-21	C12	0.11% jak2inhib								-1.33	-3.43	-0.09	-2.21	-1.76
	0.11% DMSO	100122-21	D01	0.11% dmso								-3.26	3.56	1.03	-0.38	0.24
01501197	PRIMULETIN	100122-21	D02	sample	491-78-1	C15H10O3	238.24515		Primula spp	undetermined activity	Helv Chim Acta 24:297 (1941)	2.26	-0.04	0.18	-0.32	0.52
01501207	KINETIN RIBOSIDE	100122-21	D03	sample		C15H17N5O5	347.33324		semisynthetic	undetermined activity		-0.58	1.87	0.47	-0.14	0.41
01501208	KARANJIN	100122-21	D04	sample	521-88-0	C17H10O4	278.26685		Derris and Tephrosia spp.	undetermined activity	Indian Acad Sci 17: 16 (1943)	2.28	1.20	1.24	-0.18	1.13
01501212	LINALOOL (+)	100122-21	D05	sample		C10H18O	154.25436		Mentha arvensis and related essential oils	undetermined activity		0.77	-0.75	0.16	1.12	0.33
01502113	AZASERINE	100122-21	D06	sample	115-02-6	C5H7N3O4	173.12924	antineoplastic, amino acid antagonist	Streptomyces fragilis	USAN, INN		0.92	2.21	0.65	-0.21	0.89
00330058	CHLORPYRIFOS	100122-21	D07	sample	2921-88-2	C9H11Cl3NO3PS	350.58972	insecticide	synthetic; DURSBAN	BAN		0.14	-0.19	0.07	0.52	0.14
01502223	RESVERATROL	100122-21	D08	sample	501-36-0	C14H12O3	228.24994	antifungal, antibacterial	Veratrum grandiflorum, Pinus sibirica, Vitis vinifera, Arachis	experimental	Aust J Chem 24:2427 (1971); Tet Lett 1972:2965;	1.12	1.62	1.19	0.55	1.12
01502231	CELLOBIOSE (D[+])	100122-21	D09	sample	528-50-7	C12H22O11	342.30254		enzymatic hydrolysis of cellulose	undetermined activity	J Am Chem Soc 75: 1916 (1953)	-0.45	0.22	2.80	-1.91	0.16
01502235	ASTAXANTHIN	100122-21	D10	sample	71772-51-5	C40H52O4	596.85804		Carotenoid pigment plant and animal sources	undetermined activity	Nature 184: 1714 (1959)	-0.08	-0.53	0.55	2.26	0.55
01502237	HARMOL HYDROCHLORIDE	100122-21	D11	sample	40580-83-4	C12H11ClN2O	234.68727	MAO inhibitor	common plant alkaloid	experimental	Phytochemistry 19:1573 (1980)	1.09	-0.07	0.04	-1.71	-0.16
	0.11% JAK2inhib	100122-21	D12	0.11% jak2inhib								-1.39	-4.26	-4.14	-4.10	-3.47
	0.11% JAK2inhib	100122-21	E01	0.11% jak2inhib								-4.72	-3.21	-4.22	1.95	-2.55
01502238	DEOXYADENOSINE	100122-21	E02	sample	16373-93-6	C10H13N5O3	251.24681		synthetic	undetermined activity		0.46	0.60	-0.36	0.28	0.25
01502242	SCOPOLETIN	100122-21	E03	sample	92-61-5	C10H8O4	192.17286	NO synthesis (inducible) inhibitor, anticoagulant	Scopolia spp.	experimental	J Org Chem 26: 1215 (1961)	0.58	0.37	-0.13	0.48	0.33
01502244	AMYGDALIN	100122-21	E04	sample	29883-15-6	C20H27NO11	457.43829	antiinflammatory, experimental antineoplastic	Rosaceae spp	experimental		0.80	0.18	-1.08	2.98	0.72
01502253	HEMATEIN	100122-21	E05	sample	475-25-2	C16H12O6	300.27044		logwood, Haematoxylon spp.	undetermined activity	J Chem Soc 93: 115 (1908)	0.91	1.37	-0.77	1.16	0.67
01502247	FISETIN	100122-21	E06	sample	528-48-3	C15H10O6	286.24335	antioxidant	Rhus and Acacia spp.	experimental	Biochem J 77: 315 (1960)	-1.11	-0.07	1.07	-0.29	-0.10
01502252	MONOCROTALINE	100122-21	E07	sample	315-22-0	C16H23NO6	325.36481	antineoplastic, insect sterilant	Crotalaria spp	experimental	J Am Chem Soc 72: 158 (1950)	-0.44	-0.08	-0.50	0.34	-0.17
01502261	QUEBRACHITOL	100122-21	E08	sample	642-38-6	C7H14O6	194.18603		common in dicotyledons	undetermined activity		0.98	0.12	1.55	1.12	0.94
01503346	URIDINE TRIPHOSPHATE TRISODIUM	100122-21	E09	sample	19817-92-6	C9H12N2Na3O15P3	550.09119	psychostimulant	yeast	experimental	JACS 75:5449 (1953); 105:115 (1983)	0.72	2.25	0.22	-1.57	0.40
01503427	ALOIN	100122-21	E10	sample	5133-19-7	C21H22O10	434.40349	cathartic, laxative	aloe	BAN	J Chem Soc 1932: 2573	1.85	0.29	1.04	2.19	1.34
01503620	SAFROLE	100122-21	E11	sample	94-59-7	C10H10O2	162.19	anesthetic (topical) and antiseptic, pediculicide	sassafras officinale	experimental	J Org Chem 13: 448 (1948)	1.94	-0.64	1.70	0.00	0.75
	0.11% DMSO	100122-21	E12	0.11% dmso								0.69	-1.25	0.37	-1.40	-0.40
	0.11% JAK2inhib	100122-21	F01	0.11% jak2inhib								-4.48	-1.68	-3.41	1.77	-1.95
01503639	RAUWOLSCINE HYDROCHLORIDE	100122-21	F02	sample	6211-32-1	C21H27ClN2O3	390.91394	alpha2 adrenergic antagonist	Rauwolfia, Aspidosperma and Vinca spp	experimental		-0.63	-0.39	-0.83	-0.66	-0.63
01503640	PARTHENOLIDE	100122-21	F03	sample	20554-84-1	C15H20O3	248.32485	5HT antagonist, antineoplastic, smooth muscle relaxant	Chrysanthemum parthenium, Michelia champaca	experimental	Tetrahedron 21:1509 (1965); Phytochemistry 17:957 (1978);	-0.14	1.94	-0.23	1.27	0.71
01503802	AZADIRACHTIN	100122-21	F04	sample	11141-17-6	C35H44O16	720.73133	antifeedant, insecticide	Melia azadirach and Azadirachta indica	experimental	Phytochemistry 12: 391 (1973)	1.34	1.71	0.31	1.72	1.27
01503805	DESACETYLCOLFORSIN	100122-21	F05	sample	64657-20-1	C20H32O6	368.47444		Coleus forskohlii; 8,13-epoxy-1,6,7,9-tetrahydroxy-14-labden-11-	undetermined activity		-0.04	-1.40	0.70	-0.23	-0.24
01503815	CEVADINE	100122-21	F06	sample	62-59-9	C32H49NO9	591.74863	antihypertensive	Veratrum alba; contains 20% veratridine	experimental		-1.64	0.10	-0.65	-0.05	-0.56
01503922	TRYPTAMINE	100122-21	F07	sample	61-54-1	C10H12N2	160.22054	psychotropic	Acacia spp, Lens esculenta, Prosopsis juliflora and fungi	experimental	J Chem Soc 99:270 (1911); J Sci Technol Sect B 25:157	-0.84	0.38	0.71	0.29	0.13
01503983	BATYL ALCOHOL	100122-21	F08	sample	544-62-7	C21H44O3	344.58303		fish oils	undetermined activity		-1.70	0.04	-0.12	0.69	-0.27
01503986	CAFESTOL ACETATE	100122-21	F09	sample	81760-48-7	C22H30O4	358.482		coffee bean oil	undetermined activity	Chem Ber 96: 2489 (1963); Tetrahedron 20: 1339 (1964)	0.47	-0.78	-0.42	-1.86	-0.65
01503987	CAFFEIC ACID	100122-21	F10	sample	331-39-5	C9H8O4	180.16171		widespread in plants	undetermined activity		0.43	0.56	-0.24	1.09	0.46
01503989	GLUCITOL-4-GUCOPYANOSIDE	100122-21	F11	sample		C12H24O11	344.31848		derivative cotton	undetermined activity		1.75	-0.38	-0.90	0.33	0.20
	0.04% DMSO	100122-21	F12	0.04% dmso								2.02	0.56	0.49	0.20	0.82
	0.04% JAK2inhib	100122-21	G01	0.04% jak2inhib								-3.94	-3.70	-2.62	-1.99	-3.06
01503990	CONESSINE	100122-21	G02	sample	5913-82-6, 546-06-5	C24H40N2	356.5998		Holarrhena spp	undetermined activity		-2.43	-0.67	-2.14	1.66	-0.89
01503994	CONVALLATOXIN	100122-21	G03	sample	508-75-8	C29H42O10	550.65209	cardiotonic	Convallaria majalis	experimental	Phytochemistry 13: 1805 (1974)	-3.04	-3.32	-2.24	-1.47	-2.52
01504001	CAFESTOL	100122-21	G04	sample	469-83-0	C20H28O3	316.44436		coffee bean oil	undetermined activity	Chem Ber 96: 2489 (1963); Tetrahedron 20: 1339 (1964)	-0.43	-0.35	-0.97	0.01	-0.43
01504005	TRIPTOPHENOLIDE	100122-21	G05	sample	74285-86-2	C20H24O3	312.41248		Tripterygium wilfordii	undetermined activity	Chin Chem Lett 5:217 (1994)	-0.19	-0.10	1.82	3.31	1.21
01504013	UBIDECARENEONE	100122-21	G06	sample	303-98-0	C59H90O4	863.37275	cardiovascular agent	widespread in aerobic organisms (solubility limits concentration to	experimental		-2.06	0.58	-0.33	0.88	-0.23
01504015	alpha-MANGOSTIN	100122-21	G07	sample	6147-11-1	C24H26O6	410.47122		Garcinia mangostana, Hydnocarpus octandra, H venenata	undetermined activity	JACS 80:1691 (1958); Aust J Chem 23:2539 (1970);	0.00	0.71	-0.64	0.00	0.01
01504016	HEDERAGENIN	100122-21	G08	sample	465-99-6	C30H48O4	472.71466		Clematis, Hedera spp	undetermined activity		-2.62	-0.68	0.41	-0.35	-0.81
01504018	HEDERACOSIDE C	100122-21	G09	sample		C59H96O26	1221.40737		Hedera helix	undetermined activity	(structure tentative)	1.17	0.90	0.48	-1.82	0.18
01504020	EVOXINE	100122-21	G10	sample	522-11-2	C18H21NO6	347.37117		Evodia xanthoxyloides	undetermined activity	Aust J Sci Res, Ser A 5:401 (1952); Planta Med 42:400 (1981)	2.21	0.75	2.62	1.58	1.79
01504022	SALSOLIDINE	100122-21	G11	sample	493-48-1	C12H17NO2	207.27479	antihypertensive	Salsola Richteri	experimental	Bull Soc Chim France 4L1265 (1937); Phytochemistry 12:193 (1973); 13:1628 (1974); J Nat Prod 39:175,197 (1976); 45:277 (1982)	1.54	-0.15	-0.15	0.68	0.48
	0.04% DMSO	100122-21	G12	0.04% dmso								1.27	0.88	-0.15	2.36	1.09
	0.04% JAK2inhib	100122-21	H01	0.04% jak2inhib								-3.83	-3.96	-3.45	-2.38	-3.40
01504044	RESVERATROL 4'-METHYL ETHER	100122-21	H02	sample	33626-08-3	C15H14O3	242.27703		derivative	undetermined activity	Chem Pharm Bull 25:2300 (1977); Phytochemistry 32:1083 (1993)	-1.09	-1.71	-1.12	2.52	-0.35
01504024	LAGOCHILIN	100122-21	H03	sample	23554-81-6	C20H36O5	356.50692		Lagochilus inebrians	undetermined activity	Chem Commun 1974:249	1.91	3.97	-1.00	0.54	1.36
01504025	SALSOLINE	100122-21	H04	sample	89-31-6	C11H15NO2	193.2477	antihypertensive, antihistamine	Salsola richteri	experimental	Phytochemistry 12:193 (1973); J Med Chem 33:147 (1990)	0.00	-0.06	-0.85	2.32	0.36
01504075	RHOIFOLIN	100122-21	H05	sample	17306-46-6	C27H30O14	578.53175		Chorisia, Citrus and Rhus spp	undetermined activity	Experientia 20: 562 (1964); Chem Ber 102: 2083 (1969)	0.57	0.85	2.34	1.73	1.37
01504023	THERMOPSINE PERCHLORATE	100122-21	H06	sample	486-90-8(base)	C15H21ClN2O5	344.79802		Anabasis aphylla, Thermopsis spp	undetermined activity	Ber 66:625 (1933); Can J Chem 29:13 29, 22 (1951)	-0.14	-0.02	1.16	0.34	0.34
01504053	FUCOSTANOL	100122-21	H07	sample	83-45-4	C29H52O	416.73719		Calendula officinalis	undetermined activity	J Am Chem Soc 60: 2431 (1938)	-3.55	0.10	1.82	0.34	-0.32
01504079	TOMATINE	100122-21	H08	sample	86273-92-9	C47H79NO21	994.14778	antifungal, antibacterial, antiinflammatory agent	Solanum spp.	experimental	Chem Ber 90: 203 (1957)	-3.48	2.22	2.93	0.45	0.53
01504068	DIOSMETIN	100122-21	H09	sample	520-34-3	C16H12O6	300.27044		Valeriana, Digitalis spp	undetermined activity	J Indian Chem Soc 31: 565 (1954)	-0.61	-0.07	0.10	0.10	-0.12
01504041	TRIACETYLRESVERATROL	100122-21	H10	sample	42206-94-0	C20H18O6	354.36286		Kirkpatrickia variolosa (sponge)	undetermined activity	Aust J Chem 24:2427 (1971); Phytochemistry 32:1083 (1993); J Nat Prod 58:1958 (1995)	2.07	1.13	1.88	1.83	1.73
01504065	MYRICETIN	100122-21	H11	sample	529-44-2	C15H10O8	318.24215	antiHIV, topoisomerase II inhibitor	Myrica spp	experimental	J Chem Soc 81: 203 (1902); PNAS 97: 4790 (2000)	2.05	0.44	0.57	0.64	0.92
	empty	100122-21	H12	sample								NA	NA	NA	NA	NA
	empty	100122-22	A01	sample								NA	NA	NA	NA	NA
01504081	BETULINIC ACID	100122-22	A02	sample	472-15-1	C29H46O3	442.68817	antineoplastic	Betula spp.	experimental	Acta Chem Scand 19: 1661 (1965); Nature Medicine 1: 1046 (1995)	-5.50	-2.58	1.75	-1.05	-1.84
01504115	HIERACIN	100122-22	A03	sample	1621-84-7	C15H10O7	302.24275		Ginkgo biloba, Hieracium pilosella and Isoetes spp	undetermined activity	Chem Pharm Bull 32:4935 (1984)	-0.79	-1.41	-1.54	-1.53	-1.32
01504118	DIFFRACTAIC ACID	100122-22	A04	sample	436-32-8	C20H22O7	374.39414		Usnea spp	undetermined activity	Chem Ber 65: 175, 1668 (1932)	-2.25	2.73	-0.72	-0.63	-0.22
01504123	10-HYDROXYCAMPTOTHECIN	100122-22	A05	sample	19685-09-7	C20H16N2O5	364.36092	antineoplastic	Camptotheca acuminata	experimental	J Med Chem 29:1553 (1986); J Nat Prod 53:186 (1990)	0.45	1.96	1.62	1.93	1.49
01504124	LINAMARIN	100122-22	A06	sample	554-35-8	C10H17NO6	247.25009		Linum usitatissimum, Manihot utilissimus	undetermined activity	Phytochemistry 4: 127 (1965)	0.94	-0.44	1.05	0.52	0.52
01504130	3,7-DIHYDROXYFLAVONE	100122-22	A07	sample	492-00-2	C15H10O4	254.24455		Platymiscium praecox	undetermined activity	Phytochemistry 11: 3515 (1972)	0.36	-0.92	-0.30	-0.48	-0.34
01504132	6,3'-DIMETHOXYFLAVONE	100122-22	A08	sample	79786-40-6	C17H14O4	282.29873		Pimelia decora	undetermined activity	Aust J Chem 34:1779 (1981)	0.82	-0.41	0.35	0.42	0.29
01504149	LEUCODIN	100122-22	A09	sample	17946-87-1	C15H18O3	246.30891	antiinflammatory, hypolipidemic	Artemesia & Achillea spp	experimental	Pharmacol Toxicol USSR 54: 35 (1991); J Gen Chem 33: 2663 (1963)	0.11	-1.40	-0.22	-0.65	-0.54
01504155	APHYLLIC ACID	100122-22	A10	sample	642-67-1	C15H26N2O2	266.38667		Anabasis aphylla	undetermined activity		1.53	0.25	3.88	0.02	1.42
01504156	ANABASAMINE HYDROCHLORIDE	100122-22	A11	sample	20410-87-1(base)	C16H20ClN3	289.8109		Anabasis aphylla	undetermined activity		0.85	-1.05	0.65	-1.93	-0.37
	0.04% JAK2inhib	100122-22	A12	0.04% jak2inhib								-1.09	-2.60	-1.34	-2.07	-1.78
	0.04% DMSO	100122-22	B01	0.04% dmso								-0.81	-0.69	-1.45	-0.02	-0.74
01504167	URSINOIC ACID	100122-22	B02	sample	30265-59-9	C15H16O5	276.29177		Angelica ursina	undetermined activity	Khim Prir Soedin 6:421 (1970); Chem Nat Compd (Eng Trans) 6:429 (1970)	-0.48	-0.76	0.87	0.50	0.03
01504176	RHETSININE	100122-22	B03	sample	526-43-2	C19H17N3O2	319.36624		Zanthoxylum rhetsa, Evodia rutaecarpa	undetermined activity	Tetrahedron 7: 257 (1959)	-0.78	-1.72	-2.49	-0.36	-1.34
01504180	ASARININ (-)	100122-22	B04	sample	13079-95-3	C20H18O6	354.36286	antibacterial (tuberculostatic)	Asarum & Xanthoxylum spp	experimental	Aust J Chem 1: 384 (1954); 26: 687 (1973)	1.96	0.04	-1.01	-0.14	0.21
01504185	TETRANDRINE	100122-22	B05	sample	518-34-3	C38H42N2O6	622.76824	analgesic, antineoplastic, antihypertensive, lymphotoxin	Triclisia and Cyclea spp	experimental	Phytochemistry 14: 2524 (1975); Life Sci 68: 841 (2001)	0.09	0.84	-0.05	0.84	0.43
01504200	ISOLIQUIRITIGENIN	100122-22	B06	sample	961-29-5	C15H12O4	256.26049	aldose reductase inhibitor, antineoplastic, antiinflammatory	widespread in Fabaceae	experimental	J Chem Soc 1953: 2183; Eur J Biochem 212: 87 (1992)	0.02	0.18	0.05	-2.18	-0.48
01504204	CANTHAXANTHIN (euglenanone)	100122-22	B07	sample	514-78-3	C40H52O2	564.85924		Cantharellus cinnabarinus	undetermined activity	Arch Biochem Biophys 91: 271 (1960); Biochem J 101: 735 (1966)	0.75	-0.27	0.15	0.88	0.38
01504234	18-AMINOABIETA-8,11,13-TRIENE SULFATE	100122-22	B08	sample		C20H33NO4S	383.55431		derivative of abietic acid	undetermined activity		-0.11	-0.36	0.46	-0.17	-0.05

												Screen Score (z-score)				
Compound ID	MolNAME	plate	position	Content	cas#	Formula	MolWt	Bioactivity	Source	Status	Reference	rep1	rep2	rep3	rep4	Average
01504410	PICROPODOPHYLLIN	100122-22	B09	sample	477-47-4	C22H22O8	414.41584	Insulin growth factor 1 receptor inhibitor, antineoplastic	Podophylum peltatum; epimer of podophyllotoxin; 10% cytotoxicity of podophyllotoxin	experimental	JACS 73: 2909 (1951); 75: 1308 (1953); J Med Chem 32: 604 (1989); Pharmacol Ther 59: 163 (1993); Cancer Res 64:236 (2004); Int. J. Cancer 121:1857 (2007)	0.11	-0.74	0.67	-0.01	0.01
01504519	7-HYDROXYFLAVONE	100122-22	B10	sample	6665-86-7	C15H10O3	238.24515	antifungal, analgesic	Virola spp	experimental	Mycol Res 101:920 (1997); Indian J Exp Biol 38:1172 (2000); Chem Pharm Bull 52:1255 (2004)	0.52	-0.41	0.49	-2.43	-0.46
01504614	GYROMITRIN	100122-22	B11	sample	16568-02-8	C4H8N2O	100.12116	hepatotoxin, carcinogen	Gyromitra mushroom species; metabolized to to the hepatotoxin formylmethylhydrazine	experimental	Arch Pharmazie 302:294 (1968); Z Lebensm Unters Forsch 160:325 (1976); Cancer Res 37:3458 (1977); J Appl Toxicol 11:235 (1991)	0.47	-0.66	0.83	0.15	0.20
	0.04% JAK2inhib	100122-22	B12	0.04% jak2inhib								0.38	-1.75	2.13	-3.03	-0.57
	0.04% DMSO	100122-22	C01	0.04% dmso								-1.28	-0.05	-1.16	0.05	-0.61
01504800	CHRYSANTHEMIC ACID	100122-22	C02	sample	10453-89-1	C10H16O2	168.23782	esters as insecticide	esters as constituent of pyrethrum flowers	experimental	J Chem Soc 1945: 283	1.90	-0.88	-1.52	-0.10	-0.15
01505002	LAPPACONITINE	100122-22	C03	sample	32854-75-4	C32H44N2O8	584.71608	analgesic, antiarrhythmic	Aconitum spp and Delphinium cashmirianum	experimental	Tet Lett 1964:2711; Can J Chem 45:969 (1967); J Nat Prod 42:615 (1979); Planta Medica 64:22 (1998); Mol Pharm 59:183 (2001)	-0.02	-0.59	-1.02	0.09	-0.39
01505006	DIHYDROMYRISTICIN	100122-22	C04	sample	607-91-0	C11H14O3	194.23243	GSH transferase inducer	derivative; myristicin	experimental	J Agr Food Chem 40:107 (1992)	-0.39	-1.81	-0.81	-1.60	-1.15
01505010	EUPHORBIASTEROID	100122-22	C05	sample	28649-59-4	C32H40O8	552.6708		Euphorbia lathyris, Macaranga tanarius	undetermined activity	Tet Lett 1970:2241, 3071; Experientia 27:1393 (1971)	-0.35	-0.43	-0.57	-1.07	-0.60
01505018	ASTRAGALOSIDE IV	100122-22	C06	sample	84687-43-4	C41H68O14	784.99071		Astragalus species	undetermined activity	Chem Pharm Bull 31:689, 698, 709, 716 (1983)	-6.58	0.71	-0.10	0.06	-1.48
01505030	DEMETHYLNOBILETIN	100122-22	C07	sample	2174-59-6	C20H20O8	388.3776		Citrus, Sideritis, Heteropappus and Thymus spp; Mentha piperita, Amaracus pampanini	undetermined activity	Tetrahedron 8:64 (1960); Phytochemistry 11:2283 (1972); 24:1027 (1985)	-0.68	-1.11	-0.92	-1.97	-1.17
01505034	BACCATIN III	100122-22	C08	sample	27548-93-2	C31H38O11	586.64191		Taxus baccata	undetermined activity	J Org Chem 51:3239 (1986)	0.06	-0.12	0.15	0.05	0.04
01505080	SALVINORIN A	100122-22	C09	sample	83729-01-5	C23H28O8	432.47481	k-opiod receptor agonist, psychotropic	Salvia divinorum	experimental	J Chem Soc 1982:2505; J Org Chem 49:4716 (1984); Chem Lett 1990:2015	-0.04	-1.09	1.25	-1.24	-0.28
01505121	11a-ACETOXYPROGESTERONE	100122-22	C10	sample	2268-98-6	C23H32O4	372.50909	metabolite of progesterone	semisynthetic	undetermined activity	Endocrinology 53:221 (1953); Biochem Biophys Res Commun 319:677 (2004)	-0.22	-1.30	-1.40	-2.04	-1.24
01505123	21-ACETOXYPREGNENOLONE	100122-22	C11	sample	566-78-9	C23H34O4	374.52503	precursor in corticoid biosynthesis, derivative	semisynthetic	undetermined activity	PNAS 94:4895 (1997)	2.30	-0.87	0.29	-0.57	0.29
	0.11% JAK2inhib	100122-22	C12	0.11% jak2inhib								-2.72	-2.13	2.20	-2.61	-1.31
	0.11% DMSO	100122-22	D01	0.11% dmso								-1.47	-0.92	-0.99	2.32	-0.26
01505127	GOSSYPIN	100122-22	D02	sample	652-78-8	C21H20O13	480.38575		Gossypium spp, Hibiscus spp	undetermined activity	Proc Indian Acad Sci Sect A, 24:352, 375 (1946); 25:397 (1947)	-2.15	-0.89	-1.65	-0.36	-1.26
01505129	PLUMBAGIN	100122-22	D03	sample	481-42-5	C11H8O3	188.18461	antibacterial, antifungal, tuberculostatic; antifeedant (African army worms)	Aristea, Diospyros & Plumbago spp; Dyerophyton, Drosera, Dioncophyllum, Nepenthe, Sisyrinchium and Sparaxis tricolor	experimental	JACS 58:572 (1936); Indian J Chem 6:681 (1968); Fitoterapia 1990:387	-0.16	0.08	-1.42	-1.07	-0.64
01505130	3,4-DIMETHOXYCINNAMIC ACID	100122-22	D04	sample	14737-89-4	C11H12O4	208.21589		Piper methysticum, Veronica virginica	undetermined activity		-0.27	-1.07	-0.24	-1.79	-0.84
01505132	2',4'-DIHYDROXYCHALCONE	100122-22	D05	sample	1776-30-3	C15H12O3	240.26109	anthelmintic, antiulcer	Flemingia chappar, Ceratiola ericoides, Acacia neovernicosa, Flourensia spp	experimental	J Chem Soc 1937:1737; Tetrahedron 27:2111 (1971); Planta Med 58:389 (1992)	1.07	1.75	3.19	3.27	2.32
01505134	MANGIFERIN	100122-22	D06	sample	4773-96-0	C19H18O11	422.34871	MAO inhibitor, immunostimulant	Mangifera indica, Iris & Salacia spp, Aphloia, Athyrium, Anemarrhena, Belamcanda chinensis, Hedysarum ussuriense	experimental	J Chem Soc (C) 1966:1685; Phytochemistry 6:741, 1597 (1967); Tetrahedron 23:1363 (1967); Pharm Res 3:307 (1986); Naturwissenschaften 59:651 (1972)	-0.12	-0.19	-0.96	-0.49	-0.44
01505135	PIPLARTINE	100122-22	D07	sample	20069-09-4	C17H19NO5	317.34468	anti-asthma, antibronchitis	Piper spp	experimental	Tetrahedron 23:1769 (1967); Phytochemistry 13:2327 (1974)	0.21	0.95	3.58	1.33	1.52
01505140	2',4-DIHYDROXY-3,4',6'-TRIMETHOXYCHALCONE	100122-22	D08	sample	112572-59-5	C18H18O6	330.34056		Viscum album (glucoside)	undetermined activity	Annalen 550:146 (1942); Planta 95:133 (1970); Phytochemistry 13:1561 (1974)	-0.52	0.36	0.06	2.48	0.59
01505141	2',4-DIHYDROXYCHALCONE	100122-22	D09	sample	13323-66-5	C15H12O3	240.26109		Adhatoda vasica (as glucoside)	undetermined activity	JACS 68:697 (1946)	0.80	1.53	0.76	0.04	0.78
01505142	2',5'-DIHYDROXY-4-METHOXYCHALCONE	100122-22	D10	sample	6342-92-3	C16H14O4	270.28758		Cassia javanica	undetermined activity	Chem Abs 92:55042v (1980)	1.19	0.42	0.97	2.06	1.16
01505143	GOSSYPETIN	100122-22	D11	sample	489-35-0	C15H10O8	318.24215		widespread in plants	undetermined activity	J Chem Soc 1929:24	-0.62	-0.10	0.99	0.45	0.18
	0.11% JAK2inhib	100122-22	D12	0.11% jak2inhib								-2.75	-3.99	-0.48	-2.36	-2.40
	0.11% JAK2inhib	100122-22	E01	0.11% jak2inhib								-3.60	-1.00	-3.35	-3.05	-2.75
01505151	HARPAGOSIDE	100122-22	E02	sample	19210-12-9	C23H28O11	480.47301		Melittis melissophyllum, Harpagophytum procumbens	undetermined activity	J Chem Soc Perkin 1 1979:2473; Phytochemistry 40:1569 (1995)	-1.02	0.59	1.83	0.24	0.41
01505152	2',4'-DIHYDROXY-4-METHOXYCHALCONE	100122-22	E03	sample	81674-91-1	C16H14O4	270.28758		Bauhinia manca	undetermined activity		-30.32	-30.46	-26.93	-24.85	-28.14
01505169	DESOXYPEGANINE HYDROCHLORIDE	100122-22	E04	sample	61939-05-7	C11H13ClN2	208.69266	acetylcholinesterase inhibitor, antiParkinsonism	derivative of peganine Peganum spp	experimental	Naunyn Schmeidebergs Arch Pharmacol 363:429 (2001)	2.50	0.34	-1.14	-0.79	0.23
01505153	2',3-DIHYDROXY-4,4',6'-TRIMETHOXYCHALCONE	100122-22	E05	sample	38186-71-9	C18H18O6	330.34056		Merrillia caloxylon	undetermined activity	Annalen 550:146 (1942); Planta 95:133 (1970); Phytochemistry 13:1561 (1974)	0.55	3.70	0.30	0.40	1.24
01505174	GARLICIN	100122-22	E06	sample	2179-57-9	C6H10S2	146.2746	antineoplastic, antibacterial, apoptosis inducer, insecticide	Allium spp, Descurainia sophia	experimental	Pharmazie 20:441 (1965); J Org Chem 40:1567 (1975); Food Chem Toxicol 26:297 (1988); Cancer Res 35:3493 (1993); Bioxhim Biophys Acta 1315:15 (1996)	-0.45	0.47	-0.42	0.59	0.05
01505175	ASIATIC ACID	100122-22	E07	sample	464-92-6	C30H48O5	488.71406	wound healing, experimental carcinogen	Dipterocarpus pilosus, Dryobalanops aromatica	experimental	Bull Soc Chim Fr 1959:880; 1961:1586; J Chem Soc 1961:646; Phytochemistry 8:917 (1969)	0.63	-1.11	2.90	0.35	0.69
01505176	AURAPTENE	100122-22	E08	sample	495-02-3	C19H22O3	298.38539	antineoplastic, apoptosis inducer	Citrus aurantium, Feronia elephantum, Aegle marmelos, Libanotis intermedia	experimental	Chem Pharm Bull 1:119 (1959); Cancer Res 12:2550 (1998); Mutat Res 480:201 (2001)	0.26	-0.16	-0.82	-0.62	-0.33
01505190	L(+/-)-ALLIIN	100122-22	E09	sample	556-27-4(-)	C6H11NO3S	177.22347	antibacterial, antioxidant	Allium species	experimental	Helv Chim Acta 34:481 (1951); Phytochemistry 15:521 (1976)	2.26	0.29	0.22	0.24	0.75
01505195	EPITESTOSTERONE	100122-22	E10	sample	481-30-1	C19H28O2	288.43381	androgen	cooccurs with testosterone in mammals	experimental		1.04	0.07	0.70	0.46	0.57
01505217	RHODOCLADONIC ACID	100122-22	E11	sample	26984-15-6	C15H10O8	318.24215		Cladonia spp	undetermined activity	Can J Chem 47: 2733 (1969); 61: 2055 (1983); Phytochemistry 12: 937 (1973)	-3.62	0.50	1.31	1.96	0.04
	0.11% DMSO	100122-22	E12	0.11% dmso								0.22	-0.76	0.67	-1.33	-0.30
	0.11% JAK2inhib	100122-22	F01	0.11% jak2inhib								-2.78	-3.13	-3.37	0.82	-2.12
01505234	AVOCADYNE	100122-22	F02	sample	34524-38-4	C17H32O3	284.44279	antibacterial, antifungal	Persea spp	experimental	An Acad Brasil Cienc 42 (suplemento): 45 (1970); Tetrahedron 25:4617 (1969)	-0.17	-0.69	-0.97	-0.22	-0.51
01505240	LUPANYL ACID HYDROCHLORIDE	100122-22	F03	sample		C14H25ClN2O2	288.82055		Anabasis aphylla	undetermined activity		-0.72	1.01	-1.02	0.12	-0.15
01505242	LUPANINE PERCHLORATE	100122-22	F04	sample	550-90-3 (base)	C15H25ClN2O5	348.8299		Lupinus and Cystisus spp	undetermined activity		-0.35	-0.04	-0.58	2.70	0.43
01505254	THEANINE	100122-22	F05	sample	3081-61-6	C7H14N2O3	174.20123		Thea sinensis	undetermined activity	JACS 64: 1021 (1942); J Sci Food Agric 5: 597 (1954); Biochim Biophys Acta 39: 462 (1960)	-0.47	1.18	-0.19	-0.17	0.09
01505255	HUPERZINE A	100122-22	F06	sample	102518-79-6	C15H18N2O	242.32351	anticholinesterase, cognition enhancer	Lycopodium spp	experimental	Tet Lett 1960 (10): 26; Can J Chem 64: 837 (1986); Adv Med 1: 175 (1992)	0.71	0.89	-0.49	-0.21	0.23
01505257	ICARIIN	100122-22	F07	sample	489-32-7	C33H40O15	676.67775	hepatoprotective	Epimedium spp	experimental	Planta Med 61: 523 (1995); J Chromatog A 963: 239 (2002)	0.08	1.12	2.69	0.28	1.04
01505268	NOBILETIN	100122-22	F08	sample	478-01-3	C21H22O8	402.40469	matrix metaloproteinase inhibitor; antineoplastic	Citrus spp	experimental	J Chem Soc 1938:1004; Phytochemistry 14:309 (1975); J Cancer Res 92:1322 (2001); Biochem Pharmacol 65:2065 (2003); Mol Cancer Ther 3:839 (2004)	-0.36	-0.88	-0.54	-1.43	-0.80
01505269	TANGERITIN	100122-22	F09	sample	481-53-8	C20H20O7	372.3782		Citrus spp, Fortunella japonica	undetermined activity	Chem Ind 1957: 47; Phytochemistry 14: 309 (1975)	0.35	-0.08	0.47	-0.97	-0.06
01505278	3-HYDROXY-3',4'-DIMETHOXYFLAVONE	100122-22	F10	sample	6889-80-1	C17H14O5	298.29813		synthetic	undetermined activity	Tetrahedron 32: 565 (1976); Lebensmittelchem Gerichtl.Chem 39: 9 (1985)	0.91	2.59	1.25	-0.77	1.00
01505297	PERILLYL ALCOHOL	100122-22	F11	sample	536-59-4, 18457-55-1	C10H16O	152.23842	antineoplastic, apoptosis inducer; skin irritant, LD50(rat) 2100 mg/kg po	Ocimum gratissimum	experimental	CR Hebd Seances Acad Sci 249:306 (1959); Cancer Res 57:420 (1997)	-0.14	0.09	0.66	0.41	0.25
	0.04% DMSO	100122-22	F12	0.04% dmso								0.05	-0.33	0.36	-0.12	-0.01
	0.04% JAK2inhib	100122-22	G01	0.04% jak2inhib								-1.91	-1.51	-2.94	-1.52	-1.97
01505326	SOLANESYL ACETATE	100122-22	G02	sample		C47H76O2	673.12857		Nicotiana tabacum; Murraya exotica; Pinus spp	undetermined activity	Phytochemistry 39:1383 (1995)	-5.56	2.01	-1.29	0.01	-1.21
01505320	INDOLE-3-CARBINOL	100122-22	G03	sample	700-06-1	C9H9NO	147.17818	antineoplastic	Brassica spp	experimental	Anticancer Res 15:709 (1995); Biochem Mol Biol Int 39:1125 (1996)	0.64	0.94	0.33	0.97	0.72
01505325	SOLANESOL	100122-22	G04	sample	13190-97-1	C45H74O	631.09093		Nicotiana tabacum; Betulaprenol 9: mp 42 C	undetermined activity	Biochem J 102:313, 325 (1966); 128: 11P (1972); Nature 1966: 212, 1364; Phytochemistry 39:1383 (1995); J Nat Prod 46:174 (1983); J Org Chem 54:3390 (1989)	0.21	0.78	2.05	1.53	1.14
01505302	GENETICIN	100122-22	G05	sample	108321-42-2, 49863-47-0(base)	C20H44N4O18S2	692.71768	antibacterial	Micromonospora spp; G-418	experimental	Antimicrob Agents Chemother 6:144 (1974); J Antibiotics 28:573 (1975); 30:945 (1977); 35:1 (1982)	0.02	1.58	-0.06	0.77	0.58
01505334	SECURININE	100122-22	G06	sample	5610-40-2	C13H15NO2	217.27	GABAA receptor blocker, CNS stimulant	Securinega spp and Phyllanthus discoides	INN	Chem Ind 1962:1652; 1964:838, 1691; Naturwissenschaften 50:155 (1963)	-1.67	0.47	-2.00	-2.37	-1.39
01505345	CURCUMIN	100122-22	G07	sample	458-37-7	C21H20O6	368.38995	antiedemic, antiinflammatory, bile stimulant; antibacterial, antifungal, lipo/cyclooxygenase inhibitor	Curcuma spp	experimental	Ber 51: 1347 (1918); J Pharm Pharmacol 5: 448 (1953); Cancer Res 51:813 (1991)	2.17	1.04	-0.35	0.33	0.80
01505380	3,4',5,6,7-PENTAMETHOXYFLAVONE	100122-22	G08	sample	4472-73-5	C20H20O7	372.3782		Citrus spp	undetermined activity		1.49	0.21	-0.16	-0.09	0.36
01505381	SINENSETIN	100122-22	G09	sample	2306-27-6	C20H20O7	372.3782		Citrus spp	undetermined activity		-0.42	-0.13	-0.58	-0.55	-0.42
01505382	5-HYDROXY-2',4',7,8-TETRAMETHOXYFLAVONE	100122-22	G10	sample	123316-61-0	C19H18O7	358.35111		Citrus spp, Limnophilia rugosa	undetermined activity	Chem Pharm Bull 33:3982 (1985): Phytochemistry 28:1778 (1989)	0.99	0.78	0.28	0.22	0.57
01505383	HEXAMETHYLQUERCETAGETIN	100122-22	G11	sample	1251-84-9	C21H22O8	402.40469		Citrus spp	undetermined activity	Phytochemistry 11:2283 (1972)	-0.37	-1.38	0.99	1.16	0.10
	0.04% DMSO	100122-22	G12	0.04% dmso								0.60	0.31	1.07	2.38	1.09
	0.04% JAK2inhib	100122-22	H01	0.04% jak2inhib								-3.55	-2.58	-3.73	-2.35	-3.05
01505476	HELICIN	100122-22	H02	sample	618-65-5	C13H16O7	284.26827		Spiraea spp	undetermined activity	J Chem Soc 1930:2729; Bull Chem Soc Japan 44:526 (1971)	-1.16	-0.56	-0.75	-0.99	-0.87
01505475	RHIZOCARPIC ACID	100122-22	H03	sample	18463-11-1	C28H23NO6	469.49861		Rhizocarpon spp, Calicium hyperelium; mp 178 C	undetermined activity	JACS 72:4454 (1950); Pharmazie 23:583 (1968)	-1.35	-0.43	-2.40	-1.32	-1.37
01505745	ANDROSTERONE	100122-22	H04	sample	53-41-8	C19H30O2	290.44975	androgen	male urine	experimental	Helv Chim Acta 25:988 (1942); JACS 57:1755 (1935)	0.39	-0.20	-1.79	-0.31	-0.48
01505754	SEROTONIN HYDROCHLORIDE	100122-22	H05	sample	50-67-9	C10H13ClN2O	212.68091	neurotransmitter	CNS, GI tract, all animals, many mushrooms & plants	experimental	Agents Actions 15:627 (1984)	-1.11	0.43	-1.88	-1.49	-1.01
01505766	SYRINGIC ACID	100122-22	H06	sample	530-57-4	C9H10O5	198.17705		Glycine max; widespread in plants	undetermined activity		0.22	0.33	-0.93	0.73	0.09
01505775	AGARIC ACID	100122-22	H07	sample	666-99-9	C22H40O7	416.5599	antiperspirant	Fomes and Polyporus spp	experimental	J Bacteriol 98:23 (1969)	-4.64	1.40	0.47	0.69	-0.52
01505758	SKATOLE	100122-22	H08	sample	83-34-1	C9H9N	131.17878	insect attractant	feces, beetroot, nectandra wood	experimental		-0.70	0.31	0.57	0.85	0.26
01505757	MELIBIOSE	100122-22	H09	sample	585-99-9	C12H22O11	342.30254		yeast	undetermined activity	JACS 37:2734 (1915); J Chem Soc 113:188 (1918)	0.11	2.56	0.52	1.11	1.07
01505743	ALEURETIC ACID	100122-22	H10	sample	533-87-9	C16H32O5	304.43044		shellac constituent	undetermined activity	J Chem Soc 1944:306; J Sci Ind Res 11B:458 (1952)	-1.69	0.44	0.90	1.56	0.30
01505883	alpha-ERGOCRYPTINE	100122-22	H11	sample	511-09-1	C32H41N5O5	575.71407		ergot	experimental		-0.76	-1.54	-0.33	-0.58	-0.80
	empty	100122-22	H12	sample								NA	NA	NA	NA	NA
	empty	100122-23	A01	sample								NA	NA	NA	NA	NA
01505842	PIPERIC ACID	100122-23	A02	sample	5285-18-7	C12H10O4	218.2111		Piper nigrum, Minthostachys verticillata, Menta piperita	undetermined activity	Chem Ber 103:3752 (1970)	0.34	-0.68	-0.17	0.80	0.07
01505847	LACCAIC ACID A	100122-23	A03	sample	15979-35-8	C26H19NO12	537.44083		Coccus laccae exudate; lac resin	undetermined activity	J Chem Soc C 1967:842; Indian J Chem Soc 7:977 (1969)	0.53	1.11	1.81	0.64	1.02
01505840	STRYCHNINE METHIODIDE	100122-23	A04	sample		C22H25IN2O2	476.36115	neuromuscular blocker	derivative	experimental	J Physiol 82:432 (1934); Phytochemistry 11:2547 (1972)	-1.27	-1.47	-2.11	-2.93	-1.95
01505921	PANGAMIC ACID SODIUM	100122-23	A05	sample	20858-86-0	C10H18NNaO8	303.24666		vitamin B15	experimental	Int Rec Med 164:18 (1951); Chim Ther 7:103 (1972); Indian Drugs 20:185 (1983)	-0.51	-0.06	-2.09	-2.25	-1.23
01505909	1-HYDROXY-3,6,7-TRIMETHOXY-2,8-DIPRENYLXANTHONE	100122-23	A06	sample	15404-76-9	C26H30O6	438.5254		Garcinia mangostana	undetermined activity	JACS 80:1691 (1958); Aust J Chem 23:2539 (1970); Tetrahedron 27:3919 (1971); Phytochemistry 12:232 (1973)	-2.31	-1.42	0.42	-1.07	-1.10
01505908	MANGOSTIN TRIMETHYL ETHER	100122-23	A07	sample		C27H32O6	452.55249		derivative	undetermined activity		0.90	3.20	0.41	0.77	1.32
01506003	KAEMPFEROL	100122-23	A08	sample	520-18-3	C15H10O6	286.24335		Citrus, Delphinium, Acacia spp.	undetermined activity	J Agric Food Chem 54:2951 (2006); Am J Epidem 166: 924 (2007)	-0.81	0.90	-1.96	-0.78	-0.66
01506015	MEVALONIC ACID LACTONE	100122-23	A09	sample	503-48-0	C6H10O3	130.1448		common primary metabolite	undetermined activity	JACS 78:5273 (1956)	0.13	1.18	-1.87	-0.62	-0.29
01506020	N-ACETYLMURAMIC ACID	100122-23	A10	sample	10597-89-4	C11H19NO8	293.27598		bacterial cell wall component	undetermined activity		-0.69	0.72	-2.24	-1.13	-0.83
01506075	KAWAIN	100122-23	A11	sample	3155-48-4	C14H14O3	230.26588		Piper methysticum	experimental	Tet Letts 25:4059 (1984)	-0.17	1.18	0.10	3.26	1.09
	0.04% JAK2inhib	100122-23	A12	0.04% jak2inhib								-2.13	-0.80	-3.33	-2.39	-2.16
	0.04% DMSO	100122-23	B01	0.04% dmso								-0.24	-1.52	-1.80	-0.90	-1.12

												Screen Score (z-score)				
Compound ID	MolNAME	plate	position	Content	cas#	Formula	MolWt	Bioactivity	Source	Status	Reference	rep1	rep2	rep3	rep4	Average
01701001	ANDROSTA-1,4-DIEN-3,17-DIONE	100122-23	B02	sample	897-06-3	C19H24O2	284.40193		mammary neoplasms; cholesterol metabolite	undetermined activity	J Am Chem Soc 80: 6148 (1958); 83: 4627 (1961)	0.85	-0.30	-0.50	0.22	0.07
01701060	HYDROXYPROGESTERONE	100122-23	B03	sample	3168-01-2	C21H30O3	330.47145	progestagen	urine and blood	INN, BAN		-0.07	-0.85	1.90	-0.61	0.09
01800018	LIMONIN	100122-23	B04	sample	1180-71-8	C26H30O8	470.5242		Citrus, Evodia, Dictamnus and Luvunga spp	undetermined activity	JACS 62:1307 (1940); J Chem Soc 1961:255; Citrus Sci Technol 1:355 (1977)	-0.38	-0.61	-1.52	-0.49	-0.75
01800009	GUAIOL(-)	100122-23	B05	sample	489-86-1	C15H26O	222.37387		Callitris intratropica, Eucalyptus maculata, Drimys lancelolata	undetermined activity	Tetrahedron 13: 308 (1961)	-0.36	-1.04	-0.60	-2.85	-1.21
01800005	MENTHYL BENZOATE	100122-23	B06	sample		C17H24O2	260.37963		derivative	undetermined activity		0.28	-0.71	-1.62	-2.28	-1.08
02300253	1R,2S-PHENYLPROPYLAMINE	100122-23	B07	sample	14838-15-4	C9H13NO	151.21006	decongestant	Ephedra vulgaris (MaHuang)	experimental	Phytochemistry 16: 9 (1977)	0.03	0.47	-0.58	-0.24	-0.08
02300228	KAINIC ACID	100122-23	B08	sample	487-79-6	C10H15NO4	213.23535	glutamate receptor agonist, anthelmintic	Digenia simplex	INN, JAN		-0.25	1.00	-1.98	0.66	-0.14
00300018	LOBARIC ACID	100122-23	B09	sample		C25H28O8	456.49711		lichens of the genus Stereocaulon and others	undetermined activity	Z Naturforsch 21b: 714 (1966)	1.09	1.12	-0.08	0.88	0.75
00100743	HOMOPTEROCARPIN	100122-23	B10	sample	606-91-7	C17H16O4	284.31467		mp 82-84 C; Pterocarpus santalinus	undetermined activity	J Chem Soc 1940:787	-0.99	0.21	-1.84	-0.27	-0.72
00201092	4-METHOXYDALBERGIONE	100122-23	B11	sample	4646-86-0	C16H14O3	254.28818		Dalbergia retusa and D. nigra	undetermined activity	Phytochemistry 17: 1395 (1978); Tetrahedron 21: 2683 (1965)	-0.70	1.16	-1.04	-0.44	-0.26
	0.04% JAK2inhib	100122-23	B12	0.04% jak2inhib								-2.36	-1.46	-0.08	-1.64	-1.38
	0.04% DMSO	100122-23	C01	0.04% dmso								-0.06	-0.62	-0.21	-0.76	-0.41
00231084	UMBELLIFERONE	100122-23	C02	sample	93-35-6	C9H6O3	162.14637	antifungal, phytoalexin	Angelica, Artemisia, Coronilla, Ferula and Ruta spp	experimental	Chem Pharm Bull 19:640 (1971); Phytochemistry 14:1083 (1975)	0.22	-1.14	-1.77	-1.41	-1.03
00200441	XANTHOXYLIN	100122-23	C03	sample	90-24-4	C10H12O4	196.20474		Xanthoxylium spp, Artemisia brevifolia	undetermined activity		1.36	-1.01	-0.38	0.37	0.09
00201138	DEGUELIN(-)	100122-23	C04	sample	522-17-8	C23H22O6	394.42819	antineoplastic, antiviral, insecticide	Tephrosia & Derris spp	experimental	J Am Chem Soc 56: 2415 (1934); J Org Chem 44: 2580 (1979); Applied Microbiol 18: 660 (1969); Proc Natl Acad Sci 95: 3380 (1998); J Natl Cancer Inst 95: 291 (2003)	5.10	4.22	4.74	6.25	5.08
00201315	7,8-DIHYDROXYFLAVONE	100122-23	C05	sample	38183-03-8	C15H10O4	254.24455	vascular protectant, antihaemorrhagic	Godmania aesculifolia	experimental	J Chem Soc 1939: 956, 958; 1956:4170	-0.78	-1.53	-2.26	-2.34	-1.73
00201466	MANDELIC ACID, METHYL ESTER	100122-23	C06	sample		C9H10O3	166.17825		free acid found in Poria spp.	undetermined activity		2.51	-0.14	-0.82	-1.63	-0.02
00210186	CITRININ	100122-23	C07	sample	518-75-2	C13H14O5	250.25353	antibacterial	Penicillium citrinum	experimental	J Chem Soc 1963: 3777	-1.17	-0.38	0.46	1.52	0.11
00210477	ACTINONIN	100122-23	C08	sample	13434-13-4	C19H35N3O5	385.5079	antibacterial	Streptomyces spp.	experimental	Nature 195: 701 (1962)	-2.84	-1.70	-1.09	-2.45	-2.02
00210567	DIMETHYLCAFFEIC ACID	100122-23	C09	sample	14737-89-4	C11H12O4	208.21589		Piper methysticum, Veronica virginica	undetermined activity		0.28	1.11	-0.12	-0.76	0.12
00201697	EUPHOL	100122-23	C10	sample	514-47-6	C30H50O	426.7324		Euphorbia spp.	undetermined activity	J Chem Soc 1944:249; 1958:179	-8.68	-2.36	0.85	-0.46	-2.66
00201515	THEAFLAVIN DIGALLATE	100122-23	C11	sample	30462-35-2	C43H32O20	868.72249		pigment in black tea	undetermined activity		1.20	3.65	0.96	-0.03	1.44
	0.11% JAK2inhib	100122-23	C12	0.11% jak2inhib								-4.20	-0.88	-4.20	-2.54	-2.96
	0.11% DMSO	100122-23	D01	0.11% dmso								-1.75	-1.56	-0.09	-1.43	-1.21
01505031	GARDENIN B	100122-23	D02	sample	2798-20-1	C19H18O7	358.35111		Gardenia lucida; Brickellia, Citrus and Mentha spp	undetermined activity	Tetrahedron 21:1441, 3741 (1965); Chem Pharm Bull 28:708, 717 (1980); Phytochemistry 23:2972 (1984)	-0.37	-0.91	3.10	1.47	0.82
00107022	beta-SITOSTEROL	100122-23	D03	sample	83-46-5	C29H50O	414.72125		widespread in plants	undetermined activity	J Am Chem Soc 48: 2987 (1926)	0.48	-2.23	-0.06	0.63	-0.29
00102007	FORMONONETIN	100122-23	D04	sample	485-72-3	C16H12O4	268.27164	phytoestrogen	soyabean and clover species	experimental	J Chem Soc 1933: 274	0.31	0.61	0.42	0.42	0.44
00300545	CHRYSOPHANOL	100122-23	D05	sample	481-74-3	C15H10O4	254.24455		Cassia and Rumex spp	undetermined activity	Phytochemistry 11: 2122 (1972)	0.37	-0.45	1.87	-1.32	0.12
00240565	5,7-DIHYDROXYISOFLAVONE	100122-23	D06	sample	4044-00-2	C15H10O4	254.24455		Arachis hypogaea & Derris spp	undetermined activity	J Chem Soc 1953:1852; CA 104:165440 (1986)	0.78	-0.02	-0.20	0.33	0.22
01500294	CINEOLE	100122-23	D07	sample	470-82-6	C10H18O	154.25436	anthelminthic, antiseptic, expectorant	eucalyptus and lavender oils	experimental		0.00	0.23	-0.11	-0.55	-0.11
01501210	HUMULENE (alpha)	100122-23	D08	sample	6753-98-6	C15H24	204.35853		hops and clove oils	undetermined activity	JACS 99: 3864 (1977); J Chem Soc 1980: 311	-4.42	-0.02	0.53	0.22	-0.92
01504076	YOHIMBIC ACID HYDRATE	100122-23	D09	sample	522-87-2	C20H26N2O4	358.44122		derivative; yohimbinic acid	undetermined activity	J Chem Soc 123: 3003 (1923)	-0.24	0.06	0.32	1.00	0.29
00202175	12a-HYDROXY-5-DEOXYDEHYDROMUNDUSERONE	100122-23	D10	sample		C19H18O6	342.35171		derivative	undetermined activity		0.41	0.51	-0.75	-0.77	-0.15
00212061	PYROCATECHUIC ACID	100122-23	D11	sample	303-38-8	C7H6O4	154.12347	antioxidant	Erythraea centaurium, Gentiana lutea	experimental		0.23	1.35	-0.09	0.20	0.42
	0.11% JAK2inhib	100122-23	D12	0.11% jak2inhib								-3.93	-2.83	-2.68	0.55	-2.22
	0.11% JAK2inhib	100122-23	E01	0.11% jak2inhib								-4.09	-4.72	-3.69	-2.77	-3.82
00212097	ONONETIN	100122-23	E02	sample	487-49-0	C15H14O4	258.27643		Trifolium subterraneum	undetermined activity	Monatsh Chem 63: 201 (1933); Aust J Chem 19: 1755 (1966)	0.92	-1.22	0.32	-1.02	-0.25
00211539	ARABITOL(D)	100122-23	E03	sample	488-82-4	C5H12O5	152.14839		Lecanora sordida & other lichen & fungi	undetermined activity		-0.01	-1.37	0.57	-0.27	-0.27
00211249	7,4'-DIMETHOXYISOFLAVONE	100122-23	E04	sample		C17H14O4	282.29873		Dalbergia violaceae, Pterodon apparicioi	undetermined activity		1.33	0.80	2.49	2.70	1.83
00211012	IRIGINOL HEXAACEATATE	100122-23	E05	sample		C27H22O14	570.46799		semisynthetic	undetermined activity		0.03	0.69	1.81	-0.64	0.47
00211066	2-METHOXYRESORCINOL	100122-23	E06	sample	29267-67-2	C7H8O3	140.14001		Peltophorum africanum	undetermined activity	Phytochemistry 29:283 (1990)	-0.50	-0.29	2.37	-0.62	0.24
00300048	KOBUSONE	100122-23	E07	sample	24173-71-5	C14H22O2	222.33024		Cyperus rotundus, Sindora sumatrana	undetermined activity	J Chem Soc 1957: 2988; Chem Pharm Bull 17: 1390 (1969); 42: 138 (1994); Helv Chim Acta 66: 1843 (1983)	0.04	0.06	2.99	1.66	1.19
00240645	RETUSIN 7-METHYL ETHER	100122-23	E08	sample		C17H14O5	298.29813		derivative Dalbergia spp	undetermined activity		-0.27	-0.05	0.89	1.09	0.42
00240673	ROBUSTIC ACID	100122-23	E09	sample	5307-59-5	C22H20O6	380.4011		Derris robusta	undetermined activity	J Chem Soc 1969:365	-0.87	0.10	-0.46	0.42	-0.20
00240914	PSEUDO-ANISATIN	100122-23	E10	sample	31090-37-6	C15H22O6	298.33899	GABA antagonist	Illicium anisatum	experimental	J Am Chem Soc 74: 3211 (1952)	-0.24	2.99	2.05	-0.23	1.14
00240958	4'-METHOXYFLAVONE	100122-23	E11	sample	4143-74-2	C16H12O3	252.27224		Sapindus saponaria.	undetermined activity		0.36	1.79	-0.12	-0.19	0.46
	0.11% DMSO	100122-23	E12	0.11% dmso								-0.59	1.73	-0.56	3.93	1.13
	0.11% JAK2inhib	100122-23	F01	0.11% jak2inhib								-1.46	-2.94	-2.30	-3.98	-2.67
00240944	HAEMATOXYLIN PENTAACETATE	100122-23	F02	sample		C26H24O12	528.47398		derivative	undetermined activity	Bul Soc Chim Fr 1972: 3292	-0.72	-0.85	0.26	-0.95	-0.57
01505825	DIHYDROTANSHINONE I	100122-23	F03	sample		C18H14O3	278.31048		Salvia miltiorrhiza	undetermined activity	Acta Chim Sin36:199 (1978); J Org Chem 55:3537 (1990)	-4.07	-3.08	-3.20	-2.41	-3.19
00240862	KUHLMANNIN	100122-23	F04	sample		C17H14O5	298.29813		Manchaerium spp	undetermined activity	Phytochemistry 17: 1383 (1978)	0.00	-0.66	2.85	1.98	1.04
00203010	beta-TOXICAROL	100122-23	F05	sample	82-11-1	C23H22O7	410.42759		Derris species & isomeration of alpha-toxicarol	undetermined activity	J Chem Soc 1938: 513,734; 1939: 812	3.02	3.31	4.29	2.18	3.20
00100058	6-HYDROXYANGOLENSIC ACID METHYL ESTER	100122-23	F06	sample		C27H34O8	486.56723		Meliaceae spp	undetermined activity		-1.66	-1.07	-0.36	-0.96	-1.01
01504029	LIGUSTILIDE	100122-23	F07	sample	4431-01-0	C12H14O2	190.24418	antispasmodic, smooth muscle relaxant	Ligusticum and Angelica spp	experimental	Phytochemistry 23: 2033 (1984); J Nat Prod 58: 1047 (1995)	0.18	0.02	0.20	1.84	0.56
00300532	ANDROGRAPHOLIDE	100122-23	F08	sample	5508-58-7	C20H30O5	350.4591		Adrographis peniculata	undetermined activity	Indian J Chem 6: 252 (1968)	0.61	1.05	2.95	2.14	1.69
00100576	TRIDESACETOXYKHIVORIN	100122-23	F09	sample		C26H36O7	460.57262		Khaya spp	undetermined activity		-2.05	0.69	-0.01	-0.02	-0.35
00100465	DIHYDROFISSINOLIDE	100122-23	F10	sample		C29H38O8	514.62141		derivative of fissinolide (00100031)	undetermined activity		-0.08	-0.06	2.53	-0.72	0.42
00211224	alpha-TOXICAROL	100122-23	F11	sample	82-09-7	C23H22O7	410.42759		Derris spp	undetermined activity	J Chem Soc 1938: 513	3.40	4.07	3.60	3.92	3.75
	0.04% DMSO	100122-23	F12	0.04% dmso								0.55	1.34	-0.73	0.06	0.30
	0.04% JAK2inhib	100122-23	G01	0.04% jak2inhib								-0.42	-3.27	-2.21	-1.31	-1.80
00300549	ACETYL ISOGAMBOGIC ACID	100122-23	G02	sample		C40H46O9	670.80722		derivative	undetermined activity	J Chem Soc 1966:772; Magn Reson Chem 31: 340 (1993)	-5.38	-2.18	2.30	-0.25	-1.38
00240828	3,4-DIMETHOXYDALBERGIONE	100122-23	G03	sample	41043-20-3	C17H16O4	284.31467	induces dermatitis	Dalbergia spp, Machaerium spp	undetermined activity	Tetrahedron 21: 2697 (9165)	0.50	1.59	0.64	0.69	0.85
01500657	HYDROQUINIDINE	100122-23	G04	sample	1435-55-8	C20H26N2O2	326.44242	antiarrhythmic, antimalarial	Cinchona bark	INN		0.45	-1.00	0.89	1.16	0.37
01500639	OCTOPAMINE HYDROCHLORIDE	100122-23	G05	sample	104-14-3	C8H12ClNO2	189.64334	adrenergic agonist	salivary glands of Octopus vulgaris; also Capsicum frutescens & Cyperus spp; ND-50	INN		0.43	-0.38	0.07	0.17	0.07
00270067	STIGMASTA-4,22-DIEN-3-ONE	100122-23	G06	sample	20817-72-5	C29H46O	410.68937		plant constituent	undetermined activity	J Chem Soc 1942:391; Phytochemistry 22:2087 (1983)	-2.96	-0.41	1.56	0.75	-0.26
01500843	LATHOSTEROL	100122-23	G07	sample		C27H46O	386.66707		Austeria rubens	undetermined activity	J Biol Chem 248: 6697 (1973)	-5.87	-0.31	0.86	1.48	-0.96
01500880	TRIGONELLINE	100122-23	G08	sample	535-83-1	C7H7NO2	137.13934	antihyperglycemic	Trigonella foenumgraecum and in coffee beans	experimental	J Org Chem 26: 1318 (1961)	0.75	0.81	-0.73	4.21	1.26
01500659	ARTEMISIN	100122-23	G09	sample	481-05-0	C16H20O4	276.3354	anthelmintic	Artemesia spp	undetermined activity	J Chem Soc 1963: 5235	0.09	0.19	-0.08	-0.76	-0.14
01502108	ANTIMYCIN A (A1 shown)	100122-23	G10	sample	1397-94-0	C27H38N2O9	534.61191	antifungal, antiviral, interferes in cytochrome oxidation	Streptomyces spp	experimental		-1.88	0.13	-1.19	-0.83	-0.94
01500704	N-ACETYLPROLINE	100122-23	G11	sample		C7H11NO3	157.17062	antirheumatic	synthetic	experimental		0.17	2.06	0.72	0.03	0.75
	0.04% DMSO	100122-23	G12	0.04% dmso								-1.04	0.74	-1.16	0.28	-0.29
	0.04% JAK2inhib	100122-23	H01	0.04% jak2inhib								-2.49	-2.53	-1.81	-2.98	-2.45
01503431	CYTIDINE	100122-23	H02	sample	65-46-3	C9H13N3O5	243.22106		nucleoside	undetermined activity		0.09	-0.88	-0.40	2.10	0.23
00100325	DIGITONIN	100122-23	H03	sample	11024-24-1	C56H92O29	1229.34024		Digitalis purpurea	undetermined activity	Tetrahedron 19: 621 (1963)	-5.38	-1.85	-0.33	0.67	-1.72
01500848	LITHOCHOL-11-ENIC ACID	100122-23	H04	sample		C24H38O3	374.56866		Curvularia spp.	undetermined activity	J Chem Soc 1974: 1797	-3.05	-1.49	0.37	0.02	-1.04
00300053	BROMO-3-HYDROXY-4-(SUCCIN-2-YL)-CARYOLANE gamma-LACTONE	100122-23	H05	sample		C19H27BrO4	399.32864		derivative of caryophyllene	undetermined activity		-0.20	-0.33	-0.87	-0.29	-0.42
01501022	FARNESOL	100122-23	H06	sample	4602-84-0	C15H26O	222.37387		major component in oil of Hibiscus abelmoschus	undetermined activity	J Org Chem 28:1086 (1963)	0.31	0.20	0.27	-0.34	0.11
01501132	NEROL	100122-23	H07	sample	106-25-2	C10H18O	154.25436	weak estrogen receptor blocker	neroli and bergamot oils	experimental	J Pharm Pharmacol 54:1521 (2002)	1.91	0.83	0.36	0.57	0.92
01502094	ROSMARINIC ACID	100122-23	H08	sample	537-15-5	C18H16O8	360.32342	antiinflammatory, antithrombotic, antiplatelet, cytostatic, antiviral	Rosmarinus officinalis, Melissa officinalis, Momordica balsamina, Mentha piperita, Salvia officinalis, Teucrium scorodonia, Sanicula europaea, Coleus blumei, Thymus spp	experimental	J Org Chem 40: 1804 (1975); 41: 449 (1976); Drugs of the Future 10: 756 (1985)	-1.41	0.72	-0.09	-0.77	-0.39
01502003	BUFEXAMAC	100122-23	H09	sample	2438-72-4	C12H17NO3	223.27419	antiinflammatory, analgesic, antipyretic	synthetic; CP-1044-J3	INN, BAN, JAN		-0.22	2.17	-0.16	0.98	0.69
00100310	HECOGENIN ACETATE	100122-23	H10	sample	915-35-5	C29H44O5	472.67103		Hechtia texensis, Agave & Yucca spp	undetermined activity		-0.12	-0.49	-0.78	0.62	-0.19
01502101	PERILLIC ACID (-)	100122-23	H11	sample	7694-45-3	C10H14O2	166.22188	inhibits posttranslational cys isoprenylation, blocks G-protein	Salvia dorisiana	experimental	J Am Chem Soc 72: 2381 (1950); Experientia 26: 473 (1970); J Agric Food Chem 24: 377 (1976)	0.56	1.51	0.01	0.68	0.69
	empty	100122-23	H12	sample								NA	NA	NA	NA	NA
	empty	100122-24	A01	sample								NA	NA	NA	NA	NA
00100552	beta-AMYRIN ACETATE	100122-24	A02	sample	1616-93-9	C32H52O2	468.77004		latof various species of rubber tree	undetermined activity	J Chem Soc 1952:2916; Phytocehmistry 9: 1669 (1970)	-0.62	-1.72	-1.99	-2.04	-1.59
00200015	ISOROTENONE	100122-24	A03	sample		C23H22O6	394.42819		semisynthetic	undetermined activity	Chem Rev 12, 181 (1933); J Chem Soc 1966:749	4.37	2.48	3.23	3.51	3.40
00100537	SOLASODINE	100122-24	A04	sample	126-17-0	C27H43NO2	413.64926	antineoplastic, antiinflammatory	numerous Solanum spp	experimental	J Am Chem Soc 76: 4245 (1954); Alkaloids 19: 81 (1981)	-1.08	-0.94	-0.75	-1.68	-1.11
00100541	DICTAMNINE	100122-24	A05	sample	484-29-7	C12H9NO2	199.21103		Dictamnus albus and other Rutaceae	undetermined activity	Chem & Ind 1956: 1091	0.38	1.21	0.77	0.51	0.72
00200013	ROTENONE	100122-24	A06	sample	83-79-4	C23H22O6	394.42819	acaricide, ectoparasiticide, antineoplastic, mitochondrial poison	Derris spp	agricultural use	Chem Rev 12: 181 (1933); Prog Chem Ord Nat Prod 43: 1 (1983)	5.27	6.03	5.29	5.34	5.48
00200007	GAMBOGIC ACID	100122-24	A07	sample	2752-65-0	C38H44O8	628.76958	antiinflammatory, cytotoxic, inhibits HeLa cells in vitro; LD50(rat) 88 mg/kg ip	Garcinia morella, G hanburyii	experimental	Tetrahedron 21: 1453 (1965); J Chem Soc(C) 1966:772; Tet Lett 1967:4201; Phytochemistry 41,: 815 (1996)	-6.21	-6.26	-0.28	2.20	-2.64
00200002	ORSELLINIC ACID, ETHYL ESTER	100122-24	A08	sample	2524-37-0	C10H12O4	196.20474		Roccella and Lecanora lichens	undetermined activity	J Org Chem 30: 3566 (1965)	-5.14	-4.51	-3.64	-0.58	-3.47
00201604	PYRROMYCIN	100122-24	A09	sample	668-17-7	C30H35NO11	585.61355	antibacterial	Streptomyces spp	experimental	Tet Letters 1959: 17; Chem Ber 92: 1904 (1959)	-0.53	-0.53	-0.85	1.97	0.01
00100540	OBLIQUIN	100122-24	A10	sample		C14H12O4	244.24934		sneezewood or umtati, Ptaeroxylon Obliquum	undetermined activity	J Chem Soc 1966: 114	0.75	-0.09	-0.11	-0.93	-0.10
00700024	HAEMATOPORPHYRIN	100122-24	A11	sample	14459-29-1	C34H38N4O6	598.70516	antidepressant, antineoplastic	Chlorella vulgaris; derived blood	experimental	J Biol Chem 202:801 (1953); Photochem Photobiol 39:851 (1984); 55:145 (1992)	0.48	5.53	3.92	1.93	2.97
	0.04% JAK2inhib	100122-24	A12	0.04% jak2inhib								-2.51	-1.23	-2.91	-2.75	-2.35
	0.04% DMSO	100122-24	B01	0.04% dmso								-0.57	-2.21	-2.15	3.03	-0.48
00240740	4-ACETOXYPHENOL	100122-24	B02	sample	3233-32-7	C8H8O3	152.15116	antioxidant	Ferulago aucheri, Salvia yosgadensis	experimental		-1.32	-1.82	-2.56	-2.11	-1.95
00300015	CHOLESTANE	100122-24	B03	sample	481-21-0	C27H48	372.68361		semisynthetic	undetermined activity		-0.15	-1.36	-0.86	-0.80	-0.79
00100688	DIGOXIGENIN	100122-24	B04	sample	1672-46-4	C23H34O5	390.52443		aglycon of digitoxin, thevetin, cerberin, echujin, evomonoside; mp 217-218	undetermined activity	J Chem Soc 1954: 2012	-0.47	-1.27	-0.13	-0.69	-0.64
00200774	IRIGENIN	100122-24	B05	sample	548-76-5	C18H16O8	360.32342		Iris spp	experimental	J Chem Soc 1928:1022; 1970:1219	-0.36	-0.05	-0.84	0.40	-0.21
01505490	APIGENIN DIMETHYL ETHER	100122-24	B06	sample	5728-44-9	C17H14O5	298.29813		common plant metabolite	undetermined activity		0.17	0.68	-0.72	-0.72	-0.15
00100254	5beta-12-METHOXY-4,4-BISNOR-8,11,13-PODOCARPATRIEN-3-ONE	100122-24	B07	sample		C16H20O2	244.3366		semisynthetic	undetermined activity		0.00	-0.84	-0.17	0.17	-0.21
00200424	2,3,4-TRIHYDROXY-4'-ETHOXYBENZOPHENONE	100122-24	B08	sample		C15H14O5	274.27583		semisynthetic	undetermined activity		-1.67	-1.58	-1.25	-0.94	-1.36
00310299	THYMOQUINONE	100122-24	B09	sample		C10H12O2	164.20594		Callitris, Monarda spp, Juniperus cedrus, Nigella sativa	undetermined activity	Planta Med 11: 465 (1963)	2.86	-0.48	0.04	0.09	0.63
00107013	SMILAGENIN	100122-24	B10	sample	126-18-1	C27H44O3	416.64993		Smilax spp.	undetermined activity	J Chem Soc 1936: 1399	-4.71	-0.74	-1.22	-0.27	-1.73
00200407	4'-HYDROXYCHALCONE	100122-24	B11	sample	2657-25-2	C15H12O2	224.26169		Shorea robusta aglycone	undetermined activity	JACS 52:2538 (1930)	1.49	0.78	-0.36	0.38	0.57
	0.04% JAK2inhib	100122-24	B12	0.04% jak2inhib								-0.44	-1.67	-1.40	-1.03	-1.13
	0.04% DMSO	100122-24	C01	0.04% dmso								0.10	-1.22	-1.70	-0.59	-0.85
00200034	ATRANORIN	100122-24	C02	sample	479-20-9	C19H18O8	374.35051		Common lichen metabolite	undetermined activity	Helv Chim Acta 9:650 (1926); 17:1319 (1934); J Chem Soc 1933:130; Chem Pharm Bull 24:2340 (1976); Gaia D1000	-1.44	-1.65	-0.41	-1.83	-1.33

												Screen Score (z-score)				
**Compound ID**	**MolNAME**	**plate**	**position**	**Content**	**cas#**	**Formula**	**MolWt**	**Bioactivity**	**Source**	**Status**	**Reference**	**rep1**	**rep2**	**rep3**	**rep4**	**Average**
00100205	3alpha-ACETOXYDIHYDRODEOXYGEDUNIN	100122-24	C03	sample		C30H40O7	512.6491		Meliaceae spp	undetermined activity		-0.79	-1.89	-0.39	-1.97	-1.26
00240927	CAPERATIC ACID	100122-24	C04	sample	29227-64-3	C21H38O7	402.53281	antibacterial (tuberculostatic)	lichens: Parmelia, Mycoblastus, Nephromopsis spp	undetermined activity	J Nat Prod 29:43 (1966); J Pharm Sci, 56:1611 (1967); 57:1804 (1968); Acta Chem Scand, 29:889 (1975)	-0.57	-1.60	-0.54	1.75	-0.24
00307033	3-HYDROXY-4-(SUCCIN-2-YL)-CARYOLANE delta-LACTONE	100122-24	C05	sample		C19H28O4	320.43261		derivative of caryophyllene	undetermined activity		-1.33	-1.10	-1.12	-0.70	-1.06
00100563	OXONITINE	100122-24	C06	sample		C33H43NO12	645.71016		derivative of aconitine	undetermined activity	J Am Chem Soc 76: 4048 (1954)	0.80	0.53	-0.25	-1.14	-0.02
00240942	ARTHONIOIC ACID	100122-24	C07	sample	25556-24-5	C29H36O9	528.60487		Arthonia impolita	undetermined activity	Z Naturforsch B 25: 49 (1970)	0.58	0.16	0.36	-0.76	0.09
00310002	ADONITOL	100122-24	C08	sample	488-81-3	C5H12O5	152.14839		Adonis spp	undetermined activity	Pharm Acta Helv 23: 153 (1948); JACS 104: 1109 (1982); J Chem Soc 1983: 1553	1.66	-0.39	-1.21	2.05	0.53
00240736	2-METHOXYXANTHONE	100122-24	C09	sample	1214-20-6	C14H10O3	226.234		Mammea and Keilmeyera spp.	undetermined activity	Phytochemistry 9: 447, 2537 (1970)	-0.33	0.31	-0.55	-0.51	-0.27
01500716	ALANYL-dl-LEUCINE	100122-24	C10	sample		C9H18N2O3	202.25541		synthetic	undetermined activity		-0.01	1.58	0.16	-0.30	0.36
01500865	HARMALOL HYDROCHLORIDE	100122-24	C11	sample	6028-07-5	C12H13ClN2O	236.70321	anthelmintic, narcotic agent	Peganium harmala	experimental	Biochem J 27: 727 (1933); Phytochemistry 19: 1573 (1980)	0.83	-0.18	-0.90	-0.28	-0.13
	0.11% JAK2inhib	100122-24	C12	0.11% jak2inhib								-2.67	-3.22	-3.25	-2.39	-2.88
	0.11% DMSO	100122-24	D01	0.11% dmso								-1.10	-2.00	-1.76	-0.90	-1.44
01500870	MYOSMINE	100122-24	D02	sample	532-12-7	C9H10N2	146.19345	mitogen	tobacco, nuts	undetermined activity	Tetrahedron Letters, 1967: 5185	0.00	-0.02	0.19	2.14	0.58
01500996	GLAFENINE	100122-24	D03	sample	3820-67-5	C19H17ClN2O4	372.81134	analgesic	synthetic; R-11707	INN, JAN		0.37	0.12	0.57	0.00	0.26
01501116	MEBHYDROLIN NAPHTHALENESULFONATE	100122-24	D04	sample	524-81-2	C29H28N2O6S2	564.68431	H1 antihistamine	synthetic	INN, BAN, JAN		-0.77	0.39	-0.08	-1.89	-0.59
01501113	PERUVOSIDE	100122-24	D05	sample	1182-67-2	C30H44O9	548.67978	cardiotonic	Thevetia peruviana	experimental	Helv Chim Acta 35:673, 730, 1073; 36:370 (1952); 45:907 (1962); 46:2886 (1963)	-2.69	-3.69	-2.83	2.57	-1.66
01500909	PROTOVERATRINE A	100122-24	D06	sample	143-57-7	C41H63NO14	793.95756	antihypertensive, emetic; LD50 (rat) 5 mg/kg po	Veratrum album, V viride, V nigrum	INN	J Biol Chem 149:271 (1943); JACS 82:2242, 2252 (1960); Planta Med 10:138 (1962)	1.13	0.63	1.04	-0.42	0.59
01501125	3-AMINOPROPANESULPHONIC ACID	100122-24	D07	sample	3687-18-1	C3H9NO3S	139.17408	antibacterial; GABA agonist	synthetic	experimental	Br J Pharmacol 59:373 (1977); Neuropharmacology 17:13 (1978)	-0.15	0.73	1.21	0.17	0.49
01501143	SULFAPHENAZOLE	100122-24	D08	sample	526-08-9	C15H14N4O2S	314.36843	antibacterial	synthetic	INN, BAN, JAN		0.81	0.11	0.62	-0.57	0.24
01501157	SUXIBUZONE	100122-24	D09	sample	27470-51-5	C24H26N2O6	438.48462	analgesic, antipyretic, antiinflammatory	synthetic	INN, BAN, JAN		0.91	0.11	0.82	0.71	0.64
01501172	AZOBENZENE	100122-24	D10	sample	103-33-3	C12H10N2	182.2269	acaricide	synthetic	experimental		3.99	-0.08	0.49	0.11	1.13
01501174	TODRALAZINE HYDROCHLORIDE	100122-24	D11	sample	14679-73-3	C11H13ClN4O2	268.70486	antihypertensive, peripheral vasodilator	synthetic; CEPH, BT-621	INN, BAN, JAN		0.56	0.17	0.12	2.79	0.91
	0.11% JAK2inhib	100122-24	D12	0.11% jak2inhib								-4.08	0.39	-3.16	-2.90	-2.44
	0.11% JAK2inhib	100122-24	E01	0.11% jak2inhib								-3.96	-2.55	-3.89	-3.47	-3.47
01501183	ESTRADIOL METHYL ETHER	100122-24	E02	sample	1035-77-4	C19H26O2	286.41787	estrogen	semisynthetic	experimental	JACS 100: 6218 (1978)	-2.09	-1.40	0.86	1.21	-0.36
01501198	TOLFENAMIC ACID	100122-24	E03	sample	13710-19-5	C14H12ClNO2	261.71024	antiinflammatory, analgesia	synthetic	INN, BAN, JAN		0.40	0.78	1.92	0.48	0.90
01501192	ESTRADIOL-3-SULFATE, SODIUM SALT	100122-24	E04	sample		C18H23NaO5S	374.43481	estrogen	semisynthetic	experimental		-0.41	-0.92	-0.59	4.46	0.63
01502070	6,7-DICHLORO-3-HYDROXY-2-QUINOXALINECARBOXYLIC ACID	100122-24	E05	sample		C9H4Cl2N2O3	259.04983	NMDA and kainate receptor antagonist	synthetic	experimental	Neurosci Lett 91: 194 (1988)	0.20	1.10	-0.25	4.15	1.30
01502074	4-NAPHTHALIMIDOBUTYRIC ACID	100122-24	E06	sample		C16H13NO4	283.28631	aldose reductase inhibitor	synthetic	experimental		0.60	0.83	1.26	1.22	0.98
01502073	IMIDAZOL-4-YLACETIC ACID SODIUM SALT	100122-24	E07	sample	56368-58-2	C5H5N2NaO2	148.0976	GABAc antagonist	synthetic	experimental	Brit J Pharmacol 109: 200 (1993); Gen Pharmacol 31:503 (1998)	2.77	0.02	0.82	0.17	0.95
01502083	N- (9-FLUORENYLMETHOXYCARBONYL)-L-LEUCINE	100122-24	E08	sample	35661-60-0	C21H23NO4	353.42176	antiinflammatory	synthetic; NPC-15199	experimental	PNAS 88:355 (1991); J Pharmacol Exp Ther 266:468 (1993)	0.58	0.26	-0.27	0.11	0.17
01502095	alpha-CYANO-3-HYDROXYCINNAMIC ACID	100122-24	E09	sample		C10H7NO3	189.17219	inhibit mitochondrial pyruvate transport	synthetic	experimental		-0.02	-0.09	0.32	0.52	0.18
01502092	5-FLUOROINDOLE-2-CARBOXYLIC ACID	100122-24	E10	sample	399-76-8	C9H6FNO2	179.15207	NMDA receptor antagonist (gly)	synthetic	experimental		-0.64	0.46	-0.60	-0.07	-0.21
01502114	p-FLUOROPHENYLALANINE	100122-24	E11	sample	51-65-0	C9H10FNO2	183.18395	amino acid antagonist, protein synthesis inhibitor	synthetic	experimental	J Biol Chem 269: 24637 ((1994); Biochemistry 35: 6969 (1996); Eur J Cell Biol 23: 312 (1981)	0.20	0.21	0.31	1.43	0.54
	0.11% DMSO	100122-24	E12	0.11% dmso								-0.43	0.51	-0.96	2.48	0.40
	0.11% JAK2inhib	100122-24	F01	0.11% jak2inhib								-3.64	-4.73	-2.88	-1.60	-3.21
01502103	ANTHRAQUINONE	100122-24	F02	sample	84-65-1	C14H8O2	208.21866	irritant	synthetic	experimental		-0.35	-0.82	-1.20	-0.52	-0.72
01502130	AMINOCYCLOPROPANECARBOXYLIC ACID	100122-24	F03	sample	68781-13-5	C4H7NO2	101.10589	NMDA partial agonist (gly)	synthetic; ACPC	experimental		0.76	-0.36	-0.18	-0.48	-0.07
01502128	CYCLOLEUCINE	100122-24	F04	sample	52-52-8	C6H11NO2	129.16007	NMDA receptor antagonist (gly)	synthetic	experimental		1.15	2.20	0.19	-0.46	0.77
01502129	ISOGUVACINE HYDROCHLORIDE	100122-24	F05	sample	64603-90-3	C6H10ClNO2	163.6051	GABA agonist	synthetic	experimental		-0.18	0.42	0.37	-2.09	-0.37
01502162	p-CHLOROPHENYLALANINE	100122-24	F06	sample	7424-00-2(dl); 14173-39-8(l)	C9H10ClNO2	199.63855	Irreversible inhibitor of tryptophan hydroxylase	synthetic	experimental		0.51	-0.52	0.78	-0.55	0.06
01502229	ABIETIC ACID	100122-24	F07	sample	514-10-3	C20H30O2	302.4609		common diterpene acid in conifers	undetermined activity		0.31	0.36	1.12	0.69	0.62
01502209	PHENETHYL CAFFEATE (CAPE)	100122-24	F08	sample	104594-70-9	C17H16O4	284.31467	antineoplastic, antiinflammatory, immunomodulator, NFkB blocker	synthetic	experimental	PNAS 93:9090 (1996)	0.42	-0.12	2.07	0.93	0.82
01502207	L-LEUCYL-L-ALANINE	100122-24	F09	sample	7298-84-2	C9H18N2O3	202.25541	ubiquitin blocker, neurite growth inhibitor	synthetic	experimental	Biochim Biophys Res Commun 189:280 (1992); Experientia 52:865 (1996)	-0.16	0.37	0.29	1.22	0.43
01503254	6alpha-METHYLPREDNISOLONE ACETATE	100122-24	F10	sample		C24H32O6	416.51904	glucocorticoid	semisynthetic	experimental		1.00	0.48	-0.23	1.68	0.73
01503127	DEQUALINIUM CHLORIDE	100122-24	F11	sample	522-51-0, 6707-58-0 [dequalinium]	C30H40Cl2N4	527.5861	antiinfectant	synthetic; BAQD-10	INN, BAN, JAN		0.76	0.42	2.46	-0.09	0.89
	0.04% DMSO	100122-24	F12	0.04% dmso								-0.39	0.42	-0.86	-0.41	-0.31
	0.04% JAK2inhib	100122-24	G01	0.04% jak2inhib								-2.30	-2.64	-1.36	-2.23	-2.13
01503077	PRIDINOL METHANESULFONATE	100122-24	G02	sample	511-45-5	C21H29NO4S	391.53358	anticholinergic	synthetic; C-238	INN, JAN		-1.21	-1.05	-2.05	-1.53	-1.46
01503043	BUCLADESINE	100122-24	G03	sample	362-74-3	C18H24N5O8P	469.39448	vasodilator	synthetic	INN, JAN		-1.27	0.44	-0.26	-0.55	-0.41
01502259	MORIN	100122-24	G04	sample	480-16-0	C15H10O7	302.24275	P450 and ATPase inhibitor	Chlorophora tinctoria	experimental	J Chem Soc 67: 649 (1895)	-0.59	0.09	0.52	0.00	0.00
01503391	D-PHENYLALANINE	100122-24	G05	sample	673-06-3	C9H11NO2	165.19352	antidepressant	synthetic	INN		0.38	0.26	-0.04	-0.42	0.05
01503403	EXALAMIDE	100122-24	G06	sample	53370-90-4	C13H19NO2	221.30188	antifungal	synthetic	INN, JAN	J Pharm Pharmacol 9: 855 (1957)	0.73	0.42	0.92	-0.25	0.45
01503297	HEXAMETHONIUM BROMIDE	100122-24	G07	sample	55-97-0, 60-26-4 [hexamethonium]	C12H30Br2N2	362.1943	antihypertensive, ganglionic blocker	synthetic	INN, BAN, JAN		-0.69	-0.46	0.76	0.23	-0.04
01503425	ZOPICLONE	100122-24	G08	sample	43200-80-2	C17H17ClN6O3	388.81644	hypnotic, sedative	synthetic	INN, BAN, JAN		0.00	-0.06	1.06	0.42	0.35
01503637	METITEPINE MALEATE	100122-24	G09	sample	20229-30-5	C24H28N2O4S2	472.62976	5HT1&2 receptor antagonist	synthetic	INN		-0.18	1.44	0.39	0.23	0.47
01503641	1-PHENYLBIGUANIDE HYDROCHLORIDE	100122-24	G10	sample	55-57-2	C8H12ClN5	213.67134	5HT3 receptor agonist	synthetic	experimental		2.04	-0.08	-0.27	4.73	1.60
01503672	SODIUM THIOGLYCOLATE	100122-24	G11	sample	367-51-1	C2H3NaO2S	114.09881	dipilatory, irritant	synthetic	experimental		-0.20	0.41	0.04	-0.37	-0.03
	0.04% DMSO	100122-24	G12	0.04% dmso								0.04	-0.05	-0.08	-0.56	-0.16
	0.04% JAK2inhib	100122-24	H01	0.04% jak2inhib								-2.22	-3.05	-0.08	-3.76	-2.28
01503636	N-METHYL-D-ASPARTIC ACID (NMDA)	100122-24	H02	sample	6384-92-5	C5H9NO4	147.13178	NMDA agonist	synthetic	experimental		-0.19	0.84	0.99	-1.28	0.09
01503051	RETINYL ACETATE	100122-24	H03	sample	127-47-9	C22H32O2	328.49914	vitamin precursor	semisynthetic	JAN		0.33	0.55	-0.22	3.04	0.92
01503936	PERICIAZINE	100122-24	H04	sample	2622-26-6	C21H23N3OS	365.50096	antipsychotic	synthetic	INN, BAN, JAN		-0.60	-0.65	-0.09	-0.79	-0.53
01503906	ANISOMYCIN	100122-24	H05	sample	22862-76-6	C14H19NO4	265.31183	antiprotozoal, antifungal, protein synthesis inhibitor	Streptomyces griseolus	experimental	JACS 76:4053 (1954); Chem Pharm Bull 17:1405 (1969); Prog Neurobiology 16:155 (1981); Mol Cell Biochem 14:7352 (1994)	0.79	2.19	2.55	1.29	1.71
01503947	CACODYLIC ACID	100122-24	H06	sample	75-60-5	C2H7AsO2	137.99849	antieczema, dermatologic, herbicide	synthetic	experimental		-1.26	-1.40	0.08	-0.62	-0.80
01503941	THIOCTIC ACID	100122-24	H07	sample	1077-24-7	C8H14O2S2	206.32758	hepatoprotectant	synthetic; alpha-lipoic acid	JAN	JACS 74:1868, 3455 (1992); Angewante Chemie Int Ed 4:846 (1965); Arzneim Forsch 20:1210 (1970)	-0.41	-0.13	2.91	0.73	0.78
01503928	5-METHYLFURMETHIDE	100122-24	H08	sample	1197-60-0	C9H16INO	281.13837	muscarinic agonist	synthetic	experimental	Eur J Pharmacol 186:257 (1990); 244:21 (1993)	-0.55	-0.75	3.75	-0.27	0.54
01504009	LEVULINIC ACID, 3-BENZYLIDENYL-	100122-24	H09	sample		C12H12O3	204.22764		derivative	undetermined activity		0.83	0.09	-0.12	0.05	0.21
01503970	METHOXYAMINE HYDROCHLORIDE	100122-24	H10	sample	593-56-6	CH6ClNO	83.51807	hydroxymethyltransferase inhibitor	synthetic	experimental		0.88	-0.71	0.59	-1.32	-0.14
01503954	TULOBUTEROL	100122-24	H11	sample	41570-61-0	C12H19Cl2NO	264.19733	bronchodilator, beta adrenergic agonist	synthetic	INN, BAN, JAN		0.94	-0.21	-1.25	-1.92	-0.61
	empty	100122-24	H12	sample								NA	NA	NA	NA	NA
	empty	100122-25	A01	sample								NA	NA	NA	NA	NA
01504135	TRIMEDLURE	100122-25	A02	sample	12002-53-8	C12H21ClO2	232.75297	arthropod pheromone	synthetic; 5-Cl isomer present	agricultural use	J Econ Entomol 59:1450 (1966)	NA	1.94	-1.63	-0.11	0.07
01504143	ARTENIMOL	100122-25	A03	sample	81496-81-3	C15H24O5	284.35553	antimalarial, antiinflammatory	semisynthetic; dihydroartemisinin	INN	Med Res Rev 7: 29 (1987); Am J Trop Med Hyg 61:187 (199); Trends Pharmacol Sci 20:199 (1999)	NA	-1.44	-2.41	-2.53	-2.13
01504145	GLICLAZIDE	100122-25	A04	sample	21187-98-4	C15H21N3O3S	323.41692	antidiabetic, adhesion inhibitor	synthetic; SE-1702	INN, BAN, JAN	Drugs 27:301 (1984); J Diabetes Complications 14:201 (2000); Metabolism 50: 688 (2001)	NA	-1.00	-0.75	-0.09	-0.61
01504137	ANCITABINE HYDROCHLORIDE	100122-25	A05	sample	10212-25-6	C9H12ClN3O4	261.66669	antineoplastic	synthetic	INN, JAN	Carcinogenesis 7:1167 (1992)	NA	-1.12	-0.10	-0.67	-0.63
01500840	CHOLIC ACID	100122-25	A06	sample	81-25-4	C24H40O5	408.5834		mammalian bile	undetermined activity	Biochem J 85: 236 (1962)	NA	1.33	-0.23	-0.17	0.31
01504182	3-METHYLXANTHINE	100122-25	A07	sample	1076-22-8	C6H6N4O2	166.14032	phosphodiesterase inhibitor	synthetic	experimental	Clin Exp Pharmacol Physiol 18: 163 (1991)	NA	-1.10	-0.44	-0.83	-0.79
01504151	NIMUSTINE	100122-25	A08	sample	42471-28-3	C9H13ClN6O2	272.69596	antineoplastic	synthetic	INN, JAN	Carcinogenesis 21: 1879 (2000)	NA	-0.11	1.00	-0.65	0.08
01504153	NORCANTHARIDIN	100122-25	A09	sample		C8H8O4	168.15056	antineoplastic, protein phosphatase inhibitor	synthetic	experimental	Biochem Biophys Res Comm 276: 278 (2000)	NA	2.01	-1.10	0.09	0.34
01504209	DIPLOSALSALATE	100122-25	A10	sample	530-75-6	C16H12O6	300.27044	analgesic, antipyretic	synthetic	experimental		NA	0.45	0.56	-0.88	0.04
01504221	PHENYLBUTYRATE SODIUM	100122-25	A11	sample	90-27-7 (phenylbutyric acid)	C10H11NaO2	186.18777	antihyperlipidemic	synthetic; R-757, Th-22	experimental		NA	0.43	1.18	-1.20	0.13
	0.04% JAK2inhib	100122-25	A12	0.04% jak2inhib								NA	-0.51	-1.49	-4.34	-2.11
	0.04% DMSO	100122-25	B01	0.04% dmso								NA	0.45	1.35	-1.21	0.19
01504225	2-MERCAPTOBENZOTHIAZOLE	100122-25	B02	sample	149-30-4	C7H5NS2	167.2526	antifungal	synthetic; AG-63	experimental		NA	-1.71	-0.90	0.48	-0.71
01504228	CRUSTECDYSONE	100122-25	B03	sample	5289-74-7	C27H44O7	480.64753	insect molting hormone	silkworm moth Bombyx mori & the plant Achyranthes fauriei	experimental	Yakugaku Zasshi 87: 325 (1967); Arch Insect Biochem Phsiol 33: 231 (1996); J Neurosci 18: 8886 (1998)	NA	0.23	-1.28	0.03	-0.34
01504215	N,N-HEXAMETHYLENEAMILORIDE	100122-25	B04	sample		C12H18ClN7O	311.77656	Na/H+ antiporter inhibitor, diuretic	synthetic	experimental	Biochim Biophys Acta 944: 383 (1988)	NA	0.16	-1.47	1.66	0.11
01504223	FENBUTYRAMIDE	100122-25	B05	sample	90-26-6	C10H13NO	163.22121	antihyperlipidemic	synthetic	experimental		NA	-0.75	-1.03	-0.62	-0.80
01505007	2,3-DIMERCAPTOSUCCINIC ACID	100122-25	B06	sample	304-55-2	C4H6O4S2	182.21802	chelating agent, antihypertensive	synthetic; DMSA	experimental	Biochem Pharmacol 65:1741 (2003)	NA	-0.41	-1.16	-1.03	-0.87
01505001	PUERARIN	100122-25	B07	sample	3681-99-0	C21H20O9	416.38815	beta-adrenergic blocker	Pueraria spp	experimental	Chem Pharm Bull 8:688 (1960)	NA	-0.30	-1.10	-0.85	-0.75
01504244	BETAMIPRON	100122-25	B08	sample	3440-28-6	C10H11NO3	193.20407	sweetener	synthetic	INN, JAN		NA	0.19	0.13	0.15	0.16
01504232	2,3-DIHYDROXY-6,7-DICHLOROQUINOXALINE	100122-25	B09	sample		C8H4Cl2N2O2	231.03928	NMDA receptor antagonist (gly)	synthetic	experimental	J Neurochem 54: 699 (1990)	NA	-0.14	0.35	2.47	0.89
01505015	3H-1,2-DITHIOLE-3-THIONE	100122-25	B10	sample		C3H2S3	134.24139	antioxidant, antineoplastic	synthetic; D3T	experimental	Free Rad Biomed 28:944 (2000)	NA	-0.01	0.31	-1.84	-0.51
01505011	TRIMEBUTINE MALEATE	100122-25	B11	sample	34140-59-5(base)	C26H33NO9	503.55421	antispasmodic, opiod receptor agonist	synthetic	INN, BAN, JAN	Japan J Pharmacol 33:301 (1983); Hepato-Gastroenterology 49:193 (2002)	NA	0.15	2.85	-2.29	0.23
	0.04% JAK2inhib	100122-25	B12	0.04% jak2inhib								NA	0.25	3.44	-2.38	0.44
	0.04% DMSO	100122-25	C01	0.04% dmso								NA	-1.21	-1.76	-0.19	-1.05
01505036	TANSHINONE IIA SULFONATE SODIUM	100122-25	C02	sample		C19H17NaO6S	396.39754	free radical scavenger	semisynthetic	experimental	Biochem Pharmacol 46:2327 (1993); 65:51 (2003); Biochem Mol Biol Int 38:1171 (1996)	NA	-0.91	-1.18	0.25	-0.62
01505039	2,5-DI-t-BUTYL-4-HYDROXYANISOLE	100122-25	C03	sample	1991-52-2	C15H24O2	236.35733	antioxidant	synthetic	experimental		NA	-0.34	-1.20	1.95	0.14
01505107	VALINOMYCIN	100122-25	C04	sample	2001-95-8	C54H90N6O18	1111.3488	antibiotic; LD50 (rat, po) 4 mg/kg	Streptomyces spp	experimental	Chem Ber 88:57 (1955); Biochem Biophys Res Commun 15:1964 (1964); JACS 99:2032 (1977)	NA	-0.43	1.76	0.08	0.47
01505041	BISPHENOL A	100122-25	C05	sample	80-05-7	C15H16O2	228.29357	endocrine disruptor, plastic monomer	synthetic	experimental		NA	-0.70	-0.93	-0.89	-0.84
01505104	4-(3-BUTOXY-4-METHOXYBENZYL)IMIDAZOLIDIN-2-ONE	100122-25	C06	sample	29925-17-5	C15H22N2O3	278.35419	cAMP PDE inhibitor, inhibits cellular adhesion and superoxide & platelet aggregation	synthetic; RO-20-1724	experimental	Mol Pharmacol 13: 38 (1977); Biochem Biophs Acta 763: 276 (1983); Life Sci 63: 265 (1998)	NA	2.61	0.49	0.36	1.15
01505109	MANGANESE TETRAKIS(4-CARBOXYPHENYL)PORPHYRIN CHLORDE	100122-25	C07	sample		C48H28ClMnN4O8	879.17136	SOD mimetic	synthetic	experimental	J Biol Chem 269:23471 (1994); Br J Pharmacol 120:259 (1997); Toxicol Appl Pharmacol 26:25 (1998)	NA	0.12	-0.45	-0.08	-0.14
01505083	PRASTERONE	100122-25	C08	sample	53-43-0	C19H28O2	288.43381	androgen, estrogen, treatment of menopausal syndrome	adrenal glands, male urine	INN	Lancet 343:1479 (1994)	NA	0.43	1.01	0.36	0.60
01505158	2,3-DICHLORO-5,8-DIHYDROXYNAPTHOQUINONE	100122-25	C09	sample	14918-65-5	C10H4Cl2O4	259.04698	apoptosis inducer, antiproliferative	synthetic	experimental	Bioorg Med Chem Lett 8:2507 (1998); J Biol Chem 276:24638 (2001)	NA	0.22	0.95	0.13	0.43
01505157	2,2'-AZO-bis-2-AMINOPROPANE	100122-25	C10	sample	2997-92-4	C8H20Cl2N6	271.1948	free radical initiator, apoptosis inducer	synthetic	undetermined activity	Biochemistry 36:7706 (1997); 40:1719 (2001); Biochem Biophys Acta 1543:69 (2000)	NA	1.22	0.90	-1.17	0.31

														Screen Score (z-score)		
Compound ID	MolNAME	plate	position	Content	cas#	Formula	MolWt	Bioactivity	Source	Status	Reference	rep1	rep2	rep3	rep4	Average
01505156	N-PHENYLANTHRANILIC ACID	100122-25	C11	sample	91-40-7	C13H11NO2	213.23812	ion channel (Cl) blocker	synthetic	experimental	Am J Physiol 253:C79 (1987); J Biol Chem 276:11575 (2001)	NA	1.00	0.74	-0.21	0.51
	0.11% JAK2inhib	100122-25	C12	0.11% jak2inhib								NA	-2.07	0.04	-3.71	-1.91
	0.11% DMSO	100122-25	D01	0.11% dmso								NA	-1.86	-1.71	-0.43	-1.34
01505154	3-AMINO-1,2,4-TRIAZOLE	100122-25	D02	sample	61-82-5	C2H4N4	84.08098	catalase inhibitor	synthetic	experimental	Biochem Pharmacol 42:699 (1991); Am J Physiol 270:H1165	NA	-0.92	-0.89	-0.21	-0.67
01505250	MADECASSIC ACID	100122-25	D03	sample	18449-41-7	C30H48O6	504.71346	wound healing	Centella asiatica	experimental	Bull Soc Chim Fr 1967: 1890; Phytochemistry 7: 1385 (1968); 28: 2852 (1989)	NA	-1.28	-1.39	2.24	-0.14
01505163	AURIN TRICARBOXYLIC ACID	100122-25	D04	sample	4431-00-9	C22H14O9	422.35148	apoptosis inhibitor, topoisomerase II inhibitor	synthetic	experimental	Eur J Biochem 248:30 (1997)	NA	-0.42	-0.88	-0.06	-0.45
01505167	ACADESINE	100122-25	D05	sample	2627-69-2	C9H14N4O5	258.23573	glucose uptake stimulant; AMPK activator	synthetic	INN	Eur J Biochem 229:558 (1995)Biochem J 338:783 (1999); J Mol Neurosci 17:45 (2001)	NA	-0.07	0.18	0.04	0.05
01505165	2-METHYL-4-(PIPERIDIN-1-YLCARBOXY)-5-ISOPROPYLPHENYLTRIMETHYLAMMONIUM CHLORIDE	100122-25	D06	sample		C19H31ClN2O2	354.92412	squalene-2,3-oxide cyclase inhibitor, anticholesterolemic	synthetic	experimental	Steroids 58:74 (1993); J Biol Chem 250:1571 (1975)	NA	-0.92	0.61	-0.20	-0.17
01505164	4,4'-DIISOTHIOCYANOSTILBENE-2,2'-SUFONIC ACID SODIUM SALT	100122-25	D07	sample	67483-13-0	C16H8N2Na2O6S4	498.48756	ATP transport inhibitor, anion transport inhibitor, antiulcer	synthetic; DIDS	undetermined activity	EMBO J 12:69 (1993); Biochem Biophys Res Commun 207:375 (1995)	NA	-0.29	-0.07	-0.12	-0.16
01505298	3-ISOBUTYL-1-METHYLXANTHINE (IBMX)	100122-25	D08	sample	28822-58-4	C10H14N4O2	222.24868	phosphodiesterase inhibitor	synthetic	experimental		NA	0.23	0.75	0.72	0.57
01505324	BISSALICYL FUMARATE	100122-25	D09	sample		C18H12O8	356.29154	crosslinking agent (hemoglobin)	synthetic	experimental	J Biol Chem 255:2816 (1980)	NA	0.72	4.72	-1.46	1.33
01505316	PROTIONAMIDE	100122-25	D10	sample	14222-60-7	C9H12N2S	180.27339	antibacterial	synthetic; TH-1321, RP-9778	INN, BAN, JAN	Chemotherapy 26:276 (1980)	NA	-0.27	3.87	-0.17	1.14
01505317	CARMOFUR	100122-25	D11	sample	61422-45-5	C11H16FN3O3	257.26687	antineoplastic	synthetic	INN, JAN	Am J Clin Oncology 13:477 (1990)	NA	0.13	1.97	0.43	0.84
	0.11% JAK2inhib	100122-25	D12	0.11% jak2inhib								NA	-2.77	-0.86	-2.49	-2.04
	0.11% JAK2inhib	100122-25	E01	0.11% jak2inhib								NA	-4.02	-2.61	0.84	-1.93
01505328	4'-DEMETHYLEPIPODOPHYLLOTOXIN	100122-25	E02	sample		C21H20O8	400.38875	antineoplastic	semisynthetic	experimental	Eur J Cancer Clin Oncol 24:1415 (1988)	NA	-6.87	-2.24	-1.62	-3.58
01505339	L-PHENYLALANINOL	100122-25	E03	sample	3182-95-4	C9H13NO	151.21006	antiulcer	synthetic	experimental	Chem Pharm Bull 40:3113 (1992)	NA	-1.39	-0.42	1.22	-0.20
01505336	VALERYL SALYCILATE	100122-25	E04	sample	64206-54-8	C12H14O4	222.24298	COX-1 inhibitor	synthetic	experimental	Arch Biochem Biophys 317:19 (1995); OphthalmolVis Sci 39:1888 (1998)	NA	0.11	-0.12	0.02	0.00
01505331	3,3'-DIINDOLYLMETHANE	100122-25	E05	sample	1968-05-4	C17H14N2	246.31453	apoptosis inducer	synthetic	experimental	Biochem Biophys Res Commun 228:153 (1996)	NA	1.20	-0.96	0.37	0.20
01505337	ELAIDYLPHOSPHOCHOLINE	100122-25	E06	sample		C23H48NO4P	433.61711	antineoplastic	synthetic	experimental		NA	0.76	0.85	0.42	0.68
01505342	7-NITROINDAZOLE	100122-25	E07	sample	2942-42-9	C7H5N3O2	163.1368	NO synthetase inhibitor	synthetic	experimental	Eur J Pharmacol 310:115 (1996); TIPS 18:204 (1997)	NA	0.82	0.09	0.45	0.45
01505253	SINOMENINE	100122-25	E08	sample	115-53-7	C19H23NO4	329.39946	weak abortifacient, immunosuppressant, analgesic, antiinflammatory; LD50 (po) 580 mg/kg; (ip) 285 mg/kg(mouse)	Sinomenium acutum and Stephania cepharantha	experimental	Annalen 485: 247 (1931); J Chem Soc 1955: 3252; J Immunopharmacol 7: 33 (1985); Arzneim-Forsch 44: 1223 (1994)	NA	0.29	0.61	-0.71	0.06
01505252	PALMATINE	100122-25	E09	sample	3486-67-7	C21H24NO5	370.42913	uterine contractant, antibacterial, antiarrhythmic, inotropic, adrenocorticotropic, anticholinesterase, analgesic	Jateorhesa palmata, Berberis spp	experimental	Ber 58: 2267 (1925); J Nat Prod 28: 73 (1965)	NA	2.60	0.50	0.41	1.17
01505395	BICUCULLINE(-) METHIODIDE	100122-25	E10	sample	55950-07-7	C21H20INO6	509.30105	GABAa antagonist	semisynthetic; water soluble derivative of bicuculline (01500821)	experimental	Brain Res 465:243 (1989); TiPS 20:268 (1999)	NA	0.56	-0.41	-0.30	-0.05
01505930	SR-2640	100122-25	E11	sample	105350-26-3	C23H18N2O3	370.41151	LTD4/ LTE4 antagonist	synthetic; 2[3-(quinolin-2-ylmethoxy)phenylamino]benzoic acid	experimental	Am Rev Respir Dis 137(suppl):427 (1988); Eur J Pharmacol 155:117 (1988); Biochem Pharmacol 38:2291 (1989); Agents Actions 29:299 (1990)	NA	0.52	1.07	-0.02	0.52
	0.11% DMSO	100122-25	E12	0.11% dmso								NA	-0.26	3.29	-0.73	0.77
	0.11% JAK2inhib	100122-25	F01	0.11% jak2inhib								NA	-4.29	-4.42	-1.94	-3.55
01505915	PROXYPHYLLINE	100122-25	F02	sample	6003-00-9	C10H14N4O3	238.24808	PDE inhibitor, bronchodilator, vasodilator	synthetic	INN, BAN, JAN		NA	-1.42	-1.22	0.29	-0.78
01505785	CAMYLOFINE DIHYDROCHLORIDE	100122-25	F03	sample	54-30-8	C19H34Cl2N2O2	393.40103	anticholinergic	synthetic	INN, JAN		NA	-2.00	-1.07	-0.17	-1.08
01505849	ETHIONINE	100122-25	F04	sample	67-21-0	C6H13NO2S	163.24001	antineoplastic; inhibitor DNA methylation	synthetic	experimental	J Org Chem 16:749 (1971); Adv Can Res 7:383 (1963)	NA	-0.60	0.22	1.75	0.46
01505846	11alpha-HYDROXYPROGESTERONE HEMISUCCINATE	100122-25	F05	sample	41238-98-6	C25H34O6	430.54613	glucocorticoid	semisynthetic	experimental		NA	0.15	1.71	-0.01	0.62
01505880	DIFLORASONE DIACETATE	100122-25	F06	sample	33564-31-7	C26H32F2O7	494.53754	antiinflammatory, glucocorticoid	semisynthetic; U-34865	USP, INN, BAN, JAN		NA	-0.87	-1.11	-0.79	-0.92
01505986	NIALAMIDE	100122-25	F07	sample	51-12-7	C16H18N4O2	298.34746	MAO inhibitor	synthetic	INN, BAN, NF-VIII	Toxicol Appl Pharmacol 1:524 (1959); hepatotoxic at clinical dose (100mg)	NA	0.89	1.78	-0.60	0.69
01505869	AMPYRONE	100122-25	F08	sample	83-07-8	C11H13N3O	203.24576	analgesic, antiinflammatory, antipyretic	synthetic	experimental		NA	1.04	0.05	-0.71	0.13
01505742	ASCORBYL PALMITATE	100122-25	F09	sample	137-66-6	C22H38O7	414.54396	antioxidant	semisynthetic	USAN, NF		NA	-1.41	-1.11	2.27	-0.09
01505773	FAMPROFAZONE	100122-25	F10	sample	22881-35-2	C24H31N3O	377.53417	analgesic, antipyretic, CNS stimulant	synthetic	INN, BAN	J Forensic Sci 52:479 (2007)	NA	-0.44	1.19	-0.41	0.11
01505774	OXELAIDIN CITRATE	100122-25	F11	sample	52432-72-1, 468-61-1 (base)	C26H41NO10	527.61737	antitussive	synthetic	INN, BAN	J Pharm Pharmacol 10:40 (1958)	NA	0.01	1.71	-0.38	0.45
	0.04% DMSO	100122-25	F12	0.04% dmso								NA	0.49	1.07	0.10	0.56
	0.04% JAK2inhib	100122-25	G01	0.04% jak2inhib								NA	-3.66	-3.14	-1.64	-2.81
01506010	METHIONINE SULFOXIMINE (L)	100122-25	G02	sample	15985-39-4	C5H12N2O3S	180.22699	glutamine synthetase inhibitor, ornithine decarboxylase enhancer	synthetic	experimental		NA	-1.79	-0.12	0.73	-0.39
01506041	SALSOLINOL HYDROBROMIDE	100122-25	G03	sample	38221-21-5	C10H14BrNO2	260.13258	prolactin-releasing factor	dopamine derivative	experimental	Neurosignals 12:315 (2003); Physiol Res 55:353 (2006)	NA	-0.44	-0.34	-0.26	-0.35
01506051	TRIMETHYLCOLCHICINIC ACID	100122-25	G04	sample	3482-37-9	C19H21NO5	343.38292	antineoplastic	derivative	experimental	JACS 75:5292 (1953)	NA	-0.12	-0.05	0.61	0.14
01506044	THIOGUANOSINE	100122-25	G05	sample	85-31-4	C10H13N5O4S	299.31021	antineoplastic	synthetic	experimental	Can Res 21:1015 (1961)	NA	-0.36	-0.12	4.27	1.26
01506078	MOROXYDINE HYDROCHLORIDE	100122-25	G06	sample	3731-59-7	C6H14ClN5O	207.66438	antiviral	synthetic; SKF-8898A, ABOB	INN, BAN	Acta Derm Venereol Suppl (Stockh) 183:1 [1994]	NA	-0.46	0.18	1.13	0.28
01506088	TRIMETAZIDINE DIHYDROCHLORIDE	100122-25	G07	sample	5011-34-7	C14H24Cl2N2O3	339.26498	anti-anginal	synthetic; 400045	INN, BAN, JAN	Circ Res 86:580 (2000); Am J Cardiovasc Drugs 3:361 (2003); Circulation 118:1250 (2008)	NA	1.19	-0.48	0.86	0.52
01601020	DIETHYLTOLUAMIDE	100122-25	G08	sample	134-62-3	C12H17NO	191.27539	insect repellant	synthetic; DEET	USP, INN, BAN		NA	1.41	-0.52	-0.06	0.28
01400666	METHOXYVONE	100122-25	G09	sample		C17H14O3	266.29933	anabolic	synthetic	experimental		NA	0.65	0.37	2.48	1.17
01500629	FLOPROPIONE	100122-25	G10	sample	2295-58-1	C9H10O4	182.17765	antispasmodic	synthetic	INN, JAN		NA	0.46	1.04	0.57	0.69
01500666	ACEMETACIN	100122-25	G11	sample	53164-05-9	C21H18ClNO6	415.83371	antiinflammatory	synthetic	INN, BAN, JAN		NA	0.19	1.21	-0.15	0.42
	0.04% DMSO	100122-25	G12	0.04% dmso								NA	0.24	2.75	-0.70	0.76
	0.04% JAK2inhib	100122-25	H01	0.04% jak2inhib								NA	-2.82	-1.74	-2.01	-2.19
01500671	DIHYDROFOLIC ACID	100122-25	H02	sample	4033-27-6	C19H21N7O6	443.42252	antidote to methotrexate toxicity	dihydrofolate reductase product	experimental	Nature 188:231 (1960); Compr Biochem 21:111 (1971); J Chem Soc(Perkin 1) 1985:1349; Tetrahedron 42:117 (1986)	NA	-1.00	-2.85	0.50	-1.12
01500703	ACETYLGLUTAMIC ACID	100122-25	H03	sample		C7H11NO5	189.16942	excitatory aminoacid	synthetic	experimental		NA	-1.36	-0.77	0.01	-0.70
01500998	ETHOXYQUIN	100122-25	H04	sample		C14H19NO	217.31363	antioxidant	synthetic	experimental		NA	-2.15	-0.98	0.50	-0.88
01500705	O-BENZYL-l-SERINE	100122-25	H05	sample		C10H13NO3	195.22001		synthetic	undetermined activity		NA	-0.95	0.80	1.04	0.29
01500715	ACETYLGLUCOSAMINE	100122-25	H06	sample		C8H15NO6	221.21185	antiarthritic	synthetic	experimental		NA	0.34	-0.50	0.70	0.18
01500710	ACETYLPHENYLALANINE	100122-25	H07	sample		C11H13NO3	207.23116		synthetic	undetermined activity		NA	0.72	0.06	-0.02	0.25
01500728	5,7-DIHYDROXY-4-METHYLCOUMARIN	100122-25	H08	sample		C10H8O4	192.17286		semisynthetic	undetermined activity		NA	0.81	0.94	2.64	1.46
01500802	ALLOXAN	100122-25	H09	sample	2244-11-3	C4H2N2O4	142.07154	specific cytotoxin (pacreatic islet betacell)	synthetic	experimental		NA	0.24	2.51	0.35	1.03
01500902	SODIUM DEOXYCHOLATE	100122-25	H10	sample	302-95-4	C24H39NaO4	414.56583	choleretic	bile constituent	experimental		NA	-0.61	0.40	-0.70	-0.31
01501194	TOLPERISONE HYDROCHLORIDE	100122-25	H11	sample	70312-00-4	C16H24ClNO	281.82878	muscle relaxant (skeletal)	synthetic; N-553	INN, BAN, JAN		NA	1.26	1.74	0.26	1.09
	empty	100122-25	H12	sample								NA	NA	NA	NA	NA
