# Supplementary material for: Methotrexate Is a JAK/STAT Pathway Inhibitor
Source: PLoS One. 2015 Jul 1;10(7):e0130078. doi: 10.1371/journal.pone.0130078 (PMC4489434; doi:10.1371/journal.pone.0130078)
Supplement: S2 Table — Mean 6x2xDrafLuc JAK/STAT pathway reporter activity (expressed as z-scores) following knockdown of the indicated Drosophila genes and stimulation with the three ligands Unpaired, Unpaired2 and Unpaired3. Knockdown of enzymes central to the folate, purine and thymidine synthesis pathways do not interact significantly in any assay while knockdown of hopscotch, the Drosophila JAK homologue, produces a strong and consistent reduction in activity. Each screen was carried out in triplicate and scores shown are the mean of three replicates. Scores above +3 or below -3 are considered significant. Human homologues and their enzymatic activities are shown. (PDF) [file pone.0130078.s003.pdf]

S2 Table

Symbol	*Drosophila* Gene Name	Unpaired	Unpaired2	Upaired3	Human homologue	Enzymatic Activity
Dhfr	*Dihydrofolate reductase*	-0.59	1.23	0.07	DHFR	dihydrofolate reductase
pug	*pugilist*	-0.21	0.84	0.15	MTHFD1	methylenetetrahydrofolate dehydrogenase (NADP+ dependent) 1, methenyltetrahydrofolate cyclohydrolase, formyltetrahydrofolate synthetase
Prat2	*Phosphoribosylamidotransferase 2*	**0.46**	**0.25**	**-0.79**	PPAT	phosphoribosyl pyrophosphate amidotransferase
ade3	*adenosine 3*	**0.05**	**-0.36**	**-0.35**	GART	phosphoribosylglycinamide formyltransferase, phosphoribosylglycinamide synthetase, phosphoribosylaminoimidazole synthetase
CG11089	*CG11089*	**0.10**	**0.44**	**1.00**	ATIC	5-aminoimidazole-4-carboxamide ribonucleotide formyltransferase/IMP cyclohydrolase
Ts	*Thymidylate synthetase*	-0.42	-0.71	-0.79	TYMS	thymidylate synthetase
hop	*hopscotch*	**-5.74**	**-4.56**	**-5.52**	JAK2	tyrosine kinase

6x2xDrafLuc JAK/STAT pathway reporter activity (expressed as z-scores) following knockdown of the indicated *Drosophila* genes and stimulation with the three ligands Unpaired, Unpaired2 and Unpaired3. Knockdown of enzymes central to the folate / purine and thymidine synthesis pathways do not interact significantly in any assay while knockdown of *hopscotch,* the *Drosophila* JAK homologue, produces a strong and consistent reduction in activity. Each screen was carried out in triplicate and scores shown are the mean of three replicates. Scores above +3 or below -3 are considered significant. Human homologues and their enzymatic activities are shown.
